# Harnessing large language models (LLMs) for candidate gene prioritization and selection

**DOI:** 10.1186/s12967-023-04576-8

**Published:** 2023-10-16

**Authors:** Mohammed Toufiq, Darawan Rinchai, Eleonore Bettacchioli, Basirudeen Syed Ahamed Kabeer, Taushif Khan, Bishesh Subba, Olivia White, Marina Yurieva, Joshy George, Noemie Jourde-Chiche, Laurent Chiche, Karolina Palucka, Damien Chaussabel

**Affiliations:** 1 The Jackson Laboratory for Genomic Medicine, Farmington, CT USA; 2 The Rockefeller University, New York, NY USA; 3 INSERM UMR1227, Lymphocytes B et Autoimmunité, Université de Bretagne Occidentale, Brest, France; 4 Service de Rhumatologie, CHU de Brest, Brest, France; 5 Sidra Medicine, Doha, Qatar; 6 Service de Néphrologie, Hôpital de La Conception, Marseille, France; 7 Service de Médecine Interne, Hôpital Européen, Marseille, France

**Keywords:** Transcriptomics, Erythroid cells, Feature selection, Large language models, Generative artificial intelligence

## Abstract

**Background:**

Feature selection is a critical step for translating advances afforded by systems-scale molecular profiling into actionable clinical insights. While data-driven methods are commonly utilized for selecting candidate genes, knowledge-driven methods must contend with the challenge of efficiently sifting through extensive volumes of biomedical information. This work aimed to assess the utility of large language models (LLMs) for knowledge-driven gene prioritization and selection.

**Methods:**

In this proof of concept, we focused on 11 blood transcriptional modules associated with an Erythroid cells signature. We evaluated four leading LLMs across multiple tasks. Next, we established a workflow leveraging LLMs. The steps consisted of: (1) Selecting one of the 11 modules; (2) Identifying functional convergences among constituent genes using the LLMs; (3) Scoring candidate genes across six criteria capturing the gene’s biological and clinical relevance; (4) Prioritizing candidate genes and summarizing justifications; (5) Fact-checking justifications and identifying supporting references; (6) Selecting a top candidate gene based on validated scoring justifications; and (7) Factoring in transcriptome profiling data to finalize the selection of the top candidate gene.

**Results:**

Of the four LLMs evaluated, OpenAI's GPT-4 and Anthropic's Claude demonstrated the best performance and were chosen for the implementation of the candidate gene prioritization and selection workflow. This workflow was run in parallel for each of the 11 erythroid cell modules by participants in a data mining workshop. Module M9.2 served as an illustrative use case. The 30 candidate genes forming this module were assessed, and the top five scoring genes were identified as BCL2L1, ALAS2, SLC4A1, CA1, and FECH. Researchers carefully fact-checked the summarized scoring justifications, after which the LLMs were prompted to select a top candidate based on this information. GPT-4 initially chose BCL2L1, while Claude selected ALAS2. When transcriptional profiling data from three reference datasets were provided for additional context, GPT-4 revised its initial choice to ALAS2, whereas Claude reaffirmed its original selection for this module.

**Conclusions:**

Taken together, our findings highlight the ability of LLMs to prioritize candidate genes with minimal human intervention. This suggests the potential of this technology to boost productivity, especially for tasks that require leveraging extensive biomedical knowledge.

**Supplementary Information:**

The online version contains supplementary material available at 10.1186/s12967-023-04576-8.

## Background

Systems-scale profiling technologies are unbiased, simultaneously measuring all analytes in a biological system. Transcriptomics, for instance, uses RNA-sequencing to simultaneously quantify tens of thousands of RNA species [1, 2]. The introduction of such capabilities over two decades ago was transformational and resulted in significant advances across a wide range of medical fields [3, 4], notably in oncology for risk stratification [5, 6] and in autoimmunity to elucidate the pathogenesis of diseases like SLE [7, 8]. However, while systems/omics profiling offers tremendous advantages, there is also a need to identify relevant analyte panels and design targeted profiling assays.

Targeted transcriptional profiling assays enable precise, quantitative assessments of the abundance of panels comprising tens to hundreds of transcripts [9, 10]. Owing to their simplicity, cost-effectiveness, and rapid turnaround times, these assays carry substantial potential for both research and clinical endeavors. In research scenarios, targeted assays firstly provide the advantage of enabling the processing of large number of samples. This can be critical in studies conducted with large patient cohorts, especially when incorporating a longitudinal component [11–13]. Secondly, these streamlined targeted profiling assays could also prove more suitable in resource-constrained research environments, such as in developing countries. Finally, from a translational perspective, targeted assays can assist in discovering biomarkers, evaluating drug responses, and monitoring treatments, while offering a path towards development and validation of novel diagnostic modalities.

The critical task of selecting relevant candidate genes for inclusion in targeted assays can be guided by both prior knowledge and analysis of existing omics data. As demonstrated in our previous work, we leveraged a combination of data- and knowledge-driven strategies to develop a targeted transcriptional panel for monitoring immune responses to SARS-CoV-2 infection [14]. This selection process relied on the well-established BloodGen3 fixed blood transcriptome repertoire, which we employed as a framework for data-driven identification of candidate gene pools [17]. By leveraging reference blood transcriptome datasets from SARS-CoV-2 infected subjects, 23 distinct sets of modules comprising co-expressed genes were identified. To finalize the selection of the gene panel from these pools of candidates, which included 3176 transcripts in total, we used a knowledge-driven approach. Utilizing functional profiling tools, knowledge bases, and expert curation, we were able to prioritize three "themed" panels of 23 genes each, which were categorized based on their relevance to SARS-CoV-2 biology, immunological relevance, or therapeutic relevance.

Employing knowledge-driven in addition to data-driven approaches, as we have done as part of this prior work, is valuable since it can enhance the interpretability of assay results and provide insights into mechanisms of action and potential therapeutic pathways. However, as was also the case in our study, the pools of candidates obtained through systems-scale profiling approaches may include thousands of genes, representing a large volume of associated literature, making the curation process potentially lengthy and possibly lacking in depth. And while resources such as gene ontologies and curated pathways can help, they often provide only superficial information about the genes and may lack context. Recently introduced large language models (LLMs) hold significant potential for improving the utilization of collective biomedical knowledge, and this innovative approach may offer a more efficient means of assimilating and synthesizing the extensive, context-rich information necessary for effective gene curation and analysis. Thus, we decided here to explore the use of generative LLMs to assist with prioritizing pools of co-expressed genes that form modules of potential clinical and biological relevance—with the development of a generic immune profiling Targeted Fingerprinting Assay (ImmP-TFA) as our ultimate goal. We compared the performance of GPT-3.5, GPT-4 (from OpenAI), Bard (from Google), and Claude (from Anthropic) across various tasks and created a standardized workflow inclusive of validation checks. We applied this workflow to the ongoing prioritization of 11 BloodGen3 modules that encompass the module aggregate A37. This meta-signature has been correlated with respiratory syncytial virus (RSV) infection severity [15], vaccine response [16], and elevated abundance of transcripts in patients with metastatic melanoma [15]. Our results demonstrate that LLMs can effectively prioritize large candidate gene pools for inclusion in targeted biomarker panels with minimal human input. Notably LLMs were also able to interpret and factor in reference transcriptional data when tasked to pick a top candidate for a given module.

## Methods

### BloodGen3 module repertoire

The construction and characterization of the BloodGen3 repertoire has been described in detail previously [17]. Briefly, we used as input a collection of 16 reference datasets encompassing 985 unique blood transcriptome profiles, representing 16 disease and physiological states, including infectious and autoimmune diseases, pregnancy, transplantation. Co-clustering patterns were identified and served as a basis for the constitution of a weighted network, from which densely inter-connected networks (modules or cliques) were identified. Modules were subsequently grouped into aggregates, based on patterns of transcript abundance observed across the 16 datasets. It is possible thus to obtain two tiers of dimension reduction: at the module (382 variables) or module aggregate level (38 variables).

### Large language models

ChatGPT-3.5 (OpenAI, San Francisco, CA, https://openai.com/). Description provided by GPT-3.5: “The Generalized Pre-training Transformer 3.5 (GPT-3.5) is an advanced language model. Its primary objective is to comprehend and generate human-like text. Leveraging unsupervised learning, GPT-3.5 is trained on a diverse dataset sourced from internet text, ensuring comprehensive exposure to various linguistic patterns and contexts. As of its last training cut-off in September 2021, GPT-3.5 does not integrate new information, and its responses are solely derived from the knowledge acquired during the training period.”

ChatGPT-4 (OpenAI, San Francisco CA, https://openai.com/). Description provided by GPT-4: “The Generalized Pre-training Transformer 4 (GPT-4) is similar to its predecessor GPT-3, as it uses unsupervised learning and is trained on a diverse range of internet text. However, GPT-4 has more parameters, enabling it to generate more coherent and contextually relevant outputs. As of its last training cut-off in September 2021, the model does not incorporate new data or information, and its responses are purely based on patterns and information it has learned during the training period”.

Bard (Google, Mountain View, CA, https://bard.google.com/). Description provided by Bard: “Bard is a LLM chatbot developed by Google AI. It is trained on a massive dataset of text and code, which includes books, articles, code repositories, and other forms of text data. Bard is able to communicate and generate human-like text in response to a wide range of prompts and questions. For example, Bard can provide summaries of factual topics, create stories, translate languages, and answer questions in an informative way.”

Claude (Anthropic, San Francisco, CA, https://www.anthropic.com/). Description provided by Claude: “Claude a large language model trained using a technique called Constitutional AI, which refers to the use of governance frameworks, aiming to ensure that AI systems operate within the established rules and principles. Claude contains 11 billion parameters and was trained on a large dataset of web data.”

### Candidate gene prioritization and selection workflow

The stepwise prompting strategy employed for prioritizing and selecting candidate genes is described below. The numerical and textual outputs generated by the models are available in Additional File 1 for module M9.2, which serves as our use case.

#### Selecting one of the A37 modules (Step 1)

Participants to the data mining workshop were first tasked to select a module from the set of 11 modules comprised in the BloodGen3 A37 aggregate.

#### Identifying functional convergences among the pool of candidate genes (Step 2)

The following prompts were designed and input to the LLMs via their respective chat interfaces.

PROMPT 2.1: Could you identify functional convergences among this set of genes?

[provide symbols for genes constituting the module].

PROMPT 2.2: Could you generate a R script to visualize these relationships as a network, with nodes representing themes and genes, and edges representing functional associations between genes and themes and among genes?

#### Scoring each candidate gene across multiple criteria (Step 3)

In this study, “prompt engineering” played a critical role in harnessing the capabilities of LLMs for candidate gene prioritization. Here a key aspect of our approach consisted in requesting LLMs to score on a scale from 0 to 10 each gene against a series of statements. Those statements were structured around three primary themes: (i) Relevance as a biomarker, (ii) Therapeutic relevance, and (iii) Biological significance. For benchmarking purposes, themes could be explored through two types of statements—some were explicit and direct (e.g., 'the gene is associated with erythroid cells or erythropoiesis'), while others allowed for inferencing (e.g., 'the gene has potential value as a blood transcriptional biomarker'). Each statement was scored independently, obviating the need for a specific order in the prompts. Although we considered weighted scoring based on the perceived importance of each theme, we opted for equal weighting across all themes to align with the translational focus of this project. It is worth noting that the statements were optimized through several iterations. Some of the statements will also be tailored for specific module aggregates (e.g. erythroid relevance in this case, that is more specifically pertinent for module aggregate A37).

PROMPT 3.1: I am next going to ask for a given gene to:

1. Provide the gene’s official name
2. Provide a brief summary of the gene’s function.
3. Give each of the following statements a score from 0 to 10, with 0 indicating no evidence and 10 indicating very strong evidence:
4. The gene is associated with erythroid cells or erythropoiesis.
5. The gene is currently being used as a biomarker in clinical settings.
6. The gene has potential value as a blood transcriptional biomarker.
7. The gene is relevant to circulating leukocytes immune biology.
8. The gene is a known drug target.
9. The gene is therapeutically relevant for immune-mediated diseases.

Scoring criteria:

- 0—No evidence found.
- 1–3—Very limited evidence.
- 4–6—Some evidence, but needs validation or is limited to certain conditions.
- 7–8—Good evidence, used or proposed for some clinical applications.
- 9–10—Strong evidence, firmly established as a useful biomarker.

For scores of 4 or above please provide an evaluative comment and up to three key supporting references using as a format: First author, Title, Date, Journal.

The results should be generated in the following format, using | as a delimiter and on a single line:

Gene symbol | Gene name | a brief summary | evaluative comment for statement a | supporting references for statement a | score for statement a | and so on for statements b, c, d, e and f.

PROMPT 3.2: Just to give an idea of what the output should look like, here is an example for the gene GPX4: GPX4 | Glutathione Peroxidase 4 | The GPX4 gene provides instructions for making an enzyme called glutathione peroxidase 4. This enzyme is involved in protecting cells from oxidative damage by neutralizing harmful molecules called reactive oxygen species. Specifically, GPX4 is crucial in preventing lipid peroxidation, a process damaging cell membranes. | There is limited evidence that GPX4 has direct relevance to erythroid cells or erythropoiesis. | No specific references found | 2 | GPX4 is not currently widely used as a biomarker in clinical settings, but there is research suggesting potential uses in the future. | "Wang, Glutathione peroxidase 4 and vitamin E cooperatively prevent hepatocellular degeneration, 2020, Redox Biology" | 4 | GPX4 is potentially valuable as a blood transcriptional biomarker due to its role in oxidative stress response. However, more research is needed for validation. | "Banning, Glutathione Peroxidase 4: A new player in neurodegeneration?, 2018, Molecular Neurobiology" | 5 | GPX4 has some relevance to circulating leukocytes immune biology due to its antioxidant function, though it's not the primary focus in this context. | "Iuchi, Glutathione Peroxidase 4 Overexpression Inhibits ROS-Induced Cell Death in Diffuse Large B-cell Lymphoma, 2017, Lab Invest" | 4 | GPX4 has been identified as a possible drug target, especially in the field of cancer biology where ferroptosis—a form of cell death that GPX4 inhibits—is being explored. | "Yang, Regulation of ferroptotic cancer cell death by GPX4, 2014, Cell" | 7 | The gene's role in antioxidant responses and lipid peroxidation can make it relevant for immune-mediated diseases, but it's not a primary target at this point. | "Friedmann Angeli, Inactivation of the ferroptosis regulator Gpx4 triggers acute renal failure in mice, 2014, Nature Cell Biology" | 6 |

PROMPT 3.3: Now go ahead with the evaluation of this gene: *[provide gene symbol].*

The output was recorded in a text file. It was parsed using a simple R script which read the pipe symbol | as a delimiter.

#### Prioritizing candidate genes and summarizing justifications (Step 4)

Parsed scores generated by GPT-4 and Claude obtained in the previous step were saved in a shared Google Sheets spreadsheet (Additional File 1 is the MS Excel version of this spreadsheet). Averages were computed and genes rank-ordered based on their cumulative scores. Bar plots and spider plots were generated in Google Sheets.

We next consolidate score justifications of the top 5 genes. For this we utilize the justifications previously furnished by the models in Step 3, when scoring genes against the six specified criteria (relevance to erythropoiesis, clinical biomarker, etc.).

Prompt 4.1 below operates independently for each criterion (e.g. starting with relevance to erythropoiesis), incorporating the justifications generated by GPT-4 and Claude, but for the top five genes only.

PROMPT 4.1 (for each criterion): Could you generate a coherent summary paragraph based on the information provided below? The style needs to be technical, direct and to the point. *[input text is provided here* = *justifications provided by GPT2 and Claude for top 5 genes for one of the criteria; to be repeated for each criterion].*

#### Fact checking justifications and identifying supporting references (Step 5)

Prompt 5.1 is subsequently executed for each statement in the summary generated by Prompt 4.1. It is essential to validate that the provided reference: 1) exists (is identifiable in PubMed), and 2) is relevant (abstract or full text contains information substantiating the statement). If these conditions are met, the PubMed ID is retrieved and inserted into the text. If required, additional or alternate references can be manually identified in PubMed. If no references are found to support a statement, it should be eliminated.

PROMPT 5.1 (for each statement within the summary): Could you fact-check and provide a couple of supporting references for this statement? *[input text is provided here* = *individual statements from the summaries generated by PROMPT1—record PMIDs manually in the text, and later insert references].*

#### Selecting a top candidate gene based on the validated scoring justifications (Step 6)

GPT-4 and Claude were prompted to select one of the top 5 genes as the overall pick for a given module:

PROMPT 6.1: Based on the summary provided below, could you select a top candidate based on: relevance to (1) erythroid cells or erythropoiesis, (2) current use as a biomarker, (3) potential use as a blood transcriptional biomarker, (4) relevance to leukocytes immune biology, (5) being a known drug target, and (6) being of therapeutic relevance for immune mediated diseases? Equal weight should be given to each of those 6 criteria: *[the narratives generated earlier in step 5 are compiled and passed here in the same prompt*]”.

#### Factoring in transcriptome profiling data to finalize the selection of the top candidate gene (Step 7)

In this step transcriptional profiles for the top 5 candidates from three references datasets are submitted to GPT-4 and Claude and these models are prompted to take this new information in consideration to finalize their selection.

Transcriptional profiling data were retrieved for the top 5 genes from three reference datasets and plots were generated in Google sheets. The reference datasets were contributed to the NCBI GEO repository by:

Speake et al. (GSE6042459) [18], which comprises profiles of whole blood and isolated circulating blood leukocytes. The profiles were accessed via a data browsing web application, the Gene eXpression Browser (GXB) “CD2K” collection. This team of investigators analyzed the sequencing libraries on an Illumina HiScan, targeting a read depth of approximately 20 M. They demultiplexed the reads, aligned them to ENSEMBL's human gene models, and quantified the results using HTSeq [19]. Following this, they normalized the read counts utilizing the TMM method from the edgeR package [20]. http://cd2k.gxbsidra.org/dm3/geneBrowser/show/4000098.

Novershtern et al. (GSE24759) [21], which comprises profiles of isolated leukocyte populations and hematopoietic progenitors. The profiles were accessed via a data browsing web application, the Gene eXpression Browser (GXB) developmental immunology collection [22]. For this dataset transcript levels were extracted from data image files using the RMA method [23] with corrections for sample variations via quantile normalization through the Bioconductor R package [24]. Batch effects were mitigated using the ComBat method [25] (Johnson et al., 2007). http://developmentalimmunology.gxbsidra.org/dm3/geneBrowser/show/4000026.

Altman, Rinchai et al. (GSE100150) [17], which comprises bulk blood transcriptional profiles of 16 reference cohorts encompassing 985 transcriptional profiles. The 16 cohorts encompass a wide range of pathological or physiological states, including autoimmune/inflammatory diseases, infectious diseases, transplantation, cancer or pregnancy. Gene expression profiles from whole blood samples, gathered using Illumina HumanHT-12 v3.0 expression BeadChips, were obtained from 16 patient and control groups. Each dataset underwent a preprocessing procedure, which included filtering based on detection levels, normalization using the BeadStudio algorithm, and transformations focusing on fold changes. Final data were represented as the log2 of the calculated fold changes.

A concatenating function was used to generate text that includes the relevant data and metadata. For instance, the following text was generated for BCL2L1:

From the Speake et al. dataset:

- In Human Whole blood, RNA sequencing detected BCL2L1 RNA at a count of 280
- In Human Neutrophils, RNA sequencing detected BCL2L1 RNA at a count of 34
- In Human Monocytes, RNA sequencing detected BCL2L1 RNA at a count of 32
- Etc…

From the Novershtern et al. dataset:

- In Human Basophils, Microarrays detected BCL2L1 RNA at a signal intensity unit of 267
- In Human Naive B cells, Microarrays detected BCL2L1 RNA at a signal intensity unit of 60
- In Human class switching-capable Mature B cells, Microarrays detected BCL2L1 RNA at a signal intensity unit of 66
- Etc…

From the Altman et al. dataset:

- In Human Whole blood, the abundance of BCL2L1 RNA measured by Microarrays differed in patients with B-cell deficiency compared to controls by a Log2 fold change of 0.12379294
- In Human Whole blood, the abundance of BCL2L1 RNA measured by Microarrays differed in patients with chronic obstructive pulmonary disease compared to controls by a Log2 fold change of -0.0691174
- In Human Whole blood, the abundance of BCL2L1 RNA measured by Microarrays differed in patients with acute influenza infection compared to controls by a Log2 fold change of -0.3697682
- Etc…

This text was submitted to GPT-4 and Claude as input using the following stepwise prompting strategy:

PROMPT 7.1: Based on the summary below, please recommend the top candidate gene to include in a targeted blood transcriptional profiling panel. The intent is for the selected gene to be representative of an erythroid cell signature associated with response to mRNA vaccines, severity of RSV infection and that found to be expressed at high levels in patients with metastatic melanoma and in liver transplant recipients. Please weigh the following criteria equally in your recommendation: (1) Relevance to erythroid cells and erythropoiesis, (2) Current use as a biomarker, (3) Potential use as a blood transcriptional biomarker, (4) Relevance to leukocyte immune biology, (5) Status as a known drug target, (6) Therapeutic relevance for immune mediated diseases [the narratives generated earlier in step 5 are compiled and provided as input in the same prompt]”.

PROMPT 7.2: Now that you have provided an initial recommendation based on the summary, take into account the following RNA-sequencing expression data from key immune cell types and whole blood samples: [“expression levels of the five candidate genes are provided next in the form of text”].

Given this additional RNA-seq expression data, does your recommended gene selection remain the same? Please explain which gene you would recommend at this stage and why, incorporating both the summarized information provided earlier and RNA-seq data in your rationale."

PROMPT 7.3 [skip for GPT-4, input exceeds the limit]: Now that you have provided an initial recommendation based on the summary and the RNAseq data, take into account the following microarray expression data from key immune cell types and hematopoietic precursors: [“expression levels of the five candidate genes are provided next in the form of text”].

Given this additional microarray expression data, does your recommended gene selection remain the same? Please explain which gene you would recommend at this stage and why, incorporating the summarized information and RNA-seq data provided earlier, as well as this new microarray data in your rationale."

PROMPT 7.4: Now that you have provided an initial recommendation based on the summary and the immune cells RNAseq and microarray data, take into account the following averaged log2 fold changes in RNA abundance in patient cohorts compared to controls. [“Log2 Fold changes of the five candidate genes are provided next in the form of text”].

Given this additional data, does your recommended gene selection remain the same? Please explain which gene you would recommend at this stage and why, incorporating the summarized information, the leukocytes RNA-seq and microarray data provided earlier, as well as these new patient cohort profiles in your rationale."

PROMPT 7.5: Could you summarize the key conclusions you have drawn from the conversation so far?

## Results

### Exploring the potential utility of LLMs for knowledge-driven candidate biomarker prioritization

**Fig. 1:**
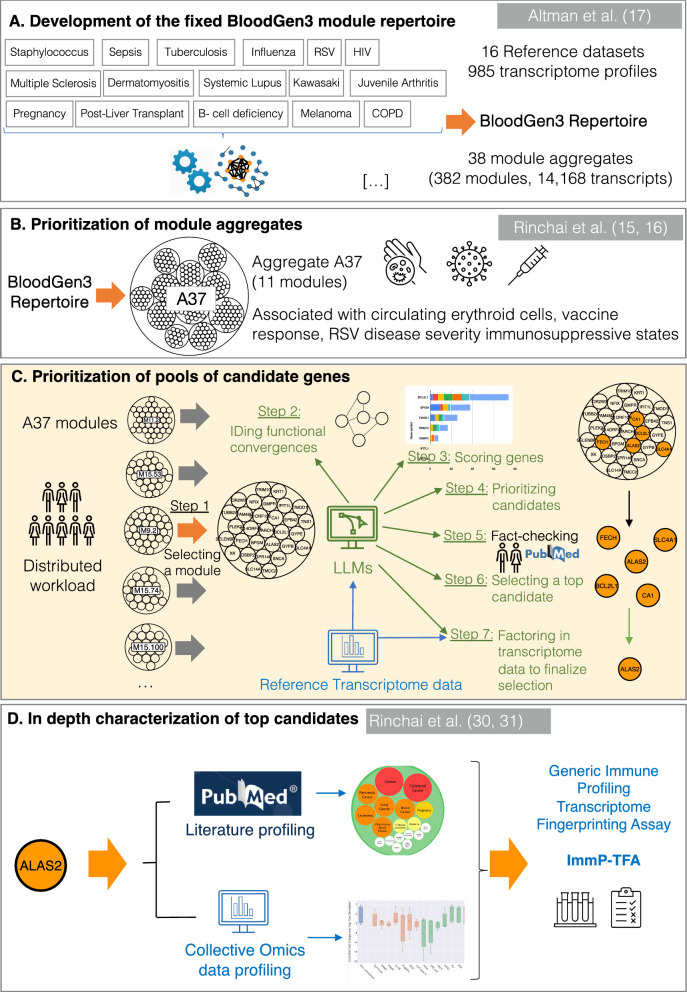
Schematic overview of the targeted panel development strategy. This figure presents our novel workflow for candidate gene prioritization (**C**), within a broader omics data-driven strategy for developing targeted “transcriptome fingerprinting assays” (TFAs). The first component involves data-driven construction of a collection of co-expressed blood transcriptional modules (**A**). This “fixed transcriptional repertoire” provides a stable framework over time for data analysis and interpretation. The BloodGen3 repertoire consists of 382 modules in 38 aggregates representing 14,168 transcripts, constructed and characterized as described in the Methods and a prior publication [17]. Using BloodGen3 in multiple studies provided insight into the potential biological and clinical relevance of its modular signatures (**B**). One signature, corresponding to the module aggregate A37, was associated with circulating erythroid cells, vaccine responses, and respiratory viral infection severity [15, 16], leading to its prioritization for inclusion in a generic Immune Profiling TFA panel (ImmP-TFA). Modules within this aggregate were selected to pilot the novel workflow for the prioritization of candidate gene pools (**C**). In doing so, we investigated the versatility of large LLMs for a range of tasks, from scoring candidates to the selection of top candidates for more comprehensive characterization in a separate workflow (**D**) [30, 31]

We hypothesized that LLMs could mitigate challenges in knowledge-driven curation and prioritization of candidate genes derived from systems-scale profiling data. In our proof of concept, we utilized LLMs to prioritize genes forming a circulating erythroid cell blood transcriptional signature (Fig. 1).

This signature is a component of the BloodGen3 transcriptional module repertoire, which we have recently constructed and characterized [17] (Fig. 1A and see methods for details). Among the 38 module aggregates, aggregate A37 comprised 11 modules, which expression in one of the reference datasets appeared to be restricted to CD71 + Glycophorin A + erythroid cells [15]. It was chosen to be subjected to candidate gene prioritization on the basis of its biological significance and potential clinical relevance (Fig. 1B): (1) we have shown in our previous work this erythroid cell signature to be associated with RSV disease severity [15]; (2) It was also increased in patients with late-stage melanoma [15], which is consistent with reports that have attributed immunomodulatory functions to this cell population [26, 27]; (3) More recently we described pronounced changes in abundance for the transcripts included in this signature following the administration of the second dose of COVID-19 mRNA vaccines [16]; and (4) Others have also found erythroid cell signatures to be associated with COVID-19 [28] and pregnancy [29].

In the current study, we introduce a novel workflow that employs LLMs to prioritize pools of candidate genes, such as those forming the A37 modules (Fig. 1C). We initiated the development of this prioritization workflow by benchmarking four LLMs: OpenAI's GPT-3.5 and GPT-4, Google's Bard, and Anthropic's Claude. Two primary benchmarking tasks were emphasized: (1) identifying functional convergences within the candidate pool to elucidate relationships and biological themes, a staple in interpreting gene lists from transcriptomics, and (2) scoring individual candidates against specific relevance criteria, such as potential utility as a blood biomarker or significance in immune cell biology. The overarching goal was to utilize LLMs for the initial prioritization of candidate genes. Those ranking highest would subsequently undergo rigorous evaluation via our previously described gene-centric workflow [30, 31] (Fig. 1D). Upon thorough characterization, these top candidates would be considered for inclusion in a targeted “Transcriptome Fingerprinting Assay” (TFA). It is important to note that, diverging from our previous work centered on specific diseases like COVID-19 [14], our aim here is to guide the gene prioritization and characterization process toward creating a generic immune profiling TFA panel (ImmP-TFA).

### Benchmarking LLMs on the identification of functional convergences among candidate genes

We first compared the LLMs' ability to identify converging functional themes among genes in a transcriptional module.

We selected the erythroid-restricted A37 module M9.2 from BloodGen3 for benchmarking. While not all 30 genes that constitute module M9.2 are associated with erythroid cells according to existing literature, we anticipated that this would be a significant overarching theme for this gene list. To this end, we utilized a direct prompt: "Could you identify functional convergences among this set of genes? ALAS2 BCL2L1 BPGM C14ORF45 C1ORF128 CA1 EPB42 FAM46C FECH GMPR GPR146 GYPB GYPE IFITL1 KRT1 MARCH8 NFIX OR2W3 OSBP2 PLEK2 SELENBP1 SLC14A1 SLC4A1 SNCA TMCC2 TMOD1 TNS1 TRIM10 TUBB2A XK?" The convergences identified by the various models are detailed in Table 1. Three out of the four models identified erythrocytes or erythropoiesis as one of the themes, with only Google's Bard failing to recognize this relationship. We also conducted tests using established functional tools, such as Ingenuity Pathway Analysis (IPA) and DAVID [32]. Functional annotation clustering performed in DAVID identified "Blood group antigen" as a primary theme (represented by XK, GYPB, SLC14A1, SLC4A1), in addition to erythrocyte development and erythrocyte differentiation (ALAS2, BPGM, SLC4A1). The IPA identified enriched pathways, including Heme biosynthesis. Overall, the LLMs and bioinformatic tools identified the following genes as being associated with erythrocyte biology: ALAS2 (GPT3.5, GPT-4, Claude, DAVID); BPGM (GPT-4, DAVID); CA1 (GPT3.5); EPB42 (GPT3.5, GPT-4); FECH (GPT3.5, GPT-4, Claude); SLC4A1 (GPT3.5, GPT-4, Claude, DAVID); GYPB (GPT3.5, GPT-4); GYPE (GPT3.5, GPT-4); XK (GPT-4, DAVID). PubMed queries using the official gene symbols AND "erythrocytes OR erythroid OR erythropoiesis" confirmed these associations. GPT-4 performed best, correctly associating 8 genes with this central theme, only missing CA1 identified uniquely by GP-T3.5. GPT-3.5 made 7 associations, while Claude made 3 associations.

**Table 1:** List of converging functional themes identified by different LLMs for a co-expression gene set (M9.2), from the BloodGen3 repertoire associated with circulating erythroid cells: ALAS2 BCL2L1 BPGM C14ORF45 C1ORF128 CA1 EPB42 FAM46C FECH GMPR GPR146 GYPB GYPE IFITL1 KRT1 MARCH8 NFIX OR2W3 OSBP2 PLEK2 SELENBP1 SLC14A1 SLC4A1 SNCA TMCC2 TMOD1 TNS1 TRIM10 TUBB2A XK

Model	Functional Themes	Associated genes	LLMs’ Comments
GPT3.5	Hemoglobin synthesis and red blood cell function	ALAS2, CA1, EPB42, FECH, GYPB, GYPE, SLC4A1	These genes are involved in processes related to hemoglobin synthesis, red blood cell membrane stability, and carbon dioxide transport
	Enzymes and metabolic processes:	BPGM, FECH, GMPR	These genes encode enzymes involved in glycolysis, heme biosynthesis, and purine nucleotide biosynthesis, respectively
	Cytoskeletal proteins:	KRT1, TUBB2A	These genes encode proteins involved in maintaining the structure and function of the cytoskeleton
GPT-4	Erythrocyte development and function	ALAS2, BPGM, EPB42, GYPB, GYPE, SLC4A1, XK, FECH	These genes are all involved in processes related to erythrocyte (red blood cell) development, function, or associated conditions. For example, ALAS2 and FECH are involved in heme biosynthesis, a crucial component of hemoglobin. BPGM, EPB42, SLC4A1, GYPB, GYPE, and XK are directly involved in erythrocyte structure or function, and mutations in these genes can lead to conditions like hereditary spherocytosis, elliptocytosis, or blood group antigen variations
	Cytoskeleton and cellular structure	TNS1, TMOD1, TUBB2A	These genes are all involved in cytoskeleton formation and function, which is essential for cell shape, division, and movement
	Ubiquitin–proteasome system	MARCH8, TRIM10	These genes are involved in the ubiquitin–proteasome system, which is essential for protein degradation, cell cycle regulation, and other crucial cellular functions
Claude	Erythrocyte biology or iron/heme metabolism	ALAS2, FECH, SLC4A1	ALAS2 and FECH are both involved in porphyrin/heme biosynthesis. SLC4A1 encodes a major anion exchanger in erythrocytes
	Cytoskeletal or cytoskeleton-associated genes	EPB42, KRT1, TMOD1, TNS1, TUBB2A	These genes encode cytoskeletal proteins like keratins, tubulins, actins
	Protein binding, degradation or trafficking	MARCH8, TRIM10	MARCH8 is an E3 ubiquitin ligase involved in protein degradation. TRIM10 is a RING-finger E3 ubiquitin ligase as well that regulates trafficking of GLUT4 glucose transporters
	Interferon response	IFITL1, SELENBP1	IFITL1 and SELENBP1 are both interferon-stimulated genes, though they have different functions
	phospholipid metabolism or vesicle trafficking	OSBP2, PLEK2	OSBP2 encodes an oxysterol binding protein that regulates lipid metabolism. PLEK2 is involved in regulating vesicle transport
	GPCR biology	GPR146, SNCA	GPR146 is an orphan GPCR. SNCA encodes alpha-synuclein, which is involved in dopamine neurotransmission and signaling
Bard	Production of Heme	ALAS2, FECH	ALAS2 and FECH are both involved in the production of heme, which is an essential molecule for many cellular processes
	Glutathione synthesis	BPGM, GYPB	BPGM and GYPB are both involved in the synthesis of glutathione, which is an antioxidant that protects cells from damage
	Cell signaling	GPR146, OR2W3	GPR146 and OR2W3 are both G protein-coupled receptors that bind to specific ligands and signal to the cell
	Solute carrier genes	SLC14A1, SLC4A1	SLC14A1 and SLC4A1 are both solute carrier genes that transport essential molecules across cell membranes

**Fig. 2:**
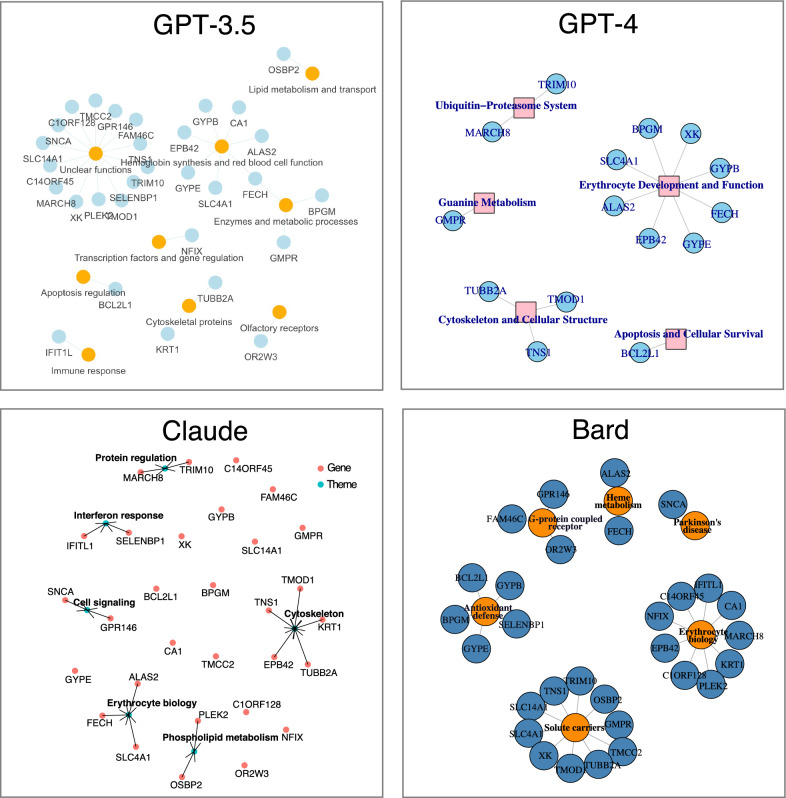
Network representation of M9.2 gene functional convergences identified by four LLMs. Four LLMs were prompted to identify functional convergences among the genes in module M9.2. Each model also generated R scripts to visualize networks of their findings. Nodes represent genes or functional themes, and edges show associations between them

We next tested the models' ability to generate simple network visualizations, a common method for representing functional associations among gene lists. Since the models cannot directly generate images, we prompted them to output R scripts to visualize a network with nodes for themes and genes, and edges for functional associations between them. All four models generated scripts, but only GPT-4's ran error-free and produced the requested network visualization (Fig. 2). Notably, GPT-4 could also successfully troubleshoot and fix the code generated by the other models. And while Bard failed to identify erythrocyte biology as a convergent theme after initial prompting, it did so when requested to generate a network representation. However, of the nine associations Bard made, only two overlapped with those from the other models/tools (CA1, EBP4).

### Benchmarking LLMs on the scoring of candidate genes.

We next tasked the four LLMs with prioritizing candidates based on their functional characteristics and potential clinical relevance.

Our prompting strategy involved presenting the models with statements about each gene and asking them to score and justify their scores for each statement. The models were also prompted to provide references supporting their assessments. To facilitate downstream analysis, we specified the desired format for the model outputs in the prompts. The full text of the prompts is provided in the Methods section. The statements presented to the LLMs were as follows: a. The gene is associated with erythroid cells or erythropoiesis. b. The gene is currently being used as a biomarker in clinical settings. c. The gene has potential value as a blood transcriptional biomarker. d. The gene is relevant to circulating leukocytes immune biology. e. The gene is a known drug target. f. The gene is therapeutically relevant for immune-mediated diseases. We generated scores in triplicates to assess within-model consistency.

**Fig. 3:**
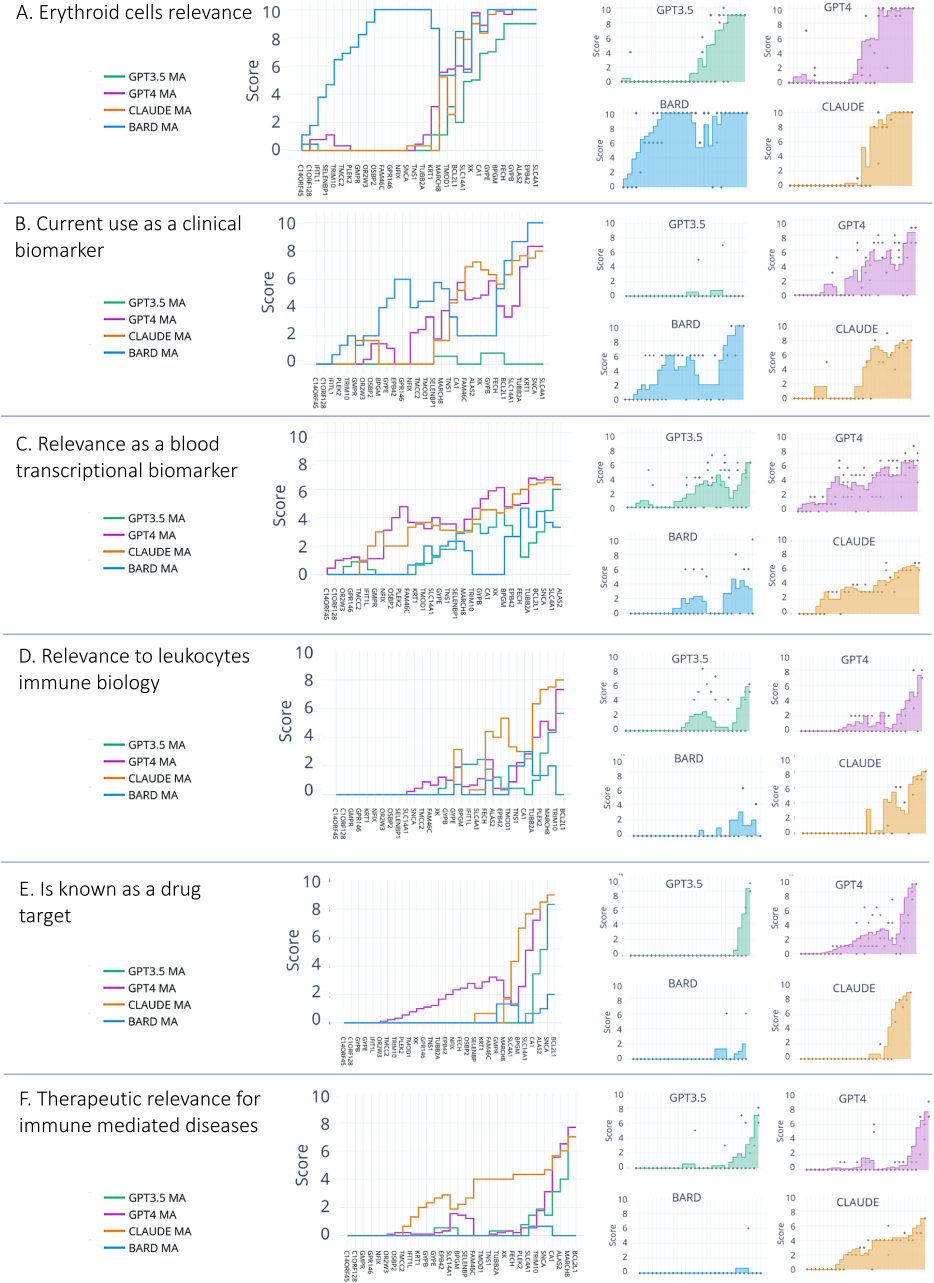
LLMs scoring of M9.2 genes across multiple criteria. Line graphs show scores attributed by four LLMs to 30 genes in module M9.2. Prompts were run in triplicate and scores plotted accordingly. Lines indicate moving averages. Genes on the x-axis were rank-ordered by averaged scores from the four models. Each panel shows scores obtained for one of six statements, which relate to the gene’s: **A** relevance to erythroid cells or erythropoiesis, **B** use as a clinical biomarker, **C** potential as a blood transcriptional biomarker, **D** relevance to leukocytes immune biology, **E** status as a known drug target, and **F** therapeutic relevance for immune mediated diseases. The actual prompts can be found in the methods section (Step 3)

We examined the scores obtained for the M9.2 genes to assess functional convergences identified earlier (Fig. 3). For the first statement probing associations with erythroid cells/erythropoiesis, three of the four LLMs showed substantial convergence. The main discrepancy arose from Google's Bard assigning high scores to genes otherwise scored low by the other models. Examining Bard's justifications for the ~ 14 genes for which scoring discrepancies occurred, we found they did not support the scores produced. For instance, the justification provided for OBP2 was that “OSBP2 mutations have been associated with age-related macular degeneration, a condition that affects the retina.”, or for another gene: “FAM46C mutations are associated with multiple myeloma, a type of cancer of the plasma cells.” And while these statements are factual (e.g. [33]), they do not pertain to erythroid cells or erythropoiesis. We also assessed justifications from the models for "consensus genes" that received high scores from all three models for the first statement. We found these scores to be well-justified. For example, GPT-4 provided this justification for SLC4A1: “SLC4A1 is directly linked to erythroid cells, as it is involved in maintaining the shape and survival of red blood cells. Mutations in this gene can lead to hereditary spherocytosis”. GPT-4 also referenced Perrotta et al. [34], which mentions SLC4A1's role in full text. However, this may be more an exception rather than the rule, as backing references generated by LLMs at this stage were rarely factual. However, in most instances supporting statements could be “manually” linked to actual references. Verifying all score justifications would be too time-consuming at this stage, but we did do this systematically in subsequent steps when focusing on the top five candidates for this module, as will be describe in detail below.

For the next statement (“b. The gene is currently being used as a biomarker in clinical settings.”), Bard appeared to show greater convergence with the other models relative to the previous statement (Fig. 3B). However, GPT3.5 this time produced overall much lower scores than the other three.

**Fig. 4:**
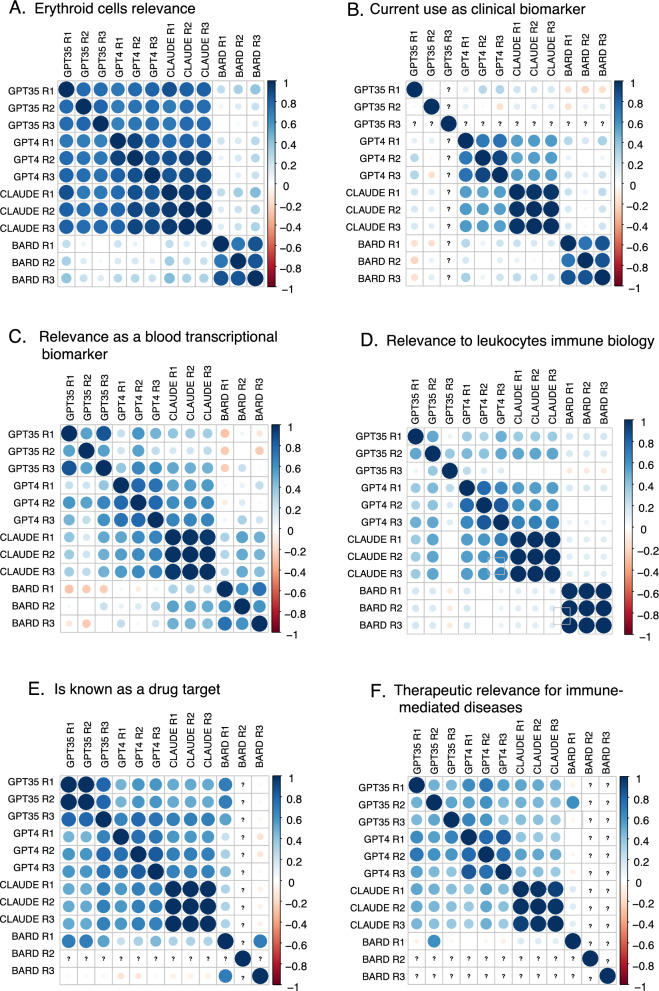
Benchmarking of LLMs on gene scoring tasks. Correlation plots show the degree of similarity between scores generated for a statement by four LLMs: GPT-3.5, GPT-4, Claude and Bard. Scores were generated in triplicate for each model. Plots show scoring similarities and differences within and between models. Each panel shows correlations for scores on a given statement regarding: **A** Relevance to erythroid cells or erythropoiesis. **B** Use as a clinical biomarker. **C** Potential as a blood transcriptional biomarker. **D** Relevance to leukocyte immune biology. **E** Status as a known drug target. **F** Therapeutic relevance for immune-mediated diseases. Actual statements and prompts can be found in the Methods section (Step 3)

We performed correlation analyses to quantify within- and across-model consistency for all six statements (Fig. 4). Overall, we found good level of agreement between GPT-4 and Claude, with high degree of intra-model consistency. Bard and GPT3.5 showed more often discrepancies and, especially in the case of GPT3.5, poor within-model consistency and overall lower scores, with several instances where all genes received a score of 0. Notably, output generated by Bard and GPT3.5 was also more prone to deviate from the specified format, which made working with these models much more difficult.

Collectively, the comparative analyses performed on the four LLMs, across two different tasks, identified GPT-4 and Claude as the most proficient models. Furthermore, the general agreement between these two models on scoring tasks presents an opportunity to cross-validate results, thereby enhancing the robustness of the prioritization process through the combined use of both models.

### Establishing a workflow for distributed candidate genes prioritization across A37 modules

Having determined that at least two of the LLMs performed satisfacorily, we next designed a prioritization workflow aiming to select a top candidate from each of the eleven A37 modules.

To streamline this task, we distributed the workload among participants during a data mining workshop. Each participant selected a specific A37 module to focus on, allowing for parallel processing. We introduced a workflow incorporating the LLMs, guiding participants to select a top candidate for their designated modules and produce a 'prioritization report' (Table 2). It should be noted that the protocol delineated for module M9.2's gene prioritization is consistent across the other ten A37 modules. Comprehensive, sequential guidelines given to the participants are detailed in the methods section.

**Table 2:** List of A37 modules undergoing prioritization

Module ID	N Genes	Gene Symbols	Status	Top pick	Report
M9.2	30	ALAS2, BCL2L1, BPGM, C14ORF45, C1ORF128, CA1, EPB42, FAM46C, FECH, GMPR, GPR146, GYPB, GYPE, IFIT1L, KRT1, MARCH8, NFIX, OR2W3, OSBP2, PLEK2, SELENBP1, SLC14A1, SLC4A1, SNCA, TMCC2, TMOD1, TNS1, TRIM10, TUBB2A, XK	Completed	ALAS2	Additional File 2
M11.2	39	ABCC4, AMFR, BAT3, BMP2K, C17ORF39, C9ORF40, DNAJB2, EIF1B, GDE1, GNA12, HS.211743, HS.57079, IQWD1, JAZF1, MED25, NSUN3, ODC1, POLR1D, PPP2R5B, PSME4, RANBP10, RNF14, RSRC1, SELK, SLC2A1, SNX3, TBC1D22B, TMEM183A, TMEM183B, UBE2F, UBQLN1, WBP2, WDR26, WDR45, WDR51B, WNK1, ZBTB44, ZER1, ZNF653	Completed	WNK1	Additional File 3
M11.3	24	ASCC2, C18ORF10, C18ORF10, DPM2, EPB49, FBXO7, FBXO7, GATA1, HAGH, HEMGN, HEMGN, HMBS, KEL, LOC284422, LOC440359, LOC441081, MBNL3, MBNL3, MYL4, PDZK1IP1, PHOSPHO1, RUNDC3A, SESN3, TESC, TGM2, TRIM10, TSPAN5, VWCE	Completed	HMBS	Additional File 4
M11.4	25	AP2M1, AP2M1, AP2S1, ATG9A, ATG9A, B4GALT3, BCL2L13, C13ORF15, C19ORF62, CYB5R3, DAP, DENND1A, FAM134A, HK1, JUND, MSI2, NTAN1, NUCB1, PA2G4, POLR1D, TCEB2, UBAC1, UBAP1, UBL7, UROD, WDR13, WIPI2, ZMAT2	Completed	MSI2	Additional File 5
M12.11	24	ALDH5A1, BNIP3L, BNIP3L, C20ORF108, DNAJA4, FAM104A, HBD, HS.105618, HS.291319, ISCA1L, LOC389293, MPP1, MXI1, PRDX2, PRDX2, RAB2B, RAD23A, RIOK3, RPIA, SIAH2, SLC1A5, TFDP1, TFDP1, TRAK2, TSTA3, UBE2O, YOD1	Completed	HBD	Additional File 6
M13.26	40	AP2A1, ATP6V0C, BMP2K, C16ORF35, C19ORF22, C2ORF24, C9ORF78, CCDC23, CREG1, EIF2AK1, ELOF1, FAM100A, FOXO4, FURIN, GABARAPL2, GCLC, GPX1, H1F0, HDGF, ISCA1, ISCA1, LYL1, MAF1, MKRN1, NINJ2, NP, PIM1, PINK1, PNPLA2, PRR6, PSMF1, RFESD, RNF123, RNF14, STK33, STOM, SYT15, TERF2IP, TFDP2, XPO7, YPEL3	Completed	SLC2A1	Additional File 7
M13.30	32	ABCC13, ADIPOR1, ARL4A, BLVRB, BOAT, C16ORF35, CARM1, CES3, CSDA, FBXO9, FIS1, FKBP8, FLJ20489, GLUL, GSPT1, GUK1, GYPC, HAGH, HBM, HBQ1, HPS1, HPS1, PBX1, PTMS, SHARPIN, SLC25A39, ST6GALNAC4, ST6GALNAC4, TMEM86B, UBL7, UBXD1	Completed	PBX1	Additional File 8
M14.53	16	BCL2L1, BSG, CDC34, CHPT1, CHPT1, FHL2, GLRX5, IGF2BP2, KLF1, LOC650832, LOC653778, LOC654103, MAP2K3, MARCH8, RBM38, RIOK3, TMEM63B	Completed	KLF1	Additional File 9
M15.53	25	BRD4, C16ORF35, CDKL1, DPM2, EPB41, HMBS, HPS1, IQWD1, KLC3, LOC643008, LOC648434, LOC650898, MAP2K3, MICAL2, MICALCL, MXI1, RAB3IL1, RP11-529I10.4, SLC38A5, SLC6A8, TCP11L2, TGM2, TMPRSS9, TTC25, WNK1	Completed	BRD4	Additional File 10
M15.74	21	ANKRD9, ATP6V0C, C22ORF25, C5ORF4, E2F2, FHL2, HMG2L1, LGALS3, LOC653907, MAP2K3, MARCH2, MCOLN1, MGC13057, PPM1A, PPM1A, RNF10, RNF11, SLC6A10P, SMOX, SRRD, TMEM158, UBE2H	Completed	LGALS3	Additional File 11
M15.100	17	ARHGEF12, C14ORF45, CISD2, CMBL, FLCN, GCAT, GYPE, HBBP1, LOC253012, PCSK1N, PLVAP, RHD, SLC6A9, SPTB, TBCEL, TMEM56, YPEL4	Completed	SPTB	Additional File 12

In summary, the workflow, described in detail below using module M9.2 as an illustrative example, was as follows:

- Step1: Selecting one of the A37 modules.
- Step 2: Identifying functional convergences among the pool of candidate genes.
- Step 3: Scoring each candidate gene across multiple criteria.
- Step 4: Prioritizing candidate genes and summarizing justifications.
- Step 5: Fact checking justifications and identifying supporting references
- Step 6: Selecting a top candidate gene based on the validated scoring justifications.
- Step 7: Factoring in transcriptome profiling data to finalize the selection of the top candidate gene.

In total, seven researchers with a diverse range of expertise and career stages are participating in these activities. Top candidates have been identified for three A37 modules so far, with work still in progress (Table 2).

### Rank scoring of the pool of candidate genes constitutive of a given A37 module

The gene count within A37 modules varies from 18 to 40, and M9.2, the module selected for this use case (Step 1) is comprised of 30 genes (Table 2). Considering the substantial volume of biomedical literature potentially linked to each specific gene, it is crucial to rapidly narrow the pool of candidates to a few top contenders.

The next step thus consisted in identifying functional convergences among the genes constituting the module of interest (Step 2). This could, in some instances, permit to prioritize genes based on their alignment to a relevant functional theme or inform the design of scoring criteria that will be applied next. GPT-4 was used for this task, as the best performing model for this task, as described above as part of LLM benchmarking. And as reported earlier, in the case of M9.2 it identified ALAS2, BPGM, EPB42, GYPB, GYPE, SLC4A1 and XK as being associated with “Erythrocyte development and function” (Table 1, Fig. 2). These could be thus considered as potential candidates, given their alignment with the function attributed to this module in our earlier work (e.g. [15]). Other themes identified by GPT-4 for M9.2 were: Cytoskeleton and cellular structure (TNS1, TMOD1, TUBB2A) and Ubiquitin–proteasome system (MARCH8, TRIM10).

**Fig. 5:**
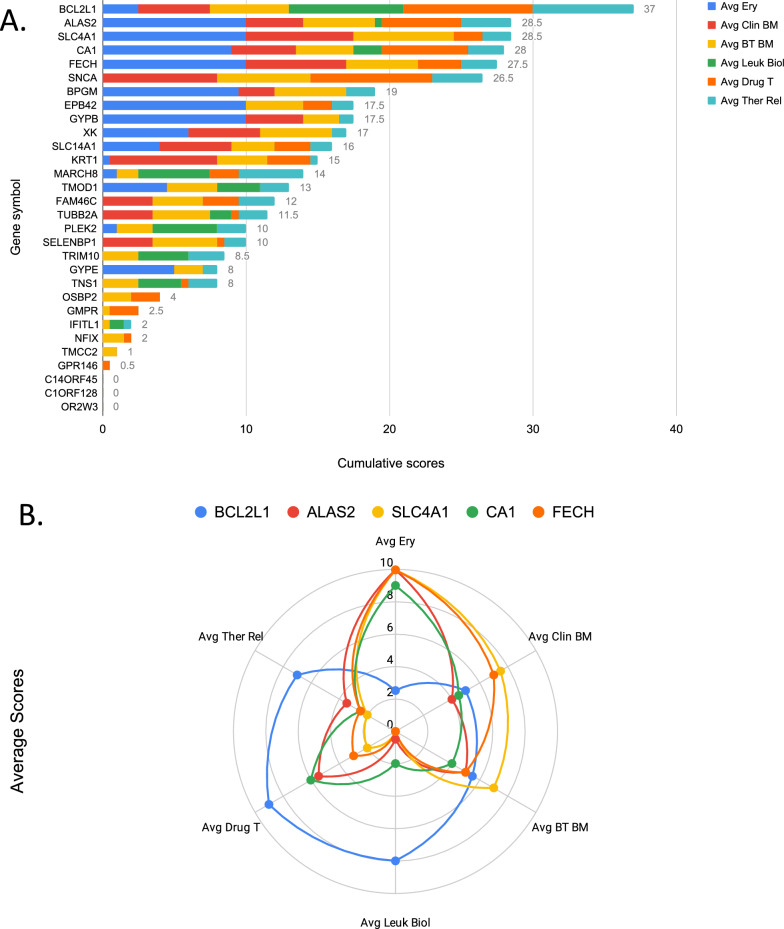
Rank score-based prioritization of M9.2 genes. **A** The stacked bar graph shows cumulative scores across six criteria for the 30 M9.2 genes. **B** The radar plot represents individual scores for the 5 top scoring genes: BCL2L1, ALAS2, SLC4A1, CA1, and FECH

We next prompted GPT-4 and Claude, as described earlier as part of LLM benchmarking, to score genes within a module of interest (Step 3) across the six pre-established criteria (a–f, including relevance to erythroid cells and erythropoiesis, being currently in use as a clinical biomarker, potential relevance as a blood transcriptional biomarker, relevance to leukocytes immune biology, is a known drug target and potential therapeutic relevance for immune-mediated diseases). After parsing of the output, the sum of average scores obtained from the two model were computed, and genes were rank ordered accordingly (Step 4; Fig. 5A). The top five M9.2 genes according to this ranking were BCL2L1, ALAS2, SLC4A1, CA1 and FECH. BCL2L1 received high scores for most categories. It presented with a distinct “scoring profile” compared to the other four high scoring genes from this module (Fig. 5B). Indeed, it received much higher scores for criteria related to therapeutic relevance (criteria e & f) and relevance to leukocyte immune biology (criterion d). It received in comparison lower scores on the criterion prompting its relevance to erythropoiesis (criterion a).

While the identification of functional associations can provide useful insights, we favored the supervised scoring approach for the selection of top candidates that will be considered in the next selection steps.

### Generating a narrative and validating LLMs statements

From the top 5 scoring candidates, we endeavored to select a single gene, which would subsequently be subjected to more in-depth characterization (Fig. 1D). Thus, we next compiled the narratives provided by GPT-4 and Claude to justify the scores they attributed to each of the statements when prompted earlier in Step 3. We then relied on GPT-4 to summarize the statements generated by the models, using the following prompt: “Could you generate a coherent summary paragraph based on the information provided below? The style needs to be technical, direct and to the point.” This was repeated for each of the six criteria. We next relied on GPT-4 and Claude to identify supporting references for each of the statements generated as part of these summaries. The prompt used this time was: “Could you fact-check and provide a couple of supporting references for this statement? “. This was repeated for each statement and verifications were performed “manually” to ensure that the references were real and contained the relevant information. The human-curated references were then added to the summary. Implementing this workflow for module M9.2, our illustrative use case, yielded the following material, which was entirely generated by GPT-4, with supporting references added manually (Step 5):

- Function:

“The BCL2L1, ALAS2, SLC4A1, CA1, and FECH genes play crucial roles in cellular apoptosis regulation, heme biosynthesis, acid-base balance, and overall erythrocyte physiology. The BCL2L1 gene encodes a critical apoptosis-regulating protein in the Bcl-2 family that exists as Bcl-xL and Bcl-xS isoforms with opposing impacts on cell survival [35, 36]. The ALAS2 gene instructs the production of the 5'-aminolevulinate synthase 2 enzyme, essential for initiating heme biosynthesis in red blood cells, with mutations linked to X-linked sideroblastic anemia [37]. The SLC4A1 gene drives the formation of an anion exchanger that modulates intracellular and extracellular pH by orchestrating the exchange of chloride, bicarbonate, and carbonate anions across the cell membrane, an integral process affected in conditions such as hereditary spherocytosis [34, 38]. The CA1 gene directs the production of carbonic anhydrase 1, a cytosolic enzyme that helps maintain tissue and blood pH balance by aiding the conversion of carbon dioxide and water into bicarbonate and protons, primarily in red blood cells [39, 40]. Lastly, the FECH gene governs the expression of ferrochelatase, the terminal enzyme in the heme biosynthesis pathway, with mutations leading to erythropoietic protoporphyria due to protoporphyrin accumulation [41].”

- Statement a—relevance to erythroid cells and erythropoiesis:

“While BCL2L1 plays a general role in controlling cell death, there is no evidence it is specifically associated with erythroid cells or erythropoiesis. In contrast, ALAS2, SLC4A1, CA1, and FECH have critical roles tied to erythroid cell function and erythropoiesis. ALAS2 is highly expressed in erythroid progenitors and is essential for heme biosynthesis, a process vital for red blood cells [42]. SLC4A1 is highly expressed in erythroid cells, where it maintains pH balance and facilitates oxygen transport. Mutations in SLC4A1 can lead to hereditary spherocytosis [43]. CA1 is strongly associated with erythroid cells due to its high expression in red blood cells and role in maintaining their pH balance [44]. FECH is also highly expressed in erythroid cells and is essential for heme biosynthesis during erythropoiesis [45, 46]. In summary, while BCL2L1 has a general role in apoptosis, ALAS2, SLC4A1, CA1, and FECH are integral to erythroid cell physiology and erythropoiesis.”

- Statement b—is used as a clinical biomarker:

“The potential utility of BCL2L1, ALAS2, SLC4A1, CA1, and FECH as clinical biomarkers varies considerably. While BCL2L1 is not commonly utilized as a biomarker in clinical settings, it might be of interest in certain malignancies due to its key role in apoptosis regulation, although further research is warranted [47]. ALAS2 is not extensively used as a clinical biomarker, except as a biomarker and therapeutic target for X-linked sideroblastic anemia [48]. SLC4A1 has clinical relevance as a biomarker for hereditary spherocytosis and as a therapeutic target in sickle cell disease and other red blood cell disorders [49].” CA1, is not broadly used as a biomarker. “Lastly, while FECH is not typically viewed as a 'biomarker' in a conventional sense, its mutations serve as genetic markers for erythropoietic protoporphyria, and its deficiency can be used as a biomarker for this and other porphyrias [50].”

- Statement c—potential relevance as a blood transcriptional biomarker:

“BCL2L1, ALAS2, SLC4A1, CA1, and FECH each exhibit potential utility as blood transcriptional biomarkers in specific clinical contexts. BCL2L1, due to its central role in apoptosis regulation, holds potential as a biomarker for diseases associated with apoptosis dysregulation, including certain cancers [47], albeit further research is necessary. ALAS2, given its fundamental role in heme biosynthesis, could potentially serve as a biomarker for conditions such as X-linked sideroblastic anemia [48], but it is not typically utilized as a blood transcriptional biomarker. SLC4A1, due to its crucial function in red blood cell physiology and its link to hereditary spherocytosis, may serve as a potential blood transcriptional biomarker for this condition [49]. CA1, due to its expression in red blood cells, could potentially be used as a biomarker for conditions impacting pH balance in the blood, although more research is warranted [46]. Lastly, FECH, due to its critical role in heme biosynthesis, could serve as a potential blood transcriptional biomarker for conditions impacting this process, however, more research is needed for its broader application [51].”

- Statement d—relevance to leukocytes immune biology:

“The BCL2L1, ALAS2, SLC4A1, CA1, and FECH genes each have varying degrees of relevance to circulating leukocytes immune biology. BCL2L1, due to its role in apoptosis regulation, is crucial to the lifespan and function of immune cells and is therefore highly relevant to leukocyte immune biology [52, 53]. Conversely, ALAS2, being erythroid-specific, has limited relevance to circulating leukocyte immune biology. There is no significant evidence pointing towards SLC4A1 having relevance to circulating leukocyte immune biology. CA1, despite being expressed in some circulating immune cells like monocytes, only has limited relevance to immune biology. Lastly, FECH does not display any significant relevance to the immune biology of circulating leukocytes, as per the current evidence.”

- Statement e—is a known drug target:

“The BCL2L1, ALAS2, SLC4A1, CA1, and FECH genes are each known to varying degrees as drug targets in clinical medicine. BCL2L1 is particularly significant as a target in cancer therapy, where strategies are being developed to inhibit its anti-apoptotic function [47, 54]. ALAS2 has been targeted for treatment of X-linked sideroblastic anemia, although its broader application as a drug target is currently limited [55–57]. While SLC4A1 could potentially be a drug target for conditions such as hereditary spherocytosis, more research is needed. CA1, as part of the carbonic anhydrase group, has been explored for drug therapy potential, especially considering its role in pH regulation, and inhibitors are currently used or studied for conditions like glaucoma, epilepsy, and cancer [58]. Lastly, while FECH is not a well-documented drug target, there is interest in its potential for treating disorders related to heme biosynthesis, including erythropoietic protoporphyria [59].”

- Statement f—potential therapeutic relevance for immune-mediated diseases:

“The BCL2L1, ALAS2, SLC4A1, CA1, and FECH genes exhibit varying degrees of therapeutic relevance for immune-mediated diseases. BCL2L1, due to its role in regulating apoptosis in lymphocytes, holds potential therapeutic value for immune-mediated diseases [60]. ALAS2, despite its primary role in erythropoiesis, may have limited relevance for immune-mediated hemolytic anemias. As of 2021, the therapeutic relevance of SLC4A1 for immune-mediated diseases remains unclear, although its potential impact on pH or oxygen balance disorders that affect red blood cells may warrant further research [43, 61]. CA1 is not typically a primary target for immune-mediated diseases, but the role of members of the carbonic anhydrase family in managing acid-base balance or bone resorption in such conditions could be of interest [62]. Similarly, while FECH is not a well-documented target for immune-mediated diseases, potential relevance may exist for certain red blood cell disorders or porphyrias, necessitating further investigation [63]. In summary, BCL2L1 is the gene with the clearest therapeutic relevance for immune-mediated diseases among this group, due to its direct role in lymphocyte apoptosis. The other genes have potential connections to immune disease management, but require more research to determine their broader therapeutic relevance in this context.”

Notably, we failed to identify backing references for only one of the LLM-generated statement, which was consequently removed from the summary: “CA1, although not broadly used as a biomarker, has its alterations associated with conditions such as renal carcinoma and primary biliary cirrhosis (PBC), hence it can be utilized as a biomarker for these conditions and erythrocytic disorders albeit with limitations.” And we had to resort to a manual PubMed search to identify appropriate references in only a few instances. Indeed, for the vast majority of statements references provided by GPT-4 and/or Claude were real and proved pertinent.

### Selection of a top candidate gene based on summarized justifications

We next endeavored to select a top candidate from each module. We initially envisioned this task to be performed by the researchers, based on the information generated and summarized by the LLMs. However, we found that GPT-4 and Claude acquitted themselves well of this task and could provide elaborate arguments to justify their choice.

Specifically, we requested from GPT-4 and Claude to each select their top candidate gene for module M9.2 based on the fact-checked summarized justifications generated in Step 5, taking the six scoring criteria into account (Step 6). For this task, we compared the model’s performances when inputting either the fact-checked summaries generated earlier in step 5 or the list of 30 candidate genes forming module M9.2. We refer to these as “stepwise” and “direct” approaches, respectively. The specific prompts and outputs from both models using these two approaches can be found side-by-side in Table 3 (Claude) and Table 4 (GPT-4). Claude selected ALAS2 as the top candidate gene in both instances. However, differences in the depth of the justifications generated employing the stepwise and direct approaches were notable. In particular, in answering the direct prompt Claude omitted information about the relevance of ALAS2 as a clinical biomarker or therapeutic target. GPT-4 selected BCL2L1 as the top candidate based on its evaluation of human verified justifications generated for the top 5 candidates (“stepwise approach”). However, it selected ALAS2 when prompted directly to select genes from the entire M9.2 list (“direct approach”). As can be seen in Table 4, despite the discrepancy the choices were well justified in both instances.

**Table 3:** Output generated by Claude pertaining to the prioritization of genes comprised in M9.2 module using two distinct prompting approaches

Approach	Stepwise prompting	Direct prompting
Prompt	Based on the summary provided below, could you select a top candidate based on: relevance to (1) erythroid cells or erythropoiesis, (2) current use as a biomarker, (3) potential use as a blood transcriptional biomarker, (4) relevance to leukocytes immune biology, (5) being a known drug target, and 6) being of therapeutic relevance for immune mediated diseases? Equal weight should be given to each of those 6 criteria: [all fact-checked statements supported by references from Step 5 were inserted here]	For the list of genes provided below, could you select a top candidate based on: relevance to (1) erythroid cells or erythropoiesis, (2) current use as a biomarker, (3) potential use as a blood transcriptional biomarker, (4) relevance to leukocytes immune biology, (5) being a known drug target, and (6) being of therapeutic relevance for immune mediated diseases? Equal weight should be given to each of those 6 criteria. ALAS2 BCL2L1 BPGM C14ORF45 C1ORF128 CA1 EPB42 FAM46C FECH GMPR GPR146 GYPB GYPE IFIT1L KRT1 NFIX OR2W3 OSBP2 PLEK2 SELENBP1 SLC14A1 SLC4A1 SNCA TMCC2 TMOD1 TNS1 TRIM10 TUBB2A XK MARCH8
Candidate selected	ALAS2	ALAS2
CLAUDE’s preface	Based on the criteria outlined, I would suggest ALAS2 as the top candidate from the genes listed. Here is a summary of my assessment:	After reviewing the provided gene list and weighing the 6 specified criteria equally, I would select ALAS2 as the top candidate. Here is the rationale:
CLAUDE on criterion 1	Relevance to erythroid cells/erythropoiesis: ALAS2 has the most direct relevance, as it encodes an enzyme essential for initiating heme biosynthesis specifically in red blood cells. Mutations are linked to an erythroid condition	Relevance to erythroid cells/erythropoiesis: ALAS2 encodes the rate-limiting enzyme in heme biosynthesis and is highly specific to erythroid cells. It is critical for hemoglobin production
CLAUDE on criterion 2	Current biomarker use: ALAS2 is used as a biomarker and therapeutic target for a red blood cell disorder, X-linked sideroblastic anemia. This is more significant biomarker use compared to the other genes	Current biomarker use: ALAS2 is not routinely used yet as a biomarker
CLAUDE on criterion 3	Potential blood transcriptional biomarker: ALAS2 has clear potential as a biomarker for conditions affecting heme biosynthesis and erythropoiesis, though not yet utilized broadly	Potential as a blood transcriptional biomarker: As an erythroid-specific gene, changes in ALAS2 expression could indicate altered erythropoiesis
CLAUDE on criterion 4	Relevance to leukocyte biology: ALAS2 has limited relevance to leukocytes given its erythroid specificity	Relevance to immune cells: ALAS2 does not have a known role in leukocytes
CLAUDE on criterion 5	Known drug target: ALAS2 is an established drug target for X-linked sideroblastic anemia	Known drug target: ALAS2 is not currently targeted by drugs
CLAUDE on criterion 6	Therapeutic relevance for immune diseases: ALAS2 may have relevance for immune-mediated hemolytic anemias, though therapeutic applications are currently limited	Therapeutic relevance in immune diseases: ALAS2 does not have a demonstrated role
CLAUDE’s conclusive statement	In summary, ALAS2 has the most direct significance for erythroid cells/erythropoiesis, an existing biomarker role, blood biomarker potential, and known drug target status. Though limited in leukocyte relevance and immune disease applications, it appears the strongest candidate based on weighting the criteria equally. Please let me know if you would like me to explain or expand on any part of my assessment	In summary, ALAS2's erythroid-specific expression and critical function in heme/hemoglobin biosynthesis make it the strongest candidate from this list based on the criteria provided. Other genes like FECH and SLC4A1 have some erythroid relevance but ALAS2 is more specific and essential

**Table 4:** Output generated by GPT-4 pertaining to the prioritization of genes comprised in M9.2 module using two distinct prompting approaches

Approach	Stepwise prompting	Direct prompting
Prompt	Based on the summary provided below, could you select a top candidate based on: relevance to (1) erythroid cells or erythropoiesis, (2) current use as a biomarker, (3) potential use as a blood transcriptional biomarker, (4) relevance to leukocytes immune biology, (5) being a known drug target, and (6) being of therapeutic relevance for immune mediated diseases? Equal weight should be given to each of those 6 criteria: [all fact-checked statements supported by references from Step 5 were inserted here]	For the list of genes provided below, could you select a top candidate based on: relevance to (1) erythroid cells or erythropoiesis, (2) current use as a biomarker, (3) potential use as a blood transcriptional biomarker, (4) relevance to leukocytes immune biology, (5) being a known drug target, and (6) being of therapeutic relevance for immune mediated diseases? Equal weight should be given to each of those 6 criteria. ALAS2 BCL2L1 BPGM C14ORF45 C1ORF128 CA1 EPB42 FAM46C FECH GMPR GPR146 GYPB GYPE IFIT1L KRT1 NFIX OR2W3 OSBP2 PLEK2 SELENBP1 SLC14A1 SLC4A1 SNCA TMCC2 TMOD1 TNS1 TRIM10 TUBB2A XK MARCH8
Candidate selected	BCL2L1	ALAS2
GPT-4’s preface	Based on the information provided and weighting all six criteria equally, BCL2L1 (B-cell lymphoma 2-like 1) appears to be the top candidate among the genes described (BCL2L1, ALAS2, SLC4A1, CA1, and FECH). Here's how this gene stacks up against the six criteria:	Based on the given criteria, the top candidate from your provided gene list appears to be ALAS2
GPT-4 on criterion 1	Relevance to erythroid cells or erythropoiesis: BCL2L1 is associated with erythroid cells through its control of apoptosis, though it's not specifically linked to erythropoiesis	Relevance to erythroid cells or erythropoiesis: ALAS2 (delta-aminolevulinate synthase 2) is highly relevant to erythroid cells and erythropoiesis. This enzyme catalyzes the first step in the heme biosynthetic pathway. It's specifically expressed in erythroid cells and mutations in this gene can lead to X-linked sideroblastic anemia
GPT-4 on criterion 2	Current use as a biomarker: Although BCL2L1 is not widely used as a biomarker, it could be of interest in certain malignancies due to its key role in apoptosis regulation	Current use as a biomarker: As of my knowledge cut-off in 2021, ALAS2 is not frequently used as a biomarker. However, changes in its expression or mutations in this gene are associated with erythropoietic diseases
GPT-4 on criterion 3	Potential use as a blood transcriptional biomarker: BCL2L1, due to its role in apoptosis regulation, has potential as a biomarker for diseases associated with apoptosis dysregulation, including certain cancers	Potential use as a blood transcriptional biomarker: Given its specific expression in erythroid cells, ALAS2 could potentially serve as a blood transcriptional biomarker, particularly in conditions affecting red blood cell formation
GPT-4 on criterion 4	Relevance to leukocytes immune biology: BCL2L1 is highly relevant to leukocyte immune biology due to its role in regulating the lifespan and function of immune cells	Relevance to leukocytes immune biology: While not directly involved in leukocyte biology, heme and iron metabolism (in which ALAS2 is involved) can impact immune cell function and overall immune responses
GPT-4 on criterion 5	Being a known drug target: BCL2L1 is significant as a target in cancer therapy, with strategies being developed to inhibit its anti-apoptotic function	Being a known drug target: Although not a direct target for drugs, ALAS2 is the target of gene therapy for X-linked sideroblastic anemia, demonstrating therapeutic relevance
GPT-4 on criterion 6	Therapeutic relevance for immune mediated diseases: BCL2L1 holds potential therapeutic value for immune-mediated diseases due to its role in regulating apoptosis in lymphocytes	Therapeutic relevance for immune mediated diseases: While not directly relevant to immune-mediated diseases, the role of iron and heme metabolism in immune responses and inflammation indirectly ties ALAS2 to immune disease processes. Additionally, alterations in erythropoiesis and red blood cells can influence immune responses and disease progression
GPT-4’s conclusive statement	While the other genes listed also meet some of these criteria to varying extents, BCL2L1 appears to be the best fit when considering all criteria equally	So while not all criteria are strongly met, given the specific criteria and the gene list provided, ALAS2 seems the most balanced candidate. The consideration of ALAS2 as the top candidate assumes that the six criteria are independent and weighted equally; changes in weighting or interactions between criteria may affect this selection. Furthermore, updates in our understanding of gene function, disease associations, or biomarker utility after 2021 could also affect this choice

This finding is noteworthy as it suggests that despite the general skepticism towards LLMs in such research applications—stemming from their inclination to generate fabricated information—they may reliably execute this specific function. Indeed, both models, when prompted directly to select candidates among the 30 genes constituting M9.2 picked ALAS2, which was the #2 ranked genes identified by our stepwise scoring approach. It was also Claude’s top pick based on the evaluation of the fact-checked justification summaries. While our results thus indicate that the models might perform candidate gene prioritization and selection directly, segmenting the evaluation into intermediate phases and collecting supplemental data during these stages (e.g., scores) enhances transparency. This structured approach not only boosts control, allowing for parameter adjustments, but also fosters trust since it facilitates verification of the data, underpinning the decisions taken.

### Leveraging reference transcriptome datasets for the final candidate selection

For module M9.2, GPT-4 and Claude selected different candidates from the top 5 ranked genes. Given this discrepancy, we proceeded to examine if the models could utilize transcriptome profiling data to refine the prioritization of leading candidates (Step 7).

We focused more specifically on three datasets, which measured transcript abundance in isolated cell populations, thus permitting to assess the restriction of the expression of the top 5 candidates.

The first dataset profiling transcript abundance across a wide range of hematopoietic cells and precursors (GSE24759, [21]), is available from our developmental immunology GXB collection (http://developmentalimmunology.gxbsidra.org/dm3/geneBrowser/show/4000026, [22]). The second dataset measuring transcript abundance in monocytes, neutrophils, B-cells, CD4 + T-cells, CD8 + T-cells and natural killer (NK) cells and in whole blood (GSE6042459, [18]). It is accessible via the “CD2K” GXB collection (http://cd2k.gxbsidra.org/dm3/geneBrowser/show/4000098). For additional perspective, a third transcriptome dataset, which measured transcript abundance in whole blood in patients with a wide range of pathological or physiological states was also accessed (GSE100150). This collection of reference datasets was employed for the development of our BloodGen3 module repertoire and has been described in detail previously [17]. The data is available via the CD2K GXB instance (http://cd2k.gxbsidra.org/dm3/geneBrowser/list): “A Transcriptome Fingerprinting Assay for Clinical Immune Monitoring” datasets] and BloodGen3 app (https://drinchai.shinyapps.io/BloodGen3Module/).

**Fig. 6:**
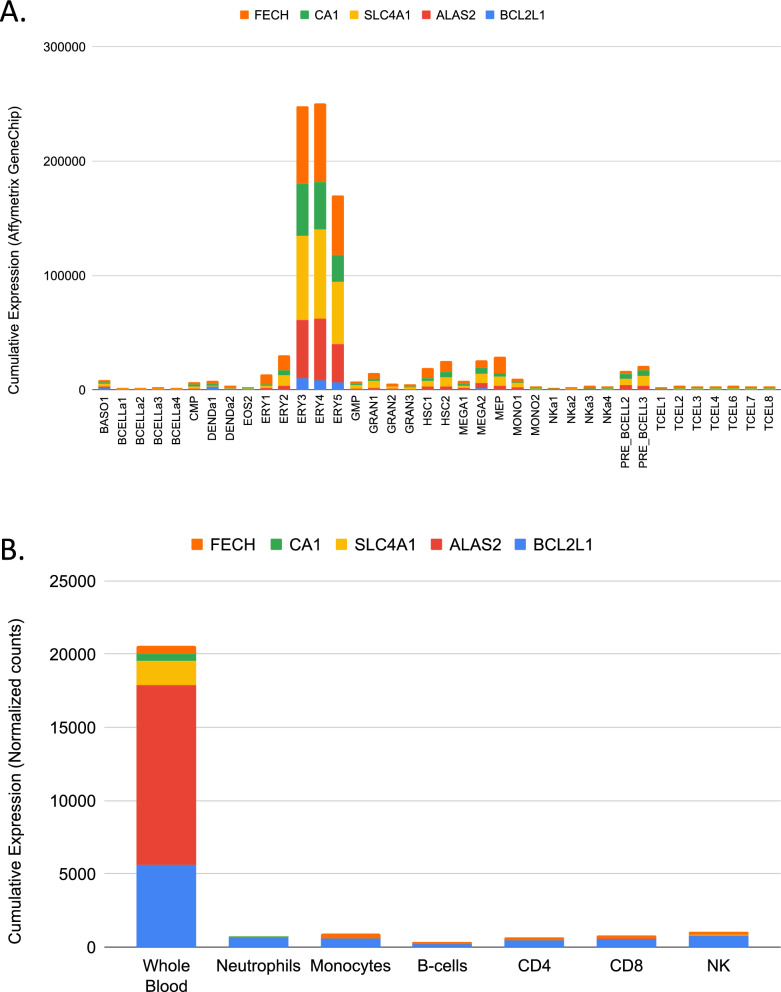
Transcriptional profiles of the top 5 scoring candidate genes in reference leukocyte transcriptome datasets. The stacked bar graphs show levels of transcript abundance for top 5 scoring M9.2 genes, in: **A** a dataset comprising isolated leukocyte and hematopoietic progenitor populations contributed to the NCBI gene expression omnibus (GEO) by Novershtern et al. (GSE24759) and **B** a dataset comprising whole blood and leukocyte populations contributed by Speake et al. (GSE6042459). Abbreviated notations for the Novershtern dataset are as follows: HSC1, Hematopoietic stem cell CD133 + CD34dim; HSC2, Hematopoietic stem cell CD38- CD34 + ; CMP, Common myeloid progenitor; MEP, Megakaryocyte/erythroid progenitor; ERY1, Erythroid CD34 + CD71 + GlyA-; ERY2, Erythroid CD34- CD71 + GlyA-; ERY3, Erythroid CD34- CD71 + GlyA + ; ERY4, Erythroid CD34- CD71lo GlyA + ; ERY5, Erythroid CD34- CD71- GlyA + ; MEGA1, Colony Forming Unit-Megakaryocytic; MEGA2, Megakaryocyte; DENDa1, Plasmacytoid dendritic cell; DENDa2, Myeloid dendritic cell; GMP, Granulocyte/monocyte progenitor; GRAN1, Colony Forming Unit-Granulocyte; GRAN2, Granulocyte (Neutrophilic Metamyelocyte); GRAN3, Granulocyte (Neutrophil); MONO1, Colony Forming Unit-Monocyte; MONO2, Monocyte; BASO1, Basophil; EOS2, Eosinophil; Pre-BCELL2, Early B cell; Pre-BCELL3, Pro-B cell; BCELLa1, Naive B cell; BCELLa2, Mature B cell, able to class switch; BCELLa3, Mature B cell; BCELLa4, Mature B cell, class switched; NKa1, Mature NK cell_CD56- CD16 + CD3-; NKa2, Mature NK cell_CD56 + CD16 + CD3-; NKa3, Mature NK cell CD56- CD16- CD3-; NKa4, NKT cell; TCELL1, CD8 + effector memory RA; TCELL2, Naive CD8 + T cell; TCELL3, CD8 + effector memory cell; TCELL4, CD8 + central memory; TCELL6, Naive CD4 + T cell; TCELL7, CD4 + effector memory cell; TCELL8, CD4 + central memory; Note: NKa1-4 as well as DENDa1 and DENDa2 cells were isolated from adult peripheral blood, other cell populations were isolated from cord blood

**Fig. 7:**
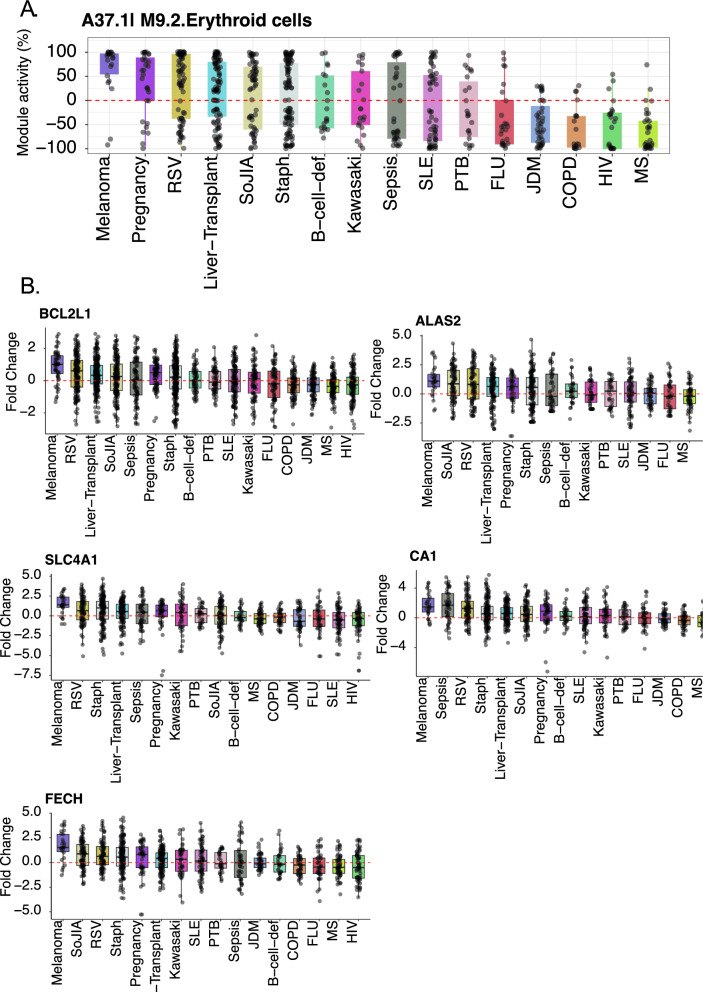
Transcriptional profiles of the top 5 scoring candidate genes across 16 reference blood transcriptome datasets. **A** The box plot shows the changes in abundance of transcripts comprising the M9.2 module, expressed within a cohort as the percentage of genes showing differential expression between each patient and the respective set of control subjects for this cohort. Differential expression is based on a fixed cut-off (Log2 Fold Change > 1.5, difference > 10). These values are expressed as % module activity and can range from + 100% (all genes are upregulated) to -100% (all genes are down regulated). **B** Box plots show expression levels for the top 5 scoring M9.2 genes in patient cohorts relative to respective control subjects matched for demographics variables. The datasets were contributed by Altman, Rinchai et al. (GSE100150) [17]

We first retrieved the transcriptional profiles of BCL2L1, ALAS2, SLC4A1, CA1 and FECH. Abundance profiles of those five genes across the comprehensive set of hematopoietic cells and precursors assembled by Novershtern et al. indicated a high level of restriction of their expression in CD71 + GYPA + erythroid cell populations (ERY3-5 populations) (Fig. 6A). As we have reported previously, it is a characteristic that is common to most genes constituting A37 modules. However, notably, BCL2L1 displayed overall much lower abundance levels in comparison to the other four genes. In the second reference dataset from Speake et al., high levels of expression were detected in whole blood for BCL2L1, SLC4A1 and ALAS2, in comparison to isolated leukocyte populations (Fig. 6B). This is consistent with these genes being expressed by circulating erythroid cells which are present in whole blood but would be excluded upon isolation of various leukocyte populations. Notably, abundance levels observed in whole blood were considerably higher for ALAS2 and to some extent BCL2L1, when compared to SLC4A1, FECH and CA1 levels. The whole blood transcriptome profiles from the BloodGen3 dataset confirmed at the level of module M9.2 that the overall abundance of the genes constituting this module was increased most prominently in the whole blood of subjects with metastatic melanoma, in pregnant women, followed by patients with acute respiratory syncytial virus (RSV) infection and liver transplant recipients (Fig. 7A).  Decreased abundance was most prominently observed in patients with multiple sclerosis (MS), HIV infection, Chronic Obstructive Pulmonary Disease (COPD), Juvenile Dermatomyositis (JDM) and Influenza virus infection.

 Integrating this information into the LLM-driven prioritization and selection process posed a challenge, especially in ensuring that the models received both the data and the essential contextual metadata for interpretation. Given that the main interface for generative LLMs is text we endeavored to convert expression tables into textual input. For this a concatenating function was utilized to automatically generate sentences such as: “In Human Neutrophils, RNA sequencing detected BCL2L1 RNA at a count of 34”, which encapsulates both measurements and parameters such as, measurement method, species, or sample types. Another such generated sentence for the Altman et al. dataset was: “In Human Whole blood, the abundance of BCL2L1 RNA measured by Microarrays differed in patients with acute influenza infection compared to controls by a Log2 fold change of − 0.3697682”. Here we adopted a multi-tier prompting strategy which permitted to factor in altogether the fact-checked summarized justifications obtained earlier in step 5 and the textualized expression values from the three reference datasets. Five separate prompts were designed to carry out this task (see methods for details).

In response to the first prompt, which provided the fact-checked summarized justifications from step 5 as input, the results obtained were similar to those reported earlier for Step 6, with GPT-4 selecting BCL2L1 as its top pick and Claude selecting ALAS2. These selections were maintained despite additional guiding contextual information being provided in the prompt: “Based on the summary below, please recommend the top candidate gene to include in a targeted blood transcriptional profiling panel. The intent is for the selected gene to be representative of an erythroid cell signature associated with response to mRNA vaccines, severity of RSV infection and that found to be expressed at high levels in patients with metastatic melanoma and in liver transplant recipients. Please weigh the following criteria equally in your recommendation*: [*etc.*…]*”. GPT-4 and Claude’s full response to this and subsequent prompts can be found in Table 5.

**Table 5:** Final prioritization prompts and output, factoring in both fact-checked justification summaries and textualized transcriptome profiling data

Prompts	CLAUDE	GPT-4
PROMPT 1: Based on the summary below, please recommend the top candidate gene to include in a targeted blood transcriptional profiling panel. The intent is for the selected gene to be representative of an erythroid cell signature associated with response to mRNA vaccines, severity of RSV infection and that found to be expressed at high levels in patients with metastatic melanoma and in liver transplant recipients. Please weigh the following criteria equally in your recommendation: (1) Relevance to erythroid cells and erythropoiesis, (2) Current use as a biomarker, (3) Potential use as a blood transcriptional biomarker, (4) Relevance to leukocyte immune biology, (5) Status as a known drug target, (6) Therapeutic relevance for immune mediated diseases: *[input summarized and fact-checked justifications from Step 5]*	Based on the provided summary, I would recommend ALAS2 as the top candidate gene to include in the targeted blood transcriptional profiling panel. The key reasons are: •ALAS2 plays a crucial and specific role in heme biosynthesis in erythroid cells, making it highly relevant to erythropoiesis. This aligns closely with the aim to select a gene representative of an erythroid signature •While not yet an established biomarker, ALAS2 has potential utility as a blood transcriptional biomarker for conditions affecting heme biosynthesis like sideroblastic anemia, given its integral involvement •ALAS2 is already an established therapeutic target for a condition affecting erythropoiesis and red blood cells—X-linked sideroblastic anemia. This demonstrates its translational relevance •In contrast, the other genes have limitations regarding erythroid specificity (BCL2L1), lack of broad biomarker potential (CA1, FECH), unclear relevance as a drug target (SLC4A1), or less direct involvement in erythropoiesis (FECH) In summary, ALAS2's crucial and specific role in heme biosynthesis in erythroid cells, translational relevance as a therapeutic target, and potential as a blood transcriptional biomarker make it the top choice to represent an erythroid signature for the intended profiling panel	Based on the provided summary, the most suitable candidate gene to include in the targeted blood transcriptional profiling panel, considering all the specified criteria, appears to be the BCL2L1 gene 1.Relevance to erythroid cells and erythropoiesis: While BCL2L1 does not have direct involvement in erythropoiesis, it plays a crucial role in cellular apoptosis regulation, which affects erythroid cells 2.Current use as a biomarker: Although not widely used, BCL2L1's role in apoptosis regulation can make it an interesting candidate for certain malignancies 3.Potential use as a blood transcriptional biomarker: BCL2L1 holds potential as a biomarker for diseases associated with apoptosis dysregulation, like certain cancers 4.Relevance to leukocyte immune biology: BCL2L1's function in apoptosis regulation is crucial to the lifespan and function of immune cells, making it highly relevant to leukocyte immune biology 5.Status as a known drug target: BCL2L1 is a significant target in cancer therapy, with strategies being developed to inhibit its anti-apoptotic function 6.Therapeutic relevance for immune-mediated diseases: BCL2L1, by regulating apoptosis in lymphocytes, holds potential therapeutic value for immune-mediated diseases While the other genes also have their unique advantages and applications, the BCL2L1 gene is the most versatile across the provided criteria. It is important to note, however, that the utility and effectiveness of BCL2L1 as a representative of an erythroid cell signature will depend on the specific clinical and research contexts, and further research might be necessary to fully confirm its potential in this regard
PROMP 2: Now that you have provided an initial recommendation based on the summary, take into account the following RNA-sequencing expression data from key immune cell types and whole blood samples: *[input textualized transcriptional data].* Given this additional RNA-seq expression data, does your recommended gene selection remain the same? Please explain which gene you would recommend at this stage and why, incorporating both the summarized information* provided earlier and RNA-seq data in your rationale."*	After reviewing the additional RNA-seq expression data, I would maintain my original recommendation of ALAS2 as the top candidate gene to include in the targeted blood transcriptional profiling panel The key reasons are: •The RNA-seq data confirms the erythroid-specific expression of ALAS2, with counts of 616 in whole blood but only 1 in all the leukocyte subsets. This aligns perfectly with the intent to select a gene representative of an erythroid signature •In contrast, the other genes like BCL2L1, SLC4A1, CA1 and FECH exhibit low but detectable expression across leukocyte subsets, indicating they are more broadly expressed across blood cell types •The erythroid-restricted expression of ALAS2 makes it a better biomarker of erythropoiesis compared to the other genes •ALAS2 remains a relevant therapeutic target for disorders of erythropoiesis like sideroblastic anemia, despite the additional RNA-seq data •The RNA-seq data does not provide any evidence to contradict ALAS2's integral role in heme biosynthesis in erythroid cells specifically In summary, the RNA-seq data confirming erythroid-restricted expression of ALAS2 further strengthens the rationale for selecting it as the top candidate gene representative of an erythroid signature for the intended blood transcriptional profiling panel, in line with my original recommendation. The other genes appear more broadly expressed across leukocytes based on the RNA-seq data Copy	With the addition of the RNA-seq data, it's clear that ALAS2 is the most abundantly expressed gene in whole blood with a count of 616. This contrasts with BCL2L1, the initially recommended gene, which exhibits a lower expression level in whole blood (280). Importantly, ALAS2 demonstrates minimal expression in all other tested immune cells (counts of 1), suggesting a strong erythroid cell-specific expression, fitting the criteria to be representative of an erythroid cell signature. BCL2L1, on the other hand, is more uniformly expressed across various cell types, which makes it less specific to erythroid cells Given these findings, I recommend reconsidering the selection and placing ALAS2 as the top candidate gene for inclusion in the targeted blood transcriptional profiling panel. Here's why: 1. Relevance to erythroid cells and erythropoiesis: ALAS2 plays an essential role in heme biosynthesis in red blood cells, a process critical for erythropoiesis 2. Current use as a biomarker: ALAS2 is used as a biomarker and therapeutic target for X-linked sideroblastic anemia 3. Potential use as a blood transcriptional biomarker: Although not typically utilized as a blood transcriptional biomarker, its high expression in whole blood and critical role in erythropoiesis highlight its potential 4. Relevance to leukocyte immune biology: Although it has limited relevance to circulating leukocyte immune biology due to its erythroid-specific nature, its low expression in other immune cells supports its specificity for erythroid cells 5. Status as a known drug target: ALAS2 has been targeted for the treatment of X-linked sideroblastic anemia 6. Therapeutic relevance for immune-mediated diseases: Although it has limited relevance for immune-mediated diseases, it can be relevant for immune-mediated hemolytic anemias 7. By combining both the RNA-seq data and the summary provided earlier, it appears that ALAS2 would be a more appropriate candidate due to its high expression in whole blood and its critical role in erythropoiesis, despite its limited involvement in leukocyte immune biology and use in immune-mediated diseases
PROMPT 3: Now that you have provided an initial recommendation based on the summary and the RNAseq data, take into account the following microarray expression data from key immune cell types and hematopoietic precursors: *[input textualized transcriptional data].* Given this additional microarray expression data, does your recommended gene selection remain the same? Please explain which gene you would recommend at this stage and why, incorporating the summarized information and RNA-seq data provided earlier, as well as this new microarray data in your rationale	After reviewing the extensive microarray expression data, I would still recommend ALAS2 as the top candidate gene for inclusion in the targeted blood transcriptional profiling panel The key reasons are: •The microarray data confirms very high, erythroid-restricted expression of ALAS2 in the GlyA + erythroblast subsets, with values of 8371, 7664, and 5566 signal intensity units. This agrees with the RNA-seq data showing erythroid specificity •ALAS2 expression is low (values < 100) in all immune cell types profiled by microarrays, including T cells, B cells, NK cells, dendritic cells. This further confirms leukocyte-restricted expression •Other genes like BCL2L1, SLC4A1, CA1 and FECH are more broadly expressed across immune cells and progenitors, based on the microarray data •No additional insight from the microarrays contradicts ALAS2's integral involvement in heme biosynthesis in erythroid lineage cells specifically •ALAS2 remains a relevant therapeutic target in erythroid disorders based on its key role, despite the new microarray data In summary, the extensive microarray expression profiling continues to support ALAS2 as the top gene choice representative of an erythroid signature, based on its high erythroid-restricted expression and lack of expression in other immune cells. The other genes appear more broadly expressed across leukocytes. Therefore, I maintain ALAS2 as my recommended top candidate gene for inclusion in the intended targeted blood transcriptional profiling panel Copy	*[N/A: input exceed GPT-4 limits. Attempts to break down inputting the textualized data were unsuccessful as GPT-4 lost the thread of the conversation. Thus, we chose to skip to the next prompt]*
PROMPT 4: Now that you have provided an initial recommendation based on the summary and the immune cells RNAseq and microarray data, take into account the following averaged log2 fold changes in RNA abundance in patient cohorts compared to controls. *[input textualized transcriptional data].* Given this additional data, does your recommended gene selection remain the same? Please explain which gene you would recommend at this stage and why, incorporating the summarized information, the leukocytes RNA-seq and microarray data provided earlier, as well as these new patient cohort profiles in your rationale	After reviewing the log2 fold change data across multiple patient cohorts, I would maintain my recommendation of ALAS2 as the top candidate gene for the intended blood transcriptional profiling panel The key reasons are: • ALAS2 shows some of the largest fold changes across conditions like RSV infection, metastatic melanoma, and juvenile arthritis that were called out in the original summary as being relevant • The magnitude of ALAS2 fold changes are greater than those seen for other genes like BCL2L1, SLC4A1, CA1 and FECH in many of the disease states • ALAS2's erythroid-specific expression makes its fold changes more amenable to interpretation compared to broadly expressed genes • No major contradictory evidence arises from the fold change data to refute ALAS2's important role in erythropoiesis • The leukocyte expression data and key role in heme synthesis remain valid points strengthening ALAS2's selection despite the new cohort profiles In summary, the gene expression fold changes across multiple patient cohorts and conditions continues to support ALAS2 as a top gene choice representative of an erythroid signature that could provide insights into disease biology based on its marked regulation. The other genes do not show as large or as consistent differences across relevant conditions based on the log2 fold change data. Therefore, I maintain ALAS2 as my recommended top candidate gene for the intended blood profiling panel	*[GPT-4 was able to interpret the input transcriptional data but lost the thread of the conversation]* In the analysis, we observed differences in RNA abundance in whole blood for BCL2L1, ALAS2, SLC4A1, CA1, and FECH across various patient groups compared to controls. Let's summarize some of the notable findings: 1.BCL2L1: BCL2L1 expression seems to increase in cases of B-cell deficiency, metastatic melanoma, sepsis, pregnancy, active pulmonary tuberculosis, acute respiratory syncytial virus infection, systemic onset juvenile idiopathic arthritis, staphylococcus aureus infection, and liver transplant recipients. The most pronounced increase was observed in patients with metastatic melanoma. On the other hand, its expression decreases in patients with diseases like chronic obstructive pulmonary disease (COPD), acute influenza, HIV, juvenile dermatomyositis, Kawasaki disease, and multiple sclerosis. The most substantial decrease was observed in patients with HIV infection 2.ALAS2: ALAS2 expression increased in patients with B-cell deficiency, Kawasaki disease, metastatic melanoma, sepsis, pregnancy, active pulmonary tuberculosis, acute respiratory syncytial virus infection, systemic onset juvenile idiopathic arthritis, staphylococcus aureus infection, and liver transplant recipients. The most pronounced increase was seen in patients with metastatic melanoma. However, its expression decreased in patients with COPD, acute influenza, HIV, and juvenile dermatomyositis. The greatest decrease was observed in HIV infection 3.SLC4A1: SLC4A1 expression increased in patients with Kawasaki disease, metastatic melanoma, sepsis, pregnancy, active pulmonary tuberculosis, acute respiratory syncytial virus infection, staphylococcus aureus infection, and liver transplant recipients, with metastatic melanoma showing the highest increase. On the other hand, it decreased in patients with B-cell deficiency, COPD, acute influenza, HIV, juvenile dermatomyositis, and multiple sclerosis. The greatest decrease was observed in patients with HIV infection 4.CA1: CA1 expression increased dramatically in most conditions, especially metastatic melanoma, sepsis, pregnancy, active pulmonary tuberculosis, acute respiratory syncytial virus infection, systemic lupus erythematosus, systemic onset juvenile idiopathic arthritis, staphylococcus aureus infection, and liver transplant recipients. The greatest increase was observed in sepsis patients. However, it decreased in patients with B-cell deficiency, COPD, acute influenza, HIV, and multiple sclerosis. The most significant decrease was seen in HIV patients 5.FECH: FECH expression increased slightly in patients with B-cell deficiency and decreased slightly in patients with COPD, but the changes were less pronounced compared to other genes. The expression decreased in conditions like acute influenza, HIV, and juvenile dermatomyositis, with the most significant decrease observed in patients with HIV In all cases, these changes in gene expression may represent physiological responses to disease or might contribute to the pathophysiology of the disease itself. These findings could be valuable for designing further research, or potentially, diagnostic or therapeutic strategies Please note that the interpretation of gene expression data is complex, with many factors influencing the results, such as patient characteristics, sample handling, and microarray technique variability. Hence, these interpretations should be considered as hypotheses that need further validation
PROMPT 5: Could you please summarize the key conclusions you have drawn from the conversation so far?	Based on reviewing the initial summary, RNA-seq data, microarray data, and patient cohort fold change data provided sequentially, my conclusions are: • I would recommend ALAS2 as the top candidate gene for inclusion in the intended targeted blood transcriptional profiling panel based on its: 1.Crucial, erythroid-specific role in heme biosynthesis, making it a good representation of an erythroid signature 2.Lack of expression in leukocytes and high, restricted expression in erythroblast subsets, confirmed by both RNA-seq and microarrays 3.Large, consistent fold changes across conditions like RSV infection, juvenile arthritis, and melanoma that were highlighted as being relevant 4.Established utility as a therapeutic target in erythroid disorders like sideroblastic anemia 5.Potential as a biomarker for disrupted erythropoiesis based on its tight regulation • The other genes (BCL2L1, SLC4A1, CA1, FECH) are more broadly expressed across immune cells and their fold changes are smaller in magnitude or less consistent across key disease states • Each additional dataset provided further evidence supporting ALAS2 as the top choice over other candidates based on erythroid specificity and associations with conditions of interest In summary, the collective evidence strongly supports ALAS2 as the top gene for inclusion in the intended blood profiling panel to represent an erythroid signature. The key factors are its erythroid-restricted expression and marked regulation across relevant conditions	*[N/A: GPT-4 could not keep the thread of the conversation following the input of large amount of textualized transcriptional profiling data]*

The second prompt provided as input the textualized transcriptional profiling data from the Speake et al. dataset. At the end of the prompt the following request was made: “Given this additional RNA-seq expression data, does your recommended gene selection remain the same? Please explain which gene you would recommend at this stage and why, incorporating both the summarized information provided earlier and RNA-seq data in your rationale”. Claude maintained its initial recommendation of ALAS2 “as the top candidate gene to include in the targeted blood transcriptional profiling panel”. Further stating that “The RNA-seq data confirms the erythroid-specific expression of ALAS2, with counts of 616 in whole blood but only 1 in all the leukocyte subsets. This aligns perfectly with the intent to select a gene representative of an erythroid signature.” (see Table 5 for the full transcript). Notably, the textualized transcriptome data provided at this step led GPT-4 to reconsider its choice of BCL2L1 as its top candidate, selecting ALAS2 instead: “With the addition of the RNA-seq data, it's clear that ALAS2 is the most abundantly expressed gene in whole blood with a count of 616. This contrasts with BCL2L1, the initially recommended gene, which exhibits a lower expression level in whole blood (280). Importantly, ALAS2 demonstrates minimal expression in all other tested immune cells (counts of 1), suggesting a strong erythroid cell-specific expression, fitting the criteria to be representative of an erythroid cell signature. BCL2L1, on the other hand, is more uniformly expressed across various cell types, which makes it less specific to erythroid cells. Given these findings, I recommend reconsidering the selection and placing ALAS2 as the top candidate gene for inclusion in the targeted blood transcriptional profiling panel.” GPT-4 goes on to detail its rationale, to finally conclude: “By combining both the RNA-seq data and the summary provided earlier, it appears that ALAS2 would be a more appropriate candidate due to its high expression in whole blood and its critical role in erythropoiesis, despite its limited involvement in leukocyte immune biology and use in immune-mediated diseases.”

We in turn attempted to input textualized transcriptional profiling data from the Novershtern et al. and Altman et al. datasets. We were not successful with GPT-4, given limits imposed on the amount of information it can receive as input (8192 tokens), which was exceeded by the Novershtern dataset. Breaking the input down in multiple prompts was unsuccessful as GPT-4 lost the thread of the conversation as a result and failed to factor in the summarized justification and the earlier RNAseq dataset in its selection of a top candidate. This was also the case when the Novershtern et al. dataset was omitted and only the Altman dataset provided (Table 5). No such issues were however encountered with Claude, which supports 100,000 tokens of context. We were thus successful in inputting transcriptional profiles for all three datasets and completed all the prompts (Table 5). Prompt 5 stated: “Could you please summarize the key conclusions you have drawn from the conversation so far? “, which Claude answered as follows: “Based on reviewing the initial summary, RNA-seq data, microarray data, and patient cohort fold change data provided sequentially, my conclusions are: I would recommend ALAS2 as the top candidate gene for inclusion in the intended targeted blood transcriptional profiling panel based on its: (1) Crucial, erythroid-specific role in heme biosynthesis, making it a good representation of an erythroid signature, (2) Lack of expression in leukocytes and high, restricted expression in erythroblast subsets, confirmed by both RNA-seq and microarrays, (3) Large, consistent fold changes across conditions like RSV infection, juvenile arthritis, and melanoma that were highlighted as being relevant, (4) Established utility as a therapeutic target in erythroid disorders like sideroblastic anemia, (5) Potential as a biomarker for disrupted erythropoiesis based on its tight regulation. The other genes (BCL2L1, SLC4A1, CA1, FECH) are more broadly expressed across immune cells and their fold changes are smaller in magnitude or less consistent across key disease states. Each additional dataset provided further evidence supporting ALAS2 as the top choice over other candidates based on erythroid specificity and associations with conditions of interest. In summary, the collective evidence strongly supports ALAS2 as the top gene for inclusion in the intended blood profiling panel to represent an erythroid signature. The key factors are its erythroid-restricted expression and marked regulation across relevant conditions.”.

The final conclusive statement generated by Claude is notable in the balance it strikes between the information provided that was derived from knowledge about candidate genes significance and from relevant transcriptional profiling datasets. Overall, the findings obtained in this use case demonstrate the capabilities of LLMs in aiding with rationale target prioritization and selection, with minimal human input.

**Fig. 8:**
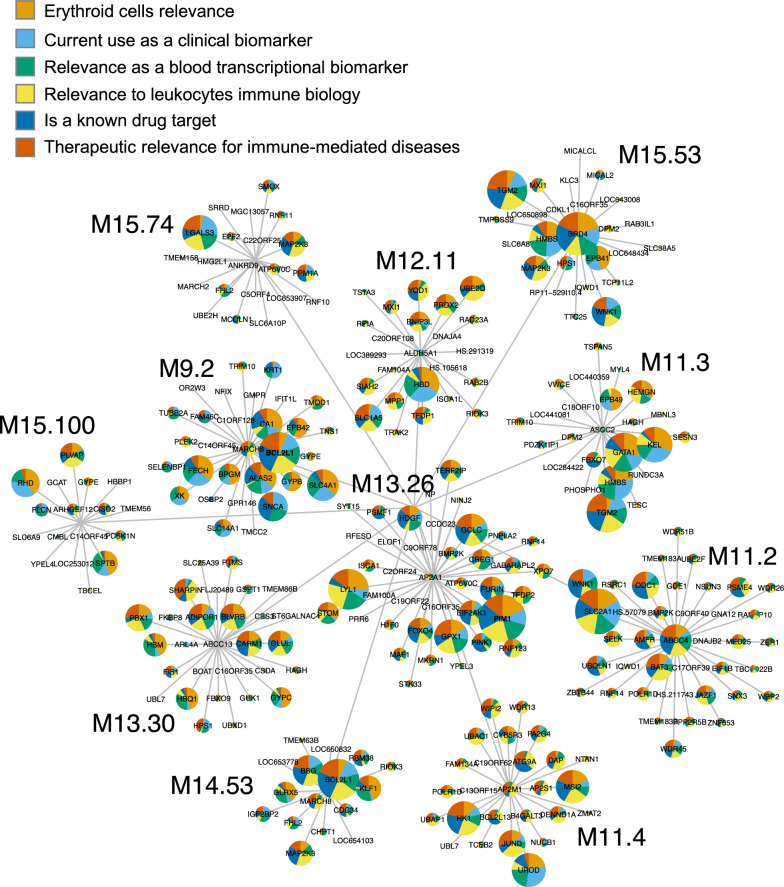
Overview of LLM-generated scores for A37 module genes. This network represents the scores generated by two LLMs, GPT-4 and Claude, for six prespecified criteria, across a pool of candidate genes distributed across 11 BloodGen3 modules from the aggregate A37. Each pie chart shows the relative magnitude of the averaged score from the two models. The size of each chart is proportional to the overall cumulative score across the six criteria

The workflow described above has been implemented in other modules by participants in the data mining workshop. As a result, we were able to parallelize this process and proceed with the selection of candidates from the 11 A37 modules (Table 2). Prioritization reports are available as supplementary material (Additional File 2, Additional File 3, Additional File 4, Additional File 5, Additional File 6 and Additional File 7, Additional File 8, Additional File 9, Additional File 10, Additional File 11, Additional File 12), and LLM scores have been generated for all 11 modules (Fig. 8).

## Discussion

In this study, we aimed to investigate the potential utility of large language models (LLMs) in addressing a significant bottleneck in the knowledge-driven selection of candidate biomarkers derived from systems-scale molecular profiling data. LLMs were successfully integrated into a new candidate gene prioritization workflow situated downstream of an established transcriptional module repertoire construction algorithm [17, 64], and upstream of a gene-centric workflow designed for the in-depth characterization of candidate genes [30, 31] (Fig. 1). LLM tasks ranged from identifying convergences among genes in a circulating erythroid module, to scoring candidates based on specific contextualized criteria, to summarizing justifications, retrieving supporting references, and determining an overall candidate for inclusion in a targeted transcriptional assay measuring a circulating erythroid signature, relying both on the interpretation of biomedical knowledge and transcriptional profiling data. Benchmarking multiple LLMs quickly revealed that not all four models tested could perform these tasks satisfactorily. Indeed, only two models were eventually employed for candidate gene prioritization and selection: GPT-4, the most advanced model from OpenAI, and Claude, a model developed by Anthropic. This decision was made despite certain accessibility limitations, as GPT-4 is available only to "OpenAI plus" subscribers, and at the time of writing, Claude was only accessible in the United States and the United Kingdom. It is worth noting that Anthropic released Claude 2 after our benchmarking was completed. We observed noticeable improvements in both the speed and quality of the output generated by Claude 2, which reaffirmed our decision to utilize both GPT-4 and Claude concurrently when performing these tasks.

One critical aspect of the work was designing suitable “prompting strategies”. One of the key prompts which we devised requested models to provide numerical scores indicating a statement's accuracy for a given gene. It also specified an output format, facilitating subsequent parsing of the large volume of information generated. LMMs proved capable of scoring straightforward statements, like evaluating a gene's association with erythroid cells or erythropoiesis – information which arguably could also at least partly be retrieved from Gene Ontology or pathway enrichment tools, albeit lacking the nuances offered by a 1–10 scoring system. But more interestingly, models could also score more contextualized and nuanced statements, like potential utility as a blood transcriptional biomarker, relevance to leukocyte biology, or therapeutic relevance for immune diseases. In doing so, LMMs performed basic inferencing, for instance linking a gene's erythropoiesis role and biomarker potential, and incorporated these inferences when scoring candidates. The process of designing prompts involved some experimentation, and in some instances, it was "collaborative" as we sought feedback from the models to enhance the clarity and scope of the prompts. We also found that the quality and accuracy of the output generated by the Large Language Models (LLMs) were directly proportional to the extent of the task. For instance, LLMs showed reluctance to identify functional convergences for extensive gene lists (~ > 30) or provided limited justifications when tasked with scoring more than 2–3 genes in a single prompt—which was the maximum number suggested by GPT-4. And as a result, we decided to request the scoring of only one gene per prompt. Perhaps most notably, the supporting references requested while prompting LLMs for scores and justifications on six different statements were seldom factual. However, they were consistently accurate at a later stage when the LLMs were prompted to provide supporting references for specific statements (Step 5). Notably, the phrasing of the prompt was found to be critical for this task. When requested directly to provide relevant references supporting a given statement GPT-4 often declined and explained that it was “beyond its ability as a language model”. But when requested to *fact-check* a statement and provide backing references it readily obliged and acquitted itself of this task well. Another powerful application of LLMs in this workflow was the synthesis of information generated by the models themselves to justify the scores attributed to the top ranked genes. Indeed, after researchers fact-checked these statements—which again involved using LLMs to retrieve backing references—it was possible to utilize these summaries to request the models to identify their top pick. Notably, both GPT-4 and Claude performed this task remarkably well, providing elaborate and logical argumentation to support their choices. Yet, divergences in “opinion” between the two models remained for the pick of the top candidate for M9.2 module, which was settled with the input of transcriptome profiling data, that the models were able to consider in making their final determination, eventually converging to select ALAS2 as the top candidate for this module. This finding is notable as it opens the possibility of generalizing the use of LLMs for biomedical data analysis and interpretation.

While our results are promising, it is important to acknowledge the inherent limitations of these models. As widely reported and experienced here, LLM-generated information is not always factual. As mentioned earlier, when prompted for backing references for the statements generated in association to their scores (Step 3), even the best models largely provided “fake” references. As demonstrated,  instances of information "hallucination" can be addressed through prompt engineering and fact-checking. However these issues, which have been widely reported, present considerable challenges  to the adoption of LLMs as research tools in biomedicine. For this reason, even though, as we have shown, LLMs could reasonably handle the task of prioritizing a list of candidate genes when directly prompted, the level of trust in the models and the answers they generate could be insufficient for systematic adoption. Thus, the multi-step process that we implemented, which includes checks and balances and improves transparency of the decision-making process, might offer a viable pathway towards more widespread adoption of LLMs as research tools.

Another inherent limitation of LLMs is that their reliability is closely tied to the quality and comprehensiveness of the training data. While they excel at aggregating and summarizing vast amounts of existing biomedical literature, they may be limited in generating insights beyond what is already documented. In our view, LLMs are unlikely to replace traditional scientific methods; instead, they serve as valuable augmentations. They streamline the laborious process of sifting through extensive literature and data, as exemplified in our knowledge-driven gene prioritization pipeline.

In recognition of the rapidly evolving landscape of Large Language Models (LLMs) and their associated tools, we also acknowledge the limitation of focusing on a select few models in this study. At the time of this research, our choice centered around widely recognized and empirically proven models, specifically OpenAI's GPT-4 and Anthropic's Claude, as these provided a robust baseline for our gene prioritization tasks. While newer models and tools continue to emerge, offering potentially different capabilities or performance metrics, the LLMs chosen for this project were deemed most suitable for this specific application. Future iterations of this work may benefit from evaluating these emerging technologies, but for the scope and timing of the current project, we believe our selection was appropriate and effective.

## Conclusions

In conclusion, our findings underscore LLMs potential in enhancing knowledge-driven candidate gene prioritization and selection processes. Through this study, we demonstrated that LLMs could direct gene selection with minimal human input, bringing about significant advancements in efficiency and productivity. Our ongoing efforts in developing scripts for interfacing with the GPT-4 and Claude APIs should further expedite the prioritization of extensive module repertoires such as BloodGen3 and pave the way for the creation of a universal immune profiling TFA panel. Moreover, basic parameter adjustments in such streamlined workflow could also allow for the design of disease-specific panels with minimal effort. As previously demonstrated with our work on COVID-19, this could include favoring the selection of genes specifically relevant to the pathophysiology of the target disease [14]. Although this development is still in its preliminary stages, our objective is to provide a tool that streamlines the gene prioritization process while maintaining transparency, accuracy, and relevance, especially in the context of disease-specific applications.

### Supplementary Information

**Additional file 1: **Is a spreadsheet including numerical and textual outputs generated by the models and data transformations operated for module M9.2.

**Additional file 2: **Is the prioritization report for module M9.2 authored by Damien Chaussabel. The file is shared in the PDF format.

**Additional file 3: **Is the prioritization report for module M11.2 authored by Damien Chaussabel. The file is shared in the PDF format.

**Additional file 4: **Is the prioritization report for module M11.3 authored by Basirudeen Kabeer. The file is shared in the PDF format.

**Additional file 5: **Is the prioritization report for module M11.4 authored by Éléonore Bettacchioli. The file is shared in the PDF format.

**Additional file 6: **Is the prioritization report for module M12.11 authored by Olivia White and Marina Yurieva. The file is shared in the PDF format.

**Additional file 7: **Is the prioritization report for module M13.26 authored by Marina Yurieva. The file is shared in the PDF format.

**Additional file 8: **Is the prioritization report for module M13.30 authored by Mohammed Toufiq. The file is shared in the PDF format.

**Additional file 9: **Is the prioritization report for module M14.53 authored by Mohammed Toufiq. The file is shared in the PDF format.

**Additional file 10: **Is the prioritization report for module M15.53 authored by Darawan Rinchai. The file is shared in the PDF format.

**Additional file 11: **Is the prioritization report for module M15.74 authored by Darawan Rinchai. The file is shared in the PDF format.

**Additional file 12: **Is the prioritization report for module M15.100 authored by Mohammed Toufiq. The file is shared in the PDF format.

## Data Availability

The transcriptome datasets used as part of this work are available publicly in the NCBI GEO repository under accession IDs GSE24759, GSE6042459 and GSE100150. The R scripts generated as part of this project are available at: https://github.com/Drinchai/A37_LLM.
