# Supplementary material for: Harnessing large language models (LLMs) for candidate gene prioritization and selection
Source: J Transl Med. 2023 Oct 16;21:728. doi: 10.1186/s12967-023-04576-8 (PMC10580627; doi:10.1186/s12967-023-04576-8)
Supplement: Supplementary file 2 — Additional file 2: Is the prioritization report for module M9.2 authored by Damien Chaussabel. The file is shared in the PDF format. [file 12967_2023_4576_MOESM2_ESM.pdf]

M9.2 Prioritization report

Prepared by: Damien Chaussabel (The Jackson Laboratory for Genomic Medicine, Farmington, CT, USA)

1. Composition

The module M9.2 belongs to Aggregate A37 from the BloodGen3 fixed module repertoire (1,2). This module aggregate is associated with circulating erythroid cells. It was found to be associated with RSV disease severity (3). We showed in the same study that the abundance of A27 transcripts is also elevated in the blood of patients with stage IV melanoma (3).

It comprises 30 genes: ALAS2, BCL2L1, BPGM, C14ORF45, C1ORF128, CA1, EPB42, FAM46C, FECH, GMPR, GPR146, GYPB, GYPE, IFIT1L, KRT1, MARCH8, NFIX, OR2W3, OSBP2, PLEK2, SELENBP1, SLC14A1, SLC4A1, SNCA, TMCC2, TMOD1, TNS1, TRIM10, TUBB2A, XK.

2. Functional convergence

Functional associations were identified among the genes constituting M9.2 using GPT-4 (see Step 2 method for details).

Functional Themes	Associated Genes	Comments
Erythrocyte development and function	ALAS2, BPGM, EPB42, GYPB, GYPE, SLC4A1, XK, FECH	These genes are all involved in processes related to erythrocyte (red blood cell) development, function, or associated conditions. For example, ALAS2 and FECH are involved in heme biosynthesis, a crucial component of hemoglobin. BPGM, EPB42, SLC4A1, GYPB, GYPE, and XK are directly involved in erythrocyte structure or function, and mutations in these genes can lead to conditions like hereditary spherocytosis, elliptocytosis, or blood group antigen variations.
Cytoskeleton and cellular structure	TNS1, TMOD1, TUBB2A	These genes are all involved in cytoskeleton formation and function, which is essential for cell shape, division, and movement.
Ubiquitin-proteasome system	MARCH8, TRIM10	These genes are involved in the ubiquitin-proteasome system, which is essential for protein degradation, cell cycle regulation, and other crucial cellular functions.

3. Scoring and prioritization

Genes were scored on six criteria using GPT-4 and Claude. The scores were averaged, and candidate genes ranked according to their cumulative scores (**Figure 1**, Methods: Step 3 and Step 4).

The two LLMs were requested to score each gene on the following six statements:

a. The gene is associated with erythroid cells or erythropoiesis.

b. The gene is currently being used as a biomarker in clinical settings.

c. The gene has potential value as a blood transcriptional biomarker.

d. The gene is relevant to circulating leukocytes immune biology.

e. The gene is a known drug target.

f. The gene is therapeutically relevant for immune-mediated diseases.

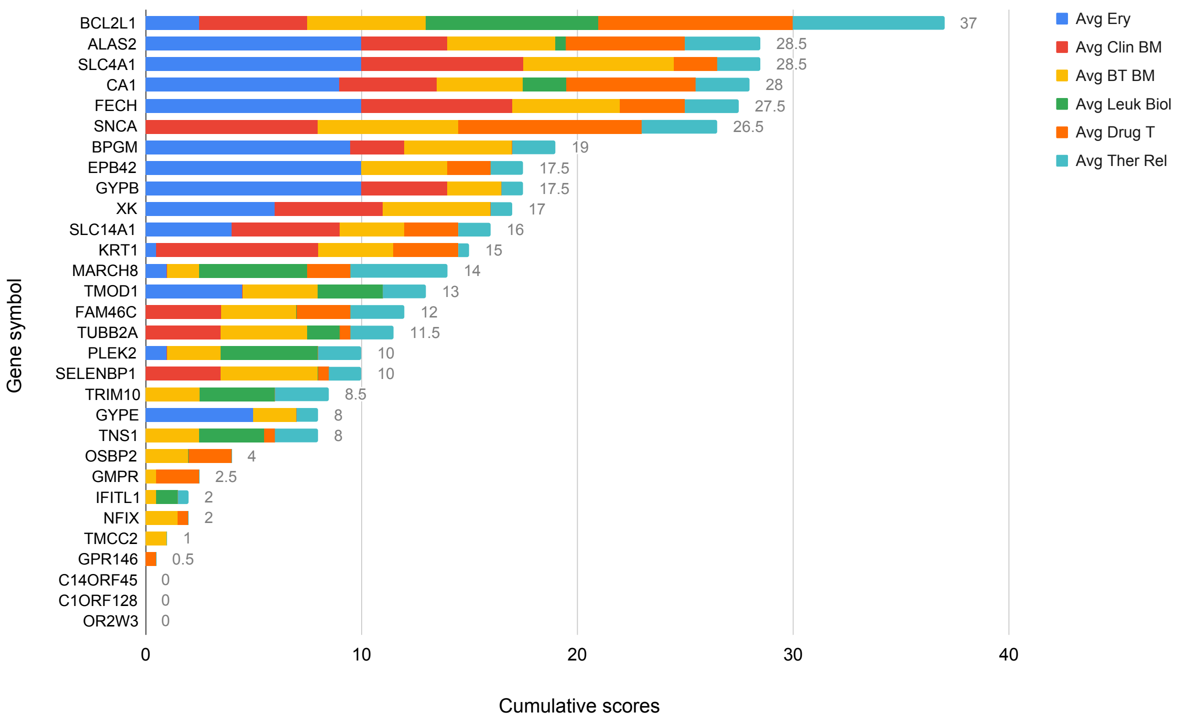

Figure 1: The stacked bar graph shows cumulative scores across six criteria for the 30 M9.2 genes. The top five genes selected for further evaluation were: BCL2L1, ALAS2, SLC4A1, CA1 and FECH.

.

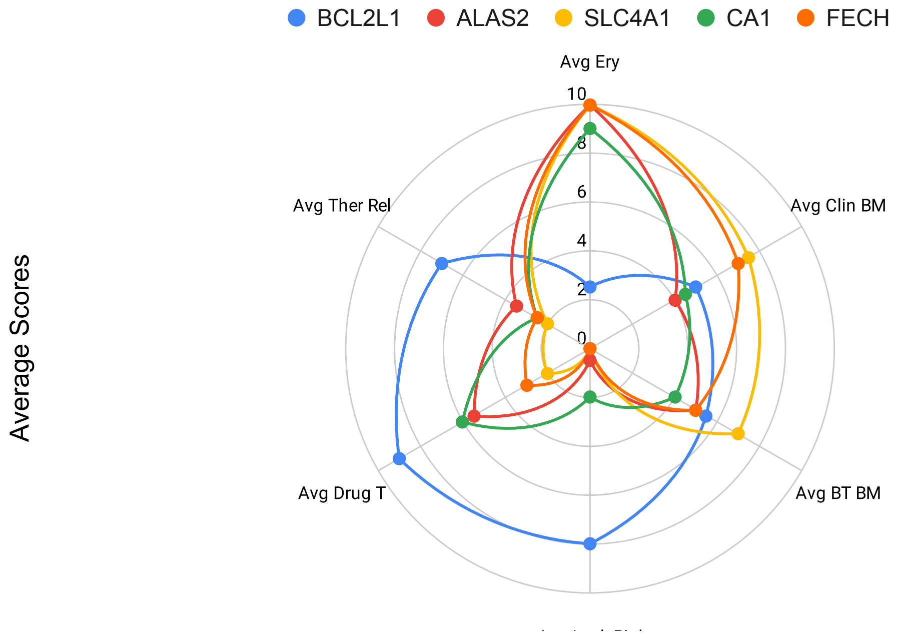

Figure 2: The radar plot represents individual scores for the 5 top scoring genes: BCL2L1, ALAS2, SLC4A1, CA1, and FECH.

4. Knowledge-driven evaluation of top five candidates

Justifications given by the GPT-4 and Claude for the scores provided across the 6 criteria were compiled and summarized by GPT-4 (Methods: Step 5). For each statement pertinent references were retrieved using GPT-4 or Claude, with the relevance of each reference checked and attributed manually by researcher authoring this report.

Function: "The BCL2L1, ALAS2, SLC4A1, CA1, and FECH genes play crucial roles in cellular apoptosis regulation, heme biosynthesis, acid-base balance, and overall erythrocyte physiology. The BCL2L1 gene encodes a critical apoptosis-regulating protein in the Bcl-2 family that exists as Bcl-xL and Bcl-xS isoforms with opposing impacts on cell survival (4,5). The ALAS2 gene instructs the production of the 5'-aminolevulinate synthase 2 enzyme, essential for initiating heme biosynthesis in red blood cells, with mutations linked to X-linked sideroblastic anemia (6). The SLC4A1 gene drives the formation of an anion exchanger that modulates intracellular and extracellular pH by orchestrating the exchange of chloride, bicarbonate, and carbonate anions across the cell membrane, an integral process affected in conditions such as hereditary

spherocytosis (7,8). The CA1 gene directs the production of carbonic anhydrase 1, a cytosolic enzyme that helps maintain tissue and blood pH balance by aiding the conversion of carbon dioxide and water into bicarbonate and protons, primarily in red blood cells (9,10). Lastly, the FECH gene governs the expression of ferrochelatase, the terminal enzyme in the heme biosynthesis pathway, with mutations leading to erythropoietic protoporphyria due to protoporphyrin accumulation (11)."

a. Relevance to erythroid cells and erythropoiesis: "While BCL2L1 plays a general role in controlling cell death, there is no evidence it is specifically associated with erythroid cells or erythropoiesis. In contrast, ALAS2, SLC4A1, CA1, and FECH have critical roles tied to erythroid cell function and erythropoiesis. ALAS2 is highly expressed in erythroid progenitors and is essential for heme biosynthesis, a process vital for red blood cells (12). SLC4A1 is highly expressed in erythroid cells, where it maintains pH balance and facilitates oxygen transport. Mutations in SLC4A1 can lead to hereditary spherocytosis (13). CA1 is strongly associated with erythroid cells due to its high expression in red blood cells and role in maintaining their pH balance (14). FECH is also highly expressed in erythroid cells and is essential for heme biosynthesis during erythropoiesis (15,16). In summary, while BCL2L1 has a general role in apoptosis, ALAS2, SLC4A1, CA1, and FECH are integral to erythroid cell physiology and erythropoiesis."

b. Is used as a clinical biomarker: "The potential utility of BCL2L1, ALAS2, SLC4A1, CA1, and FECH as clinical biomarkers varies considerably. While BCL2L1 is not commonly utilized as a biomarker in clinical settings, it might be of interest in certain malignancies due to its key role in apoptosis regulation, although further research is warranted (17). ALAS2 is not extensively used as a clinical biomarker, except as a biomarker and therapeutic target for X-linked sideroblastic anemia (18). SLC4A1 has clinical relevance as a biomarker for hereditary spherocytosis and as a therapeutic target in sickle cell disease and other red blood cell disorders (19)." CA1, is not broadly used as a biomarker. "Lastly, while FECH is not typically viewed as a 'biomarker' in a conventional sense, its mutations serve as genetic markers for erythropoietic protoporphyria, and its deficiency can be used as a biomarker for this and other porphyrias (20)."

c. Potential relevance as a blood transcriptional biomarker: "BCL2L1, ALAS2, SLC4A1, CA1, and FECH each exhibit potential utility as blood transcriptional biomarkers in specific clinical

contexts. BCL2L1, due to its central role in apoptosis regulation, holds potential as a biomarker for diseases associated with apoptosis dysregulation, including certain cancers (17), albeit further research is necessary. ALAS2, given its fundamental role in heme biosynthesis, could potentially serve as a biomarker for conditions such as X-linked sideroblastic anemia (18), but it is not typically utilized as a blood transcriptional biomarker. SLC4A1, due to its crucial function in red blood cell physiology and its link to hereditary spherocytosis, may serve as a potential blood transcriptional biomarker for this condition (19). CA1, due to its expression in red blood cells, could potentially be used as a biomarker for conditions impacting pH balance in the blood, although more research is warranted (16). Lastly, FECH, due to its critical role in heme biosynthesis, could serve as a potential blood transcriptional biomarker for conditions impacting this process, however, more research is needed for its broader application (21)."

d. Relevance to leukocytes immune biology: "The BCL2L1, ALAS2, SLC4A1, CA1, and FECH genes each have varying degrees of relevance to circulating leukocytes immune biology. BCL2L1, due to its role in apoptosis regulation, is crucial to the lifespan and function of immune cells and is therefore highly relevant to leukocyte immune biology (22,23). Conversely, ALAS2, being erythroid-specific, has limited relevance to circulating leukocyte immune biology. There is no significant evidence pointing towards SLC4A1 having relevance to circulating leukocyte immune biology. CA1, despite being expressed in some circulating immune cells like monocytes, only has limited relevance to immune biology. Lastly, FECH does not display any significant relevance to the immune biology of circulating leukocytes, as per the current evidence."

e. Is a known drug target: "The BCL2L1, ALAS2, SLC4A1, CA1, and FECH genes are each known to varying degrees as drug targets in clinical medicine. BCL2L1 is particularly significant as a target in cancer therapy, where strategies are being developed to inhibit its anti-apoptotic function (17,24). ALAS2 has been targeted for treatment of X-linked sideroblastic anemia, although its broader application as a drug target is currently limited (25–27). While SLC4A1 could potentially be a drug target for conditions such as hereditary spherocytosis, more research is needed. CA1, as part of the carbonic anhydrase group, has been explored for drug therapy potential, especially considering its role in pH regulation, and inhibitors are currently used or studied for conditions like glaucoma, epilepsy, and cancer (28). Lastly, while FECH is not a well-

documented drug target, there is interest in its potential for treating disorders related to heme biosynthesis, including erythropoietic protoporphyria (29)."

f. Potential therapeutic relevance for immune-mediated diseases: "The BCL2L1, ALAS2, SLC4A1, CA1, and FECH genes exhibit varying degrees of therapeutic relevance for immune-mediated diseases. BCL2L1, due to its role in regulating apoptosis in lymphocytes, holds potential therapeutic value for immune-mediated diseases (30). ALAS2, despite its primary role in erythropoiesis, may have limited relevance for immune-mediated hemolytic anemias. As of 2021, the therapeutic relevance of SLC4A1 for immune-mediated diseases remains unclear, although its potential impact on pH or oxygen balance disorders that affect red blood cells may warrant further research (13,31). CA1 is not typically a primary target for immune-mediated diseases, but the role of members of the carbonic anhydrase family in managing acid-base balance or bone resorption in such conditions could be of interest (32). Similarly, while FECH is not a well-documented target for immune-mediated diseases, potential relevance may exist for certain red blood cell disorders or porphyrias, necessitating further investigation (33). In summary, BCL2L1 is the gene with the clearest therapeutic relevance for immune-mediated diseases among this group, due to its direct role in lymphocyte apoptosis. The other genes have potential connections to immune disease management, but require more research to determine their broader therapeutic relevance in this context."

5. **Pick(s) for M9.2 based on summarized justifications.**

We aim to establish a universal immune profiling assay that measures transcript abundance of a panel of genes in whole blood using targeted molecular profiling technology (Immune profiling transcriptome fingerprinting assay). We first attempted to identify top candidates by prompting the GPT-4 and Claude using as input the summarized and fact check justifications obtained from the previous step.

Claude and GPT-4 were prompted to select a top candidate on the basis of the information summarized above in section 4, taking into account all six scoring criteria equally (Methods: Step 6).

Claude chose ALAS2: "Based on the criteria outlined, I would suggest ALAS2 as the top candidate from the genes listed. Here is a summary of my assessment:

a) Relevance to erythroid cells/erythropoiesis: ALAS2 has the most direct relevance, as it encodes an enzyme essential for initiating heme biosynthesis specifically in red blood cells. Mutations are linked to an erythroid condition.
b) Current biomarker use: ALAS2 is used as a biomarker and therapeutic target for a red blood cell disorder, X-linked sideroblastic anemia. This is more significant biomarker use compared to the other genes.
c) Potential blood transcriptional biomarker: ALAS2 has clear potential as a biomarker for conditions affecting heme biosynthesis and erythropoiesis, though not yet utilized broadly.
d) Relevance to leukocyte biology: ALAS2 has limited relevance to leukocytes given its erythroid specificity.
e) Known drug target: ALAS2 is an established drug target for X-linked sideroblastic anemia.
f) Therapeutic relevance for immune diseases: ALAS2 may have relevance for immune-mediated hemolytic anemias, though therapeutic applications are currently limited.

In summary, ALAS2 has the most direct significance for erythroid cells/erythropoiesis, an existing biomarker role, blood biomarker potential, and known drug target status. Though limited in leukocyte relevance and immune disease applications, it appears the strongest candidate based on weighting the criteria equally. Please let me know if you would like me to explain or expand on any part of my assessment.

GPT-4 chose BCL2L1: "Based on the information provided and weighting all six criteria equally, BCL2L1 (B-cell lymphoma 2-like 1) appears to be the top candidate among the genes described (BCL2L1, ALAS2, SLC4A1, CA1, and FECH). Here's how this gene stacks up against the six criteria:

a) Relevance to erythroid cells or erythropoiesis: BCL2L1 is associated with erythroid cells through its control of apoptosis, though it's not specifically linked to erythropoiesis.
b) Current use as a biomarker: Although BCL2L1 is not widely used as a biomarker, it could be of interest in certain malignancies due to its key role in apoptosis regulation.

c) Potential use as a blood transcriptional biomarker: BCL2L1, due to its role in apoptosis regulation, has potential as a biomarker for diseases associated with apoptosis dysregulation, including certain cancers.
d) Relevance to leukocytes immune biology: BCL2L1 is highly relevant to leukocyte immune biology due to its role in regulating the lifespan and function of immune cells.
e) Being a known drug target: BCL2L1 is significant as a target in cancer therapy, with strategies being developed to inhibit its anti-apoptotic function.
f) Therapeutic relevance for immune mediated diseases: BCL2L1 holds potential therapeutic value for immune-mediated diseases due to its role in regulating apoptosis in lymphocytes.

While the other genes listed also meet some of these criteria to varying extents, BCL2L1 appears to be the best fit when considering all criteria equally."

6. Examining expression patterns of top 5 candidates across leukocyte populations.

The LLMs did not incorporate reference transcriptional profiling data in their evaluation and prioritization of candidate genes. The expression profiles of the top 5 candidate genes were retrieved for three reference datasets. These encompassed diverse leukocyte populations and hematopoietic progenitors as well as the whole blood of patients with a wide range of conditions.

6.1 <u>Densely interconnected transcriptional circuits control cell states in human hematopoiesis</u> – <u>(GSE24759) (34)</u>
http://developmentalimmunology.gxbsidra.org/dm3/geneBrowser/show/4000026

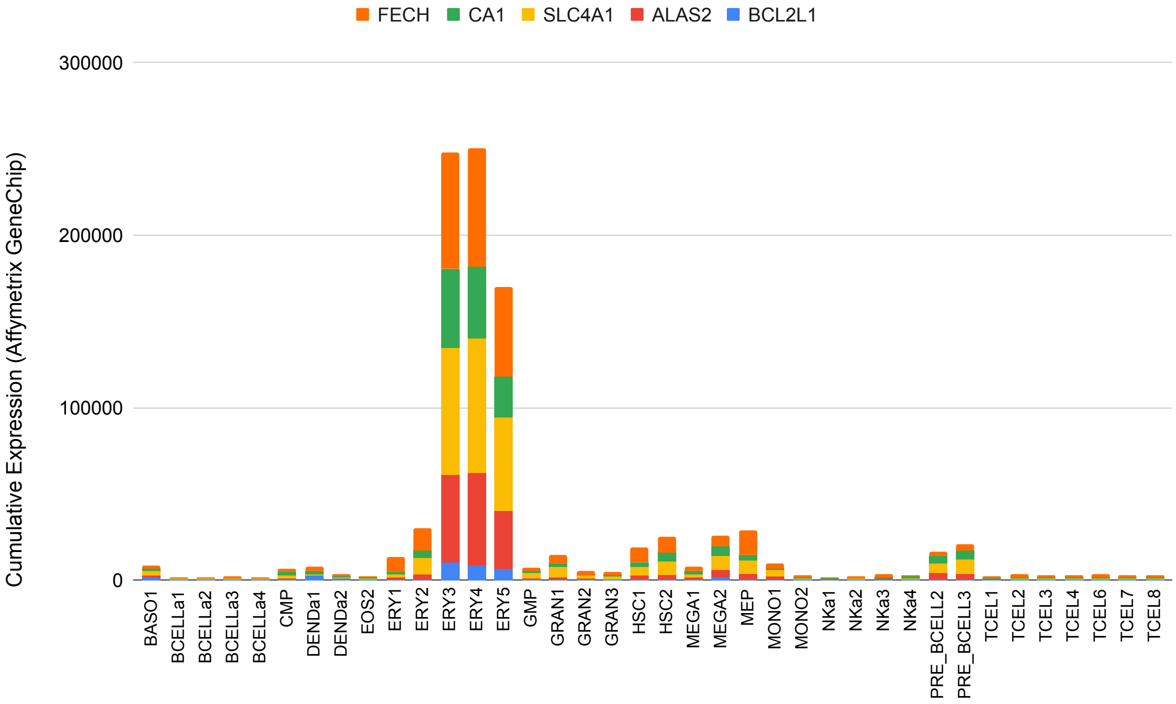

Figure 3. This stacked bar graph shows the expression levels of the five candidate genes. ERY3-5 populations are CD71+ GLYA+ erythroid cells. The original article provides full experimental details: (34). The web link above also provides access to more complete information about this dataset as well as individual gene profiles.

6.2 Next generation sequencing of human immune cell subsets across diseases – (GSE60424)

http://cd2k.gxbsidra.org/dm3/geneBrowser/show/4000098 (35)

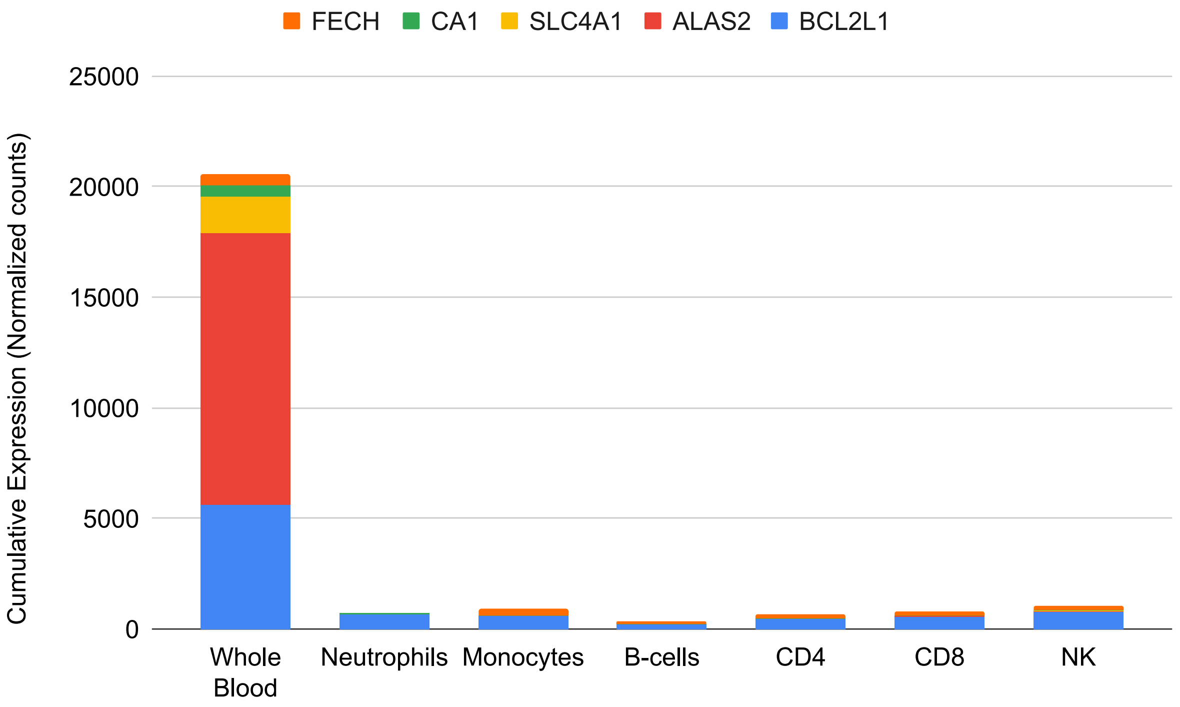

Figure 4: This stacked bar graph shows the expression levels measured by RNAseq of the five candidate genes across six leukocyte populations and whole blood. The abundance is shown for each gene in each population for an average of up to 20 samples. Additional experimental details are accessible via the link provided above and is available as part of this sample series GEO record (GSE60424) (35).

6. 3 BloodGen3 reference cohorts (GSE100150)

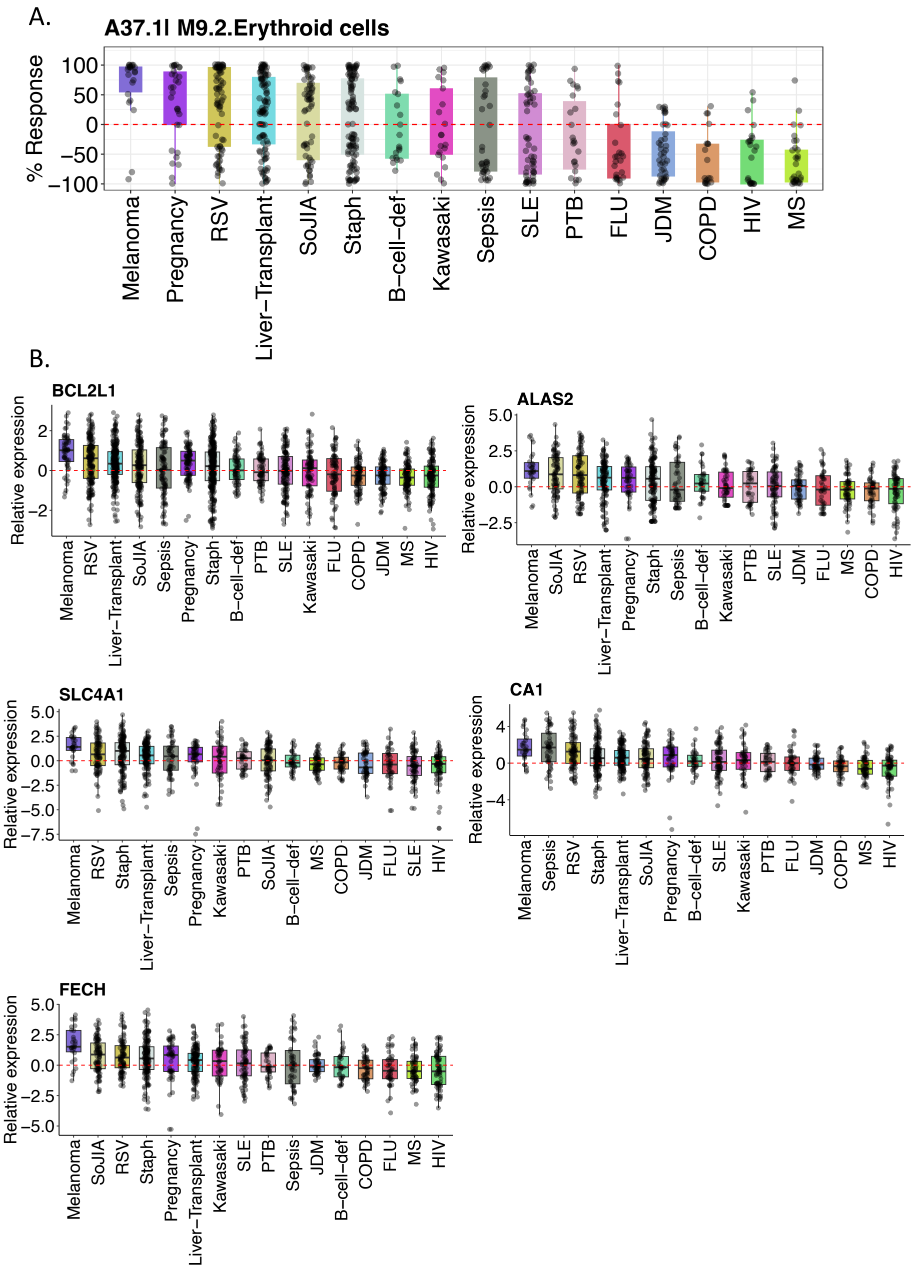

Figure 5: The box plot in panel A below shows the changes in abundance of transcripts comprising the M9.2 module, expressed within a cohort as the percentage of genes showing differential expression between each patient and the respective set of control subjects for this cohort. Differential expression is based on a fixed cut-off (Log2 Fold Change > 1.5, difference >10). These values are expressed as % module activity and can range from +100% (all genes are upregulated) to -100% (all genes are down regulated). The Box plots in panel B show expression levels for the top 5 scoring M9.2 genes in patient cohorts relative to respective control subjects matched for demographics variables.

7. **Overall pick for M9.2 based on summarized justifications and reference transcriptional datasets.**

   Through multi-tier prompting, GPT-4 and Claude were requested to select a top candidate, based on the fact-checked justifications summaries generated earlier, but this time also taking into account the transcriptional profiles from the three reference datasets described above.

   This was achieved using multiple prompts. This strategy was successful when using Claude but we GPT-4 could not follow our directives from start to finish when presented with large amounts of textualized transcriptional data.

   <u>However, both models did converge on ALAS2 as the top candidate for module M9.2. It is a choice that we concurred with, given the rationale provided by both LLMs and our understanding of ALAS2 and BCL2L1 biological and clinical relevance, in conjunction with our reading of the reference transcriptional data which showed comparatively higher levels of expression and restriction of ALAS2 across leukocyte populations.</u>

   The transcription of the final round of prompting is shown below.

**Prompts**	**CLAUDE**	**GPT-4**
PROMPT 1: Based on the summary below, please recommend the top candidate gene to include in a targeted blood transcriptional profiling panel. The intent is for the selected gene to be representative of an erythroid cell signature associated with response to mRNA vaccines, severity of RSV infection and that found to be expressed at high levels in patients with metastatic melanoma and in liver transplant recipients. Please weigh the following criteria equally in your recommendation: 1) Relevance to erythroid cells and erythropoiesis, 2) Current use as a biomarker, 3) Potential use as a blood transcriptional biomarker, 4) Relevance to leukocyte immune biology, 5) Status as a known drug target, 6) Therapeutic relevance for	Based on the provided summary, I would recommend ALAS2 as the top candidate gene to include in the targeted blood transcriptional profiling panel. The key reasons are:<br>• ALAS2 plays a crucial and specific role in heme biosynthesis in erythroid cells, making it highly relevant to erythropoiesis. This aligns closely with the aim to select a gene representative of an erythroid signature.<br>• While not yet an established biomarker, ALAS2 has potential utility as a blood transcriptional biomarker for conditions affecting heme biosynthesis like sideroblastic anemia, given its integral involvement.	Based on the provided summary, the most suitable candidate gene to include in the targeted blood transcriptional profiling panel, considering all the specified criteria, appears to be the BCL2L1 gene.<br>1. Relevance to erythroid cells and erythropoiesis: While BCL2L1 does not have direct involvement in erythropoiesis, it plays a crucial role in cellular apoptosis regulation, which affects erythroid cells.<br>2. Current use as a biomarker: Although not widely used, BCL2L1's role in apoptosis regulation can make it an interesting candidate for certain malignancies.<br>3. Potential use as a blood transcriptional biomarker: BCL2L1

immune mediated diseases: *[input summarized and fact-checked justifications from Step 5]*	• ALAS2 is already an established therapeutic target for a condition affecting erythropoiesis and red blood cells - X-linked sideroblastic anemia. This demonstrates its translational relevance.<br>• In contrast, the other genes have limitations regarding erythroid specificity (BCL2L1), lack of broad biomarker potential (CA1, FECH), unclear relevance as a drug target (SLC4A1), or less direct involvement in erythropoiesis (FECH).<br><br>In summary, ALAS2's crucial and specific role in heme biosynthesis in erythroid cells, translational relevance as a therapeutic target, and potential as a blood transcriptional biomarker make it the top choice to represent an erythroid signature for the intended profiling panel.	holds potential as a biomarker for diseases associated with apoptosis dysregulation, like certain cancers.<br>4. Relevance to leukocyte immune biology: BCL2L1's function in apoptosis regulation is crucial to the lifespan and function of immune cells, making it highly relevant to leukocyte immune biology.<br>5. Status as a known drug target: BCL2L1 is a significant target in cancer therapy, with strategies being developed to inhibit its anti-apoptotic function.<br>6. Therapeutic relevance for immune-mediated diseases: BCL2L1, by regulating apoptosis in lymphocytes, holds potential therapeutic value for immune-mediated diseases.<br>While the other genes also have their unique advantages and applications, the BCL2L1 gene is the most versatile across the provided criteria. It is important to note, however, that the utility and effectiveness of BCL2L1 as a representative of an erythroid cell signature will depend on the specific clinical and research contexts, and further research might be necessary to fully confirm its potential in this regard.
PROMPT 2: Now that you have provided an initial recommendation based on the summary, take into account the following RNA-sequencing expression data from key immune cell types and whole blood samples: *[input textualized transcriptional data].* Given this additional RNA-seq expression data, does your recommended gene selection remain the same? Please explain which gene you would recommend at this stage and why, incorporating both the summarized information *provided earlier and RNA-seq data in your rationale."*	After reviewing the additional RNA-seq expression data, I would maintain my original recommendation of ALAS2 as the top candidate gene to include in the targeted blood transcriptional profiling panel.<br>The key reasons are:<br>• The RNA-seq data confirms the erythroid-specific expression of ALAS2, with counts of 616 in whole blood but only 1 in all the leukocyte subsets. This aligns perfectly with the intent to select a gene representative of an erythroid signature.<br>• In contrast, the other genes like BCL2L1, SLC4A1, CA1	With the addition of the RNA-seq data, it's clear that ALAS2 is the most abundantly expressed gene in whole blood with a count of 616. This contrasts with BCL2L1, the initially recommended gene, which exhibits a lower expression level in whole blood (280). Importantly, ALAS2 demonstrates minimal expression in all other tested immune cells (counts of 1), suggesting a strong erythroid cell-specific expression, fitting the criteria to be representative of an erythroid cell signature. BCL2L1, on the other hand, is more uniformly expressed across various cell types,

	and FECH exhibit low but detectable expression across leukocyte subsets, indicating they are more broadly expressed across blood cell types.<br>• The erythroid-restricted expression of ALAS2 makes it a better biomarker of erythropoiesis compared to the other genes.<br>• ALAS2 remains a relevant therapeutic target for disorders of erythropoiesis like sideroblastic anemia, despite the additional RNA-seq data.<br>• The RNA-seq data does not provide any evidence to contradict ALAS2's integral role in heme biosynthesis in erythroid cells specifically.<br><br>In summary, the RNA-seq data confirming erythroid-restricted expression of ALAS2 further strengthens the rationale for selecting it as the top candidate gene representative of an erythroid signature for the intended blood transcriptional profiling panel, in line with my original recommendation. The other genes appear more broadly expressed across leukocytes based on the RNA-seq data.<br>Copy	which makes it less specific to erythroid cells.<br>Given these findings, I recommend reconsidering the selection and placing ALAS2 as the top candidate gene for inclusion in the targeted blood transcriptional profiling panel. Here's why:<br>1. Relevance to erythroid cells and erythropoiesis: ALAS2 plays an essential role in heme biosynthesis in red blood cells, a process critical for erythropoiesis.<br>2. Current use as a biomarker: ALAS2 is used as a biomarker and therapeutic target for X-linked sideroblastic anemia.<br>3. Potential use as a blood transcriptional biomarker: Although not typically utilized as a blood transcriptional biomarker, its high expression in whole blood and critical role in erythropoiesis highlight its potential.<br>4. Relevance to leukocyte immune biology: Although it has limited relevance to circulating leukocyte immune biology due to its erythroid-specific nature, its low expression in other immune cells supports its specificity for erythroid cells.<br>5. Status as a known drug target: ALAS2 has been targeted for the treatment of X-linked sideroblastic anemia.<br>6. Therapeutic relevance for immune-mediated diseases: Although it has limited relevance for immune-mediated diseases, it can be relevant for immune-mediated hemolytic anemias.<br>7. By combining both the RNA-seq data and the summary provided earlier, it appears that ALAS2 would be a more appropriate candidate due to its high expression in whole blood and its critical role in erythropoiesis, despite its limited involvement in leukocyte immune biology and use in immune-mediated diseases.

PROMPT 3: Now that you have provided an initial recommendation based on the summary and the RNAseq data, take into account the following microarray expression data from key immune cell types and hematopoietic precursors: *[input textualized transcriptional data].* Given this additional microarray expression data, does your recommended gene selection remain the same? Please explain which gene you would recommend at this stage and why, incorporating the summarized information and RNA-seq data provided earlier, as well as this new microarray data in your rationale.	After reviewing the extensive microarray expression data, I would still recommend ALAS2 as the top candidate gene for inclusion in the targeted blood transcriptional profiling panel.<br>The key reasons are:<br>• The microarray data confirms very high, erythroid-restricted expression of ALAS2 in the GlyA+ erythroblast subsets, with values of 8371, 7664, and 5566 signal intensity units. This agrees with the RNA-seq data showing erythroid specificity.<br>• ALAS2 expression is low (values <100) in all immune cell types profiled by microarrays, including T cells, B cells, NK cells, dendritic cells. This further confirms leukocyte-restricted expression.<br>• Other genes like BCL2L1, SLC4A1, CA1 and FECH are more broadly expressed across immune cells and progenitors, based on the microarray data.<br>• No additional insight from the microarrays contradicts ALAS2's integral involvement in heme biosynthesis in erythroid lineage cells specifically.<br>• ALAS2 remains a relevant therapeutic target in erythroid disorders based on its key role, despite the new microarray data.<br><br>In summary, the extensive microarray expression profiling continues to support ALAS2 as the top gene choice representative of an erythroid signature, based on its high erythroid-restricted expression and lack of expression in other immune cells. The other genes appear more broadly expressed across leukocytes. Therefore, I maintain ALAS2 as my recommended top candidate gene for inclusion in the intended targeted blood transcriptional profiling panel.<br>Copy	*[N/A: input exceed GPT-4 limits. Attempts to break down inputting the textualized data were unsuccessful as GPT-4 lost the thread of the conversation. Thus, we chose to skip to the next prompt]*

PROMPT 4: Now that you have provided an initial recommendation based on the summary and the immune cells RNAseq and microarray data, take into account the following averaged log2 fold changes in RNA abundance in patient cohorts compared to controls. *[input textualized transcriptional data]*. Given this additional data, does your recommended gene selection remain the same? Please explain which gene you would recommend at this stage and why, incorporating the summarized information, the leukocytes RNA-seq and microarray data provided earlier, as well as these new patient cohort profiles in your rationale.	After reviewing the log2 fold change data across multiple patient cohorts, I would maintain my recommendation of ALAS2 as the top candidate gene for the intended blood transcriptional profiling panel.<br>The key reasons are:<br>• ALAS2 shows some of the largest fold changes across conditions like RSV infection, metastatic melanoma, and juvenile arthritis that were called out in the original summary as being relevant.<br>• The magnitude of ALAS2 fold changes are greater than those seen for other genes like BCL2L1, SLC4A1, CA1 and FECH in many of the disease states.<br>• ALAS2's erythroid-specific expression makes its fold changes more amenable to interpretation compared to broadly expressed genes.<br>• No major contradictory evidence arises from the fold change data to refute ALAS2's important role in erythropoiesis.<br>• The leukocyte expression data and key role in heme synthesis remain valid points strengthening ALAS2's selection despite the new cohort profiles.<br>In summary, the gene expression fold changes across multiple patient cohorts and conditions continues to support ALAS2 as a top gene choice representative of an erythroid signature that could provide insights into disease biology based on its marked regulation. The other genes do not show as large or as consistent differences across relevant conditions based on the log2 fold change data. Therefore, I maintain ALAS2 as my recommended top candidate gene for the intended blood profiling panel.	*[GPT-4 was able to interpret the input transcriptional data but lost the thread of the conversation].*<br><br>In the analysis, we observed differences in RNA abundance in whole blood for BCL2L1, ALAS2, SLC4A1, CA1, and FECH across various patient groups compared to controls. Let's summarize some of the notable findings:<br>1. BCL2L1: BCL2L1 expression seems to increase in cases of B-cell deficiency, metastatic melanoma, sepsis, pregnancy, active pulmonary tuberculosis, acute respiratory syncytial virus infection, systemic onset juvenile idiopathic arthritis, staphylococcus aureus infection, and liver transplant recipients. The most pronounced increase was observed in patients with metastatic melanoma. On the other hand, its expression decreases in patients with diseases like chronic obstructive pulmonary disease (COPD), acute influenza, HIV, juvenile dermatomyositis, Kawasaki disease, and multiple sclerosis. The most substantial decrease was observed in patients with HIV infection.<br>2. ALAS2: ALAS2 expression increased in patients with B-cell deficiency, Kawasaki disease, metastatic melanoma, sepsis, pregnancy, active pulmonary tuberculosis, acute respiratory syncytial virus infection, systemic onset juvenile idiopathic arthritis, staphylococcus aureus infection, and liver transplant recipients. The most pronounced increase was seen in patients with metastatic melanoma. However, its expression decreased in patients with COPD, acute influenza, HIV, and juvenile dermatomyositis. The greatest decrease was observed in HIV infection.<br>3. SLC4A1: SLC4A1 expression increased in patients with Kawasaki disease, metastatic

		melanoma, sepsis, pregnancy, active pulmonary tuberculosis, acute respiratory syncytial virus infection, staphylococcus aureus infection, and liver transplant recipients, with metastatic melanoma showing the highest increase. On the other hand, it decreased in patients with B-cell deficiency, COPD, acute influenza, HIV, juvenile dermatomyositis, and multiple sclerosis. The greatest decrease was observed in patients with HIV infection.<br>4. CA1: CA1 expression increased dramatically in most conditions, especially metastatic melanoma, sepsis, pregnancy, active pulmonary tuberculosis, acute respiratory syncytial virus infection, systemic lupus erythematosus, systemic onset juvenile idiopathic arthritis, staphylococcus aureus infection, and liver transplant recipients. The greatest increase was observed in sepsis patients. However, it decreased in patients with B-cell deficiency, COPD, acute influenza, HIV, and multiple sclerosis. The most significant decrease was seen in HIV patients.<br>5. FECH: FECH expression increased slightly in patients with B-cell deficiency and decreased slightly in patients with COPD, but the changes were less pronounced compared to other genes. The expression decreased in conditions like acute influenza, HIV, and juvenile dermatomyositis, with the most significant decrease observed in patients with HIV.<br><br>In all cases, these changes in gene expression may represent physiological responses to disease or might contribute to the pathophysiology of the disease itself. These findings could be valuable for designing further research, or potentially, diagnostic or therapeutic strategies.

		Please note that the interpretation of gene expression data is complex, with many factors influencing the results, such as patient characteristics, sample handling, and microarray technique variability. Hence, these interpretations should be considered as hypotheses that need further validation.
PROMPT 5: Could you please summarize the key conclusions you have drawn from the conversation so far?	Based on reviewing the initial summary, RNA-seq data, microarray data, and patient cohort fold change data provided sequentially, my conclusions are:<br><br>• I would recommend ALAS2 as the top candidate gene for inclusion in the intended targeted blood transcriptional profiling panel based on its:<br>1. Crucial, erythroid-specific role in heme biosynthesis, making it a good representation of an erythroid signature<br>2. Lack of expression in leukocytes and high, restricted expression in erythroblast subsets, confirmed by both RNA-seq and microarrays<br>3. Large, consistent fold changes across conditions like RSV infection, juvenile arthritis, and melanoma that were highlighted as being relevant<br>4. Established utility as a therapeutic target in erythroid disorders like sideroblastic anemia<br>5. Potential as a biomarker for disrupted erythropoiesis based on its tight regulation<br><br>• The other genes (BCL2L1, SLC4A1, CA1, FECH) are more broadly expressed across immune cells and their fold changes are smaller in magnitude or less consistent across key disease states.<br><br>• Each additional dataset provided further evidence supporting ALAS2 as the top choice over other	*[N/A: GPT-4 could not keep the thread of the conversation following the input of large amount of textualized transcriptional profiling data]*

	candidates based on erythroid specificity and associations with conditions of interest.<br>In summary, the collective evidence strongly supports ALAS2 as the top gene for inclusion in the intended blood profiling panel to represent an erythroid signature. The key factors are its erythroid-restricted expression and marked regulation across relevant conditions.	

8. References

1. Altman MC, Rinchai D, Baldwin N, Toufiq M, Whalen E, Garand M, et al. Development of a fixed module repertoire for the analysis and interpretation of blood transcriptome data. Nat Commun. 2021 Jul 19;12(1):4385.

2. Rinchai D, Roelands J, Toufiq M, Hendrickx W, Altman MC, Bedognetti D, et al. BloodGen3Module: Blood transcriptional module repertoire analysis and visualization using R. Bioinforma Oxf Engl. 2021 Feb 24;btab121.

3. Rinchai D, Altman MC, Konza O, Hässler S, Martina F, Toufiq M, et al. Definition of erythroid cell-positive blood transcriptome phenotypes associated with severe respiratory syncytial virus infection. Clin Transl Med. 2020 Dec;10(8):e244.

4. Boise LH, González-García M, Postema CE, Ding L, Lindsten T, Turka LA, et al. bcl-x, a bcl-2-related gene that functions as a dominant regulator of apoptotic cell death. Cell. 1993 Aug 27;74(4):597–608.

5. Hsu SY, Kaipia A, McGee E, Lomeli M, Hsueh AJ. Bok is a pro-apoptotic Bcl-2 protein with restricted expression in reproductive tissues and heterodimerizes with selective anti-apoptotic Bcl-2 family members. Proc Natl Acad Sci U S A. 1997 Nov 11;94(23):12401–6.

6. Bottomley SS, May BK, Cox TC, Cotter PD, Bishop DF. Molecular defects of erythroid 5-aminolevulinate synthase in X-linked sideroblastic anemia. J Bioenerg Biomembr. 1995 Apr;27(2):161–8.

7. Al-Samir S, Papadopoulos S, Scheibe RJ, Meißner JD, Cartron JP, Sly WS, et al. Activity and distribution of intracellular carbonic anhydrase II and their effects on the transport activity of anion exchanger AE1/SLC4A1. J Physiol. 2013 Oct 15;591(20):4963–82.

8. Perrotta S, Gallagher PG, Mohandas N. Hereditary spherocytosis. Lancet Lond Engl. 2008 Oct 18;372(9647):1411–26.

9. Tufts BL, Esbaugh A, Lund SG. Comparative physiology and molecular evolution of carbonic anhydrase in the erythrocytes of early vertebrates. Comp Biochem Physiol A Mol Integr Physiol. 2003 Oct;136(2):259–69.

10. Widdas WF, Baker GF, Baker P. The acceleration of pH volume changes in human red cells by bicarbonate and the role of carbonic anhydrase. Cytobios. 1994;80(320):7–24.

11. Inafuku K, Takamiyagi A, Oshiro M, Kinjo T, Nakashima Y, Nonaka S. Alteration of mRNA levels of delta-aminolevulinic acid synthase, ferrochelatase and heme oxygenase-1 in griseofulvin induced protoporphyria mice. J Dermatol Sci. 1999 Apr;19(3):189–98.

12. Bishop DF, Henderson AS, Astrin KH. Human delta-aminolevulinate synthase: assignment of the housekeeping gene to 3p21 and the erythroid-specific gene to the X chromosome. Genomics. 1990 Jun;7(2):207–14.

13. Alper SL. Genetic diseases of acid-base transporters. Annu Rev Physiol. 2002;64:899–923.

14. Geers C, Gros G. Carbon dioxide transport and carbonic anhydrase in blood and muscle. Physiol Rev. 2000 Apr;80(2):681–715.

15. Wu CK, Dailey HA, Rose JP, Burden A, Sellers VM, Wang BC. The 2.0 A structure of human ferrochelatase, the terminal enzyme of heme biosynthesis. Nat Struct Biol. 2001 Feb;8(2):156–60.

16. Vince JW, Reithmeier RA. Carbonic anhydrase II binds to the carboxyl terminus of human band 3, the erythrocyte C1-/HCO3- exchanger. J Biol Chem. 1998 Oct 23;273(43):28430–7.

17. Delbridge ARD, Grabow S, Strasser A, Vaux DL. Thirty years of BCL-2: translating cell death discoveries into novel cancer therapies. Nat Rev Cancer. 2016 Feb;16(2):99–109.

18. Harigae H, Furuyama K. Hereditary sideroblastic anemia: pathophysiology and gene mutations. Int J Hematol. 2010 Oct;92(3):425–31.

19. Bruce LJ, Robinson HC, Guizouarn H, Borgese F, Harrison P, King MJ, et al. Monovalent cation leaks in human red cells caused by single amino-acid substitutions in the transport domain of the band 3 chloride-bicarbonate exchanger, AE1. Nat Genet. 2005 Nov;37(11):1258–63.

20. Balwani M, Doheny D, Bishop DF, Nazarenko I, Yasuda M, Dailey HA, et al. Loss-of-function ferrochelatase and gain-of-function erythroid-specific 5-aminolevulinate synthase mutations causing erythropoietic protoporphyria and x-linked protoporphyria in North American patients reveal novel mutations and a high prevalence of X-linked protoporphyria.

Mol Med Camb Mass. 2013 Apr 30;19(1):26-35 [Claude; "Reviews how FECH mutations underlie erythropoietic protoporphyria, supporting use as a genetic marker."].

21. Dailey HA, Meissner PN. Erythroid heme biosynthesis and its disorders. Cold Spring Harb Perspect Med. 2013 Apr 1;3(4):a011676.

22. Akgul C, Moulding DA, Edwards SW. Molecular control of neutrophil apoptosis. FEBS Lett. 2001 Jan 5;487(3):318–22.

23. Opferman JT, Korsmeyer SJ. Apoptosis in the development and maintenance of the immune system. Nat Immunol. 2003 May;4(5):410–5.

24. Vogler M, Dinsdale D, Dyer MJS, Cohen GM. Bcl-2 inhibitors: small molecules with a big impact on cancer therapy. Cell Death Differ. 2009 Mar;16(3):360–7.

25. Mirmiran A, Schmitt C, Lefebvre T, Manceau H, Daher R, Oustric V, et al. Erythroid-Progenitor-Targeted Gene Therapy Using Bifunctional TFR1 Ligand-Peptides in Human Erythropoietic Protoporphyria. Am J Hum Genet. 2019 Feb 7;104(2):341–7.

26. Bottomley SS, Fleming MD. Sideroblastic anemia: diagnosis and management. Hematol Oncol Clin North Am. 2014 Aug;28(4):653–70, v.

27. Bergmann AK, Campagna DR, McLoughlin EM, Agarwal S, Fleming MD, Bottomley SS, et al. Systematic molecular genetic analysis of congenital sideroblastic anemia: evidence for genetic heterogeneity and identification of novel mutations. Pediatr Blood Cancer. 2010 Feb;54(2):273–8.

28. Supuran CT. Carbonic anhydrases: novel therapeutic applications for inhibitors and activators. Nat Rev Drug Discov. 2008 Feb;7(2):168–81.

29. Halloy F, Iyer PS, Ćwiek P, Ghidini A, Barman-Aksözen J, Wildner-Verhey van Wijk N, et al. Delivery of oligonucleotides to bone marrow to modulate ferrochelatase splicing in a mouse model of erythropoietic protoporphyria. Nucleic Acids Res. 2020 May 21;48(9):4658–71.

30. Ottina E, Tischner D, Herold MJ, Villunger A. A1/Bfl-1 in leukocyte development and cell death. Exp Cell Res. 2012 Jul 1;318(11):1291–303.

31. Kager L, Bruce LJ, Zeitlhofer P, Flatt JF, Maia TM, Ribeiro ML, et al. Band 3 nullVIENNA , a novel homozygous SLC4A1 p.Ser477X variant causing severe hemolytic anemia, dyserythropoiesis and complete distal renal tubular acidosis. Pediatr Blood Cancer. 2017 Mar;64(3).

32. Lehenkari P, Hentunen TA, Laitala-Leinonen T, Tuukkanen J, Väänänen HK. Carbonic anhydrase II plays a major role in osteoclast differentiation and bone resorption by effecting the steady state intracellular pH and Ca2+. Exp Cell Res. 1998 Jul 10;242(1):128–37.

33. Kieke MC, Klemm J, Tondin AR, Alencar V, Johnson N, Driver AM, et al. Characterization of a novel pathogenic variant in the FECH gene associated with erythropoietic protoporphyria. Mol Genet Metab Rep. 2019 Sep;20:100481.

34. Novershtern N, Subramanian A, Lawton LN, Mak RH, Haining WN, McConkey ME, et al. Densely interconnected transcriptional circuits control cell states in human hematopoiesis. Cell. 2011 Jan 21;144(2):296–309.

35. Linsley PS, Speake C, Whalen E, Chaussabel D. Copy number loss of the interferon gene cluster in melanomas is linked to reduced T cell infiltrate and poor patient prognosis. PloS One. 2014;9(10):e109760.
