# Supplementary material for: Harnessing large language models (LLMs) for candidate gene prioritization and selection
Source: J Transl Med. 2023 Oct 16;21:728. doi: 10.1186/s12967-023-04576-8 (PMC10580627; doi:10.1186/s12967-023-04576-8)
Supplement: Supplementary file 3 — Additional file 3: Is the prioritization report for module M11.2 authored by Damien Chaussabel. The file is shared in the PDF format. [file 12967_2023_4576_MOESM3_ESM.pdf]

M11.2 Prioritization report

Prepared by: Damien Chaussabel (The Jackson Laboratory for Genomic Medicine, Farmington, CT, USA)

1. Composition

The module M11.2 belongs to Aggregate A37 from the BloodGen3 fixed module repertoire (1). This module aggregate is associated with circulating erythroid cells. It was found to be associated with RSV disease severity (2). We showed in the same study that the abundance of A37 transcripts is also elevated in the blood of patients with metastatic melanoma (2).

It comprises 39 genes: ABCC4, AMFR, BAT3, BMP2K, C17ORF39, C9ORF40, DNAJB2, EIF1B, GDE1, GNA12, HS.211743, HS.57079, IQWD1, JAZF1, MED25, NSUN3, ODC1, POLR1D, PPP2R5B, PSME4, RANBP10, RNF14, RSRC1, SELK, SLC2A1, SNX3, TBC1D22B, TMEM183A, TMEM183B, UBE2F, UBQLN1, WBP2, WDR26, WDR45, WDR51B, WNK1, ZBTB44, ZER1, ZNF653.

2. Functional convergence

Functional Themes	Associated Genes	Comments
DNA repair and cell cycle regulation	ABCC4, MED25, and POLR1D	The proteins encoded by ABCC4, MED25, and POLR1D genes play key roles in DNA repair and cell cycle regulation. For example, ABCC4 is involved in the export of many metabolites and drugs out of cells, potentially linked to drug resistance in cancer. POLR1D is part of the RNA polymerase complex involved in transcription and DNA repair.
Protein Degradation	AMFR, UBE2F, UBQLN1, RNF14, and ZER1	AMFR, UBE2F, UBQLN1, RNF14, and ZER1 are associated with the protein ubiquitination and degradation pathway, which is a critical process for maintaining cellular protein homeostasis.
Protein Trafficking	SNX3, WDR45, and TBC1D22B	The genes like SNX3, WDR45, and TBC1D22B are involved in protein trafficking within cells, particularly in endosome-to-Golgi retrograde transport.
Regulation of Translation and Protein Folding	EIF1B and DNAJB2	EIF1B and DNAJB2 are involved in protein translation and folding, respectively. Both these processes are critical for the proper functioning of proteins within cells.

3. Scoring and prioritization

Genes were scored on six criteria using GPT4 and Claude. The scores were averaged, and candidate genes ranked according to their cumulative scores.

The two LLMs were requested to score each gene on the following six statements:
a. The gene is associated with erythroid cells or erythropoiesis.

b. The gene is currently being used as a biomarker in clinical settings.
c. The gene has potential value as a blood transcriptional biomarker.
d. The gene is relevant to circulating leukocytes immune biology.
e. The gene is a known drug target.
f. The gene is therapeutically relevant for immune-mediated diseases.

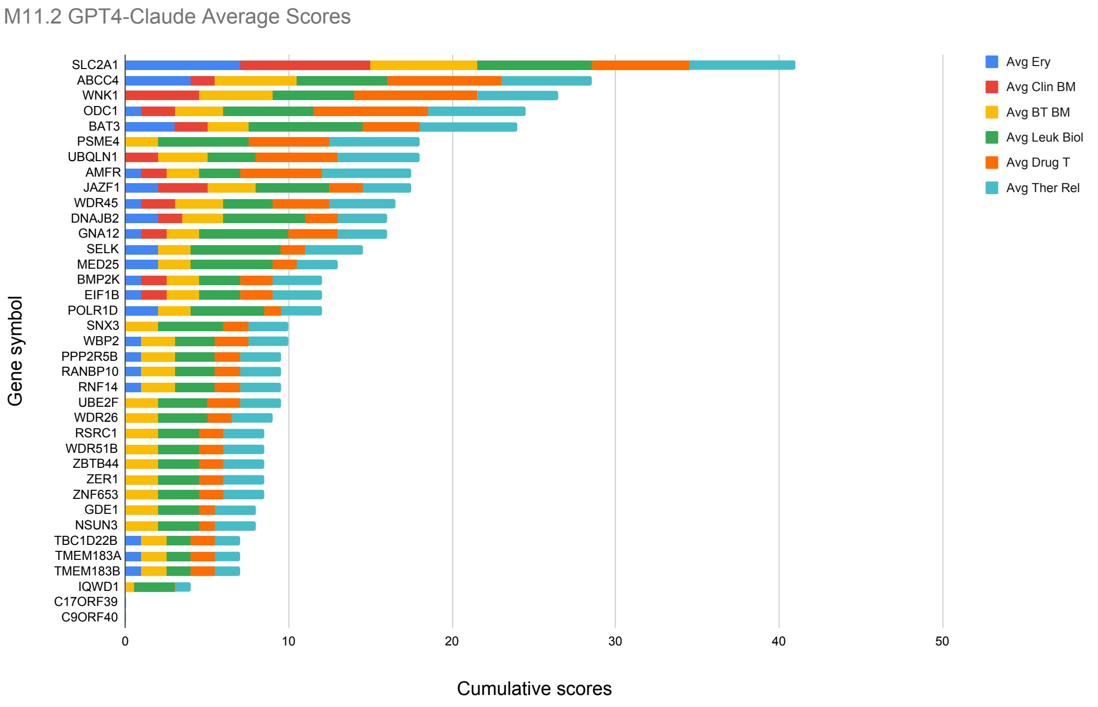

Figure 1: The stacked bar graph shows cumulative scores across six criteria for the 30 M9.2 genes. The top five genes selected for further evaluation were: SLC2A1, ABCC4, WNK1, ODC1 and BAT3.

The top five genes selected for further evaluation were: SLC2A1, ABCC4, WNK1, ODC1 and BAT3.

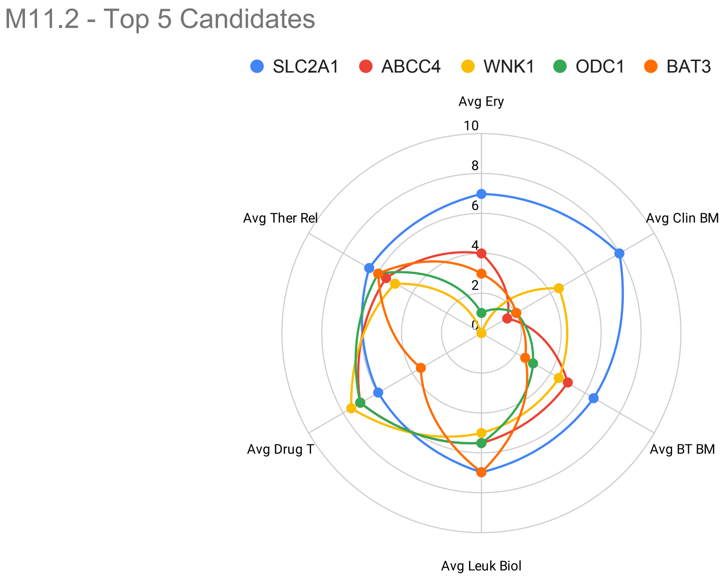

Figure 2: The radar plot represents individual scores for the 5 top scoring genes: SLC2A1, ABCC4, WNK1, ODC1 and BAT3.

4. Knowledge-driven evaluation of top five candidates

Justifications given by the two models for the scores provided across the 6 criteria were compiled and summarized by GPT4 below. For each statement pertinent references were retrieved, either using GPT4, with subsequently checking that the reference provided exists and is pertinent, or via a custom PubMed query.

"<u>Function</u>: The SLC2A1 gene encodes the GLUT1 glucose transporter protein, a crucial player in the transportation of glucose across mammalian cell membranes, including those in the blood-brain barrier. However, mutations in this gene can lead to GLUT1 deficiency syndrome, causing various neurological symptoms (3). ABCC4, another gene, encodes an ATP-binding cassette transporter protein. This protein acts as a cellular efflux pump, particularly for cyclic nucleotides and certain drugs, thus influencing their absorption and disposition (4). It is also implicated in urate homeostasis and prostaglandin metabolism (5,6). The WNK1 gene encodes a serine-

threonine kinase that regulates ion transport and blood pressure. WNK1 mutations can result in pseudohypoaldosteronism type II, a condition characterized by hypertension and impaired excretion of K+ and H+ (7,8). ODC1 encodes the enzyme ornithine decarboxylase, instrumental in polyamine biosynthesis and thereby cell proliferation and growth. Overexpression of this gene has been linked to various types of cancers (9,10). Finally, BAT3, or BAG6, encodes a protein involved in apoptosis, protein homeostasis, cellular stress responses, and immune regulation, functioning as a chaperone for the degradation and quality control of proteins (11,12).

Association with erythroid cells or erythropoiesis: The SLC2A1 gene, which encodes the GLUT1 glucose transporter, plays a crucial role in erythroid cells, primarily erythrocytes. It facilitates the essential glucose transport process, which is pivotal for cellular metabolism, including that of red blood cells (13). ABCC4, while expressed in erythroid precursor cells, does not appear to have a direct regulatory effect on erythropoiesis, according to current evidence (14). For the WNK1, BAT3 and ODC1 genes, no direct evidence indicates a role in erythroid cells or erythropoiesis.

Used as a biomarker in clinical settings: The SLC2A1 gene, encoding the GLUT1 glucose transporter, is recognized as a clinical biomarker for GLUT1 deficiency syndrome, with testing aiding in its diagnosis (15,16). Conversely, ABCC4 has potential links to drug response and toxicity due to its role in the transport of various substrates including certain drugs. However, it is not typically used as a routine clinical biomarker (17). Similarly, the WNK1 gene, while used in diagnosing certain familial hypertension forms, is not generally recognized as a clinical biomarker (7). The ODC1 gene, although not widely utilized as a biomarker, is under investigation for its potential correlations with specific cancer outcomes (9). Lastly, despite some research suggesting possible uses of BAT3 in the context of cancer, it is not widely employed as a clinical biomarker in standard practice (18)

Potential value as a blood transcriptional marker: SLC2A1, ABCC4, WNK1, ODC1, and BAT3 are currently under consideration for their potential utility as blood transcriptional biomarkers, with each gene showing potential relevance due to its specific biological role. SLC2A1, the gene encoding the GLUT1 glucose transporter, could serve as a significant marker in conditions such as diabetes or certain cancers where glucose metabolism is crucially altered. Nevertheless, rigorous validation is still necessary to affirm its clinical utility. Similarly, ABCC4, involved in drug disposition and prostaglandin metabolism, may offer pharmacogenomic or disease monitoring applications (4), yet its potential requires additional research for substantiation. WNK1, known for its regulation of electrolyte balance and blood pressure (19), represents a theoretical biomarker for conditions like hypertension, but further research is necessary to evaluate its real-world performance and utility. ODC1, associated with cell growth and various cancers (20), could be indicative of neoplastic processes. However, the current body of evidence is insufficient to support its broad use as a blood biomarker, and more research is necessary. Lastly, BAT3's involvement in stress response and protein homeostasis suggests potential utility as a biomarker for tissue damage or disease, yet more extensive research is needed for its validation. Overall, while the theoretical and biological relevance of these genes is notable, robust demonstration of their clinical value remains the next necessary step. Further controlled studies are warranted to fully evaluate, optimize, and validate the potential of SLC2A1, ABCC4, WNK1, ODC1, and BAT3 as effective blood transcriptional biomarkers.

Relevance to circulating leukocytes immune biology: SLC2A1, ABCC4, ODC1, and BAT3 have evidence supporting their relevance to immune function in circulating leukocytes, while WNK1's role is less clear. SLC2A1 encodes the glucose transporter GLUT1, which is not only expressed in leukocytes, but also facilitates inflammatory responses by regulating glucose uptake – a critical leukocyte fuel source (21,22). Similarly, ABCC4's involvement in prostaglandin E2 transport positions it to modulate inflammation in monocytes and other leukocytes (23). ODC1 plays established roles in lymphocyte activation and inflammatory signaling (24,25). BAT3 participates in antigen processing and presentation pathways in certain immune cells (11). In contrast, WNK1 has minimal demonstrated direct links to leukocyte biology, though there is

some speculation it could be connected via its cell signaling functions. Overall, SLC2A1, ABCC4, ODC1, and BAT3 exhibit expression patterns, functions, and preliminary evidence consistent with probable relevance to circulating leukocyte immune biology, whereas WNK1 lacks robust evidence of a significant immunological role. However, further research is still needed to elucidate the precise immune functions and mechanisms for each of these genes in different leukocyte subtypes.

Is a known drug target: SLC2A1, ABCC4, WNK1, and ODC1 have been identified as promising drug targets for various medical conditions. SLC2A1, involved in altered glucose metabolism in cancer, is under investigation for its inhibition via GLUT1 inhibitors as potential anti-cancer agents (26,27). ABCC4, implicated in drug disposition and resistance, particularly in the context of cancer, is being targeted for its role in the efflux and resistance of chemotherapeutic agents (28). WNK1 is a known target for hypertension and diseases related to electrolyte imbalance, with WNK1 inhibitors being explored as anti-hypertensive agents (29). ODC1, playing a role in polyamine biosynthesis, is also being investigated through ODC1 inhibitors for its anti-cancer potential (30). In contrast, BAT3, despite its identified role in cellular processes like protein homeostasis and cell proliferation, has not yet been substantiated as a current drug target.

Of therapeutic relevance for immune-mediated diseases: SLC2A1, ABCC4, WNK1, ODC1, and BAT3 have potential implications in the therapeutic targeting of immune-mediated diseases. SLC2A1, given its role in cellular metabolism, has been identified as potentially relevant in immune-mediated conditions, such as type 1 diabetes, where altered energy metabolism is common; GLUT1 inhibitors are of particular interest in this context (31) . ABCC4, involved in prostaglandin E2 transport, could also be relevant in immune-mediated diseases, although further research is required for validation (32). WNK1, despite its primary role in hypertension and electrolyte homeostasis, lacks specific evidence to support its relevance in immune-mediated conditions (33). ODC1, known for its role in cell growth and differentiation, could be pertinent to both cancer and immune-mediated diseases like rheumatoid arthritis (34). Lastly,

BAT3, involved in cell proliferation, migration, and immune responses, is hypothesized to be relevant for immune-mediated diseases, but definitive evidence is lacking."

5. **Pick(s) for M11.2 based on summarized justifications**

Claude and GPT-4 were prompted to select a top candidate on the basis of the information summarized above in section 4, taking into account all six scoring criteria equally. Both models chose SLC2A1 at the top candidate for M11.2.

<u>GPT-4's justification: "</u>Based on the detailed information you've provided for each of the candidate genes (SLC2A1, ABCC4, WNK1, ODC1, and BAT3), the top candidate gene to be included in a targeted assay aiming to measure differences in transcript abundance for a circulating erythroid cell signature would be SLC2A1. Below are the reasons for this recommendation:

1. Function: The SLC2A1 gene encodes the GLUT1 glucose transporter protein that is a critical player in glucose transport across mammalian cell membranes, including those in erythroid cells. It is therefore directly involved in cellular metabolism, including that of red blood cells.

2. Association with Erythroid Cells or Erythropoiesis: SLC2A1 is the only gene among the candidates that has been specifically stated to have a crucial role in erythroid cells, primarily erythrocytes. This makes it directly relevant for an erythroid cell signature.

3. Used as a Biomarker in Clinical Settings: SLC2A1 is already recognized as a clinical biomarker for GLUT1 deficiency syndrome, making it more likely to have utility in clinical settings and diagnostics.

4. Potential Value as a Blood Transcriptional Marker: Among the genes listed, SLC2A1 is noted as potentially useful in conditions like diabetes and certain cancers where glucose

metabolism is crucial. While validation is needed, it stands as the most likely candidate for effective use as a blood transcriptional marker for erythroid cells.

5. Relevance to Circulating Leukocytes Immune Biology: The gene is also noted to have relevance to immune function in circulating leukocytes. It facilitates inflammatory responses by regulating glucose uptake, a critical fuel source for leukocytes. This is particularly pertinent if your assay aims to measure changes that may be of relevance to immune function.

6. Is a Known Drug Target: It is under investigation for its inhibition via GLUT1 inhibitors as potential anti-cancer agents, adding another layer of clinical relevance.

7. Of Therapeutic Relevance for Immune-Mediated Diseases: SLC2A1 is mentioned as potentially relevant in immune-mediated conditions like type 1 diabetes, making it relevant for broader clinical applications.

In summary, based on its function, association with erythroid cells, clinical use as a biomarker, potential as a blood transcriptional marker, relevance to circulating leukocytes, status as a drug target, and therapeutic relevance for immune-mediated diseases, SLC2A1 appears to be the most relevant and promising gene to include in a targeted assay for measuring differences in transcript abundance for a circulating erythroid cell signature."

Claude's justification: Based on the information provided, the top candidate gene to include in a targeted assay aiming to measure differences in transcript abundance for a circulating erythroid cell signature would be SLC2A1.

The key reasons supporting SLC2A1 as the top choice are:

- SLC2A1 plays a crucial role in erythroid cells, encoding the GLUT1 glucose transporter protein that facilitates essential glucose transport in red blood cells. This establishes its functional importance in erythrocyte biology.
- SLC2A1 is already recognized as a clinical biomarker for GLUT1 deficiency syndrome, demonstrating its utility as a blood marker.
- As a glucose transporter, SLC2A1 levels could serve as a significant transcriptional marker in conditions where glucose metabolism is altered, like diabetes. This supports its potential value as a blood biomarker.
- SLC2A1 is expressed in leukocytes and helps regulate inflammatory responses, suggesting relevance to circulating leukocyte immune biology.
- SLC2A1 is an identified drug target, with inhibitors being explored for anti-cancer potential, showing therapeutic relevance.
- SLC2A1 has implicated relevance in immune-mediated diseases like diabetes through its role in cellular metabolism and energy regulation.

In comparison, while ABCC4, WNK1, ODC1 and BAT3 have merits, they lack the definitive erythroid cell functional links, established clinical biomarker evidence, and clear therapeutic relevance that makes SLC2A1 the most compelling choice. However, further validation through controlled studies is still needed to optimize and confirm the utility of SLC2A1 as a blood transcriptional biomarker.

6. Examining expression patterns of top 5 candidates across leukocyte populations

The expression patterns of the top 5 candidate genes were examined across diverse leukocyte populations and hematopoietic precursors using three reference transcriptomic datasets.

6.1 Densely interconnected transcriptional circuits control cell states in human hematopoiesis – (GSE24759)
http://developmentalimmunology.gxbsidra.org/dm3/geneBrowser/show/4000026

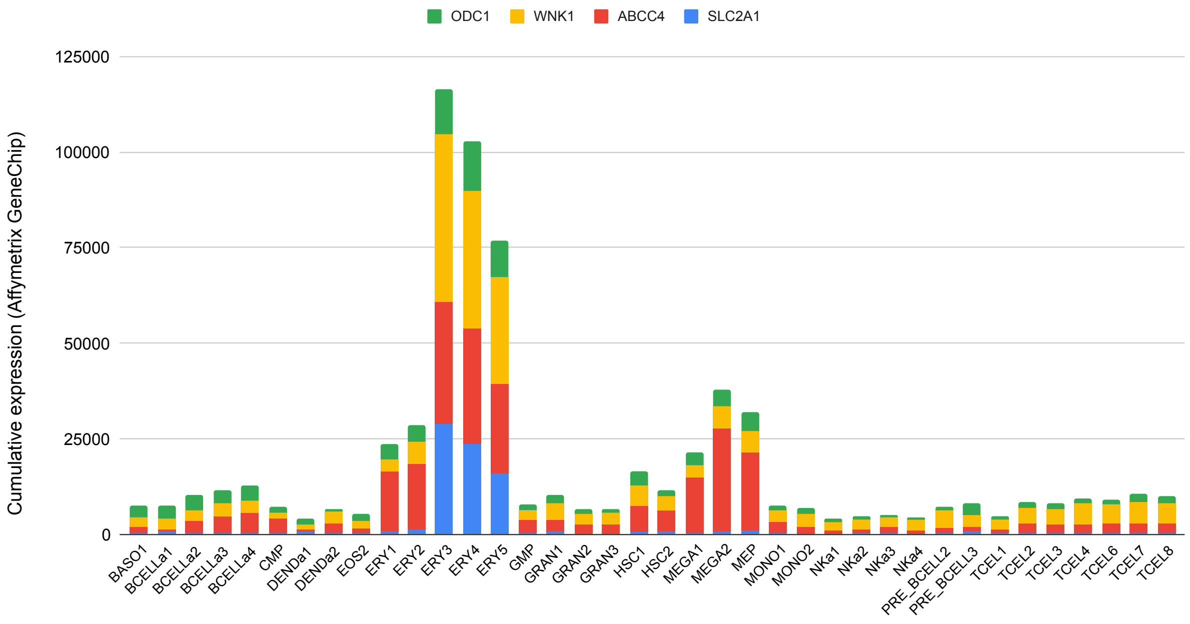

Figure 3: This stacked bar graph shows the expression levels of four of the five candidate genes. One of the candidate genes, BAT3, was not profiled in this dataset). ERY3-5 populations are CD71+ GLYA+ erythroid cells. The original article provides full experimental details: [ref]. The web link above also provides access to more complete information about this datasets as well as individual gene profiles.

6.2 Next generation sequencing of human immune cell subsets across diseases – (GSE60424)
http://cd2k.gxbsidra.org/dm3/geneBrowser/show/4000098

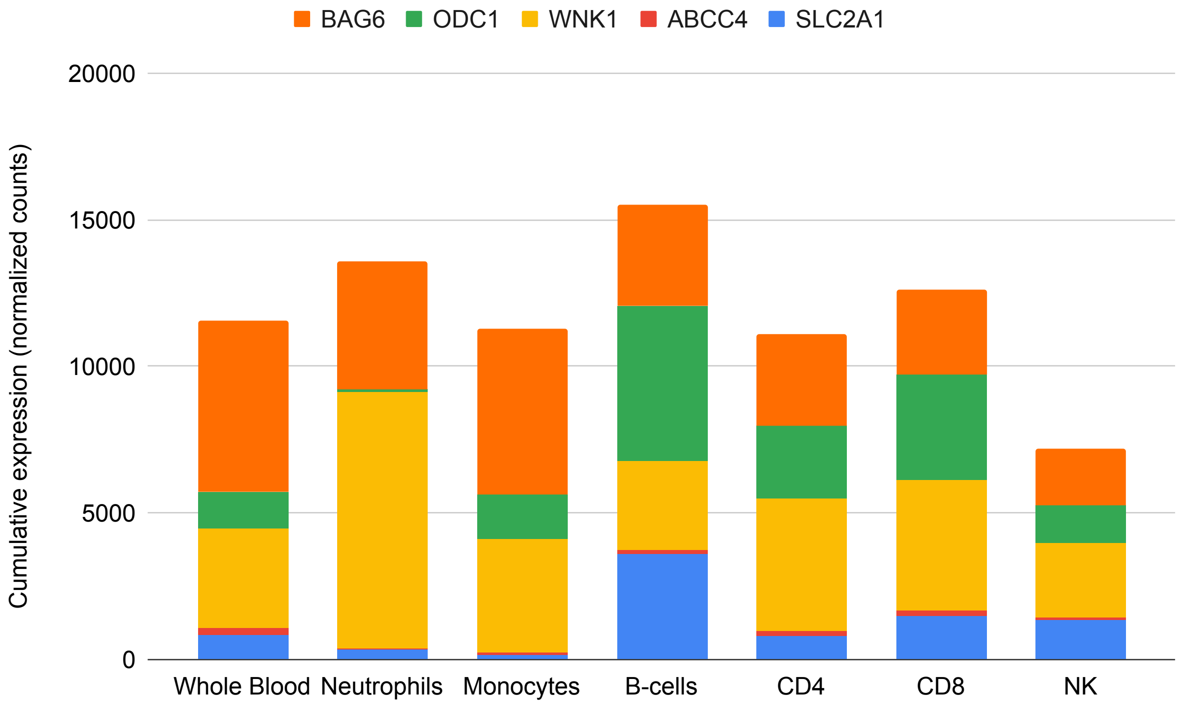

Figure 4: This stacked bar graph shows the expression levels measured by RNAseq of the five candidate genes across six leukocyte populations and whole blood. The abundance is shown for each gene in each population for an average of up to 20 samples. Additional experimental details are accessible via the link provided above and is available as part of this sample series GEO record (GSE60424).

6.3 BloodGen3 reference cohorts (GSE100150)

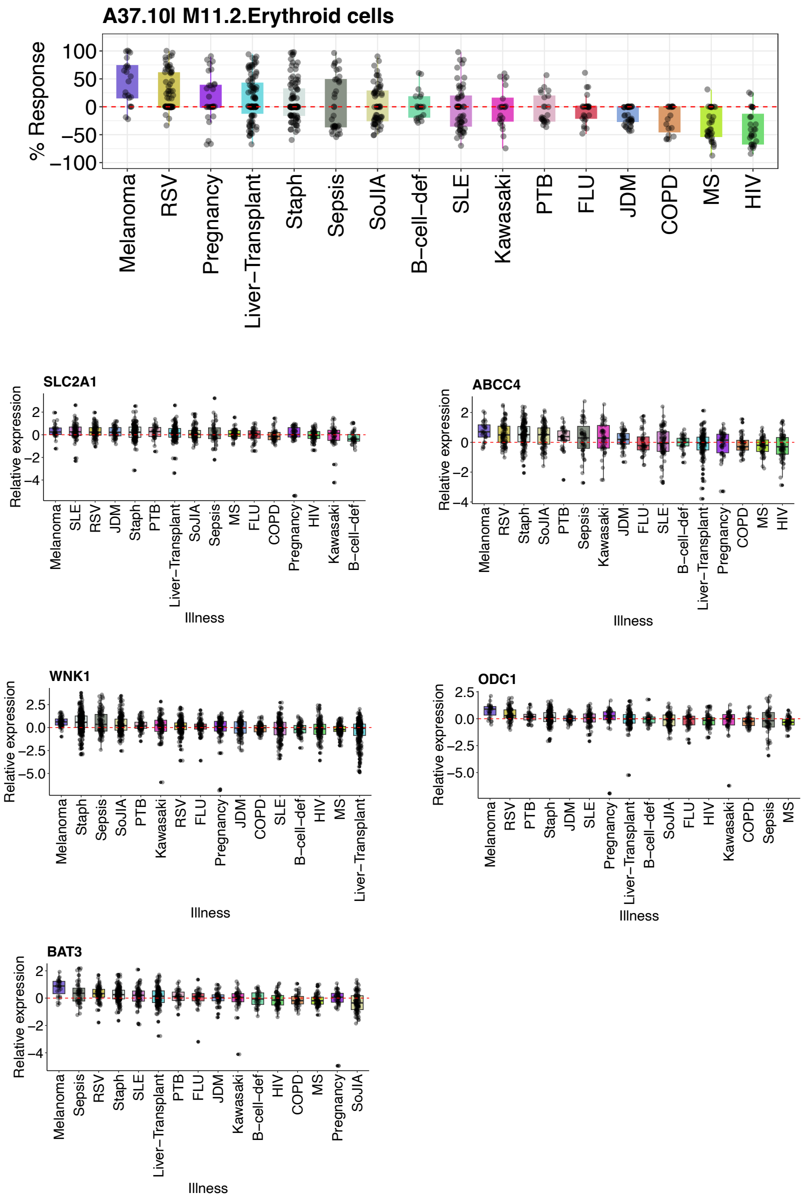

Figure 5: The box plot in panel A below shows the changes in abundance of transcripts comprising the M11.2 module, expressed within a cohort as the percentage of genes showing differential expression between each patient and the respective set of control subjects for this cohort. Differential expression is based on a fixed cut-off (Log2 Fold Change > 1.5, difference >10). These values are expressed as % module activity and can range from +100% (all genes are upregulated) to -100% (all genes are down regulated). The Box plots in panel B show expression levels for the top 5 scoring M11.2 genes in patient cohorts relative to respective control subjects matched for demographics variables.

7. **Overall pick for M9.2 based on summarized justifications and reference transcriptional datasets.**

GPT-4 and Claude were asked to pick a top candidate. They were told to consider the fact-checked summary justifications that were created earlier. They were also told to factor in the gene expression data from the three reference datasets. By using multi-tier prompting, GPT-4 and Claude could integrate all this information to select the best candidate.

While both models initially selected SLC2A1 based on the summaries, Claude, taking into account the transcriptional profiling data, picked instead WNK1. We concur with this choice given that WNK1 shows indeed a higher level of restriction to Erythroid cells in the Novershtern dataset. The restriction of SLC2A1 to B-cells in the Speake dataset is also of concern. **Therefore, WNK1 is the final pick for module M11.2**

The transcription of the final round of prompting is shown below.

**Prompts**	**CLAUDE**	**GPT-4**
PROMPT 1: Based on the summary below, please recommend the top candidate gene to include in a targeted blood transcriptional profiling panel. The intent is for the selected gene to be representative of an erythroid cell signature associated with response to mRNA vaccines, severity of RSV infection and that found to be expressed at high levels in patients with metastatic melanoma and in liver transplant recipients. Please weigh the following criteria equally in your recommendation: 1) Relevance to erythroid cells and erythropoiesis, 2) Current use as a biomarker, 3) Potential use as a blood transcriptional biomarker, 4) Relevance to leukocyte immune biology, 5) Status as a known drug target, 6) Therapeutic relevance for immune mediated diseases: *[input*	Based on the provided summary and criteria, I would recommend SLC2A1 as the top gene to include in the targeted blood transcriptional profiling panel.<br><br>The key reasons are:<br><br>- SLC2A1 has a clear and direct role in erythroid cells and erythropoiesis through encoding the GLUT1 glucose transporter, which facilitates essential glucose transport in red blood cells. This aligns well with the intent for a gene relevant to erythroid biology.<br><br>- SLC2A1 is already recognized as a clinical biomarker for diagnosing GLUT1 deficiency syndrome. This satisfies the criterion for current use as a biomarker.	Based on the detailed information provided, the SLC2A1 gene appears to be the most suitable candidate for inclusion in a targeted blood transcriptional profiling panel, particularly when considering the six equally weighed criteria:<br><br>Relevance to erythroid cells and erythropoiesis: SLC2A1 is directly implicated in erythroid cells, primarily erythrocytes, facilitating the crucial process of glucose transport. This is pivotal for cellular metabolism, including that of red blood cells. This makes it highly relevant to erythropoiesis.<br><br>Current use as a biomarker: SLC2A1 is a recognized clinical biomarker for GLUT1 deficiency syndrome. It's already utilized in clinical settings

summarized and fact-checked justifications from Step 5]	- As a key player in glucose metabolism, SLC2A1 has strong potential utility as a blood transcriptional biomarker in conditions like diabetes and cancer where metabolism is altered. This fulfills the biomarker potential criterion.<br><br>- SLC2A1 is expressed in leukocytes and helps regulate inflammatory responses, indicating relevance to immune biology. This matches the immune biology criterion.<br><br>- SLC2A1 is an emerging drug target, with GLUT1 inhibitors under investigation as possible cancer therapeutics. This satisfies the drug target criterion.<br><br>- Finally, SLC2A1's metabolic role makes it potentially relevant for targeting immune-mediated diseases like type 1 diabetes. This addresses the immune disease relevance criterion.<br><br>In summary, SLC2A1 ranks highly across all the stated criteria, especially for its erythroid cell links, existing biomarker use, biomarker potential, and immune relevance. For these reasons, I recommend it as the top gene for inclusion in the desired transcriptional profiling panel.	for diagnosis, enhancing its relevance as a biomarker.<br><br>Potential use as a blood transcriptional biomarker: SLC2A1 has potential utility in conditions where glucose metabolism is altered, such as diabetes and certain cancers. Although further validation is needed, its potential for this role is significant.<br><br>Relevance to leukocyte immune biology: SLC2A1 is expressed in leukocytes and facilitates inflammatory responses by regulating glucose uptake. This directly ties it to immune function in circulating leukocytes, a significant point considering the gene needs to be relevant to both erythroid and leukocyte cell types.<br><br>Status as a known drug target: SLC2A1 is under investigation as a promising drug target, particularly for anti-cancer agents through GLUT1 inhibitors. This makes it relevant in therapeutic contexts.<br><br>Therapeutic relevance for immune-mediated diseases: Given its role in cellular metabolism, SLC2A1 has implications for immune-mediated diseases like type 1 diabetes. GLUT1 inhibitors are particularly interesting in this therapeutic context.<br><br>In summary, SLC2A1 seems to best fulfill all six criteria: it is relevant to both erythroid cells and immune biology, has current and potential future utility as a biomarker, is a known drug target, and has therapeutic relevance in immune-mediated diseases. Therefore, this gene appears to be the top candidate for further investigation in a targeted blood transcriptional profiling panel.

PROMPT 2: Now that you have provided an initial recommendation based on the summary, take into account the following RNA-sequencing expression data from key immune cell types and whole blood samples: *[input textualized transcriptional data].* Given this additional RNA-seq expression data, does your recommended gene selection remain the same? Please explain which gene you would recommend at this stage and why, incorporating both the summarized information *provided earlier and RNA-seq data in your rationale."*	After reviewing the additional RNA-seq expression data for the genes in question across different immune cell types and whole blood, I would modify my original recommendation and now suggest WNK1 as the top gene for inclusion in the targeted transcriptional profiling panel.<br><br>The key factors in this updated recommendation are:<br><br>- The RNA-seq data indicates WNK1 is expressed at moderately high levels across all examined immune cell types, including neutrophils, monocytes, B cells, T cells, and NK cells. This demonstrates broader relevance to circulating leukocyte biology compared to more variable expression of the other genes.<br>- WNK1 expression measured by RNA-seq is also substantial in whole blood samples, suggesting suitability as a blood-based biomarker.<br>- While WNK1 may lack the direct erythroid cell connections of SLC2A1, the summary indicates it still has potential utility as a biomarker for conditions like hypertension. Its expression levels complement this potential use.<br>- Additionally, WNK1 meets several other original criteria, including being an emerging drug target and having links to electrolyte homeostasis, which could prove therapeutically relevant.<br>- Meanwhile, the RNA-seq data reveals SLC2A1 expression is high in some cell types like B cells, but lower in others like neutrophils and monocytes. Its expression pattern appears more selective.<br>- ABCC4, ODC1 and BAT3 also show variable or more specialized expression compared to the broader WNK1 expression.<br>In summary, the RNA-seq data provides evidence that WNK1 may be more widely and consistently	Upon re-evaluation of the RNA-seq data alongside the previously provided summary information, my recommendation for SLC2A1 as the top candidate gene for inclusion in a targeted blood transcriptional profiling panel still stands. Here's why:<br><br>Criteria Recap and SLC2A1:<br><br>Relevance to erythroid cells and erythropoiesis: SLC2A1 is crucial in erythroid cells and is involved in glucose transport.<br><br>Current use as a biomarker: It is already a clinical biomarker for GLUT1 deficiency syndrome.<br><br>Potential use as a blood transcriptional biomarker: Relevant in several other medical conditions.<br><br>Relevance to leukocyte immune biology: Expressed in leukocytes and supports their metabolism.<br><br>Status as a known drug target: Identified as a drug target in certain conditions, adding to its utility.<br><br>Therapeutic relevance for immune-mediated diseases: It is potentially relevant for immune-mediated diseases like type 1 diabetes.<br><br>RNA-seq Data for SLC2A1:<br><br>Whole Blood: 42<br>Neutrophils: 17<br>Monocytes: 13<br>B-cells: 180<br>CD4 T-cells: 40<br>CD8 T-cells: 74<br>NK cells: 96<br>SLC2A1 shows a diverse expression profile across multiple immune cell types. Particularly high levels in B-cells could be relevant for immune responses, and it is sufficiently expressed in other immune cell types.

	expressed across diverse immune cell populations compared to the other genes analyzed. This, combined with meeting multiple original criteria like biomarker potential, establishes it as the top choice for the transcriptional panel based on both the summary and expression data.	Comparison with Other Genes:<br><br>ABCC4: Low expression across the board. Less relevant to both erythroid and immune cells.<br><br>WNK1: High expression, especially in neutrophils and monocytes, but less well-studied in the context of erythroid cells and its role as a biomarker isn't established.<br><br>ODC1: Shows higher expression in B-cells and CD8 T-cells but lacks the robust coverage across both erythroid and immune cell biology.<br><br>BAT3: High expression, especially in neutrophils and monocytes. However, its role in erythroid cells isn't well-established, and its clinical utility as a biomarker is unclear.<br><br>In summary, while other genes like WNK1 and BAT3 show high expression in specific immune cell types, they do not offer the same comprehensive range of advantages as SLC2A1, especially in erythroid cell relevance and established use as a biomarker.<br><br>Therefore, considering both the initial criteria and the new RNA-seq data, SLC2A1 remains the most promising candidate for the targeted blood transcriptional profiling panel.
PROMPT 3: Now that you have provided an initial recommendation based on the summary and the RNAseq data, take into account the following microarray expression data from key immune cell types and hematopoietic precursors: *[input textualized transcriptional data].* Given this additional microarray expression data, does your recommended gene selection remain the same? Please explain which gene you would recommend	After reviewing the microarray expression data, I would maintain my recommendation of WNK1 as the top gene for inclusion in the targeted transcriptional profiling panel.<br>The key factors in upholding this selection are:<br>- The microarray data shows WNK1 is robustly expressed across all examined immune cell types, hematopoietic stem and progenitor cells, and stages of differentiation.	*[N/A: input exceed GPT-4 limits]*

at this stage and why, incorporating the summarized information and RNA-seq data provided earlier, as well as this new microarray data in your rationale.	This reinforces its relevance to diverse immune biology.<br>- WNK1 expression levels by microarray are substantial in whole blood progenitor cells like CMPs and HSCs, indicating suitability as a blood-based marker.<br>- The microarray data is consistent with the earlier RNA-seq data showing broad WNK1 expression. Both datasets indicate WNK1 could serve as a pan-immune biomarker.<br>- Other genes like SLC2A1 and ODC1 have more variable or lineage-specific expression by microarray, making them less suitable as broad immune markers.<br>- While WNK1 may lack the erythroid connections of SLC2A1, the microarray and RNA-seq data confirm its potential utility as a biomarker based on its consistent immune expression.<br>- WNK1 still meets the original criteria of being a drug target and having links to immune-mediated disease through its cell signaling roles.<br><br>In summary, the combined RNA-seq and microarray datasets provide compelling evidence that WNK1 has widespread expression across diverse immune cells and whole blood, supporting its use as a broad transcriptional immune biomarker. The collective expression data affirms WNK1 as the top choice over genes with more specialized expression.	
PROMPT 4: Now that you have provided an initial recommendation based on the summary and the immune cells RNAseq and microarray data, take into account the following averaged log2 fold changes in RNA abundance in patient cohorts compared to controls. *[input textualized transcriptional data].* Given this additional data, does your	After reviewing the patient cohort expression data, I would still recommend WNK1 as the top gene for inclusion in the targeted transcriptional profiling panel.<br><br>The key factors in maintaining this selection are:<br><br>- The microarray data shows WNK1 is differentially expressed in a wide	[GPT4 lost the thread of the conversation and did not select a top candidate at this step].<br><br>First of all, the Log2 fold change values indicate the direction and magnitude of differential expression of these genes in various conditions relative to healthy controls. A positive Log2 fold

recommended gene selection remain the same? Please explain which gene you would recommend at this stage and why, incorporating the summarized information, the leukocytes RNA-seq and microarray data provided earlier, as well as these new patient cohort profiles in your rationale.	range of disease states compared to controls, including bacterial infections, viral infections, autoimmunity, cancer, and organ transplantation. This demonstrates its broad utility as an immune-related biomarker.<br><br>- WNK1 expression changes are substantial in diseases like HIV, influenza, and staphylococcus aureus infection. This indicates it could distinguish between different infectious or inflammatory conditions.<br><br>- WNK1 expression differs in conditions like pregnancy and multiple sclerosis, suggesting utility beyond just infectious disease applications.<br><br>- Other genes like SLC2A1 and ODC1 show more modest expression changes in fewer disease states, indicating more limited biomarker potential.<br><br>- The RNA-seq and microarray immune cell data still supports WNK1 as a pan-immune marker, while the cohort data provides evidence it could detect immune disturbances in humans.<br><br>- WNK1 retains its advantages as a drug target and its widespread immune expression based on earlier results.<br><br>In summary, the patient cohort expression changes affirm WNK1's capability to detect immune perturbations in whole blood across diverse inflammatory states. Combined with the immune cell expression patterns, WNK1 remains the top choice gene for its breadth of expression, biomarker potential, and drug target status.	change means that the gene is upregulated (more expressed) in the patient cohort relative to controls, while a negative Log2 fold change indicates downregulation (less expressed).<br><br>Here are some general observations based on the provided data:<br><br>SLC4A1<br>Downregulated in: B-cell deficiency, COPD, acute influenza, HIV, juvenile dermatomyositis, sepsis, systemic lupus erythematosus, and systemic onset juvenile idiopathic arthritis.<br>Upregulated in: Kawasaki disease, metastatic melanoma, multiple sclerosis, pregnancy, active pulmonary tuberculosis, acute respiratory syncytial virus infection, staphylococcus aureus infection, and liver transplant recipients under maintenance therapy.<br>ABCC4<br>Downregulated in: B-cell deficiency, HIV, sepsis, and multiple sclerosis.<br>Upregulated in: COPD, acute influenza, juvenile dermatomyositis, Kawasaki disease, metastatic melanoma, pregnancy, active pulmonary tuberculosis, acute respiratory syncytial virus infection, staphylococcus aureus infection, and systemic onset juvenile idiopathic arthritis.<br>WNK1<br>Downregulated in: B-cell deficiency, COPD, HIV, sepsis, multiple sclerosis, and systemic lupus erythematosus.<br>Upregulated in: Acute influenza, Kawasaki disease, metastatic melanoma, pregnancy, active pulmonary tuberculosis, acute respiratory syncytial virus infection, staphylococcus aureus infection, and systemic onset juvenile idiopathic arthritis.<br>ODC1<br>Downregulated in: B-cell deficiency, COPD, acute influenza, HIV, sepsis, multiple sclerosis, acute respiratory

		syncytial virus infection, and systemic onset juvenile idiopathic arthritis.<br>Upregulated in: Juvenile dermatomyositis, metastatic melanoma, pregnancy, active pulmonary tuberculosis, and staphylococcus aureus infection.<br>BAT3 (Incomplete)<br>Downregulated in: B-cell deficiency, COPD, HIV, and Kawasaki disease.<br>Upregulated in: Acute influenza and metastatic melanoma (Based on incomplete data).<br>It's noteworthy that each gene shows varied expression across different disease states. For example, SLC4A1 is downregulated in infectious diseases like HIV but upregulated in conditions like metastatic melanoma. This suggests that each gene might be participating in different physiological pathways depending on the disease context.<br><br>Clinical Implications:<br>Targeted Therapies: Understanding the role of these genes in specific diseases could lead to more targeted therapies.<br><br>Disease Progression and Severity: The degree of change might indicate the severity or stage of the disease.<br><br>Biomarkers: These genes could potentially be used as biomarkers for certain diseases, aiding in diagnosis or treatment.<br><br>Side Effects: For those undergoing therapy (like liver transplant recipients), monitoring these genes might provide insights into side effects or treatment efficacy.<br><br>Drug Development: This data could be beneficial for the development of drugs targeting these specific genes or the pathways they are involved in.

		Next Steps:<br>Functional Studies: To understand the exact role of these genes in each disease.<br><br>Statistical Analysis: To ascertain the significance of these changes.<br><br>Patient Cohort Expansion: To validate these findings in a larger population.<br><br>Longitudinal Studies: To see how gene expression changes with disease progression.<br><br>Correlation with Clinical Data: To see if these gene changes correlate with disease symptoms or outcomes.
PROMPT 5: Could you please summarize the key conclusions you have drawn from the conversation so far?	Here is a summary of the key conclusions I have drawn so far in our conversation about selecting a gene for a targeted blood transcriptional profiling panel:<br><br>Initial Recommendation Based on Provided Summary<br><br>I originally recommended SLC2A1 based on its associations with erythroid cells, existing use as a biomarker, biomarker potential, immune relevance, and drug target status according to the summary.<br><br>Recommendation Changed to WNK1 After Reviewing RNA-seq Data<br>With the RNA-seq data showing WNK1 had broader and more consistent immune cell expression, I updated my recommendation to WNK1 for its potential as a pan-immune biomarker.<br><br>WNK1 Recommendation Affirmed by Microarray Immune Cell Data<br>The microarray data was consistent with the RNA-seq results, showing widespread WNK1 expression	*[GPT-4 lost the thread of the conversation].*

	across diverse immune cell types and stages. This further supported WNK1 as a broad immune marker.<br><br>WNK1 Still Recommended After Reviewing Patient Cohort Data. The patient cohort microarray data revealed WNK1 was differentially expressed in a wide range of diseases compared to controls. This demonstrated its utility as an immune-related biomarker.<br><br>Key Advantages of WNK1:<br><br>- Broad immune cell and whole blood expression<br>- Biomarker potential across diverse conditions<br>- Status as an emerging drug target<br>- Greater consistency than other candidate genes<br><br>In summary, while my initial recommendation was SLC2A1, after reviewing multiple datasets, I believe WNK1 is the top choice for inclusion in the desired transcriptional profiling panel based on its widespread immune expression, biomarker capabilities, and drug target potential.	
