# Supplementary material for: Harnessing large language models (LLMs) for candidate gene prioritization and selection
Source: J Transl Med. 2023 Oct 16;21:728. doi: 10.1186/s12967-023-04576-8 (PMC10580627; doi:10.1186/s12967-023-04576-8)
Supplement: Supplementary file 4 — Additional file 4: Is the prioritization report for module M11.3 authored by Basirudeen Kabeer. The file is shared in the PDF format. [file 12967_2023_4576_MOESM4_ESM.pdf]

M11.3 Prioritization report

Prepared by: Basirudeen Kabeer (Sidra Medicine, Doha, Qatar)

1. Composition

The module M11.3 belongs to Aggregate A37 from the BloodGen3 fixed module repertoire [1,2]. This module aggregate is associated with circulating erythroid cells. It was found to be associated with RSV disease severity (3). We showed in the same study that the abundance of A27 transcripts is also elevated in the blood of patients with stage IV melanoma [3].

It comprises 24 genes: ASCC2, C18ORF10, DPM2, EPB49, FBXO7, GATA1, HAGH, HEMGN, HMBS, KEL, LOC284422, LOC440359, LOC441081, MBNL3, MYL4, PDZK1IP1, PHOSPHO1, RUNDC3A, SESN3, TESC, TGM2, TRIM10, TSPAN5, VWCE.

2. Functional convergence

Functional associations were identified among the genes constituting M11.3 using GPT-4 (see Step 2 method for details).

**Functional Themes**	**Associated Genes**	**Comments**
Blood cell development and function	GATA1, EPB49, HMBS	GATA1 is a transcription factor critical for erythroid development and function. EPB49 (also known as EL2 or TMEM38B) is involved in the maintenance of erythrocyte shape and mechanical properties. HMBS is involved in heme biosynthesis, a critical process for erythrocyte function.
Protein regulation and signaling	FBXO7, TGM2, MBNL3, SESN3	These are involved in various aspects of protein regulation and cellular signaling, including FBXO7 (protein ubiquitination), TGM2 (protein cross-linking), MBNL3 (alternative splicing regulation), and SESN3 (regulation of reactive oxygen species).
Intracellular trafficking and membrane dynamics	DPM2, TSPAN5	These genes are involved in various aspects of intracellular trafficking and membrane dynamics. These include DPM2 (involved in glycosylation, a process important for protein folding and stability), and TSPAN5 (a member of the tetraspanin family, which is known to be involved in various cellular processes, including intracellular trafficking).
Response to DNA damage	ASCC2	ASCC2, part of the ASC-1 complex, is known to play a role in the cellular response to DNA damage.

3. Scoring and prioritization

Genes were scored on six criteria using GPT-4 and Claude. The scores were averaged, and candidate genes were ranked according to their cumulative scores (**Figure 1**, Methods: Step 3 and Step 4).

The two LLMs were requested to score each gene on the following six statements:

a. The gene is associated with erythroid cells or erythropoiesis.

b. The gene is currently being used as a biomarker in clinical settings.

c. The gene has potential value as a blood transcriptional biomarker.

d. The gene is relevant to circulating leukocytes immune biology.

e. The gene is a known drug target.

f. The gene is therapeutically relevant for immune-mediated diseases.

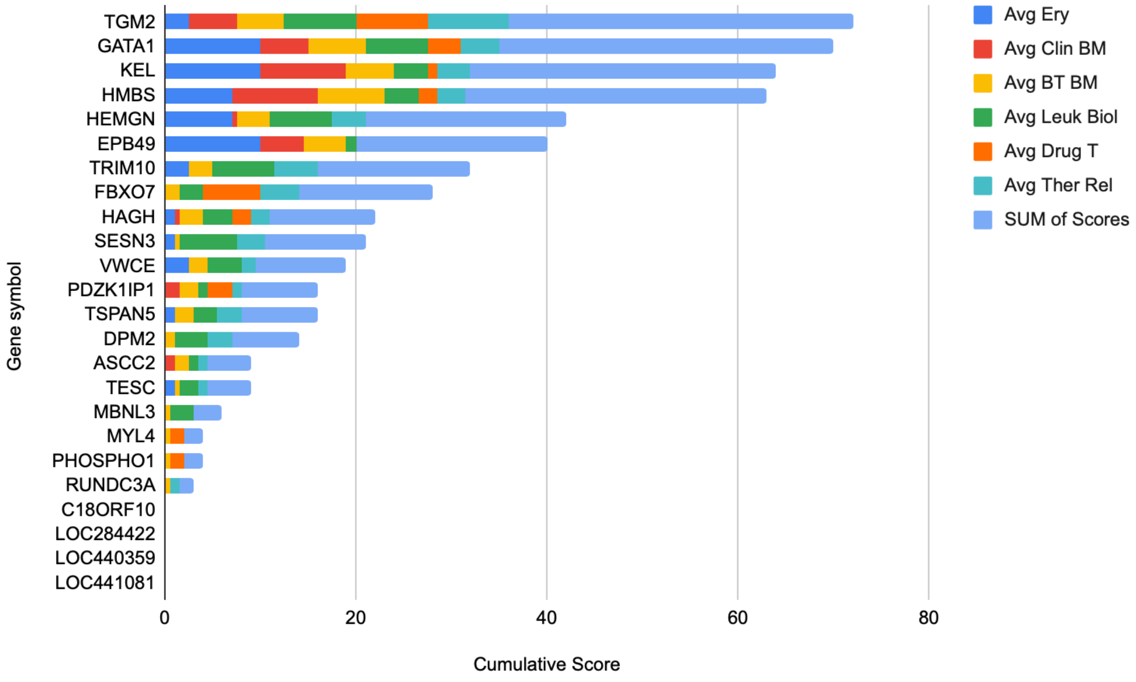

Figure 1: The stacked bar graph shows cumulative scores across six criteria for the 24 M11.3 genes. The top five genes selected for further evaluation were: TGM2, GATA1, KEL, HMBS, and HEMGN.

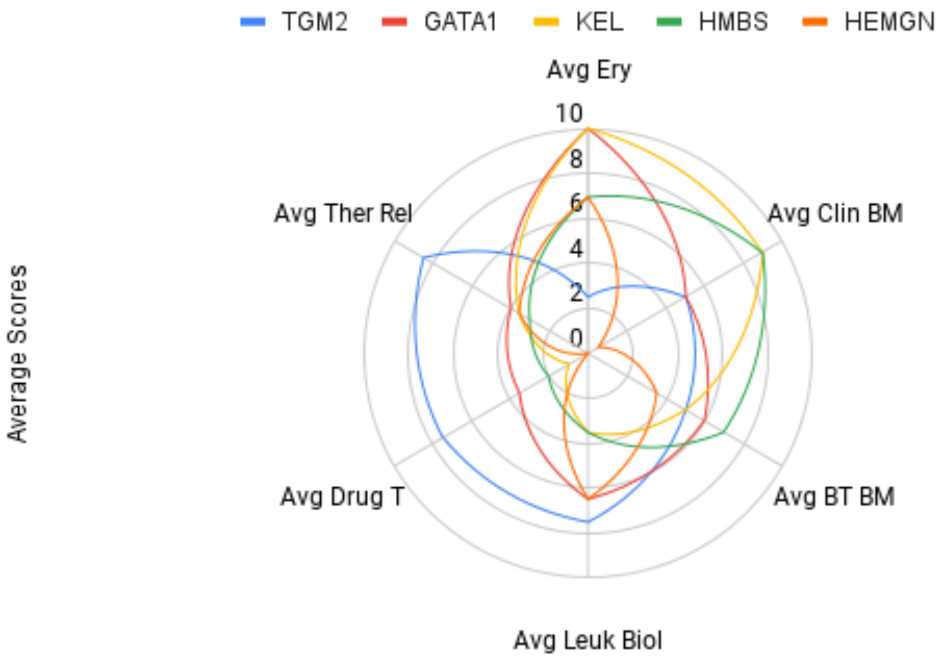

Figure 2: The radar plot represents individual scores for the 5 top scoring genes: TGM2, GATA1, KEL, HMBS, and HEMGN.

4. Knowledge-driven evaluation of top five candidates

Justifications given by the GPT-4 and Claude for the scores provided across the 6 criteria were compiled and summarized by GPT-4 (Methods: Step 5). For each statement pertinent references were retrieved using GPT-4 or Claude, with the relevance of each reference checked and attributed manually by the researcher authoring this report.

Function: "Transglutaminase 2, encoded by the TGM2 gene, catalyzes protein crosslinking and the conjugation of polyamines to proteins. It plays a pivotal role in various cellular processes such as apoptosis, cellular differentiation, wound healing, cellular signaling, extracellular matrix assembly, cell-matrix interactions, and cytoskeletal organization, and it may influence certain autoimmune diseases [4,5]. The GATA1 gene encodes the GATA-1 transcription factor, vital for the development and differentiation of erythroid cells and some white blood cells like megakaryocytes and eosinophils. Mutations in GATA1 are linked to disorders like X-linked

dyserythropoietic anemia and thrombocytopenia [6]. The KEL gene, integral to the Kell blood group system, encodes a transmembrane protein that ensures structural integrity and mediates cell-cell interactions. Mutations in KEL can result in conditions such as McLeod syndrome and Kx red blood cell incompatibility [7,8]. HMBS is responsible for encoding the hydroxymethylbilane synthase enzyme, a crucial component in heme biosynthesis that facilitates the conversion of porphobilinogen molecules into hydroxymethylbilane. Defects in HMBS can lead to acute intermittent porphyria [9]. Lastly, the HEMGN gene encodes the hemogen protein, predominantly found in bone marrow and fetal liver. While its association with hematopoiesis and erythroid cell development is evident, its exact function remains elusive [10]."

a. Relevance to erythroid cells and erythropoiesis: "Transglutaminase 2 (TGM2), while not directly associated with erythroid cells or erythropoiesis, plays a pivotal role in cellular processes, including cellular differentiation. Evidence associating TGM2 with erythropoiesis, the development of red blood cells, is currently limited. In contrast, the transcription factor GATA1 exhibits a substantial association with erythroid cells and erythropoiesis. It is indispensable for the development of erythroid precursors and the maturation of red blood cells [11]. Kell blood group antigen (KEL) also exhibits strong association with erythroid cells, owing to its essential role in red blood cell function. KEL is prominently expressed in erythroid cells, where it defines the Kell blood group antigens and aids in the structure and adhesion of red blood cells [12]. Hydroxymethylbilane Synthase (HMBS) is relevant to erythroid cells and erythropoiesis due to its significant role in the heme synthesis pathway, a crucial process for erythropoiesis [13]. Evidence linking HMBS to erythropoiesis is limited, with its principal function in the heme biosynthesis pathway. Finally, Hemogen (HEMGN) shows substantial relevance to erythroid cells or erythropoiesis due to its role in hematopoiesis [14,15]. However, evidence associating HEMGN with erythropoiesis or red blood cell development is limited".

b. Is used as a clinical biomarker: "The Transglutaminase 2 (TGM2) protein, while not utilized extensively as a clinical biomarker, has been implicated in a range of pathologies, including celiac disease and certain neurological disorders [16]. Despite investigations into its diagnostic utility,

TGM2 has not yet gained broad acceptance as a clinical biomarker. The GATA Binding Protein 1 (GATA1) gene is clinically tested for diagnosing specific congenital dyserythropoietic anemias and related conditions [17], but it is not widely employed as a clinical biomarker. The Kell blood group antigen (KEL), however, is used extensively as a biomarker in transfusion medicine to ensure the compatibility of donor blood with the recipient. The clinical applications of KEL testing encompass blood typing and prenatal screening. Hydroxymethylbilane Synthase (HMBS) enzyme deficiency serves as a biomarker for a group of diseases called porphyrias, particularly acute intermittent porphyria (AIP). Consequently, HMBS testing is clinically employed to diagnose such conditions. Hemogen (HEMGN), meanwhile, is not currently used as a standard clinical biomarker, and is thus not broadly utilized in clinical settings."

c. Potential relevance as a blood transcriptional biomarker: "Transglutaminase 2 (TGM2), due to its varied roles in cellular activities, might prove to be a valuable blood transcriptional biomarker, potentially for anemias, autoimmune diseases, or fibrosis, although this necessitates further investigation [18]. Similarly, GATA Binding Protein 1 (GATA1) may hold potential as a blood transcriptional biomarker, primarily due to its integral role in erythropoiesis. It is plausible that GATA1 levels or activity could serve as a biomarker for erythroid disorders, including congenital anemias [19]. Kell blood group antigen (KEL) gene mutations might serve as a blood transcriptional biomarker, particularly for certain anemias or conditions impacting red blood cells, but this hypothesis requires further research [20]. Hydroxymethylbilane Synthase (HMBS), given its role in heme synthesis and its association with porphyrias, might prove useful as a blood transcriptional biomarker. The potential use of HMBS levels or activity as a biomarker for porphyrias or certain anemias warrants further study. Hemogen (HEMGN), due to its role in hematopoiesis and erythroid differentiation, could potentially serve as a valuable blood transcriptional biomarker, although the current evidence supporting its utility is limited and further research is required for validation."

d. Relevance to leukocytes immune biology: "Transglutaminase 2 (TGM2) has significance in circulating leukocytes' immune biology, primarily due to its involvement in cellular signaling and apoptosis, with a high expression level in most leukocytes and participation in inflammatory responses and immune tolerance. GATA Binding Protein 1 (GATA1), while primarily implicated in

erythropoiesis, has relevance to leukocyte immune biology due to its role in the development of certain white blood cells, such as eosinophils and mast cells. The Kell blood group antigen (KEL), known mainly for its role in red blood cell function, indirectly affects circulating leukocytes' immune biology, particularly concerning potential immune responses to transfused red blood cells, even though there is limited evidence of KEL directly impacting leukocyte or immune cell biology [21]. Hydroxymethylbilane Synthase (HMBS), while not directly linked to leukocyte or immune cell biology, has some relevance due to its role in heme synthesis, a vital process for the function of various proteins involved in immune responses [22]. Lastly, Hemogen (HEMGN) holds relevance to circulating leukocytes' immune biology due to its role in hematopoiesis, with its expression seen in certain blood cells, especially those from the myeloid lineage, even though there is limited evidence directly linking HEMGN to immune function."

e. Is a known drug target: "Transglutaminase 2 (TGM2) is recognized as a drug target, particularly in the realms of cancer, inflammation, and fibrotic diseases. TGM2 inhibitors are currently under investigation for a range of conditions, including fibrosis, autoimmunity, and wound healing [23]. While GATA Binding Protein 1 (GATA1) is suggested as a potential drug target for blood-related disorders such as anemia and leukemia, no evidence exists to identify it as a direct drug target currently [24]. Similarly, while the Kell blood group antigen (KEL) could hypothetically be considered in drug development due to its role in blood transfusion compatibility, there is no evidence supporting KEL as a current drug target. Hydroxymethylbilane Synthase (HMBS), despite not being primarily known as a drug target, is indirectly related to therapeutic approaches due to its role in heme biosynthesis, particularly in the context of treatments for porphyrias. Lastly, Hemogen (HEMGN) has not been recognized as a drug target to date."

f. Potential therapeutic relevance for immune-mediated diseases: "Transglutaminase 2 (TGM2), given its role in apoptosis and inflammation, possesses therapeutic relevance for immune-mediated diseases. It is seen as a potential therapeutic target for conditions such as celiac disease, Crohn's disease, and multiple sclerosis. GATA Binding Protein 1 (GATA1), while having a significant role in the development and function of certain white blood cells, does not appear to be directly relevant as a therapeutic target for immune-mediated diseases. However,

it might indirectly influence these conditions through the modulation of erythropoiesis [25]. The Kell blood group antigen (KEL) may hold therapeutic relevance in situations involving immune-mediated hemolysis, like hemolytic disease of the fetus and newborn (HDFN), despite not being directly relevant as a therapeutic target for immune-mediated diseases, given its specificity to erythroid cells and the Kell blood group. Hydroxymethylbilane Synthase (HMBS) and the heme biosynthesis process could be therapeutically relevant for immune-mediated diseases associated with dysregulated heme metabolism, although HMBS itself does not appear to be directly relevant as a therapeutic target. Lastly, Hemogen (HEMGN) could potentially be relevant for therapeutic targeting, especially considering its role in hematopoiesis. However, a deeper understanding of its specific molecular function is required."

5. Pick(s) for M11.3 based on summarized justifications.

We aim to establish a universal immune profiling assay that measures transcript abundance of a panel of genes in whole blood using targeted molecular profiling technology (Immune profiling transcriptome fingerprinting assay). We first attempted to identify top candidates by prompting the GPT-4 and Claude using as input the summarized and fact check justifications obtained from the previous step.

Claude and GPT-4 were prompted to select a top candidate on the basis of the information summarized above in section 4, taking into account all six scoring criteria equally (Methods: Step 6). Both Claude and GPT-4 chose GATA1 and output provided them is given below.

Output from Claude: "Based on the provided information focusing on the association with erythroid cells and erythropoiesis, I would recommend GATA1 as the top candidate gene to be included in a targeted assay aiming to measure differences in transcript abundance for a circulating erythroid cell signature.

The key reasons are:

- GATA1 exhibits the strongest direct association with erythroid cells and erythropoiesis compared to the other genes. It is described as indispensable for erythroid precursor development and red blood cell maturation.

- TGM2, HMBS, and HEMGN show limited or indirect evidence linking them to erythroid cells/erythropoiesis. Their primary roles lie in other pathways.
- While KEL does associate with erythroid cells through its role in red blood cell function and Kell antigens, GATA1 has a more central role in erythropoiesis itself.
- For the specific purpose of measuring a circulating erythroid cell signature, GATA1 stands out as the top choice given its vital and specific function in erythroid development.

In summary, GATA1's critical involvement in erythropoiesis and red blood cell maturation makes it the best candidate for an assay focused on quantifying transcript differences linked to circulating erythroid cells. The provided information highlights its substantial and directed relevance to this cell lineage over the other genes."

<u>Output from GPT-4</u>: "From the provided information, we are aiming to identify a candidate gene that best represents a circulating erythroid cell signature for the purpose of measuring differences in transcript abundance. Let's summarize the relevant attributes for each gene:

1. **TGM2 (Transglutaminase 2)**:
    - Involved in various cellular processes.
    - No direct association with erythroid cells or erythropoiesis.
    - Clinical utility not extensively established.
2. **GATA1 (GATA Binding Protein 1)**:
    - Vital for the development and differentiation of erythroid cells.
    - Directly associated with erythroid cells and erythropoiesis.
    - Clinically tested for diagnosing specific congenital dyserythropoietic anemias.
3. **KEL (Kell blood group antigen)**:
    - Integral to the Kell blood group system, thus directly related to erythroid cells.
    - Clinically used in transfusion medicine.
4. **HMBS (Hydroxymethylbilane Synthase)**:
    - Crucial component in heme biosynthesis.

- Not directly linked to erythropoiesis but indirectly important due to its role in heme production.

5. **HEMGN (Hemogen)**:
    - Found predominantly in bone marrow and fetal liver.
    - Associated with hematopoiesis and erythroid cell development.
    - Exact function in erythroid development remains elusive.

Based on this summarization, the gene that stands out as most directly involved with erythroid cell development and differentiation is **GATA1**. It plays a vital role in the development and differentiation of erythroid cells, and its clinical relevance is already established in diagnosing certain erythroid disorders.

Thus, for a targeted assay aiming to measure differences in transcript abundance for a circulating erythroid cell signature, **GATA1 would be the top candidate gene** to consider based on the provided information."

6. Examining expression patterns of top 5 candidates across leukocyte populations.

The LLMs did not incorporate reference transcriptional profiling data in their evaluation and prioritization of candidate genes. The expression profiles of the top 5 candidate genes were retrieved for three reference datasets. These encompassed diverse leukocyte populations and hematopoietic progenitors as well as the whole blood of patients with a wide range of conditions.

6.1 Densely interconnected transcriptional circuits control cell states in human hematopoiesis – (GSE24759) [26]
http://developmentalimmunology.gxbsidra.org/dm3/geneBrowser/show/4000026

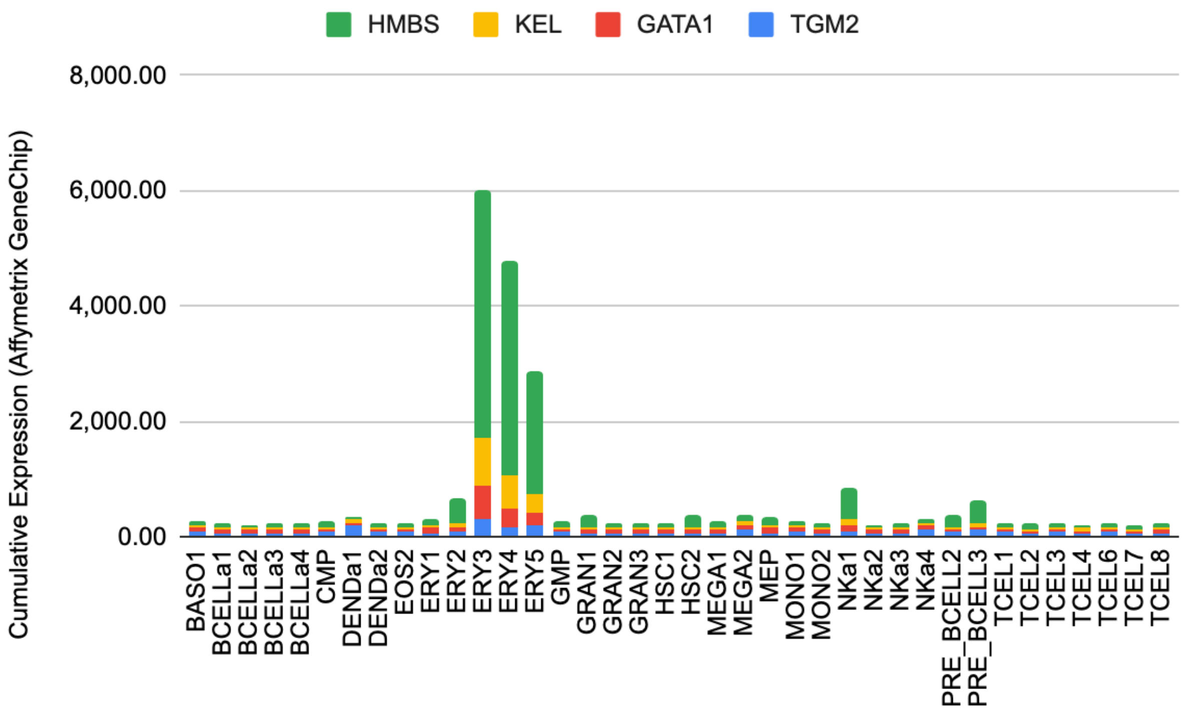

Figure 3: This stacked bar graph shows the expression levels of the five candidate genes.
ERY3-5 populations are CD71+ GLYA+ erythroid cells. The original article provides full experimental details: [26]. The web link above also provides access to more complete information about this dataset as well as individual gene profiles.

6.2 Next generation sequencing of human immune cell subsets across diseases – (GSE60424)

http://cd2k.gxbsidra.org/dm3/geneBrowser/show/4000098 [27]

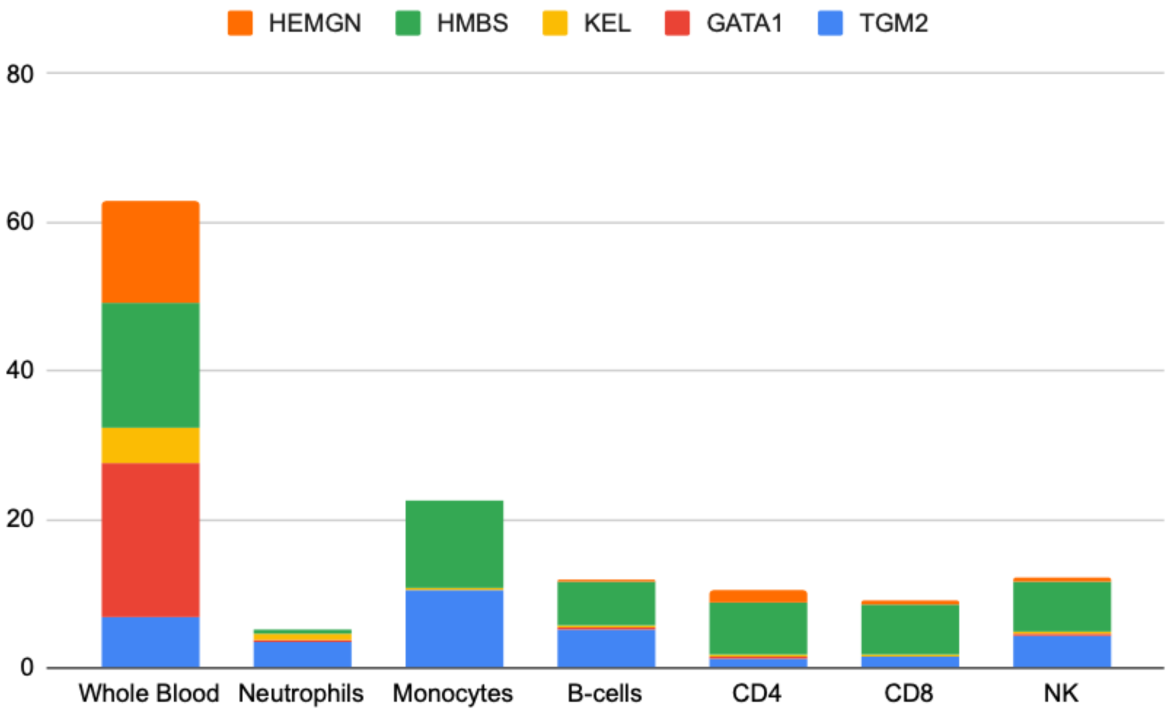

Figure 4: This stacked bar graph shows the expression levels measured by RNAseq of the five candidate genes across six leukocyte populations and whole blood. The abundance is shown for each gene in each population for an average of up to 20 samples. Additional experimental details are accessible via the link provided above and is available as part of this sample series GEO record (GSE60424) [27]

6.3 <u>BloodGen3 reference cohorts (GSE100150)</u>

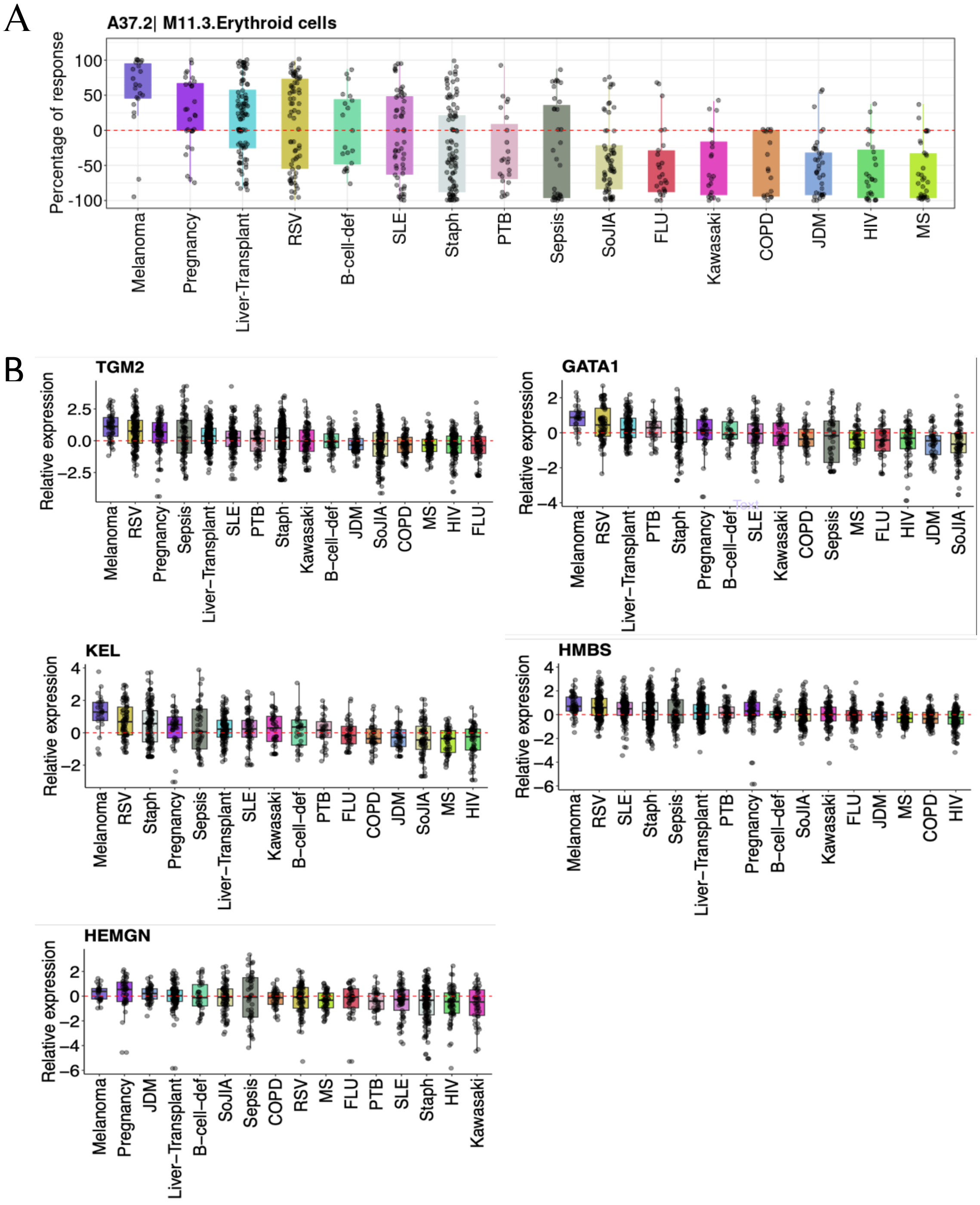

Figure 5: The box plot in panel A shows the changes in abundance of transcripts comprising the M11.3 module, expressed within a cohort as the percentage of genes showing differential expression between each patient and the respective set of control subjects for this cohort. Differential expression is based on fixed cut-off (Log2 Fold Change >1.5, difference>10). These values are expressed as % module activity and can range from 100% (all genes are upregulated) to -100% (all genes are downregulated). The box plot in panel B show expression levels for the

top 5 scoring M11.3 genes in patient cohorts relative to respective control subjects matched for demographic variables.

7. **Overall pick for M11.3 based on summarized justifications and reference transcriptional datasets.**

   Through multi-tier prompting, GPT-4 and Claude were requested to select a top candidate, based on the fact-checked justification summaries generated earlier and taking into account the transcriptional profiles from the three reference datasets.

   This was achieved using multiple prompts. This strategy was successful when using Claude but we GPT-4 could not follow our directives from start to finish when presented with large amounts of textualized transcriptional data.

   Both models chose HMBS as the top option for module M11.3 after analyzing the RNAseq expression data. Based on the explanations provided by LLMs, as well as the expression levels across the leukocyte population and its crucial involvement in heme production, we chose HMBS as the top candidate for M11.3.

   The transcription of the final round of prompting is shown below.

Prompts	CLAUDE	GPT-4
PROMPT 1: Based on the summary below, please recommend the top candidate gene to include in a targeted blood transcriptional profiling panel. The intent is for the selected gene to be representative of an erythroid cell signature associated with response to mRNA vaccines, severity of RSV infection and that found to be expressed at high levels in patients with metastatic melanoma and in liver transplant recipients. Please weigh the following criteria equally in your recommendation: 1) Relevance to erythroid cells and erythropoiesis, 2) Current use as a biomarker, 3) Potential use as a blood transcriptional biomarker, 4) Relevance to leukocyte immune biology, 5) Status as a known drug target, 6) Therapeutic relevance for immune mediated diseases: *[input summarized and fact-checked justifications from Step 5]*	Based on the provided summary and criteria, I recommend GATA1 as the top candidate gene to include in the targeted blood transcriptional profiling panel.<br>The key reasons are:<br>• GATA1 has a substantial association with erythroid cells and erythropoiesis. It plays an indispensable role in erythroid precursor development and red blood cell maturation. This aligns closely with the criteria for relevance to erythroid cells and erythropoiesis.<br>• GATA1 is already clinically utilized as a biomarker for diagnosing certain anemias and related conditions. This satisfies the criteria for current use as a biomarker.<br>• As a transcription factor vital for erythropoiesis, GATA1 has potential for use as a blood transcriptional biomarker, particularly for erythroid disorders	Based on the detailed summary provided and considering the equally weighted criteria of relevance to erythroid cells and erythropoiesis, current use as a biomarker, potential use as a blood transcriptional biomarker, relevance to leukocyte immune biology, status as a known drug target, and therapeutic relevance for immune-mediated diseases, the top candidate gene appears to be the GATA Binding Protein 1 (GATA1). Here's a breakdown according to the criteria:<br>1. **Relevance to erythroid cells and erythropoiesis**: GATA1 is indispensable for the development of erythroid precursors and the maturation of red blood cells. It shows a substantial association with erythroid cells and erythropoiesis.<br>2. **Current use as a biomarker**: While not

	or anemias. This matches the specified criterion.<br>• While not directly implicated in immune biology, GATA1 does have some relevance through its role in eosinophil and mast cell development. This loosely aligns with the immune biology criterion.<br>• Compared to the other genes, GATA1 rates more favorably across the key criteria of erythroid relevance, biomarker use, and transcriptional biomarker potential. Other genes like KEL are strong on certain criteria but weaker on others.<br>In summary, GATA1's extensive erythroid associations, current biomarker use, and promising transcriptional biomarker applications make it the top choice for inclusion in the targeted blood transcriptional profiling panel. Its partial immune relevance is a secondary benefit.	widely employed, GATA1 is clinically tested for diagnosing specific congenital dyserythropoietic anemias and related conditions.<br>3. **Potential use as a blood transcriptional biomarker**: GATA1 may hold potential as a blood transcriptional biomarker, primarily due to its integral role in erythropoiesis, for erythroid disorders, including congenital anemias.<br>4. **Relevance to leukocyte immune biology**: GATA1 has relevance to leukocyte immune biology due to its role in the development of certain white blood cells, such as eosinophils and mast cells.<br>5. **Status as a known drug target**: While not identified as a direct drug target currently, GATA1 is suggested as a potential drug target for blood-related disorders.<br>6. **Therapeutic relevance for immune-mediated diseases**: While not directly relevant as a therapeutic target for immune-mediated diseases, GATA1 might indirectly influence these conditions through the modulation of erythropoiesis.<br>Among the other candidates, GATA1 stands out in terms of its substantial relevance to erythroid cells and erythropoiesis, its potential use as a transcriptional biomarker, and its relevance to leukocyte immune biology. The other genes, including TGM2, KEL, HMBS, and HEMGN, each have strengths in specific areas, but GATA1 seems to present a well-rounded profile that covers all the criteria requested. Therefore,

		GATA1 would be the recommended candidate gene to include in a targeted blood transcriptional profiling panel.
PROMP 2: Now that you have provided an initial recommendation based on the summary, take into account the following RNA-sequencing expression data from key immune cell types and whole blood samples: *[input textualized transcriptional data].* Given this additional RNA-seq expression data, does your recommended gene selection remain the same? Please explain which gene you would recommend at this stage and why, incorporating both the summarized information *provided earlier and RNA-seq data in your rationale."*	After reviewing the additional RNA-sequencing expression data, I would modify my recommendation from GATA1 to HMBS as the top candidate gene to include in the targeted blood transcriptional profiling panel.<br>The key reasons are:<br>• While GATA1 showed high expression in whole blood, its expression was undetected across all the key immune cell types like neutrophils, monocytes, B cells, T cells, and NK cells. In contrast, HMBS showed expression across these cell types.<br>• HMBS meets the criteria of relevance to erythroid cells/erythropoiesis as it plays a role in heme synthesis, which is critical for erythropoiesis.<br>• It is used clinically as a biomarker for porphyrias, satisfying the biomarker criteria.<br>• The expression across immune cells and whole blood makes it a better candidate for a blood transcriptional biomarker compared to GATA1 and the other genes.<br>• Its role in heme synthesis provides some relevance to immune cell biology.<br>• It serves as a drug target for porphyria therapies.<br>In summary, while GATA1 is more specific to erythroid lineage, the lack of expression in key immune cells reduces its utility for this particular blood transcriptional profiling panel focused on immune response. HMBS has broader expression, retains erythroid relevance through heme synthesis, and meets more of the stated criteria, making it a better fit as the top candidate gene. The RNA-seq data provides key new insights that shift the recommendation from GATA1 to HMBS.	Based on the RNA-sequencing expression data provided, we can assess the expression of each gene across various immune cell types and whole blood samples. This data will influence our recommendation as it provides a quantitative measure of the gene's expression in relevant immune contexts.<br>Here's a quick breakdown of average expression across all cell types provided:<br>• **TGM2**: 4.57<br>• **GATA1**: 3<br>• **KEL**: 0.86<br>• **HMBS**: 7.14<br>• **HEMGN**: 2.57<br>Firstly, let's review our previous analysis:<br>1. **GATA1**: This gene had a significant association with erythroid cells and erythropoiesis, some clinical utility, potential as a transcriptional biomarker, relevance to leukocyte immune biology (though limited), and some therapeutic relevance for immune-mediated diseases.<br>However, the RNA-seq data indicates that GATA1 has a high expression in whole blood but negligible to no expression in individual immune cell types. This could suggest that GATA1's high expression in whole blood is primarily due to its expression in erythroid cells (red blood cells and their precursors) rather than leukocytes.<br>2. **TGM2**: The RNA-seq data indicates moderate expression across various immune cells, suggesting its involvement in immune function. This complements our initial understanding of TGM2's diverse cellular roles,

		including in immune processes.<br>3. **HMBS**: This gene stands out with a higher average expression across immune cell types compared to others. This is in line with its involvement in the heme synthesis pathway, but its direct relevance to immune function was previously less established.<br>Considering the RNA-seq data and our initial summary:<br>1. **Relevance to erythroid cells and erythropoiesis**: GATA1 still stands out.<br>2. **Current and potential use as a biomarker**: GATA1 and HMBS have the most evidence.<br>3. **Relevance to leukocyte immune biology**: TGM2 and HMBS show broader expression across immune cell types.<br>4. **Status as a known drug target**: TGM2 has the most evidence.<br>5. **Therapeutic relevance**: TGM2 and GATA1, with HMBS having some indirect relevance.<br>Given the RNA-seq data and the other criteria, **HMBS** emerges as a stronger candidate due to its more consistent expression across immune cell types and whole blood, in addition to its known clinical utility and potential as a transcriptional biomarker. It also has some relevance in immune biology and therapeutic contexts, even if indirect.<br>While GATA1 remains significant in the context of erythroid biology and potential as a biomarker, its lack of expression in specific immune cell types somewhat diminishes its candidacy as a holistic representative for a blood transcriptional profile that encompasses both erythroid and immune signatures.

		Therefore, **HMBS** would be our revised recommendation given its broader expression in immune cells, its known clinical utility, and its potential as a transcriptional biomarker.
PROMPT 3: Now that you have provided an initial recommendation based on the summary and the RNAseq data, take into account the following microarray expression data from key immune cell types and hematopoietic precursors: *[input textualized transcriptional data].* Given this additional microarray expression data, does your recommended gene selection remain the same? Please explain which gene you would recommend at this stage and why, incorporating the summarized information and RNA-seq data provided earlier, as well as this new microarray data in your rationale.	After reviewing the microarray expression data, I would maintain my recommendation of HMBS as the top candidate gene for inclusion in the targeted blood transcriptional profiling panel.<br>The key reasons are:<br>• HMBS showed strong expression in all the erythroid precursor stages, aligning with its erythroid relevance through the heme synthesis pathway.<br>• It exhibited expression across the immune cell types - basophils, B cells, T cells, monocytes, dendritic cells, neutrophils etc. This breadth strengthens its case as a blood transcriptional biomarker.<br>• The high expression in erythroid precursors and more moderate expression in immune cells fits well with the goals of capturing both erythroid and immune biology.<br>• Other genes like GATA1 remain more restricted to just the erythroid lineage based on the microarray data.<br>• TGM2 and KEL show some limitations - TGM2 lacks erythroid specificity while KEL lacks immune cell expression.<br>• HEMGN did not show detectable expression in the microarray data.<br>In summary, the addition of the microarray expression data provides further support for HMBS as the top choice. Its expression profile aligns well with the criteria of spanning both erythroid and immune cell relevance. The other genes do not match HMBS in striking this important balance.<br>Therefore, I continue recommending HMBS as the top gene for inclusion after	*[N/A: input exceed GPT-4 limits. Attempts to break down inputting the textualized data were unsuccessful as GPT-4 lost the thread of the conversation. Thus, we chose to skip to the next prompt]*

	incorporating the initial summary, RNA-seq data, and new microarray expression information.	
PROMPT 4: Now that you have provided an initial recommendation based on the summary and the immune cells RNAseq and microarray data, take into account the following averaged log2 fold changes in RNA abundance in patient cohorts compared to controls. *[input textualized transcriptional data]*. Given this additional data, does your recommended gene selection remain the same? Please explain which gene you would recommend at this stage and why, incorporating the summarized information, the leukocytes RNA-seq and microarray data provided earlier, as well as these new patient cohort profiles in your rationale.	After reviewing the RNA abundance log fold change data across various patient cohorts, I would maintain my recommendation of HMBS as the top candidate gene for inclusion in the targeted blood transcriptional profiling panel.<br>The key reasons are:<br>• HMBS exhibited the most consistent and moderate differential expression across the diverse disease states and cohorts analyzed. The log fold changes ranged from -0.4 to 0.83, indicating transcriptomic perturbations but not dramatic fluctuations.<br>• Other genes like GATA1 and KEL showed more variable and sometimes large fold changes (up to -1.15 for KEL), implying their expression is more affected in certain disease contexts.<br>• TGM2 and HEMGN had weaker and less consistent signals overall in the cohort data.<br>• The moderate but detectable changes for HMBS across myriad immune and inflammatory conditions make it well suited as a blood transcriptional biomarker reflecting both erythroid and immune activity.<br>• The cohort data reinforces prior evidence of HMBS expression in immune cells and its involvement in heme synthesis for erythropoiesis.<br>In summary, the cohort RNA abundance analysis provides additional supporting evidence that HMBS is responsive to immune	*[GPT-4 was able to interpret the input transcriptional data but lost the thread of the conversation].*<br><br>Here's a comprehensive analysis of the expression patterns for the genes TGM2, GATA1, KEL, HMBS, and HEMGN across various conditions:<br><br>**TGM2 (Transglutaminase 2)**<br>• Generally downregulated in most conditions (chronic obstructive pulmonary disease, acute influenza, HIV infection, juvenile dermatomyositis, Kawasaki disease, sepsis).<br>• Upregulated in metastatic melanoma, multiple sclerosis, active pulmonary tuberculosis, acute respiratory syncytial virus infection.<br>• Variations in expression in other conditions (e.g., pregnancy, liver transplant recipients).<br>**GATA1 (GATA Binding Protein 1)**<br>• Downregulated in acute influenza, HIV infection, juvenile dermatomyositis, Kawasaki disease, systemic onset juvenile idiopathic arthritis.<br>• Upregulated in metastatic melanoma, pregnancy, active pulmonary tuberculosis, liver transplant recipients.<br>• Minimal changes in other conditions like B-cell deficiency and multiple sclerosis.<br>**KEL (Kell Metallo-Endopeptidase)**<br>• Generally downregulated in COPD, acute influenza, HIV, juvenile dermatomyositis, sepsis.

	shifts in blood but is not radically altered, retaining medium-level expression. This profile suits the intended purpose of a broad blood transcriptional biomarker. The other genes show limitations in their cohort expression profiles. Therefore, I continue to recommend HMBS as the top choice after reviewing the full spectrum of data provided.	• Upregulated in Kawasaki disease, metastatic melanoma, active pulmonary tuberculosis, systemic lupus erythematosus, liver transplant recipients.<br>• Mixed or minimal changes in other conditions like pregnancy, acute respiratory syncytial virus infection.<br>**HMBS (Hydroxymethylbilane Synthase)**<br>• Downregulated in COPD, acute influenza, HIV infection, sepsis.<br>• Upregulated in metastatic melanoma, multiple sclerosis, pregnancy, active pulmonary tuberculosis, systemic lupus erythematosus, liver transplant recipients.<br>• Minimal changes in other conditions such as Kawasaki disease, systemic onset juvenile idiopathic arthritis.<br>**HEMGN (Hemogen)**<br>• Data only partially provided, but from what is available:<br>• Downregulated in chronic obstructive pulmonary disease, acute influenza, HIV infection, Kawasaki disease.<br>• Upregulated in metastatic melanoma.<br>• Mixed changes in juvenile dermatomyositis.<br>**Interpretation and Insights**<br>1. **TGM2** seems to be downregulated in several inflammatory and infectious conditions, possibly indicating its role in immune response regulation.<br>2. **GATA1** expression pattern shows complex responses to different diseases, suggesting a multifaceted role in

		immune regulation, possibly in hematopoietic differentiation or inflammation response.<br>3. **KEL** expression may be linked to specific viral infections, immune responses, and certain cancer types, reflecting its role in red blood cell physiology.<br>4. **HMBS** expression seems to be upregulated in several autoimmune diseases, suggesting a possible role in immune system modulation.<br>5. **HEMGN** data is limited but suggests a significant decrease in specific viral infections, indicating a potential role in antiviral response.<br>These observations are valuable for understanding the complex regulatory patterns of these genes in different diseases. However, this is a high-level analysis and should be complemented by further experimental validation, functional studies, pathway analyses, and integration with clinical data for a more comprehensive understanding.
PROMPT 5: Could you please summarize the key conclusions you have drawn from the conversation so far?	Here is a summary of the key conclusions I have drawn regarding the selection of a candidate gene for inclusion in a targeted blood transcriptional profiling panel:<br>Initial Summary:<br>• Based on the initial criteria, GATA1 was recommended due to its strong associations with erythroid cells/erythropoiesis, use as a clinical biomarker, and potential as a transcriptional biomarker.<br>Leukocyte RNA-seq Data:<br>• With RNA-seq data showing GATA1 expression restricted to whole blood only, the recommendation was	*[N/A: GPT-4 could not keep the thread of the conversation following the input of large amount of textualized transcriptional profiling data]*

	changed to HMBS based on its broader expression across immune cells while retaining erythroid relevance.<br>Leukocyte Microarray Data:<br>• The microarray data further reinforced HMBS as the top choice given its robust expression across erythroid and immune cells.<br>Patient Cohort RNA Abundance Data:<br>• Moderate fold changes in HMBS RNA abundance across diverse disease states and cohorts supported its suitability as a broad blood transcriptional biomarker.<br>In summary, the recommendation evolved from GATA1 to HMBS based on expression data revealing HMBS as a better candidate for reflecting both erythroid and immune biology per the aims of the panel. The cohort abundance data then provided confirming evidence for HMBS.	

8. References

1. Altman, M. C. et al. Development of a fixed module repertoire for the analysis and interpretation of blood transcriptome data. Nat Commun 12, 4385 (2021).

2. Rinchai, D. *et al.* BloodGen3Module: blood transcriptional module repertoire analysis and visualization using R. *Bioinformatics* **37**, 2382–2389 (2021).

3. Rinchai, D. *et al.* Definition of erythroid cell-positive blood transcriptome phenotypes associated with severe respiratory syncytial virus infection. *Clin Transl Med* **10**, e244 (2020).

4. Iismaa, S. E., Mearns, B. M., Lorand, L. & Graham, R. M. Transglutaminases and disease: lessons from genetically engineered mouse models and inherited disorders. *Physiol Rev* **89**, 991–1023 (2009).

5. Kárpáti, S. *et al.* Transglutaminases in autoimmune and inherited skin diseases: The phenomena of epitope spreading and functional compensation. *Exp Dermatol* **27**, 807–814 (2018).
6. Ferreira, R., Ohneda, K., Yamamoto, M. & Philipsen, S. GATA1 function, a paradigm for transcription factors in hematopoiesis. *Mol Cell Biol* **25**, 1215–1227 (2005).
7. Ying, Y. *et al.* A case of McLeod syndrome caused by a nonsense variation c.942G>A in the XK gene: A case report. *Front Genet* **14**, 1073139 (2023).
8. Redman, C. M., Russo, D. & Lee, S. Kell, Kx and the McLeod syndrome. *Baillieres Best Pract Res Clin Haematol* **12**, 621–635 (1999).
9. Whatley, S. D. *et al.* Diagnostic strategies for autosomal dominant acute porphyrias: retrospective analysis of 467 unrelated patients referred for mutational analysis of the HMBS, CPOX, or PPOX gene. *Clin Chem* **55**, 1406–1414 (2009).
10. Jiang, J. *et al.* Hemgn is a direct transcriptional target of HOXB4 and induces expansion of murine myeloid progenitor cells. *Blood* **116**, 711–719 (2010).
11. Weiss, M. J. & Orkin, S. H. Transcription factor GATA-1 permits survival and maturation of erythroid precursors by preventing apoptosis. *Proc Natl Acad Sci U S A* **92**, 9623–9627 (1995).
12. Wagner, T., Lanzer, G. & Geissler, K. Kell expression on myeloid progenitor cells. *Leuk Lymphoma* **43**, 479–485 (2002).
13. Dailey, H. A. & Meissner, P. N. Erythroid heme biosynthesis and its disorders. *Cold Spring Harb Perspect Med* **3**, a011676 (2013).
14. Zheng, W.-W. *et al.* EDAG positively regulates erythroid differentiation and modifies GATA1 acetylation through recruiting p300. *Stem Cells* **32**, 2278–2289 (2014).
15. Zhao, K. *et al.* Hemgn Protects Hematopoietic Stem and Progenitor Cells Against Transplantation Stress Through Negatively Regulating IFN-γ Signaling. *Adv Sci (Weinh)* **9**, e2103838 (2022).
16. Dieterich, W. *et al.* Identification of tissue transglutaminase as the autoantigen of celiac disease. *Nat Med* **3**, 797–801 (1997).
17. Nichols, K. E. *et al.* Familial dyserythropoietic anaemia and thrombocytopenia due to an inherited mutation in GATA1. *Nat Genet* **24**, 266–270 (2000).

18. Gundemir, S., Colak, G., Tucholski, J. & Johnson, G. V. W. Transglutaminase 2: a molecular Swiss army knife. *Biochim Biophys Acta* **1823**, 406–419 (2012).
19. Orkin, S. H. & Zon, L. I. Hematopoiesis: an evolving paradigm for stem cell biology. *Cell* **132**, 631–644 (2008).
20. Yu, L. C., Twu, Y. C., Chang, C. Y. & Lin, M. Molecular basis of the Kell-null phenotype: a mutation at the splice site of human KEL gene abolishes the expression of Kell blood group antigens. *J Biol Chem* **276**, 10247–10252 (2001).
21. Kausar, T. *et al. Kell Blood Group System: A Systematic Review and Meta-Analysis*. https://www.researchsquare.com/article/rs-1904178/v1 (2022) doi:10.21203/rs.3.rs-1904178/v1.
22. Handschin, C. *et al.* Nutritional regulation of hepatic heme biosynthesis and porphyria through PGC-1alpha. *Cell* **122**, 505–515 (2005).
23. Eckert, R. L. *et al.* Transglutaminase regulation of cell function. *Physiol Rev* **94**, 383–417 (2014).
24. Shimizu, R., Engel, J. D. & Yamamoto, M. GATA1-related leukaemias. *Nat Rev Cancer* **8**, 279–287 (2008).
25. Cantor, A. B. & Orkin, S. H. Transcriptional regulation of erythropoiesis: an affair involving multiple partners. *Oncogene* **21**, 3368–3376 (2002).
26. Novershtern N, Subramanian A, Lawton LN, Mak RH, Haining WN, McConkey ME, et al. Densely interconnected transcriptional circuits control cell states in human hematopoiesis. Cell. 2011 Jan 21;144(2):296–309.
27. Linsley PS, Speake C, Whalen E, Chaussabel D. Copy number loss of the interferon gene cluster in melanomas is linked to reduced T cell infiltrate and poor patient prognosis. PloS One. 2014;9(10):e109760.
