# Supplementary material for: Harnessing large language models (LLMs) for candidate gene prioritization and selection
Source: J Transl Med. 2023 Oct 16;21:728. doi: 10.1186/s12967-023-04576-8 (PMC10580627; doi:10.1186/s12967-023-04576-8)
Supplement: Supplementary file 5 — Additional file 5: Is the prioritization report for module M11.4 authored by Éléonore Bettacchioli. The file is shared in the PDF format. [file 12967_2023_4576_MOESM5_ESM.pdf]

M11.4 Prioritization report

Prepared by: Eléonore Bettacchioli (CHU de Brest, Brest, France ; Université de Bretagne Occidentale, Brest, France)

1. Composition

The module M11.4 belongs to Aggregate A37 from the BloodGen3 fixed module repertoire [1,2]. This module aggregate is associated with circulating erythroid cells. It was found to be associated with RSV disease severity [3]. We showed in the same study that the abundance of A27 transcripts is also elevated in the blood of patients with stage IV melanoma [3].
It comprises 24 genes: AP2M1, AP2S1, ATG9A, B4GALT3, BCL2L13, C13ORF15, C19ORF62, CYB5R3, DAP, DENND1A, FAM134A, HK1, JUND, MSI2, NTAN1, NUCB1, PA2G4, POLR1D, TCEB2, UBAC1, UBAP1, UBL7, UROD, WDR13, WIPI2, ZMAT2.

2. Functional convergence

Functional associations were identified among the genes constituting M11.4 using GPT-4 (see Step 2 method for details).

Functional Themes	Associated Genes	Comments
Autophagy and Endocytosis	AP2M1, AP2S1, ATG9A, BCL2L13, FAM134A, WIPI2	AP2M1 and AP2S1 are part of the adaptor protein complex 2 (AP-2), which is involved in clathrin-mediated endocytosis. ATG9A plays a role in autophagy, a process that delivers cytoplasmic components to the lysosome for degradation. BCL2L13 is an autophagy related gene. FAM134A is a reticulophagy regulator, selectively removing portions of the endoplasmic reticulum via autophagy. WIPI2 also plays a role in autophagy.
RNA Processing	POLR1D, MSI2, DENND1A	POLR1D is involved in RNA Polymerase I and III, participating in transcription. MSI2 is an RNA binding protein involved in post-transcriptional gene regulation. DENND1A has been associated with clathrin-mediated endocytosis and is also a Rab guanine nucleotide exchange factor (RabGEF), possibly tying into processes of intracellular trafficking and RNA processing.
Ubiquitination	UBAC1, UBAP1	UBAC1 and UBAP1 are part of the ubiquitin pathway. Ubiquitination is a process that marks proteins for degradation and plays a significant role in a variety of cellular processes.
Energy Metabolism	HK1, CYB5R3	HK1 encodes hexokinase, which is the first enzyme in the glycolytic pathway. CYB5R3 is involved in electron transport and therefore energy production.

Other Cellular Processes	B4GALT3, DAP, JUND, NTAN1, NUCB1, PA2G4, TCEB2, UBL7, UROD, WDR13, ZMAT2	Several of these genes (e.g., B4GALT3, DAP, JUND, NTAN1, NUCB1, PA2G4, TCEB2, UBL7, UROD, WDR13, and ZMAT2) have diverse roles in other cellular processes such as signal transduction, cellular growth and differentiation, DNA repair, cell cycle control, and more.

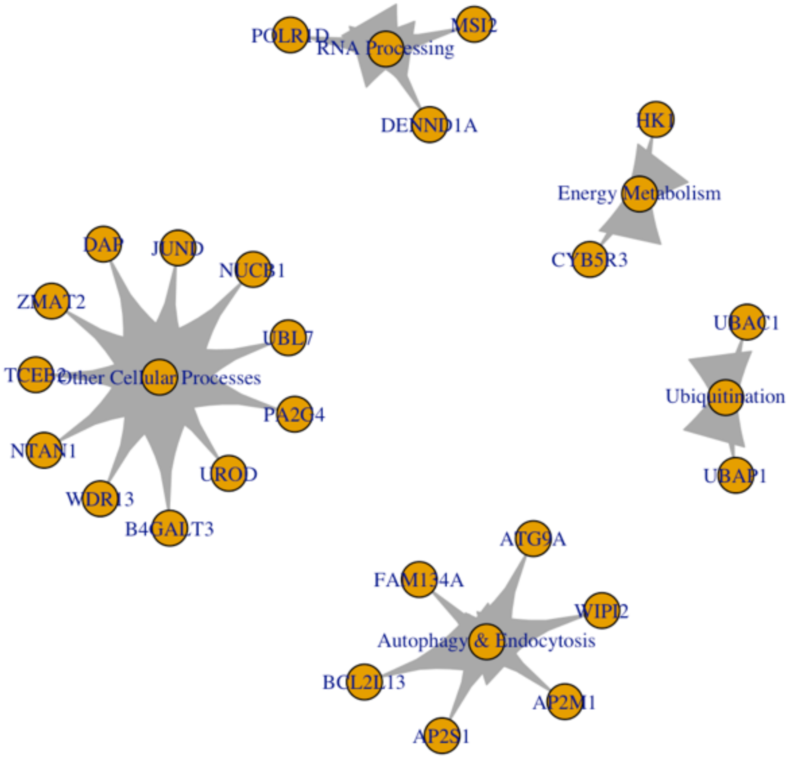

3. Scoring and prioritization

Genes were scored on six criteria using GPT-4 and Claude. The scores were averaged, and candidate genes were ranked according to their cumulative scores (**Figure 1**, Methods: Step 3 and Step 4).

The two LLMs were requested to score each gene on the following six statements:

a. The gene is associated with erythroid cells or erythropoiesis.

b. The gene is currently being used as a biomarker in clinical settings.

c. The gene has potential value as a blood transcriptional biomarker.

d. The gene is relevant to circulating leukocytes immune biology.

e. The gene is a known drug target.

f. The gene is therapeutically relevant for immune-mediated diseases.

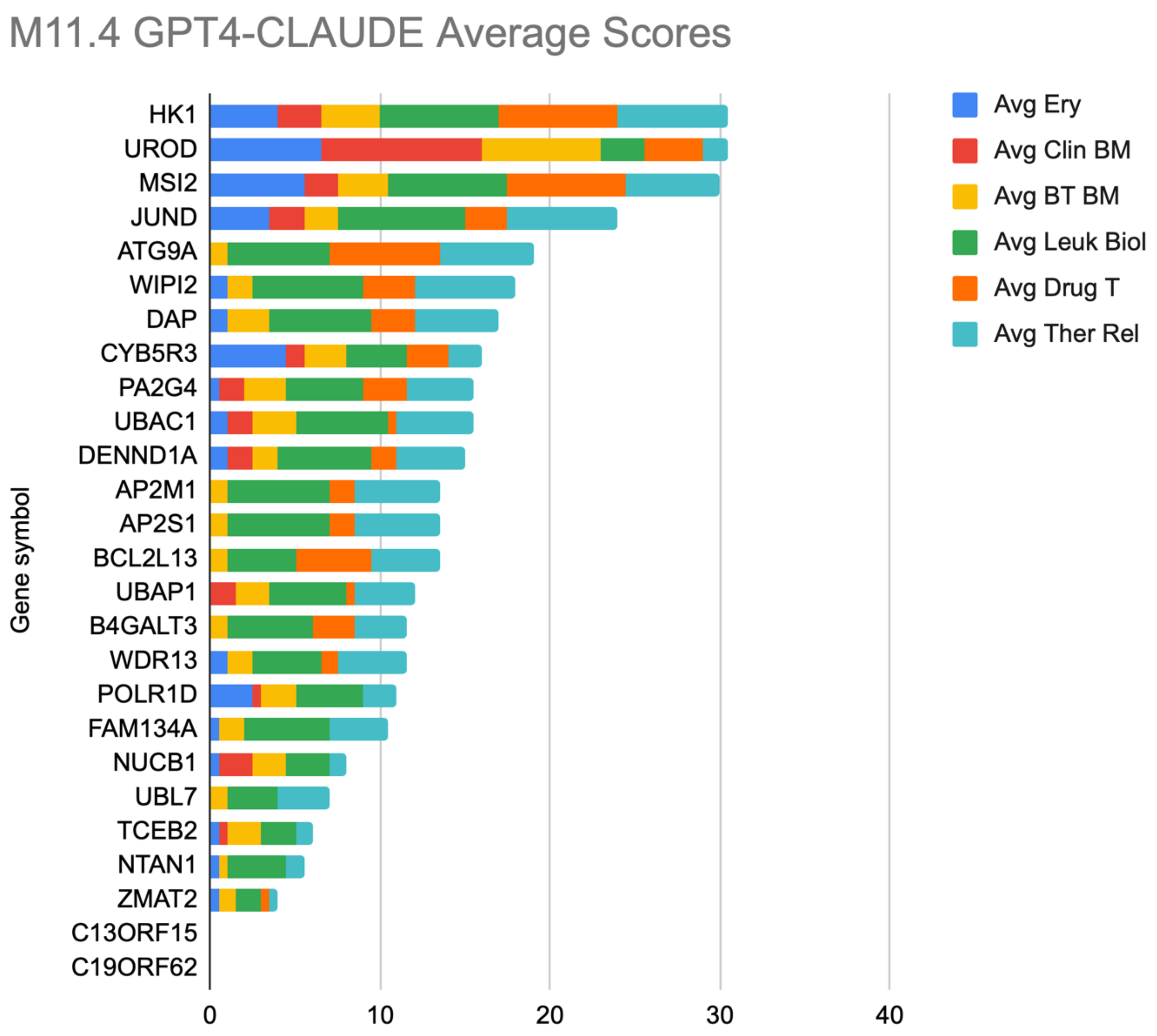

Figure 1: The stacked bar graph shows cumulative scores across six criteria for the 26 M11.4 genes. The top five genes selected for further evaluation were: HK1, UROD, MSI2, JUND, and ATG9A.

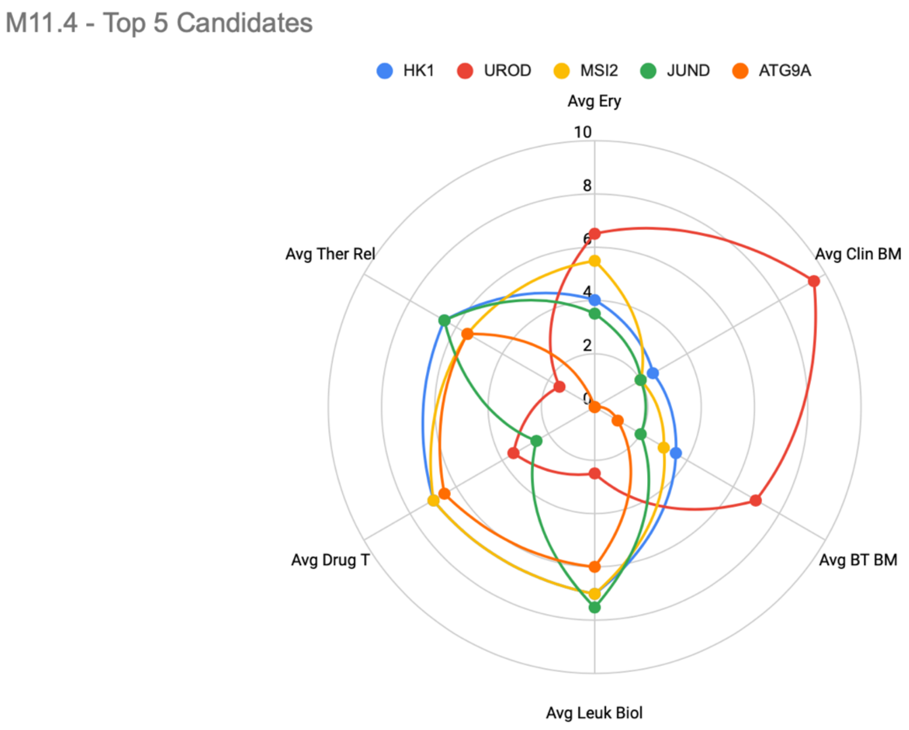

Figure 2: The radar plot represents individual scores for the 5 top scoring genes: HK1, UROD, MSI2, JUND, and ATG9A.

4. Knowledge-driven evaluation of top five candidates

Justifications given by the GPT-4 and Claude for the scores provided across the 6 criteria were compiled and summarized by GPT-4 (Methods: Step 5). For each statement pertinent references were retrieved using GPT-4 or Claude, with the relevance of each reference checked and attributed manually by the researcher authoring this report.

Function: The ATG9A gene encodes a protein pivotal for autophagy, a cellular self-degradation and recycling mechanism. This protein is believed to act as a membrane transporter, facilitating the transition between the Golgi network, plasma membrane, and autophagosomes, being essential for autophagosome formation [4]. On the other hand, HK1 is responsible for producing a homodimeric enzyme central to glycolysis, catalyzing the phosphorylation of glucose to yield glucose-6-phosphate, playing an indispensable role in glucose metabolism across most mammalian tissues [5]. JUND, a member of the Jun family, encodes a transcription factor which binds to AP-1 promoter elements. It modulates gene expression in reaction to various stimuli, overseeing cellular processes like proliferation, differentiation, apoptosis, and even tumorigenesis, with a noted role in hematopoiesis and immune responses [6]. MSI2, meanwhile, produces an RNA-binding protein that governs the translation and stability of certain mRNAs, influencing stem cell self-renewal, cell fate, tumorigenesis, and playing a potential role in

hematopoiesis and some leukemias [7]. Finally, the UROD gene synthesizes an enzyme vital to heme biosynthesis and porphyrin metabolism. This enzyme mediates the conversion of uroporphyrinogen to coproporphyrinogen, and its mutations are linked to forms of porphyria, marked by photosensitivity and liver disorders [8].

a. Relevance to erythroid cells and erythropoiesis: There is no direct evidence associating ATG9A with erythroid cells or erythropoiesis [4]. While HK1 plays a fundamental role in glucose metabolism essential for all cell types, there's no specific evidence linking it directly to erythroid cells or erythropoiesis [5]. JUND's association with erythroid cells or erythropoiesis is limited and indirect, although it may influence hematopoietic stem cell function [6]. MSI2 has a notable association with erythroid cells and erythropoiesis, primarily due to its involvement in hematopoiesis and stem cell maintenance [7,9]. UROD is intrinsically linked with erythroid cells and erythropoiesis because of its crucial function in heme biosynthesis, a key process for hemoglobin production in erythroid cells [8].

b. Is used as a clinical biomarker: ATG9A is not recognized as a biomarker in standard clinical practice [4]. Similarly, HK1, while indicative of cellular metabolic state and linked to various diseases including cancer, is not presently employed as a clinical biomarker [5]. JUND, despite its altered expression in multiple cancers [10], is not widely adopted as a biomarker in clinical settings [6,11]. MSI2, while associated with overexpression in certain malignancies such as acute myeloid leukemia (AML), is not standardly utilized as a clinical biomarker [7]. Conversely, UROD mutations serve as established clinical biomarkers for diagnosing specific porphyrias, including porphyria cutanea tarda and congenital erythropoietic porphyria [8,12].

c. Potential relevance as a blood transcriptional biomarker: Currently, the evidence for ATG9A's utility as a blood transcriptional biomarker is scant [4]. While HK1's involvement in glucose metabolism positions it as a potential biomarker, especially in the context of diseases like cancer, definitive evidence remains limited [13]. JUND, a transcription factor, is not directly considered for blood transcriptional biomarking; however, its activities may influence the expression of other prospective biomarkers [6]. MSI2's role in hematopoiesis and certain cancers earmarks it as a potential blood transcriptional biomarker, awaiting further validation [9]. UROD, with mutations linked to porphyria cutanea tarda, has inherent value as a blood biomarker, particularly for specific anemias and porphyrias, but further research is paramount for a comprehensive understanding [8].

d. Relevance to leukocytes immune biology: ATG9A is expressed in various cell types, including circulating leukocytes, and plays a significant role in autophagy-dependent processes, such as pathogen clearance, antigen presentation, and cytokine production, although its direct influence on leukocyte biology is not clearly delineated [14,15]. HK1, being crucial for glucose metabolism, is pertinent to the immune function and activation of circulating leukocytes [16]. JUND is present in a majority of leukocytes, orchestrating immune responses, inflammation, and hematopoiesis [17]. MSI2, vital for hematopoiesis, is expressed in specific leukocytes, predominantly hematopoietic stem and progenitor cells, where it governs processes like self-renewal, differentiation, and tumorigenesis [7]. In contrast, UROD, predominantly engaged in the

heme biosynthesis pathway within erythroid and hepatic cells, has only a tangential connection to leukocyte biology and immune function, warranting further investigation.

e. Is a known drug target: ATG9A, owing to its involvement in autophagy [4] —a process linked with diseases like cancer and neurodegeneration—emerges as a promising drug target. There is potential in probing indirect ATG9A modulators to regulate autophagy in immune and inflammatory diseases [18]. ~~HK1, especially its inhibitors, has garnered interest as a therapeutic avenue, primarily for its anti-cancer potential, though no such inhibitors have gained clinical approval.~~ JUND, while not a conventional drug target due to its transcription factor role [17], presents opportunities for influencing disease pathways, especially in inflammation and immunity, though approved inhibitors are absent. MSI2, observed to be overexpressed in malignancies such as AML, offers potential as a therapeutic target, particularly for certain leukemias, but again lacks approved inhibitors [7]. ~~In contrast, UROD has been directly targeted in therapeutic strategies for porphyria cutanea tarda, and further exploration into UROD modulators could offer treatments for porphyrias or heme regulation, pending approval. (35327450).~~

f. Potential therapeutic relevance for immune-mediated diseases: ATG9A, implicated in autophagy, presents potential relevance in immune-mediated diseases, and while it may serve as a therapeutic target in specific cancers and immune conditions, comprehensive research is warranted [4]. HK1, pivotal for glucose metabolism, is potentially therapeutically significant, especially in metabolic diseases or cancers; however, its direct impact on immune-mediated diseases remains less substantiated [19,20]. ~~JUND's involvement in immune responses earmarks it as a possible therapeutic target for conditions like allergies, asthma, and rheumatoid arthritis.~~ MSI2, recognized for its influence on immune responses and hematopoiesis, may be pertinent as a therapeutic target in hematological malignancies, yet its role in immune-mediated diseases needs further elucidation [7]. In contrast, UROD's direct relevance to immune-mediated diseases appears minimal, with its primary function rooted in porphyrin and heme metabolism [21].

5. Pick(s) for M11.4 based on summarized justifications.

We aim to establish a universal immune profiling assay that measures transcript abundance of a panel of genes in whole blood using targeted molecular profiling technology (Immune profiling transcriptome fingerprinting assay). We first attempted to identify top candidates by prompting the GPT-4 and Claude using as input the summarized and fact check justifications obtained from the previous step.

Claude and GPT-4 were prompted to select a top candidate on the basis of the information summarized above in section 4, taking into account all six scoring criteria equally (Methods: Step 6). Both Claude and GPT-4 chose UROD and output provided are given below.

Output from Claude: "Based on the information provided, UROD would be my top candidate gene to include in a targeted assay aiming to measure differences in transcript abundance for a circulating erythroid cell signature.

The key reasons are:

- UROD is intrinsically linked with erythroid cells and erythropoiesis because of its crucial function in heme biosynthesis, which is a key process for hemoglobin production in erythroid cells. This makes it a highly relevant gene for erythroid biology.
- UROD mutations serve as established clinical biomarkers for diagnosing specific porphyrias, indicating its close association with erythropoiesis and utility as a biomarker.
- UROD has inherent value as a blood biomarker, particularly for anemias and porphyrias related to erythropoiesis, though further research would strengthen this.
- In contrast to the other genes, UROD does not have major direct roles in leukocyte biology and immune function, making it more specific to erythroid cells.
- While MSI2 is also linked to erythroid cells through its hematopoietic role, UROD is more directly involved in heme/hemoglobin synthesis that defines erythroid cell maturation and function.

In summary, UROD's integral role in heme biosynthesis, established links to porphyrias, and blood biomarker potential make it the strongest candidate among these options for inclusion in an erythroid cell transcriptional signature assay."

<u>Output from GPT-4</u>: "The goal is to identify the top candidate gene for a targeted assay aiming to measure differences in transcript abundance for a circulating erythroid cell signature.
Here's a summarized breakdown of the genes:

1. **ATG9A**: Essential for autophagy. Not directly linked to erythroid cells or erythropoiesis.
2. **HK1**: Central to glycolysis in most tissues. Not directly associated with erythroid cells or erythropoiesis.
3. **JUND**: Influences hematopoietic stem cell function but its association with erythroid cells is limited and indirect.
4. **MSI2**: Plays a potential role in hematopoiesis and some leukemias. Noted association with erythroid cells and erythropoiesis.
5. **UROD**: Key for heme biosynthesis and directly linked to erythroid cells and erythropoiesis.

Considering the given information:

- **ATG9A** is not directly linked to erythroid cells or erythropoiesis.
- **HK1**'s involvement is general, and it's not directly associated with erythroid cells.
- **JUND** has limited and indirect association with erythroid cells or erythropoiesis.
- **MSI2** has a notable association with erythroid cells and erythropoiesis due to its involvement in hematopoiesis.
- **UROD** is directly linked with erythroid cells and erythropoiesis due to its essential role in heme biosynthesis, which is crucial for hemoglobin production in erythroid cells.

Given the above information and considering the focus on circulating erythroid cell signatures, the top candidate gene to be included in the targeted assay would be **UROD**. This gene is directly and intrinsically linked with erythroid cells and erythropoiesis because of its central role in heme biosynthesis."

6. Examining expression patterns of top 5 candidates across leukocyte populations.

The LLMs did not incorporate reference transcriptional profiling data in their evaluation and prioritization of candidate genes. The expression profiles of the top 5 candidate genes were retrieved for three reference datasets. These encompassed diverse leukocyte populations and hematopoietic progenitors as well as the whole blood of patients with a wide range of conditions.

6.1 Densely interconnected transcriptional circuits control cell states in human hematopoiesis – (GSE24759) [22]
http://developmentalimmunology.gxbsidra.org/dm3/geneBrowser/show/4000026

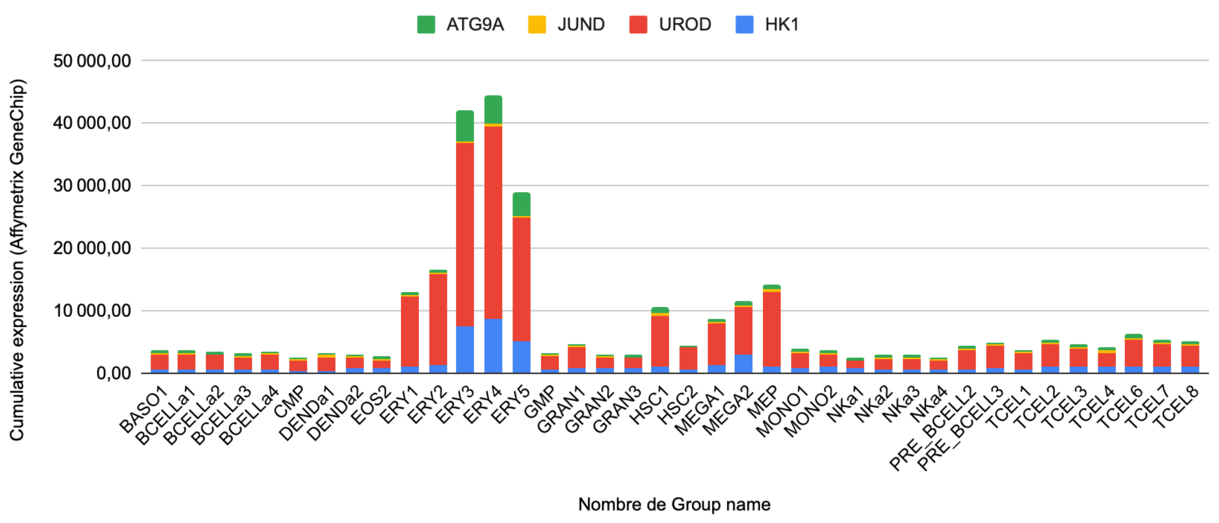

Figure 3: This stacked bar graph shows the expression levels of the five candidate genes.
ERY3-5 populations are CD71+ GLYA+ erythroid cells. The original article provides full experimental details: [22]. The web link above also provides access to more complete information about this dataset as well as individual gene profiles.

6.2 Next generation sequencing of human immune cell subsets across diseases – (GSE60424)
http://cd2k.gxbsidra.org/dm3/geneBrowser/show/4000098 [23]

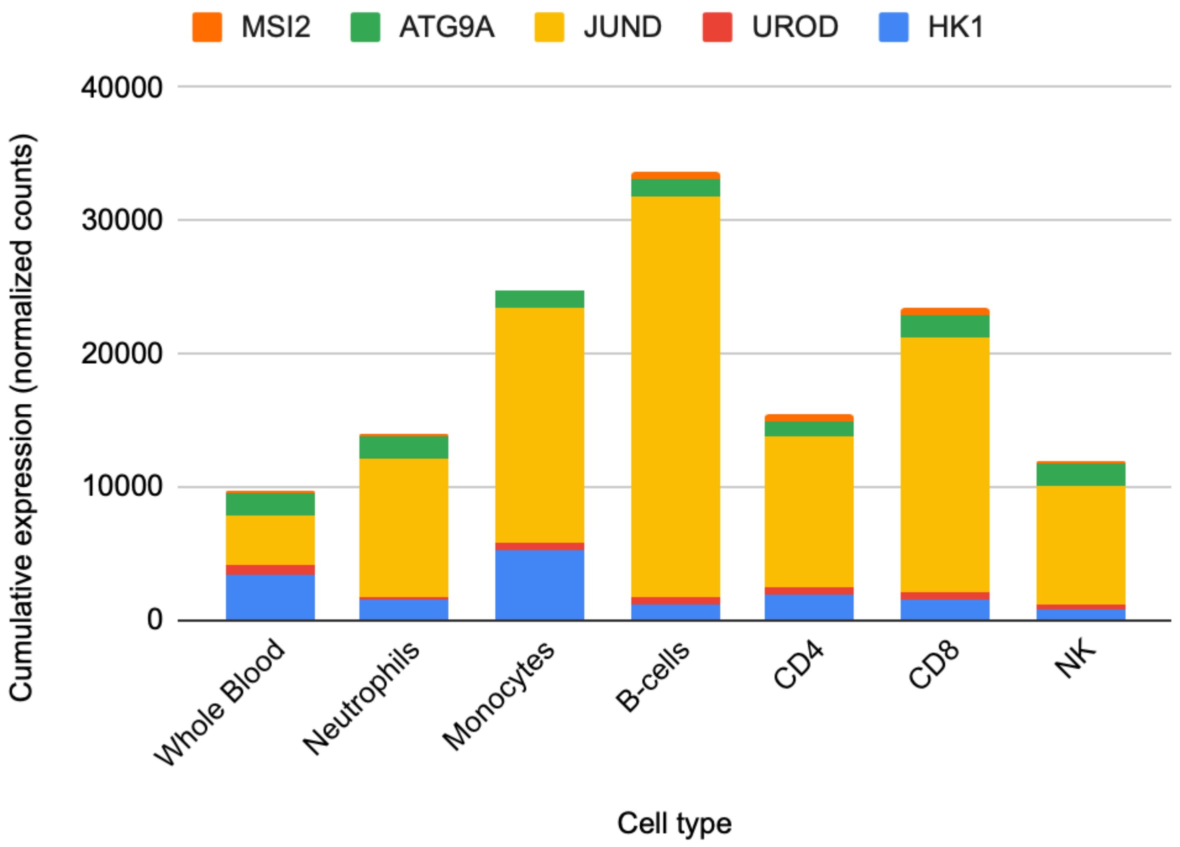

Figure 4: This stacked bar graph shows the expression levels measured by RNAseq of the five candidate genes across six leukocyte populations and whole blood. The abundance is shown for each gene in each population for an average of up to 20 samples. Additional experimental details are accessible via the link provided above and is available as part of this sample series GEO record (GSE60424) [23]

6.3 <u>BloodGen3 reference cohorts (GSE100150)</u>

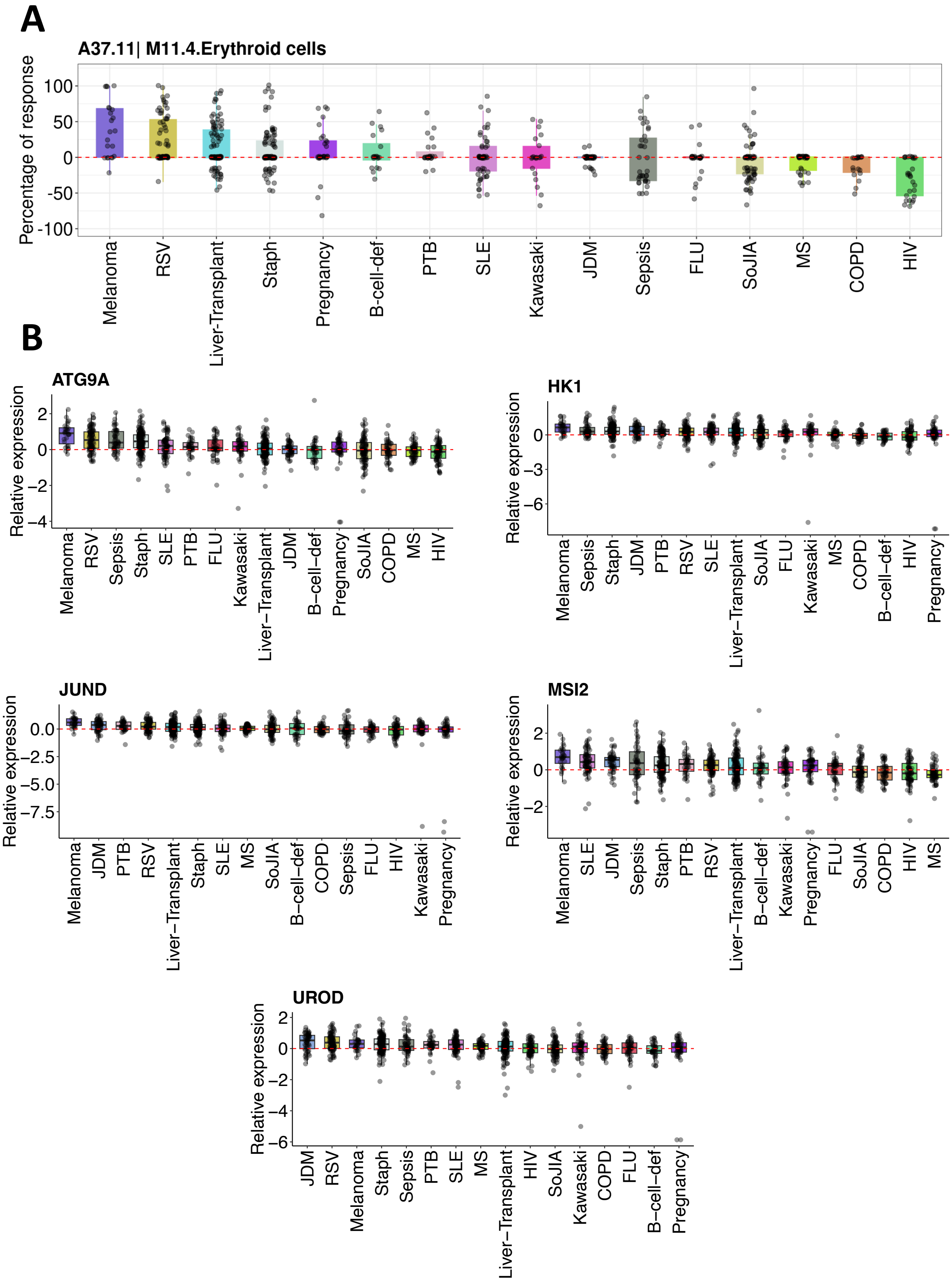
A
A37.11| M11.4.Erythroid cells
Percentage of response
100
50
0
-50
-100
Melanoma
RSV
Liver-Transplant
Staph
Pregnancy
B-cell-def
PTB
SLE
Kawasaki
JDM
Sepsis
FLU
SoJIA
MS
COPD
HIV
B
ATG9A
Relative expression
Melanoma
RSV
Sepsis
Staph
SLE
PTB
FLU
Kawasaki
Liver−Transplant
JDM
B−cell−def
Pregnancy
SoJIA
COPD
MS
HIV
HK1
Relative expression
Melanoma
Sepsis
Staph
JDM
PTB
RSV
SLE
Liver−Transplant
SoJIA
FLU
Kawasaki
MS
COPD
B−cell−def
HIV
Pregnancy
JUND
Relative expression
0.0
−2.5
−5.0
−7.5
Melanoma
JDM
PTB
RSV
Liver−Transplant
Staph
SLE
MS
SoJIA
B−cell−def
COPD
Sepsis
FLU
HIV
Kawasaki
Pregnancy
MSI2
Relative expression
Melanoma
SLE
JDM
Sepsis
Staph
PTB
RSV
Liver−Transplant
B−cell−def
Kawasaki
Pregnancy
FLU
SoJIA
COPD
HIV
MS
UROD
Relative expression
JDM
RSV
Melanoma
Staph
Sepsis
PTB
SLE
MS
Liver−Transplant
HIV
SoJIA
Kawasaki
COPD
FLU
B−cell−def
Pregnancy

Figure 5: The box plot in panel A shows the changes in abundance of transcripts comprising the M11.4 module, expressed within a cohort as the percentage of genes showing differential expression between each patient and the respective set of control subjects for this cohort. Differential expression is based on fixed cut-off (Log2 Fold Change >1.5, difference>10). These values are expressed as % module activity and can range from 100% (all genes are upregulated) to -100% (all genes are downregulated). The box plot in panel B show expression levels for the top 5 scoring M11.4 genes in patient cohorts relative to respective control subjects matched for demographic variables.

7. Overall pick for M11.4 based on summarized justifications and reference transcriptional datasets.

Through multi-tier prompting, GPT-4 and Claude were requested to select a top candidate, based on the fact-checked justification summaries generated earlier and taking into account the transcriptional profiles from the three reference datasets.

This was achieved using multiple prompts. This strategy was successful when using Claude but GPT-4 could not follow our directives from start to finish when presented with large amounts of textualized transcriptional data.

Among the top 5 genes (MSI2, UROD, JUND, HK1, and ATG9A), CLAUDE initially selected UROD, but switched to MSI2 from the third prompt onward. GPT-4 first chose MSI2 but shifted to JUND at the second prompt, likely due to MSI2's low RNA expression in leukocytes.

We choose, MSI2 as the top candidate for M11.4, for the following reasons:

- UROD is specialized in heme biosynthesis, making it of limited interest for broader applications.
- JUND, being a transcription factor, is susceptible to modulation by various drugs and clinical situations. This variability could make it challenging to interpret in patients with diverse backgrounds, although its significance in cellular biology still makes it a good option.
- HK1 is ubiquitously expressed and is frequently compared to HK2, which has been much more extensively studied for its importance in cancer cell metabolism—specifically, glycolysis in tumor cells appears to rely on HK2.
- ATG9A has been minimally researched in the literature and is notable for its role in autophagy. However, there isn't sufficient data to warrant its inclusion in a transcriptional panel.
- MSI2 plays a critical role in controlling hematopoiesis and tumorigenesis, particularly in blood cancers. Its significance has been thoroughly investigated across fundamental, transversal, and clinical settings.

Prompts	CLAUDE	GPT-4

PROMPT 1: Based on the summary below, please recommend the top candidate gene to include in a targeted blood transcriptional profiling panel. The intent is for the selected gene to be representative of an erythroid cell signature associated with response to mRNA vaccines, severity of RSV infection and that found to be expressed at high levels in patients with metastatic melanoma and in liver transplant recipients. Please weigh the following criteria equally in your recommendation: 1) Relevance to erythroid cells and erythropoiesis, 2) Current use as a biomarker, 3) Potential use as a blood transcriptional biomarker, 4) Relevance to leukocyte immune biology, 5) Status as a known drug target, 6) Therapeutic relevance for immune mediated diseases: *[input summarized and fact-checked justifications from Step 5]*	Based on the provided summary and criteria, I would recommend UROD as the top candidate gene to include in the targeted blood transcriptional profiling panel. The key reasons are:<br>• UROD has the strongest direct relevance to erythroid cells and erythropoiesis due to its crucial role in heme biosynthesis.<br>• UROD mutations already serve as established clinical biomarkers for porphyrias, indicating utility as a biomarker.<br>• UROD has inherent value as a blood biomarker for anemias and porphyrias, though more research would be beneficial.<br>• UROD has minimal direct relevance to leukocyte biology and immune function compared to the other genes.<br>• While not an established drug target itself, UROD has been explored for modulating heme pathways linked to porphyrias and anemias.<br>• UROD has low direct relevance as a drug target for immune diseases.<br>In summary, UROD's integral ties to erythropoiesis, utility as a clinical biomarker, and blood biomarker potential make it the top choice for meeting the stated criteria and intent for inclusion in the transcriptional profiling panel compared to the other genes. The key goal of representing an erythroid signature is best achieved by UROD based on the provided information.	Given the extensive summary provided on the genes ATG9A, HK1, JUND, MSI2, and UROD, let's assess each gene according to the six criteria:<br>1. **Relevance to erythroid cells and erythropoiesis**: MSI2 and UROD have the most direct links, with UROD intrinsically tied to heme biosynthesis, essential for hemoglobin production in erythroid cells, and MSI2 being pivotal for hematopoiesis.<br>2. **Current use as a biomarker**: UROD mutations are established biomarkers for diagnosing certain porphyrias, while the others are not standardly utilized in clinical settings.<br>3. **Potential use as a blood transcriptional biomarker**: UROD has potential here, particularly in the context of specific anemias and porphyrias, but MSI2 is also flagged as a potential biomarker given its role in hematopoiesis and links to certain cancers.<br>4. **Relevance to leukocyte immune biology**: JUND and MSI2 emerge as relevant here. JUND is involved in orchestrating immune responses and inflammation, whereas MSI2 is essential for hematopoiesis and expressed in specific leukocytes.<br>5. **Status as a known drug target**: MSI2 and ATG9A seem promising. MSI2, for its overexpression in cancers like AML, and ATG9A for its involvement in autophagy, a process linked with diseases like cancer.<br>6. **Therapeutic relevance for immune mediated diseases**: MSI2's influence on immune responses and hematopoiesis suggests potential relevance, whereas ATG9A's involvement in autophagy may offer some therapeutic relevance for specific cancers and immune conditions.<br>**Recommendation**: Given the provided criteria, **MSI2** appears to be the top candidate to include in the targeted blood transcriptional profiling panel. It's notably associated with erythroid cells and erythropoiesis due to its role in hematopoiesis, has potential as a blood transcriptional biomarker, is significant in

		leukocyte biology, and offers therapeutic relevance in hematological malignancies. UROD is a close contender, especially in terms of its erythroid relevance and its status as a clinical biomarker, but MSI2 has broader implications across multiple criteria.
PROMP 2: Now that you have provided an initial recommendation based on the summary, take into account the following RNA-sequencing expression data from key immune cell types and whole blood samples: *[input textualized transcriptional data].* Given this additional RNA-seq expression data, does your recommended gene selection remain the same? Please explain which gene you would recommend at this stage and why, incorporating both the summarized information *provided earlier and RNA-seq data in your rationale."*	After reviewing the additional RNA-seq expression data, I would still recommend UROD as the top candidate gene for inclusion in the targeted blood transcriptional profiling panel. The key reasons are:<br>• The original rationale and criteria for UROD being the best representation of an erythroid signature still stand based on the summary information provided earlier.<br>• While the RNA-seq data shows UROD expression is lower than some other genes like HK1 and JUND in certain leukocytes and whole blood, it is still substantially expressed across all the sampled immune cell types and blood.<br>• Crucially, UROD remains the most erythroid-specific gene based on the background summary. The other genes have more established roles in leukocyte biology and immune function.<br>• The goal is a representative erythroid signature, for which UROD is ideally suited. Higher expression in leukocytes for the other genes makes them less specific.<br>• The absolute expression level of UROD is sufficiently high in all immune cell types to still be reliably measured and serve as an erythroid marker.	Given the new RNA-seq data, let's re-evaluate our recommendation based on the combined summary and the expression data. To assist the decision-making, we can summarize the RNA-seq data for each gene across the provided immune cell types and whole blood.<br>1. **HK1**<br>• Whole blood: 172,023<br>• Consistently high expression across multiple immune cell types with the highest in monocytes (2,581,615).<br>2. **UROD**<br>• Whole blood: 35,408<br>• Moderate expression across immune cells, the highest in monocytes (31,036) but significantly lower than HK1's expression.<br>3. **JUND**<br>• Whole blood: 183,356.5<br>• High expression across multiple immune cell types with the highest in monocytes (882,702).<br>4. **ATG9A**<br>• Whole blood: 82,870.5<br>• Moderate expression levels across immune cell types with the highest in neutrophils (84,985).<br>5. **MSI2**<br>• Whole blood: 9,100.5<br>• Generally lower expression across the board when compared to other genes. The highest expression is observed in CD8 T-cells (27,006).<br>From the RNA-seq data, **JUND** and **HK1** have the most prominent expression in whole blood and across different immune cell types. While MSI2 was the previous top candidate based on the summarized information, its expression level in whole blood and various immune cells is comparatively lower than the

	In summary, while the RNA-seq data provides useful additional expression context, UROD is still the top choice to fulfill the stated goal of reflecting an erythroid signature, based on its erythroid-specific function and reasonable expression levels across leukocytes and whole blood samples.	others, which could impact its utility as a blood transcriptional biomarker.<br>Considering the initial criteria:<br>1. **Relevance to erythroid cells and erythropoiesis**: MSI2 and UROD had the most relevance.<br>2. **Current use as a biomarker**: UROD was established, while the others, including JUND and HK1, were not standard.<br>3. **Potential use as a blood transcriptional biomarker**: MSI2 and UROD had potential, but JUND and HK1 had higher expression levels which could make them better biomarkers due to easier detectability.<br>4. **Relevance to leukocyte immune biology**: JUND stood out here.<br>5. **Status as a known drug target**: JUND and HK1 showed potential.<br>6. **Therapeutic relevance for immune mediated diseases**: JUND and MSI2 were notable.<br>**Recommendation**: Considering both the RNA-seq data and the initial criteria, **JUND** seems to be a strong candidate. It has a high expression in whole blood and across multiple immune cell types. Its relevance to leukocyte immune biology and potential as a drug target, combined with its high expression levels, make it a promising choice. Although MSI2 had a strong case based solely on the initial criteria, the expression data introduced a significant variable that cannot be overlooked, especially if the intention is to develop a robust blood transcriptional profile.<br>Thus, based on the combined information, I would recommend **JUND** as the top candidate gene.
PROMPT 3: Now that you have provided an initial recommendation based on the summary and the RNAseq data, take into account the following microarray expression data from key immune cell types and hematopoietic precursors: *[input textualized transcriptional data]*. Given this additional microarray expression data, does your recommended gene selection remain the same? Please	After reviewing the additional microarray expression data, I would modify my recommendation and suggest MSI2 as the top candidate gene for inclusion in the targeted transcriptional profiling panel. The key reasons are:<br>• The microarray data shows MSI2 expression is substantially higher in key erythroid precursor stages like CD34-CD71+ GlyA+ cells compared to the other	*[N/A: input exceed GPT-4 limits. Attempts to break down inputting the textualized data were unsuccessful as GPT-4 lost the thread of the conversation. Thus, we chose to skip to the next prompt]*

explain which gene you would recommend at this stage and why, incorporating the summarized information and RNA-seq data provided earlier, as well as this new microarray data in your rationale.	genes. This indicates it is more erythroid-specific.<br>• MSI2 also has strong expression in hematopoietic stem cells, aligning with its role in hematopoiesis and erythropoiesis based on the summary.<br>• While UROD is still linked to erythroid function, its expression does not stand out as selectively high in erythroid precursors based on the microarray data.<br>• MSI2 has more relevance to hematopoietic processes and progenitors than UROD based on the summary.<br>• MSI2's expression patterns make it a better representation of an erythroid signature across maturation stages compared to UROD.<br>• MSI2 remains a reasonable option for a biomarker and drug target based on the summary.<br>• While the RNA-seq shows higher UROD in whole blood, MSI2 is still sufficiently expressed.<br>In summary, the microarray data provides greater granularity on erythroid precursor stages, where MSI2 demonstrates more selective expression. Considering the full context, MSI2 is better positioned to serve as a representative erythroid signature compared to UROD or the other genes.	
PROMPT 4: Now that you have provided an initial recommendation based on the summary and the immune cells RNAseq and microarray data, take into account the	After reviewing the additional patient cohort data, I would maintain my recommendation of MSI2 as the top candidate gene for inclusion in the targeted transcriptional profiling panel.	*[GPT-4 was able to interpret the input transcriptional data but lost the thread of the conversation].*

following averaged log2 fold changes in RNA abundance in patient cohorts compared to controls. *[input textualized transcriptional data].* Given this additional data, does your recommended gene selection remain the same? Please explain which gene you would recommend at this stage and why, incorporating the summarized information, the leukocytes RNA-seq and microarray data provided earlier, as well as these new patient cohort profiles in your rationale.	The key reasons are:<br>MSI2 shows relevant and consistent changes across many of the patient cohorts of interest, including viral infections like influenza and RSV, autoimmune diseases like juvenile arthritis, and cancer like melanoma.<br>The magnitude of MSI2 changes appears appreciable in cases like melanoma, SLE, and RSV. This suggests useful detection sensitivity.<br>MSI2 outperforms the other genes in cohorts like melanoma and RSV that were called out as being relevant in the initial criteria.<br>While some other genes like ATG9A change more in certain cohorts, MSI2 has a broader range of disease associations based on this data.<br>MSI2 has already been selected based on its erythroid specificity per the earlier RNA-seq and microarray data.<br>The patient cohort data further validates MSI2's utility as a biomarker across a range of physiological states.<br>This data adds confidence in detecting MSI2 expression differences in whole blood relevant to the desired erythroid signature.<br>In summary, the patient cohort profiles provide additional evidence supporting MSI2 as a sensitive and informative biomarker for an erythroid signature related to conditions of interest, reinforcing it as the top choice. The combined dataset establishes MSI2 as the best selection to meet the specified criteria.	Alright, you've provided an exhaustive list of data about log2 fold changes in RNA abundance across a range of health conditions for five genes (HK1, UROD, JUND, ATG9A, and MSI2). Let's summarize and interpret this data.<br>**Observations:**<br>1. **HK1**:<br>• Decreased expression in patients with B-cell deficiency, HIV infection, and multiple sclerosis.<br>• Increased expression in patients with juvenile dermatomyositis, metastatic melanoma, pregnant subjects, active pulmonary tuberculosis, acute respiratory syncytial virus infection, systemic lupus erythematosus, systemic onset juvenile idiopathic arthritis, and staphylococcus aureus infection.<br>• Relatively unchanged or negligible differences in some diseases.<br>2. **UROD**:<br>• Decreased expression in patients with B-cell deficiency, chronic obstructive pulmonary disease, acute influenza infection, Kawasaki disease, multiple sclerosis, and systemic onset juvenile idiopathic arthritis.<br>• Increased expression in patients with juvenile dermatomyositis, metastatic melanoma, sepsis, pregnant subjects, active pulmonary tuberculosis, acute respiratory syncytial virus infection, systemic lupus erythematosus, and staphylococcus aureus infection.<br>3. **JUND**:<br>• Decreased expression in patients with acute influenza infection, HIV infection, Kawasaki disease, multiple sclerosis,

		and acute respiratory syncytial virus infection.<br>• Increased expression in patients with juvenile dermatomyositis, metastatic melanoma, sepsis, pregnant subjects, active pulmonary tuberculosis, systemic lupus erythematosus, systemic onset juvenile idiopathic arthritis, and staphylococcus aureus infection.<br>4. **ATG9A**:<br>• Decreased expression in patients with B-cell deficiency, chronic obstructive pulmonary disease, HIV infection, sepsis, multiple sclerosis, and systemic onset juvenile idiopathic arthritis.<br>• Increased expression in patients with acute influenza infection, metastatic melanoma, pregnant subjects, active pulmonary tuberculosis, acute respiratory syncytial virus infection, systemic lupus erythematosus, and staphylococcus aureus infection.<br>5. **MSI2**:<br>• The data provided cuts off after HIV infection. However, from the given data, there's a decreased expression in HIV infected patients and an increased expression in patients with B-cell deficiency, chronic obstructive pulmonary disease, and acute influenza infection.<br>**Interpretation:**<br>• **HK1**: Given the variety of diseases where HK1 is upregulated, it seems like a non-specific response in several inflammatory and infectious conditions. The downregulation in B-cell deficiency and multiple sclerosis may indicate some specific immune response associations.

		• **UROD**: The upregulation in several infectious and inflammatory conditions suggests a potential role in host response. The consistent downregulation in autoimmune conditions like lupus, Kawasaki disease, and multiple sclerosis hints towards an association with autoimmunity.<br>• **JUND**: The upregulation in malignancies like melanoma and infections such as tuberculosis indicates its potential role in both tumorigenesis and infection response. The downregulation in HIV and multiple sclerosis might show its importance in viral infections and neuroinflammatory processes.<br>• **ATG9A**: The significant upregulation in metastatic melanoma stands out. ATG9A may have a role in melanoma progression. Its role in infectious diseases is also evident, especially in respiratory infections like influenza and respiratory syncytial virus.<br>• **MSI2**: Data is limited, but there's a noticeable downregulation in HIV infection. This could indicate a potential role in viral host responses.<br>**Conclusions:**<br>1. There seems to be a differential regulation of these genes in infectious vs. autoimmune diseases, hinting towards their potential roles in specific pathways of the immune response.<br>2. Some genes like ATG9A show significant upregulation in specific conditions like metastatic melanoma, suggesting their potential role in disease progression or as potential therapeutic targets.<br>3. Further investigation into the function of these genes in specific diseases, combined with protein-level data and pathway analysis, would provide a clearer picture of their role and potential as therapeutic targets.
PROMPT 5: Could you please summarize the key conclusions you have drawn	Here are the key conclusions I have drawn so far:<br>• The genes ATG9A, HK1, JUND, MSI2, and	*[N/A: GPT-4 could not keep the thread of the conversation following the input of large*

from the conversation so far?	UROD were evaluated as candidates for an erythroid signature blood transcriptional profiling panel.<br>• Based on background summaries, UROD initially stood out as the top choice due to its direct role in heme synthesis and links to erythropoiesis.<br>• However, examination of RNA-seq expression data in immune cells indicated UROD may not be erythroid-specific, though it had reasonable expression.<br>• Microarray data in hematopoietic progenitors shifted preference to MSI2, which showed selective high expression in erythroid precursors.<br>• MSI2 also had stronger relevance to hematopoiesis and biomarkers based on the background summary.<br>• Patient cohort data further validated MSI2's potential utility as a sensitive biomarker for physiological states of interest.<br>• Taken together, the combined dataset establishes MSI2 as the top candidate to best meet the criteria for an informative erythroid signature in blood transcriptional profiling.<br>In summary, the recommendation is to select MSI2 for inclusion in the targeted panel based on its expression specificity to erythroid lineages, biomarker potential, and detection sensitivity for conditions like viral infection, autoimmunity, and cancer. The full context supports MSI2 as the optimal choice.	*amount of textualized transcriptional profiling data]*

8. References

[1] Altman MC, Rinchai D, Baldwin N, Toufiq M, Whalen E, Garand M, et al. Development of a fixed module repertoire for the analysis and interpretation of blood transcriptome data. Nat Commun 2021;12:4385. https://doi.org/10.1038/s41467-021-24584-w.

[2] Rinchai D, Roelands J, Toufiq M, Hendrickx W, Altman MC, Bedognetti D, et al. BloodGen3Module: Blood transcriptional module repertoire analysis and visualization using R. Bioinforma Oxf Engl 2021:btab121. https://doi.org/10.1093/bioinformatics/btab121.

[3] Rinchai D, Altman MC, Konza O, Hässler S, Martina F, Toufiq M, et al. Definition of erythroid cell-positive blood transcriptome phenotypes associated with severe respiratory syncytial virus infection. Clin Transl Med 2020;10:e244. https://doi.org/10.1002/ctm2.244.

[4] Orsi A, Razi M, Dooley HC, Robinson D, Weston AE, Collinson LM, et al. Dynamic and transient interactions of Atg9 with autophagosomes, but not membrane integration, are required for autophagy. Mol Biol Cell 2012;23:1860–73. https://doi.org/10.1091/mbc.E11-09-0746.

[5] Wilson JE. Isozymes of mammalian hexokinase: structure, subcellular localization and metabolic function. J Exp Biol 2003;206:2049–57. https://doi.org/10.1242/jeb.00241.

[6] Shaulian E, Karin M. AP-1 as a regulator of cell life and death. Nat Cell Biol 2002;4:E131-136. https://doi.org/10.1038/ncb0502-e131.

[7] Kharas MG, Lengner CJ, Al-Shahrour F, Bullinger L, Ball B, Zaidi S, et al. Musashi-2 regulates normal hematopoiesis and promotes aggressive myeloid leukemia. Nat Med 2010;16:903–8. https://doi.org/10.1038/nm.2187.

[8] Phillips JD, Bergonia HA, Reilly CA, Franklin MR, Kushner JP. A porphomethene inhibitor of uroporphyrinogen decarboxylase causes porphyria cutanea tarda. Proc Natl Acad Sci U S A 2007;104:5079–84. https://doi.org/10.1073/pnas.0700547104.

[9] Park S-M, Deering RP, Lu Y, Tivnan P, Lianoglou S, Al-Shahrour F, et al. Musashi-2 controls cell fate, lineage bias, and TGF-β signaling in HSCs. J Exp Med 2014;211:71–87. https://doi.org/10.1084/jem.20130736.

[10] Hernandez JM, Floyd DH, Weilbaecher KN, Green PL, Boris-Lawrie K. Multiple facets of junD gene expression are atypical among AP-1 family members. Oncogene 2008;27:4757–67. https://doi.org/10.1038/onc.2008.120.

[11] Henry NL, Hayes DF. Cancer biomarkers. Mol Oncol 2012;6:140–6. https://doi.org/10.1016/j.molonc.2012.01.010.

[12] Anderson KE, Bloomer JR, Bonkovsky HL, Kushner JP, Pierach CA, Pimstone NR, et al. Recommendations for the diagnosis and treatment of the acute porphyrias. Ann Intern Med 2005;142:439–50. https://doi.org/10.7326/0003-4819-142-6-200503150-00010.

[13] Wolf A, Agnihotri S, Micallef J, Mukherjee J, Sabha N, Cairns R, et al. Hexokinase 2 is a key mediator of aerobic glycolysis and promotes tumor growth in human glioblastoma multiforme. J Exp Med 2011;208:313–26. https://doi.org/10.1084/jem.20101470.

[14] Young ARJ, Chan EYW, Hu XW, Köchl R, Crawshaw SG, High S, et al. Starvation and ULK1-dependent cycling of mammalian Atg9 between the TGN and endosomes. J Cell Sci 2006;119:3888–900. https://doi.org/10.1242/jcs.03172.

[15] Deretic V. Autophagy in immunity and cell-autonomous defense against intracellular microbes. Immunol Rev 2011;240:92–104. https://doi.org/10.1111/j.1600-065X.2010.00995.x.

[16] Palsson-McDermott EM, O'Neill LAJ. The Warburg effect then and now: from cancer to

inflammatory diseases. BioEssays News Rev Mol Cell Dev Biol 2013;35:965–73. https://doi.org/10.1002/bies.201300084.
[17] Hess J, Angel P, Schorpp-Kistner M. AP-1 subunits: quarrel and harmony among siblings. J Cell Sci 2004;117:5965–73. https://doi.org/10.1242/jcs.01589.
[18] Shoji-Kawata S, Sumpter R, Leveno M, Campbell GR, Zou Z, Kinch L, et al. Identification of a candidate therapeutic autophagy-inducing peptide. Nature 2013;494:201–6. https://doi.org/10.1038/nature11866.
[19] Robey RB, Hay N. Is Akt the “Warburg kinase”?-Akt-energy metabolism interactions and oncogenesis. Semin Cancer Biol 2009;19:25–31. https://doi.org/10.1016/j.semcancer.2008.11.010.
[20] Pelicano H, Martin DS, Xu R-H, Huang P. Glycolysis inhibition for anticancer treatment. Oncogene 2006;25:4633–46. https://doi.org/10.1038/sj.onc.1209597.
[21] Phillips JD. Heme biosynthesis and the porphyrias. Mol Genet Metab 2019;128:164–77. https://doi.org/10.1016/j.ymgme.2019.04.008.
[22] Novershtern N, Subramanian A, Lawton LN, Mak RH, Haining WN, McConkey ME, et al. Densely interconnected transcriptional circuits control cell states in human hematopoiesis. Cell 2011;144:296–309. https://doi.org/10.1016/j.cell.2011.01.004.
[23] Linsley PS, Speake C, Whalen E, Chaussabel D. Copy number loss of the interferon gene cluster in melanomas is linked to reduced T cell infiltrate and poor patient prognosis. PloS One 2014;9:e109760. https://doi.org/10.1371/journal.pone.0109760.
