# Supplementary material for: Harnessing large language models (LLMs) for candidate gene prioritization and selection
Source: J Transl Med. 2023 Oct 16;21:728. doi: 10.1186/s12967-023-04576-8 (PMC10580627; doi:10.1186/s12967-023-04576-8)
Supplement: Supplementary file 6 — Additional file 6: Is the prioritization report for module M12.11 authored by Olivia White and Marina Yurieva. The file is shared in the PDF format. [file 12967_2023_4576_MOESM6_ESM.pdf]

M12.11 Prioritization report

Prepared by: Olivia White, Marina Yurieva (The Jackson Laboratory for Genomic Medicine, Farmington, CT, USA)

1. Composition

The module M12.11 belongs to Aggregate A37 from the BloodGen3 fixed module repertoire (1,2). This module aggregate is associated with circulating erythroid cells. It was found to be associated with RSV disease severity (3). We showed in the same study that the abundance of A27 transcripts is also elevated in the blood of patients with stage IV melanoma (3).
It comprises 24 genes: ALDH5A1, BNIP3L, C20ORF108, DNAJA4, FAM104A, HBD, HS.105618, HS.291319, ISCA1L, LOC389293, MPP1, MXI1, PRDX2, RAB2B, RAD23A, RIOK3, RPIA, SIAH2, SLC1A5, TFDP1, TRAK2, TSTA3, UBE2O, YOD1

2. Functional convergence

Functional associations were identified among the genes constituting M12.11 using GPT-4 (see Step 2 method for details).

Functional Themes	Associated Genes	Comments
Protein homeostasis	DNAJA4, SIAH2, and UBE2O	are all involved in processes related to protein folding, degradation, or response to stress, which are crucial aspects of protein homeostasis.
Cellular stress responses and survival	BNIP3L and PRDX2	BNIP3L is involved in apoptosis and autophagy, two processes that can be activated in response to cellular stress. PRDX2 is an antioxidant enzyme that helps protect cells from damage caused by reactive oxygen species, which can be produced under conditions of stress.
Metabolic processes	ALDH5A1 and RAB2B	ALDH5A1 plays a role in GABA degradation, which is a crucial part of neurotransmitter metabolism. RAB2B is involved in vesicle trafficking, which is essential for the transport of various substances, including metabolic intermediates and signaling molecules, within cells.

3. Scoring and prioritization

Genes were scored on six criteria using GPT-4 and Claude. The scores were averaged, and candidate genes were ranked according to their cumulative scores (**Figure 1**, Methods: Step 3 and Step 4).

The two LLMs were requested to score each gene on the following six statements:

a. The gene is associated with erythroid cells or erythropoiesis.

b. The gene is currently being used as a biomarker in clinical settings.

c. The gene has potential value as a blood transcriptional biomarker.

d. The gene is relevant to circulating leukocytes immune biology.

e. The gene is a known drug target.

f. The gene is therapeutically relevant for immune-mediated diseases.

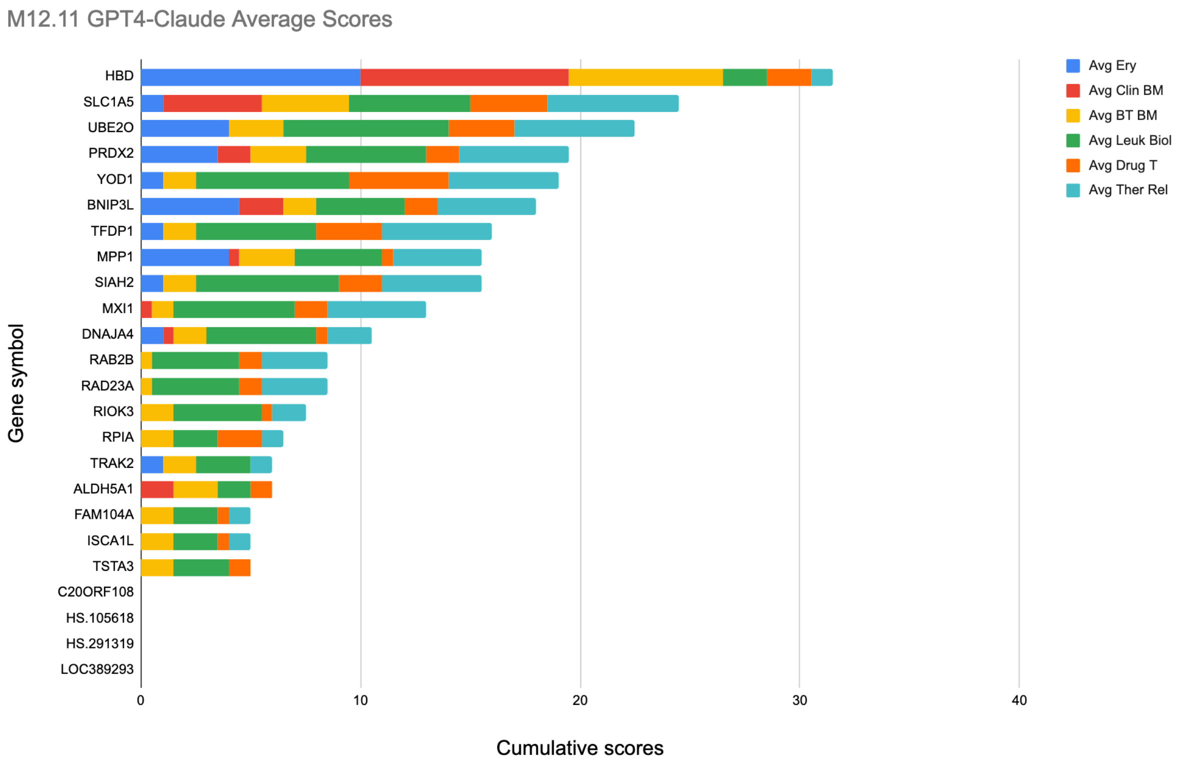

Figure 1: The stacked bar graph shows cumulative scores across six criteria for the 24 M12.11 genes. The top five genes selected for further evaluation were: HBD, SLC1A5, UBE2O, PRDX2 and YOD1.

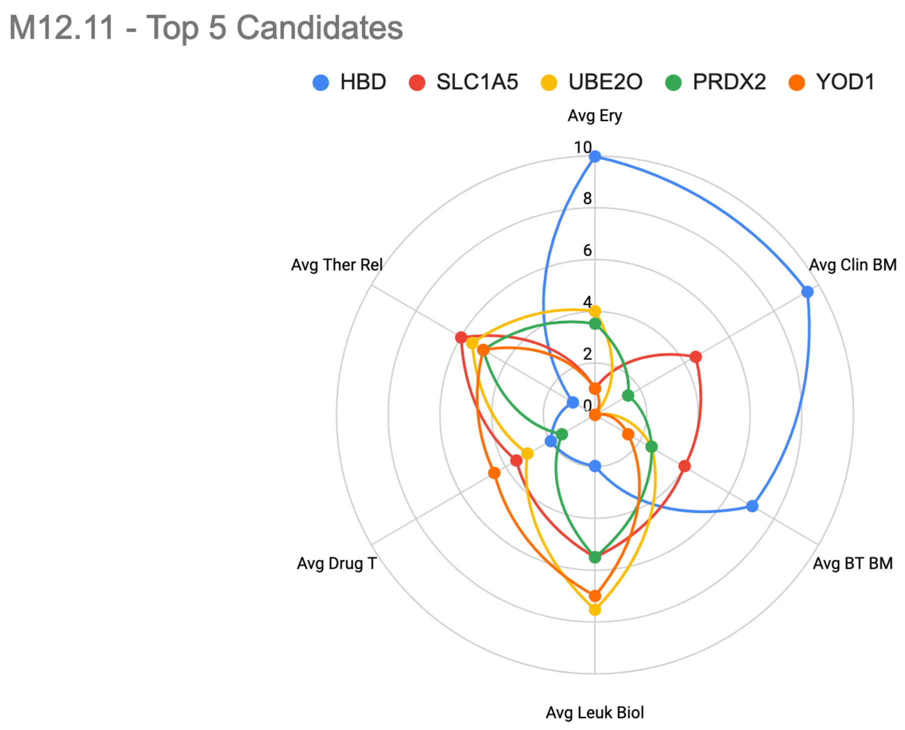

Figure 2: The radar plot represents individual scores for the 5 top scoring genes: HBD, SLC1A5, UBE2O, PRDX2 and YOD1.

4. Knowledge-driven evaluation of top five candidates

Function: "The HBD gene is responsible for encoding the delta subunit of hemoglobin. Post fetal development, its expression diminishes as the dominant form of hemoglobin becomes HbA (7). Mutations in HBD may lead to certain types of beta-thalassemia (8). The SLC1A5 gene codes for the ASCT2 protein, which mediates the cellular influx of neutral amino acids, significantly impacting cell growth and survival (9). The UBE2O gene codes for an E2 enzyme that functions in the ubiquitin-proteasome system, instrumental in protein degradation and involved in various biological processes including cellular differentiation and immune response (10). PRDX2 is an antioxidant enzyme, part of the peroxiredoxin family, playing a critical role in cellular redox regulation and defense against oxidative damage (11). Finally, the YOD1 gene encodes a deubiquitinating enzyme, crucial in the ubiquitin-proteasome system, controlling protein stability

and activity by removing ubiquitin molecules from target proteins, and contributing to protein quality control, cell cycle regulation, and immune response (12)."

a. Relevance to erythroid cells and erythropoiesis:

"Hemoglobin subunit delta (HBD) plays a critical role in erythroid cell function, constituting a significant component of the hemoglobin found within these cells (13). On the other hand, SLC1A5, though involved in general cellular growth and survival, shows no specific connection to erythroid cells or erythropoiesis (14), as does UBE2O, despite its recognized significance in erythroid cell development and maturation (10). Similarly, while PRDX2, found in erythrocytes, contributes to protecting these cells from oxidative damage, no direct link with erythroid cells or erythropoiesis has been established (15). Lastly, YOD1, despite limited evidence implying its involvement in erythropoiesis, lacks any direct association with erythroid cells or erythropoiesis (16)."

b. Is used as a clinical biomarker:

There is limited evidence of HBD or SLC1A5 being used as biomarkers in clinical settings. Likewise: "There is currently no clear evidence of UBE2O and YOD1 being utilized as clinical biomarkers in standard medical practice (18)(19). And as of the last update in September 2021, PRDX2 testing is not extensively used as a biomarker in routine clinical settings, despite some research suggesting potential applications (20)."

c. Potential relevance as a blood transcriptional biomarker:

"HBD, a crucial player in oxygen transport, shows promise as a blood transcriptional biomarker, especially in conditions affecting erythropoiesis and hemoglobin synthesis, such as beta-thalassemia or certain persistent hemoglobin variants. Similarly, SLC1A5, involved in cellular growth and survival, holds potential as a blood transcriptional biomarker for conditions related to glutamine metabolism. However, both HBD and SLC1A5 require further investigation to establish their reliability as biomarkers. On the other hand, UBE2O, implicated in hematopoiesis, and PRDX2, with its antioxidant defense role, may also serve as blood transcriptional biomarkers, but their utility remains uncertain, necessitating additional research. Likewise, YOD1, though offering limited evidence, warrants further investigation to determine its validity as a blood transcriptional biomarker."

d. Relevance to leukocytes immune biology:

“HBD primarily functions within erythrocytes, having little to no direct involvement in leukocyte biology or immune function, despite its potential to indirectly impact immune responses (21). SLC1A5, though not conclusively tied to leukocyte biology, may have significance due to its role in cellular survival and proliferation, regulating glutamine uptake and metabolism in various cell types (9). UBE2O and YOD1, both ubiquitously expressed, play crucial roles in immune responses by managing protein degradation and NF-kappaB signaling pathways respectively in immune and leukocyte cells (22)(23). Lastly, PRDX2, a component of the antioxidant system, may have indirect relevance to circulating leukocytes' biology (24). However, its specific role within these cells is not thoroughly researched as of the last update in 2021.”

e. Is a known drug target:

“The HBD gene encodes the delta subunit of fetal hemoglobin (HbF), a crucial component of the oxygen-carrying protein, hemoglobin, predominantly expressed during fetal development. In adults, the major hemoglobin form is HbA, with mutations in HBD leading to certain types of beta-thalassemia. The SLC1A5 gene encodes the ASCT2 protein, responsible for transporting neutral amino acids into cells, pivotal for cellular growth, survival, and proliferation. This transporter is essential for glutamine uptake, particularly in the kidneys, brain, and immune cells, impacting acid-base balance, neurotransmitter signaling, and immunity. Mutations in SLC1A5 are linked to glutamine deficiency disorders. The UBE2O gene produces an E2 enzyme, a part of the ubiquitin-proteasome system, which targets proteins for degradation. This enzyme plays a role in protein ubiquitination, influencing cellular processes such as growth, differentiation, apoptosis, and immune responses. PRDX2 is a member of the peroxiredoxin family, encoding an enzyme that neutralizes peroxides and safeguards cells from oxidative damage, thereby regulating cellular proliferation, differentiation, apoptosis, and other processes related to aging, immunity, and inflammation. Lastly, the YOD1 gene produces a deubiquitinating enzyme integral to the ubiquitin-proteasome system. This enzyme removes ubiquitin from proteins, impacting cell growth, differentiation, and proliferation. Its functions may extend to immunity, inflammation, and oncogenesis.”

f. Potential therapeutic relevance for immune-mediated diseases:

"The HBD gene, while influencing hemoglobin which can have indirect effects on the immune system, is not directly implicated in immune-mediated diseases, nor is it a potential therapeutic target for such conditions; its main functions pertain to erythropoiesis and hemoglobin structure (25). In contrast, SLC1A5, due to its role in cellular survival and proliferation, might have therapeutic relevance for immune-mediated diseases, though it also holds potential for metabolic or neurological conditions; however, conclusive evidence is lacking (9). UBE2O and YOD1, both implicated in immune modulation, are prospective therapeutic targets for immune-mediated diseases, possibly including cancers, but the depth of their potential roles remains under-researched (26)(19). Similarly, PRDX2, with its function in oxidative stress response, may hold potential as a therapeutic target in immune-mediated diseases or metabolic disorders, but its exact role is not yet extensively determined as of September 2021 (27)."

5. Examining expression patterns of top 5 candidates across leukocyte populations

The expression patterns of the top 5 candidate genes were examined across diverse leukocyte populations and hematopoietic precursors using two reference transcriptomic datasets.

5.1 Densely interconnected transcriptional circuits control cell states in human hematopoiesis – (GSE24759)
http://developmentalimmunology.gxbsidra.org/dm3/geneBrowser/show/4000026

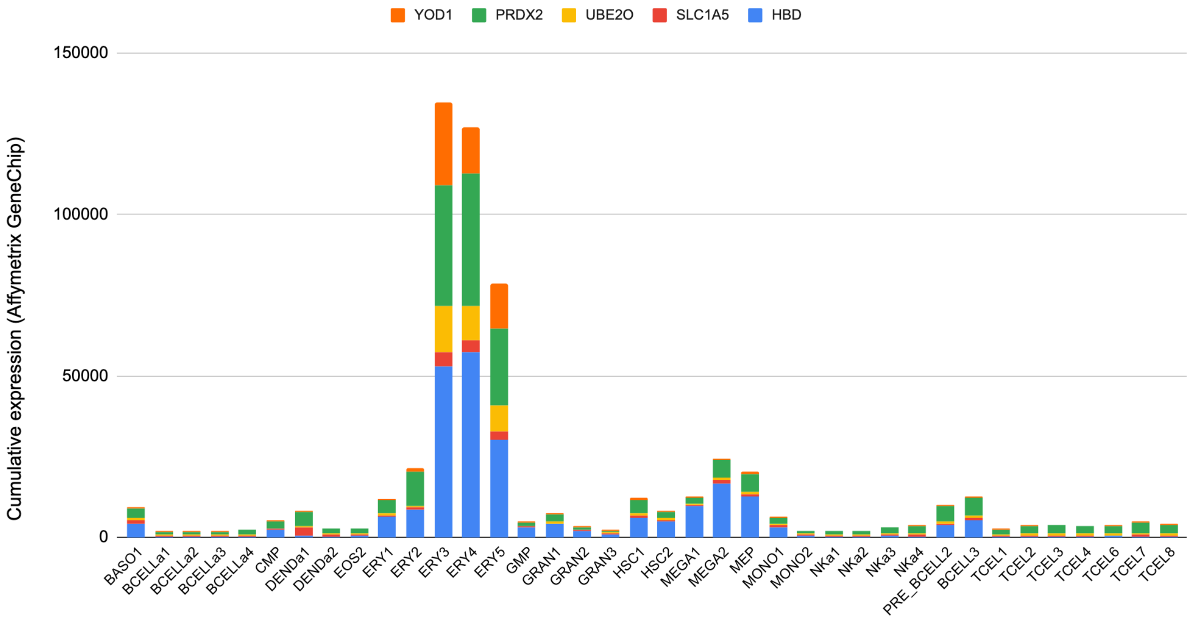

This stacked bar graph shows the expression levels of the five candidate genes. ERY3-5 populations are CD71+ GLYA+ erythroid cells. The original article provides full experimental details: (NN). The web link above also provides access to more complete information about this dataset as well as individual gene profiles.

5.2 <u>Next generation sequencing of human immune cell subsets across diseases – (GSE60424)</u>

http://cd2k.gxbsidra.org/dm3/geneBrowser/show/4000098

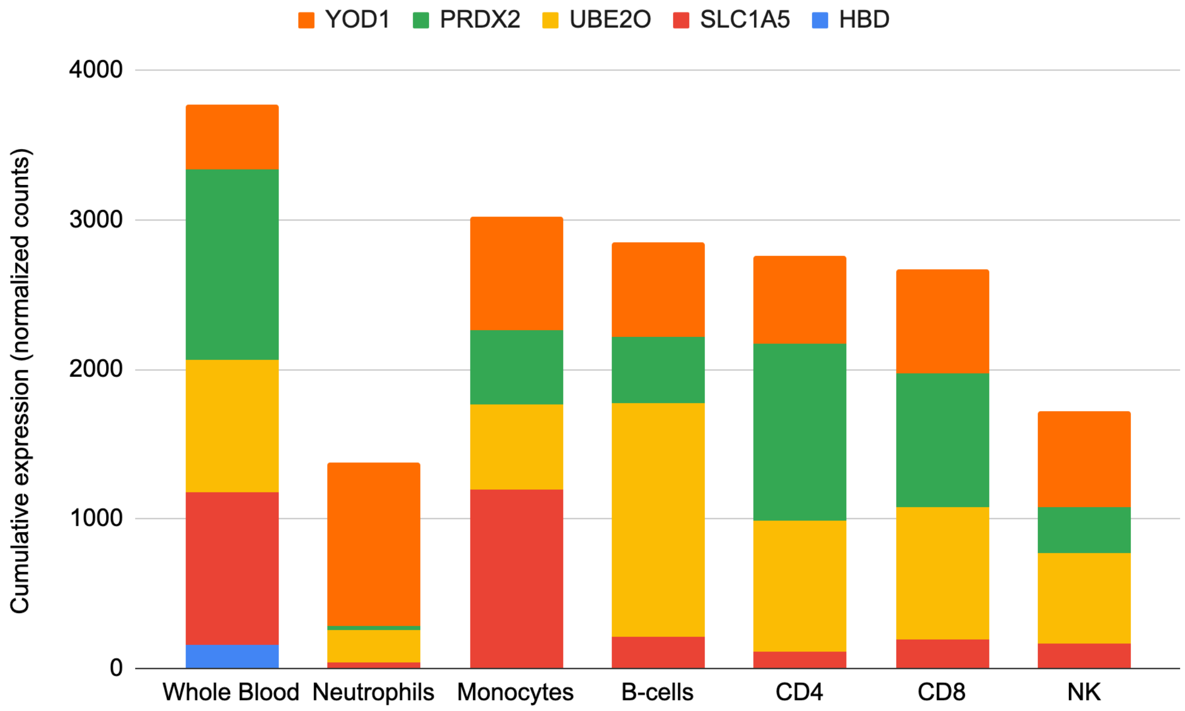

This stacked bar graph shows the expression levels measured by RNAseq of the five candidate genes across six leukocyte populations and whole blood. The abundance is shown for each gene in each population for an average of up to 20 samples. Additional experimental details are accessible via the link provided above and is available as part of this sample series GEO record (GSE60424) (4).

6. Overall pick(s) for M12.11

We aim to establish a universal immune profiling assay that measures transcript abundance of a panel of genes in whole blood using targeted molecular profiling technology (Immune profiling transcriptome fingerprinting assay). The top candidate identified here among the set of genes constituting M12.11 will be subjected to more in-depth evaluation using a pre-established workflow (5, 6).

Claude and GPT-4 were prompted to select a top candidate on the basis of the information summarized above in section 4, taking into account all six scoring criteria equally (Methods: Step 7).

Claude chose HBD: “Based on the additional information provided, I would suggest HBD as the top candidate gene for inclusion in a targeted assay aiming to measure differences in transcript abundance for a circulating erythroid cell signature.

The key reasons are:

- HBD plays a critical and direct role in erythroid cell function as a key component of hemoglobin, which is the most abundant protein in erythrocytes. Its expression is closely tied to erythropoiesis.
- It is highly specific to erythroid cells compared to the other genes. The other genes have more general cellular functions or lack direct ties to erythroid biology.
- Mutations in HBD lead to hemoglobin disorders like thalassemias, indicating its importance in proper erythroid cell function. Its expression levels could provide insight into these disorders.
- HBD testing is already used clinically to diagnose hemoglobin variants and thalassemias, demonstrating its utility as a biomarker relevant to erythroid cells.
- As a hemoglobin subunit, its expression directly reflects erythroid cell functional status. Altered levels would be informative of disturbances in erythropoiesis or oxygen transport.
- It has potential as a blood transcriptional biomarker in conditions affecting hemoglobin synthesis and erythropoiesis.

In summary, the critical role of HBD in oxygen transport and its specificity to erythroid cells, along with its clinical relevance as a diagnostic marker, make it the top choice for inclusion in an erythroid cell transcriptional signature assay. Its expression levels can offer valuable insights into erythroid cell function and hemoglobin disorders.”

GPT-4 chose HBD: “Given the provided information, we should consider the following criteria to select the top candidate gene for a targeted assay to measure differences in transcript abundance for a circulating erythroid cell signature:

1. **Direct Relevance to Erythroid Cells**: The gene should have a clear and well-established role in erythroid cells and erythropoiesis.
2. **Clinical Biomarker Usage**: The gene should already be utilized or show promise as a clinical biomarker, suggesting its measurable importance in clinical settings.

3. **Potential as a Blood Transcriptional Biomarker**: The gene should have potential relevance as a blood transcriptional biomarker, especially in the context of erythropoiesis.
4. **Minimal Relevance to Leukocyte Immune Biology**: As we are targeting erythroid cells, we should prioritize genes with minimal relevance to leukocyte biology or immune function.
5. **Drug Target Relevance**: While this is not the main criterion, knowing whether the gene is a target of existing drugs can suggest its significance in medical contexts.
6. **Therapeutic Relevance for Immune-mediated Diseases**: Given that the assay is for a circulating erythroid cell signature, direct implication in immune-mediated diseases isn't the primary criteria but can offer additional insight.

With the above criteria in mind:

- **HBD**:
    - Has a critical role in erythroid cell function.
    - Is used as a clinical biomarker for diagnosing specific hemoglobin variants and thalassemia.
    - Shows promise as a blood transcriptional biomarker for conditions affecting erythropoiesis.
    - Primarily functions within erythrocytes with no direct involvement in leukocyte biology.
    - Encodes a crucial component of hemoglobin.
    - Not a direct therapeutic target for immune-mediated diseases.
- **SLC1A5, UBE2O, PRDX2, YOD1**:
    - Vary in their relevance to erythroid cells, but none have as direct and clear a connection as HBD.
    - Have varying relevance to leukocyte biology and immune functions.

- Their roles as clinical biomarkers, drug targets, and therapeutic relevance for immune-mediated diseases also vary, but none of these roles prioritize erythroid cell relevance over HBD.

Conclusion: Considering the outlined criteria, **HBD** is the top candidate gene to be included in a targeted assay aiming to measure differences in transcript abundance for a circulating erythroid cell signature. Its direct involvement in erythroid cell function, use as a clinical biomarker, and potential as a blood transcriptional biomarker clearly sets it apart from the other genes in the context of erythroid cells."

Both GPT-4 and Claude identified HBD as the top candidate. Based on its direct role in erythroid cell function, we identify HBD as our primary M12.11 selection for subsequent characterization.

7. Overall pick for M12.11 based on summarized justifications and reference transcriptional datasets.

GPT-4 and Claude were asked to pick a top candidate. They were told to consider the fact-checked summary justifications that were created earlier. They were also told to factor in the gene expression data from the three reference datasets. By using multi-tier prompting, GPT-4 and Claude could integrate all this information to select the best candidate.

8. References

1. Altman MC, Rinchai D, Baldwin N, Toufiq M, Whalen E, Garand M, et al. Development of a fixed module repertoire for the analysis and interpretation of blood transcriptome data. Nat Commun. 2021 Jul 19;12(1):4385.
2. Rinchai D, Roelands J, Toufiq M, Hendrickx W, Altman MC, Bedognetti D, et al. BloodGen3Module: Blood transcriptional module repertoire analysis and visualization using R. Bioinforma Oxf Engl. 2021 Feb 24;btab121.
3. Rinchai D, Altman MC, Konza O, Hässler S, Martina F, Toufiq M, et al. Definition of erythroid cell-positive blood transcriptome phenotypes associated with severe respiratory syncytial virus infection. Clin Transl Med. 2020 Dec;10(8):e244.
4. Linsley PS, Speake C, Whalen E, Chaussabel D. Copy number loss of the interferon gene cluster in melanomas is linked to reduced T cell infiltrate and poor patient prognosis. PloS One. 2014;9(10):e109760.

5. Rinchai D, Chaussabel D. A training curriculum for retrieving, structuring, and aggregating information derived from the biomedical literature and large-scale data repositories. [Internet]. F1000Research; 2022 [cited 2023 Mar 29]. Available from: https://f1000research.com/articles/11-994

6. Rinchai D, Chaussabel D. Assessing the potential relevance of CEACAM6 as a blood transcriptional biomarker [Internet]. F1000Research; 2022 [cited 2023 Mar 31]. Available from: https://f1000research.com/articles/11-1294

7. Sankaran VG, Menne TF, Xu J, Akie TE, Lettre G, Van Handel B, Mikkola HK, Hirschhorn JN, Cantor AB, Orkin SH. Human fetal hemoglobin expression is regulated by the developmental stage-specific repressor BCL11A. *Science*. 2008 Dec 19;322(5909):1839-42. doi: 10.1126/science.1165409. Epub 2008 Dec 4. PMID: 19056937.

8. Verma S, Bhargava M, Mittal S, Gupta R. Homozygous delta-beta Thalassemia in a Child: a Rare Cause of Elevated Fetal Hemoglobin. *Iran J Ped Hematol Oncol*. 2013;3(1):222-7. Epub 2013 Jan 22. PMID: 24575268; PMCID: PMC3915439.

9. Scalise M, Pochini L, Console L, Losso MA and Indiveri C (2018) The Human SLC1A5 (ASCT2) Amino Acid Transporter: From Function to Structure and Role in Cell Biology. Front. *Cell Dev. Biol.* 6:96. doi: 10.3389/fcell.2018.00096

10. Nguyen AT, Prado MA, Schmidt PJ, Sendamarai AK, Wilson-Grady JT, Min M, Campagna DR, Tian G, Shi Y, Dederer V, Kawan M, Kuehnle N, Paulo JA, Yao Y, Weiss MJ, Justice MJ, Gygi SP, Fleming MD, Finley D. UBE2O remodels the proteome during terminal erythroid differentiation. *Science*. 2017 Aug 4;357(6350):eaan0218. doi: 10.1126/science.aan0218. PMID: 28774900; PMCID: PMC5812729.

11. Wood ZA, Poole LB, Karplus PA. Peroxiredoxin evolution and the regulation of hydrogen peroxide signaling. *Science*. 2003 Apr 25;300(5619):650-3. doi: 10.1126/science.1080405. PMID: 12714747.

12. Kim Y, Kim W, Song Y, et al. Deubiquitinase YOD1 potentiates YAP/TAZ activities through enhancing ITCH stability. Proc Natl Acad Sci USA. 2017;114(18):4691–6. https://doi.org/10.1073/pnas.1620306114.

13. Zuo Q, Cheng S, Huang W, Bhatti MZ, Xue Y, Zhang Y, Zhang B, Li L, Wu L, Fu J, Chen J, Li X. REGγ Contributes to Regulation of Hemoglobin and Hemoglobin δ Subunit. *Oxid Med Cell Longev.* 2017;2017:7295319. doi: 10.1155/2017/7295319. Epub 2017 Jul 16. PMID: 28798860; PMCID: PMC5534318.

14. Ni, F., Yu, WM., Li, Z. et al. Critical role of ASCT2-mediated amino acid metabolism in promoting leukaemia development and progression. *Nat Metab* 1, 390–403 (2019). https://doi-org.libproxy2.usc.edu/10.1038/s42255-019-0039-6

15. Sobotta, M., Liou, W., Stöcker, S. et al. Peroxiredoxin-2 and STAT3 form a redox relay for H2O2 signaling. *Nat Chem Biol* 11, 64–70 (2015). https://doi.org/10.1038/nchembio.1695

16. Sharjeel Syed, Jihad Aljabban, Jonathan Trujillo, Saad Syed, Robert Cameron, Maryam Panahiazar, Dexter Hadley. Identifying Potential Drug Targets for Sickle Cell Disease through Gene Expression and Pathway Analysis of GEO Data. *Blood* 136, 15–16 (2020). https://doi.org/10.1182/blood-2020-140904.

17. Yangzom D. Bhutia, Ellappan Babu, Sabarish Ramachandran, Vadivel Ganapathy; Amino Acid Transporters in Cancer and Their Relevance to "Glutamine Addiction": Novel Targets for the Design of a New Class of Anticancer Drugs. Cancer Res 1 May 2015; 75 (9): 1782–1788. https://doi.org/10.1158/0008-5472.CAN-14-3745

18. Voutsadakis IA. The ubiquitin-proteasome system in colorectal cancer. *Biochim Biophys Acta*. 2008 Dec;1782(12):800-8. doi: 10.1016/j.bbadis.2008.06.007. Epub 2008 Jun 19. PMID: 18619533.

19. Ernst A, Avvakumov G, Tong J, Fan Y, Zhao Y, Alberts P, Persaud A, Walker JR, Neculai AM, Neculai D, Vorobyov A, Garg P, Beatty L, Chan PK, Juang YC, Landry MC, Yeh C, Zeqiraj E, Karamboulas K, Allali-Hassani A, Vedadi M, Tyers M, Moffat J, Sicheri F, Pelletier L, Durocher D, Raught B, Rotin D, Yang J, Moran MF, Dhe-Paganon S, Sidhu SS. A strategy for modulation of enzymes in the ubiquitin system. *Science*. 2013 Feb 1;339(6119):590-5. doi: 10.1126/science.1230161. Epub 2013 Jan 3. PMID: 23287719; PMCID: PMC3815447.

20. Cunniff B, Benson K, Stumpff J, Newick K, Held P, Taatjes D, Joseph J, Kalyanaraman B, Heintz NH. Mitochondrial-targeted nitroxides disrupt mitochondrial architecture and inhibit expression of peroxiredoxin 3 and FOXM1 in malignant mesothelioma cells. *J Cell Physiol*. 2013 Apr;228(4):835-45. doi: 10.1002/jcp.24232. PMID: 23018647; PMCID: PMC3928986.

21. Forget, B.G. (1998), Molecular Basis of Hereditary Persistence of Fetal Hemoglobin. *Annals of the New York Academy of Sciences*, 850: 38-44. https://doi-org.libproxy1.usc.edu/10.1111/j.1749-6632.1998.tb10460.x

22. Zhang X, Zhang J, Zhang L, van Dam H, ten Dijke P. UBE2O negatively regulates TRAF6-mediated NF-κB activation by inhibiting TRAF6 polyubiquitination. *Cell Res*. 2013 Mar;23(3):366-77. doi: 10.1038/cr.2013.21. Epub 2013 Feb 5. PMID: 23381138; PMCID: PMC3587711.

23. Schweitzer K, Bozko PM, Dubiel W, Naumann M. CSN controls NF-kappaB by deubiquitinylation of IkappaBalpha. *EMBO J*. 2007 Mar 21;26(6):1532-41. doi: 10.1038/sj.emboj.7601600. Epub 2007 Feb 22. PMID: 17318178; PMCID: PMC1829370.

24. Rhee SG, Kil IS. Multiple Functions and Regulation of Mammalian Peroxiredoxins. *Annu Rev Biochem*. 2017 Jun 20;86:749-775. doi: 10.1146/annurev-biochem-060815-014431. Epub 2017 Feb 2. PMID: 28226215.

25. Thein SL. The molecular basis of β-thalassemia. *Cold Spring Harb Perspect Med*. 2013 May 1;3(5):a011700. doi: 10.1101/cshperspect.a011700. PMID: 23637309; PMCID: PMC3633182.

26. Yau RG, Doerner K, Castellanos ER, Haakonsen DL, Werner A, Wang N, Yang XW, Martinez-Martin N, Matsumoto ML, Dixit VM, Rape M. Assembly and Function of Heterotypic Ubiquitin Chains in Cell-Cycle and Protein Quality Control. *Cell*. 2017 Nov 2;171(4):918-933.e20. doi: 10.1016/j.cell.2017.09.040. Epub 2017 Oct 12. PMID: 29033132; PMCID: PMC5669814.

27. Rhee SG, Woo HA, Kil IS, Bae SH. Peroxiredoxin functions as a peroxidase and a regulator and sensor of local peroxides. *J Biol Chem*. 2012 Feb 10;287(7):4403-10. doi: 10.1074/jbc.R111.283432. Epub 2011 Dec 6. PMID: 22147704; PMCID: PMC3281607.
