# Supplementary material for: Harnessing large language models (LLMs) for candidate gene prioritization and selection
Source: J Transl Med. 2023 Oct 16;21:728. doi: 10.1186/s12967-023-04576-8 (PMC10580627; doi:10.1186/s12967-023-04576-8)
Supplement: Supplementary file 7 — Additional file 7: Is the prioritization report for module M13.26 authored by Marina Yurieva. The file is shared in the PDF format. [file 12967_2023_4576_MOESM7_ESM.pdf]

M13.26 Prioritization report

Prepared by: Marina Yurieva (The Jackson Laboratory for Genomic Medicine, Farmington, CT, USA)

1. Composition

The module M13.26 belongs to Aggregate A37 from the BloodGen3 fixed module repertoire [22, 23]. This module aggregate is associated with circulating erythroid cells. It was found to be associated with RSV disease severity [24]. We showed in the same study that the abundance of A27 transcripts is also elevated in the blood of patients with stage IV melanoma [24].
It comprises 40 genes: AP2A1, ATP6V0C, BMP2K, C16ORF35, C19ORF22, C2ORF24, C9ORF78, CCDC23, CREG1, EIF2AK1, ELOF1, FAM100A, FOXO4, FURIN, GABARAPL2, GCLC, GPX1, H1F0, HDGF, ISCA1, LYL1, MAF1, MKRN1, NINJ2, PNP (NP), PIM1, PINK1, PNPLA2, PRR6, PSMF1, RFESD, RNF123, RNF14, STK33, STOM, SYT15, TERF2IP, TFDP2, XPO7, YPEL3

2. Functional convergence

Functional associations were identified among the genes constituting M13.26 using GPT-4 (see Step 2 method for details).

Functional Themes	Associated Genes	Comments
Cellular Stress and Homeostasis	GPX1, GCLC, PIM1, FOXO4, EIF2AK1, PINK1	Several genes (GPX1, GCLC, FOXO4, EIF2AK1, PINK1) are involved in pathways that counteract cellular stress, particularly oxidative stress.
Protein Trafficking, Processing, and Degradation	AP2A1, GABARAPL2, FURIN, XPO7, PNPLA2	Genes like AP2A1, GABARAPL2, and FURIN play roles in processing, modifying, or trafficking proteins within the cell.
Regulation of Gene Expression	LYL1, MAF1, TERF2IP, TFDP2	Several genes (LYL1, MAF1, TERF2IP, TFDP2) regulate the expression of other genes, impacting a wide range of cellular functions.
Signaling and Cellular Communication	BMP2K, STK33	BMP2K has a role in the bone morphogenetic protein (BMP) signaling pathway.<br>STK33 is a serine/threonine kinase, suggesting involvement in cellular signaling.

Nucleotide Metabolism and Cell Proliferation	PNP (NP)	PNP (NP) is involved in purine metabolism.
Cellular Structures and Interactions	STOM, NINJ2	STOM is involved in erythrocyte membrane integrity.<br>NINJ2 is associated with nerve injury and regeneration.

3. Scoring and prioritization

Genes were scored on six criteria using GPT-4 and Claude. The scores were averaged, and candidate genes were ranked according to their cumulative scores (**Figure 1**, Methods: Step 3 and Step 4).

The two LLMs were requested to score each gene on the following six statements:

a. The gene is associated with erythroid cells or erythropoiesis.

b. The gene is currently being used as a biomarker in clinical settings.

c. The gene has potential value as a blood transcriptional biomarker.

d. The gene is relevant to circulating leukocytes immune biology.

e. The gene is a known drug target.

f. The gene is therapeutically relevant for immune-mediated diseases.

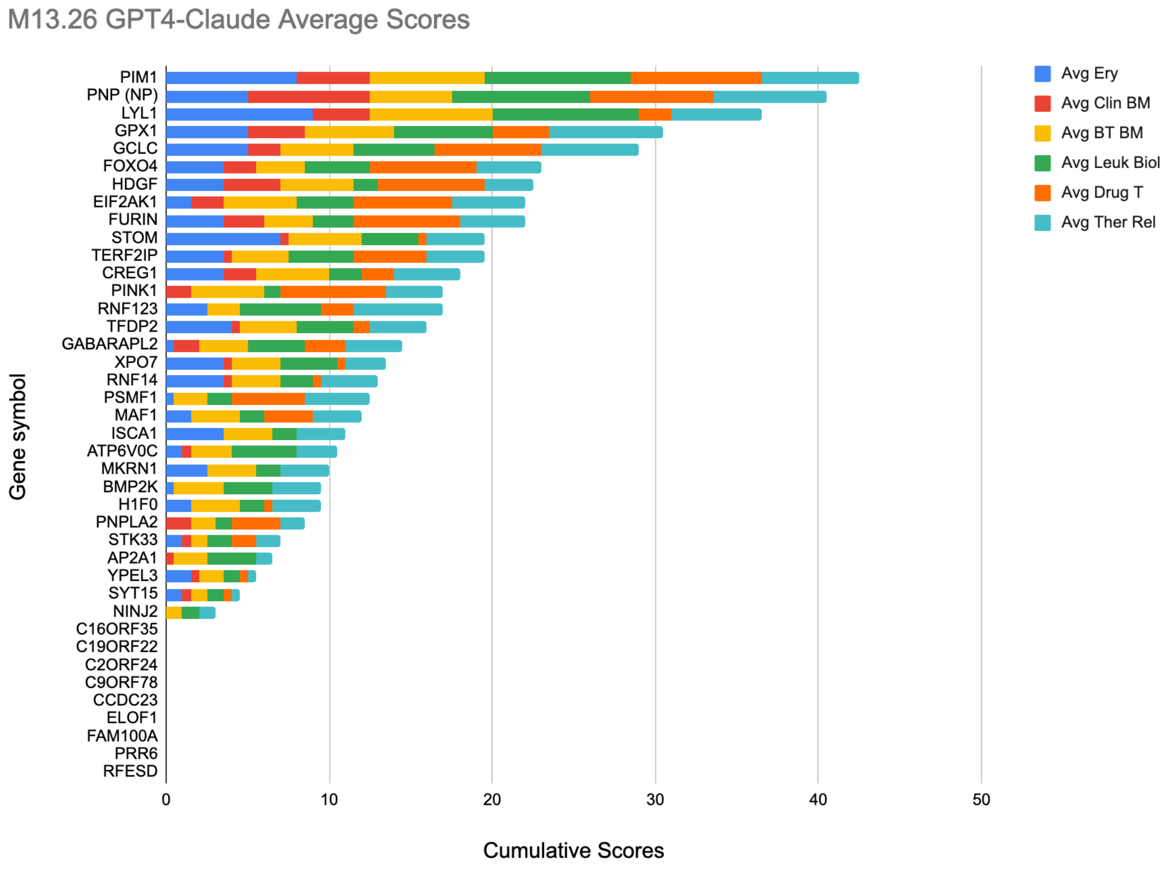

Figure 1: The stacked bar graph shows cumulative scores across six criteria for the 40 M13.26 genes. The top five genes selected for further evaluation were: PIM1, PNP (NP), LYL1, GPX1 and GCLC.

M13.26 - Top 5 Candidates

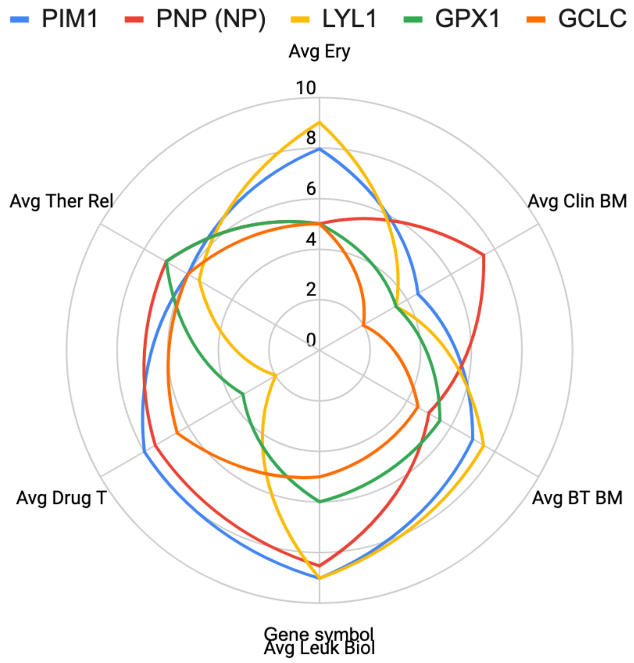

Figure 2: The radar plot represents individual scores for the 5 top scoring genes: PIM1, PNP (NP), LYL1, GPX1 and GCLC.

4. Knowledge-driven evaluation of top five candidates

Justifications given by the GPT-4 and Claude for the scores provided across the 6 criteria were compiled and summarized by GPT-4 (Methods: Step 5). For each statement pertinent references were retrieved using GPT-4 or Claude, with the relevance of each reference checked and attributed manually by the researcher authoring this report.

Function: "The PIM1 gene encodes a serine/threonine kinase, a member of the serine/threonine kinases family, which phosphorylates other proteins by adding a phosphate group. This phosphorylation is vital for various cellular processes, such as cell division, survival, proliferation, and differentiation. Notably, PIM1 acts as a proto-oncogene, and its mutation or overexpression can lead to hematologic malignancies, with its role evident in hematopoiesis, leukemias, and lymphomas [1]. Similarly, the LYL1 gene instructs the synthesis of a protein fundamental for early hematopoietic stem cell and lymphocyte development. As a transcription factor, LYL1 regulates the proliferation and differentiation of hematopoietic stem cells,

influencing both lymphoid and myeloid lineages [2]. In contrast, the GPX1 gene produces the selenium-containing enzyme glutathione peroxidase 1, essential for neutralizing reactive oxygen species and preventing oxidative damage by reducing harmful molecules like hydrogen peroxide, lipid peroxides, and organic hydroperoxide [3]. Lastly, the GCLC gene produces the heavy subunit of glutamate-cysteine ligase (GCL), the pivotal enzyme in glutathione biosynthesis. Glutathione, composed of glutamate, cysteine, and glycine, serves as a primary defense against oxidative stress and ensures cellular redox balance [4]. On a different note, the enzyme PNP, central to purine metabolism, drives the purine salvage pathway by facilitating the reversible phosphorolysis of purine nucleosides into their corresponding purine bases and ribose-1-phosphate [5]."

a. Relevance to erythroid cells and erythropoiesis:

"Current evidence underscores the significant roles of PIM1 and LYL1 in erythropoiesis. Specifically, PIM1 is closely associated with erythropoiesis, notably within the context of malignancies [6]. Similarly, LYL1 has been firmly established as a vital player in hematopoiesis, with an emphasis on the development of erythroid cells [7]. On the other hand, while the GPX1 gene is implicated in the antioxidant defense mechanism of erythroid cells due to its anti-oxidative functions, its direct role in erythropoiesis awaits more extensive validation [8]. The GCLC gene, recognized for its contribution to redox balance and cellular defense against oxidative stress, is presumed to be relevant to erythroid cell health and erythropoiesis, yet its direct involvement requires additional verification [9]. Contradictorily, even though PNP was previously believed to lack a direct association with erythropoiesis, recent insights reveal its elevated expression in erythroid lineage cells, emphasizing its importance. Any loss in PNP activity triggers a dGTP accumulation, impairing DNA synthesis and leading to hematopoietic anomalies [10]. " **<I don't think the last statement about PNP is correct - MY>**

b. Is used as a clinical biomarker:

"PIM1, while indicating prognostic significance in certain hematological malignancies, is not conventionally employed as a clinical biomarker [6]. Similarly, LYL1's association with specific leukemia types hints at its potential biomarker utility, yet its routine clinical use is absent [11]. In contrast, GPX1 has clinical relevance as a biomarker, especially in gauging antioxidant capacity

and detecting oxidative stress, making it particularly pertinent in cardiovascular diseases and cancer contexts [12]. GCLC's involvement in glutathione synthesis and oxidative stress suggests potential as a biomarker for conditions like neurodegenerative diseases and cancer, though its current clinical application is limited [9]. Lastly, PNP serves as a biomarker for PNP deficiency and has established significance in monitoring disease status in conditions like acute leukemias; however, its decreased activity's connection to T-cell lymphoblastic leukemia/lymphoma needs further validation [13].”

c. Potential relevance as a blood transcriptional biomarker:

“PIM1, with its established role in hematopoiesis and hematological malignancies, presents promising potential as a blood transcriptional biomarker, though it awaits complete validation [6]. LYL1, integral to hematopoiesis, similarly holds promise as a transcriptional biomarker in hematopoietic differentiation [14]. While GPX1's role in the oxidative stress response suggests its potential utility as a blood transcriptional biomarker, its validation remains in the preliminary stages [8]. GCLC's pivotal function in glutathione synthesis, crucial for cellular redox balance, hints at its potential as a transcriptional biomarker, especially in conditions linked to oxidative stress; however, its validation is not yet comprehensive [9]. PNP, although not widely recognized as a transcriptional biomarker, has been explored in specific contexts such as its deficiency and in ALL patients, suggesting its potential prognostic value, but it too requires further validation [13].”

d. Relevance to leukocytes immune biology:

“PIM1 is robustly associated with the biology of circulating leukocytes, stemming from its pivotal role in hematopoiesis and hematological malignancies [15]. LYL1, similarly, has a pronounced link to both leukocyte and lymphocyte biology, particularly given its involvement in lymphocyte development [2]. GPX1, through its antioxidative action, plays a role in leukocyte immune biology, as it counteracts reactive oxygen species which function as immune response modulators, though its direct relationship requires additional validation [8]. GCLC, due to its influence on redox balance and oxidative stress, holds potential relevance to immune biology in circulating leukocytes; however, this association warrants further investigation [9]. Lastly, PNP,

prominently expressed in lymphocytes, plays a central role in their proliferation and function, with a PNP deficiency directly resulting in compromised T cell immunity [13].”

e. Is a known drug target:

“PIM1 kinase is robustly identified as a potential drug target, particularly for hematological malignancies and cancer, echoing the significant evidence pointing towards its relevance in hematological cancers [6]. Although LYL1's association with hematopoiesis and leukemia hints at its potential as a drug target, it is not currently recognized as such [14]. GPX1, given its role in addressing oxidative stress, is postulated as a potential drug target in cancer biology, but it isn't established as a major one yet [8]. GCLC's importance in oxidative stress positions it as a promising drug target, especially in the realms of neurodegenerative diseases and cancer [9]. Meanwhile, PNP stands out with tangible progress in drug development: PNP inhibitors, such as forodesine, are being formulated for lymphoid malignancies, including T-cell acute lymphoblastic leukemia (ALL) [16].”

f. Potential therapeutic relevance for immune-mediated diseases:

“PIM1, while established in hematopoiesis and cancer, requires further elucidation concerning its role in immune-mediated diseases, despite evidence hinting at its relevance [6]. LYL1, pivotal in lymphocyte development, may possess significance for immune-mediated diseases, but concrete evidence, particularly linking it to immune-mediated leukemias, remains scarce [14]. GPX1, instrumental in antioxidant responses, holds potential relevance to immune-mediated diseases, with some evidence supporting this association, yet more comprehensive studies are needed [8]. Similarly, GCLC, central to glutathione synthesis and redox balance, may play a part in the pathogenesis of immune-mediated conditions, meriting additional research [9]. PNP stands distinct in this context: its deficiency leads to compromised lymphocyte function and immune irregularities, underscoring the therapeutic promise of PNP supplementation, especially for PNP-deficient individuals and select autoimmune conditions [13, 17].”

5. Examining expression patterns of top 5 candidates across leukocyte populations

The expression patterns of the top 5 candidate genes were examined across diverse leukocyte populations and hematopoietic precursors using two reference transcriptomic datasets.

5.1 Densely interconnected transcriptional circuits control cell states in human hematopoiesis – (GSE24759)
http://developmentalimmunology.gxbsidra.org/dm3/geneBrowser/show/4000026

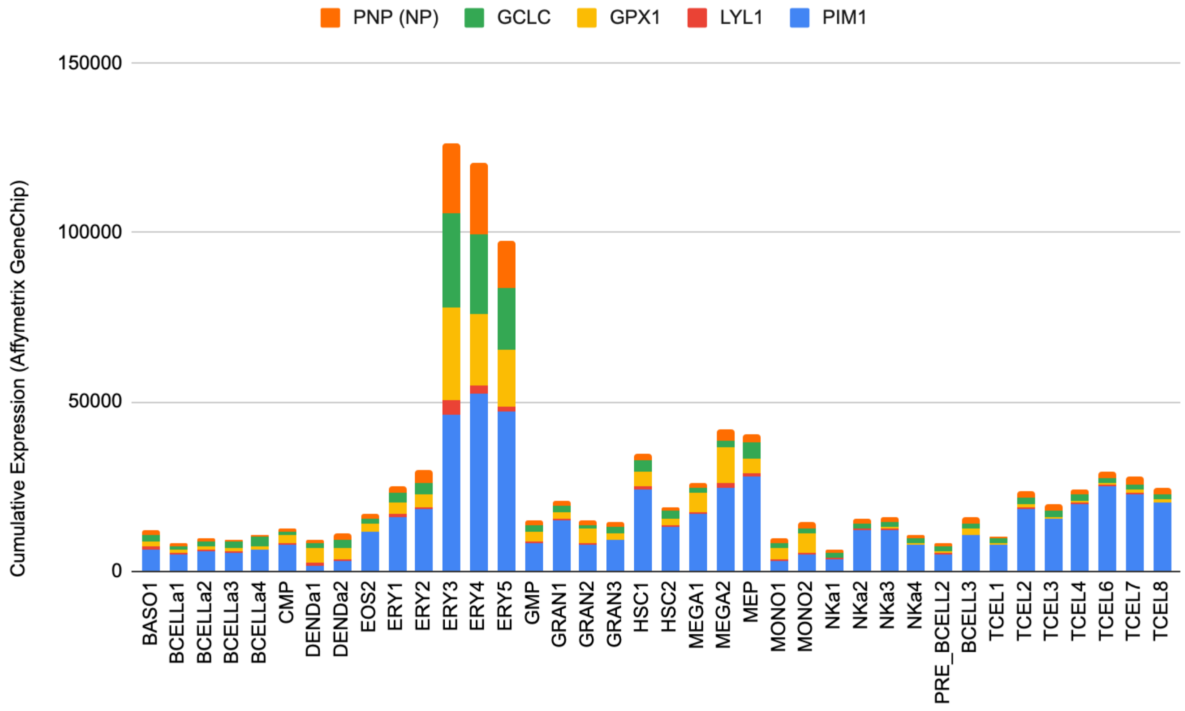

This stacked bar graph shows the expression levels of the five candidate genes. ERY3-5 populations are CD71+ GLYA+ erythroid cells. The original article provides full experimental details: [18]. The web link above also provides access to more complete information about this dataset as well as individual gene profiles.

5.2 Next generation sequencing of human immune cell subsets across diseases – (GSE60424)
http://cd2k.gxbsidra.org/dm3/geneBrowser/show/4000098

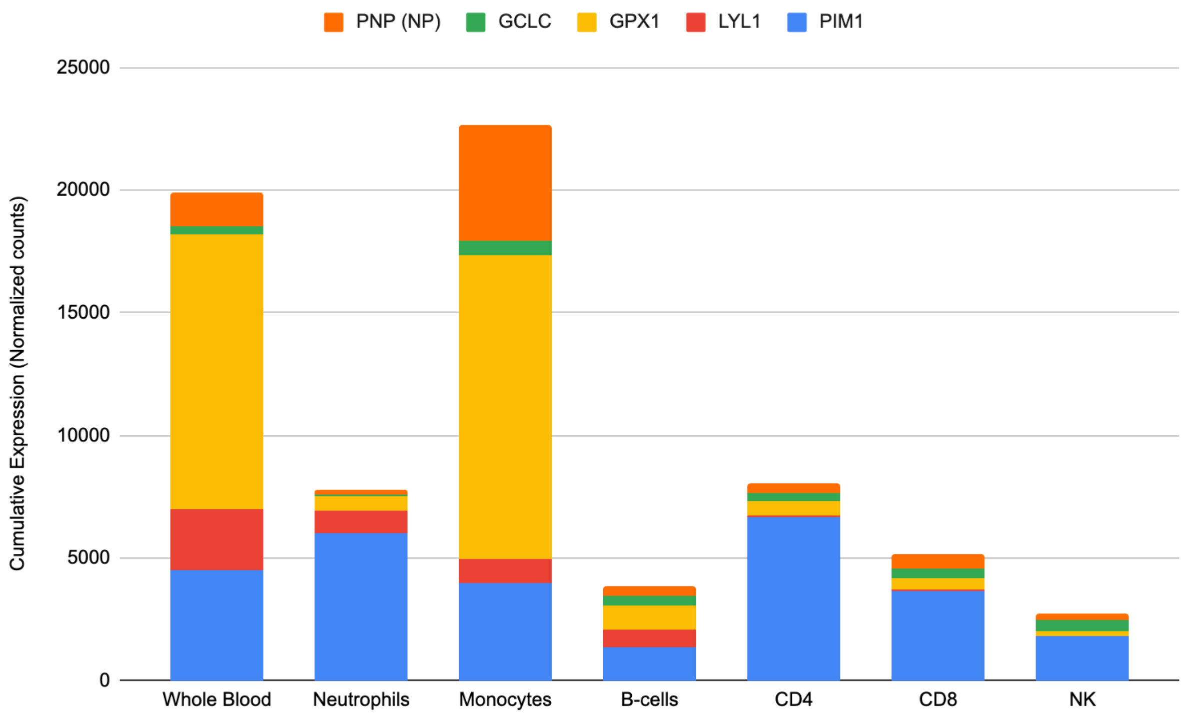

This stacked bar graph shows the expression levels measured by RNAseq of the five candidate genes across six leukocyte populations and whole blood. The abundance is shown for each gene in each population for an average of up to 20 samples. Additional experimental details are accessible via the link provided above and is available as part of this sample series GEO record (GSE60424) [19].

6. Overall pick(s) for M13.26

We aim to establish a universal immune profiling assay that measures transcript abundance of a panel of genes in whole blood using targeted molecular profiling technology (Immune profiling transcriptome fingerprinting assay). The top candidate identified here among the set of genes constituting M13.26 will be subjected to more in-depth evaluation using a pre-established workflow [20, 21].

Claude and GPT-4 were prompted to select a top candidate on the basis of the information summarized above in section 4, taking into account all six scoring criteria equally (Methods: Step 7).

Claude chose PIM1 and LYL1: "Based on the information provided, I would suggest PIM1 and LYL1 as the top candidate genes for inclusion in a targeted assay measuring differences in transcript abundance for a circulating erythroid cell signature.

The key reasons are:

- Both PIM1 and LYL1 have well-established roles in erythropoiesis and are highly relevant to erythroid cell biology. This makes them strong candidates for an erythroid cell signature.
- They are involved in key processes like proliferation, differentiation and development of erythroid lineage cells. Altered expression could reflect disturbances in erythropoiesis.
- PIM1 in particular is closely tied to hematological malignancies involving erythroid cells, so it could be informative of disease states.
- While not conventional biomarkers, both genes have potential clinical and biomarker utility based on their biological importance and links to hematopoietic diseases.
- As transcription factors and regulators, their expression levels are likely to reflect functional alterations in erythroid cells.

In comparison, while GPX1 and GCLC are antioxidants with presumed roles in erythropoiesis, their direct relevance requires more validation. PNP is important for hematopoiesis but its link to erythroid cells specifically is less evident.

So in summary, PIM1 and LYL1 emerge as lead candidate genes for an erythroid cell transcriptional signature based on their biological significance and high relevance to erythroid cell processes and diseases. Their expression levels can offer key insights into erythroid cell biology and function."

GPT-4 chose LYL1: "Considering the criteria, **LYL1** appears as the top candidate for inclusion in a targeted assay aiming to measure differences in transcript abundance for a circulating erythroid cell signature. This decision is based on its pivotal role in erythropoiesis, the development of erythroid cells, its potential as a blood transcriptional biomarker, and its pronounced links to leukocyte and lymphocyte biology. While other genes also show relevance, LYL1 stands out for its multifaceted significance in the context of erythroid cell signature.

Based on the provided information, let's assess each gene against the specified criteria:

1. **Relevance to erythroid cells and erythropoiesis**:
   - PIM1 and LYL1 are closely associated with erythropoiesis, notably within the context of malignancies and the development of erythroid cells.
   - GPX1 and GCLC are involved in redox balance and oxidative stress, which are essential for erythroid cell health, but their direct roles in erythropoiesis are still under validation.
   - PNP has shown elevated expression in erythroid lineage cells, indicating its importance in erythropoiesis.
2. **Used as a clinical biomarker**:
   - PIM1 and LYL1 are not conventionally employed as clinical biomarkers.
   - GPX1 and GCLC have potential but limited current clinical applications.
   - PNP serves as a biomarker for its deficiency and in specific leukemia contexts.
3. **Potential relevance as a blood transcriptional biomarker**:
   - Both PIM1 and LYL1 hold significant potential as blood transcriptional biomarkers.
   - GPX1, GCLC, and PNP also show promise but require further validation.
4. **Relevance to leukocytes immune biology**:
   - PIM1 and LYL1 have pronounced links to leukocyte and lymphocyte biology.
   - GPX1, GCLC, and PNP also have relevance, with PNP particularly central to lymphocyte proliferation and function.
5. **Known drug target**:
   - PIM1 is a recognized drug target, especially for hematological malignancies.
   - PNP has tangible progress in drug development, especially for lymphoid malignancies.
   - GPX1, GCLC, and LYL1 hint at potential but are not major drug targets yet.
6. **Potential therapeutic relevance for immune-mediated diseases**:
   - PIM1, LYL1, GPX1, and GCLC all show potential relevance but require further studies for validation.

- PNP is especially relevant due to its direct impact on lymphocyte function and implications in immune-mediated diseases.”

The LLMs did not incorporate reference transcriptional profiling data in their evaluation and prioritization of candidate genes. Given its comparatively higher expression levels and restriction across leukocyte populations, our top choice based on this criterion is PIM1. Considering this, in conjunction with the rationale provided by both LLMs and our understanding of PIM1 and LYL1 biological and clinical relevance, we identify PIM1 as our primary M13.26 selection for subsequent characterization.

7. **Overall pick for M13.26 based on summarized justifications and reference transcriptional datasets.**

   GPT-4 and Claude were asked to pick a top candidate. They were told to consider the fact-checked summary justifications that were created earlier. They were also told to factor in the gene expression data from the three reference datasets. By using multi-tier prompting, GPT-4 and Claude could integrate all this information to select the best candidate.

   The transcription of the final round of prompting is shown below.

Prompts	CLAUDE	GPT-4

PROMPT 1: Based on the summary below, please recommend the top candidate gene to include in a targeted blood transcriptional profiling panel. The intent is for the selected gene to be representative of an erythroid cell signature associated with response to mRNA vaccines, severity of RSV infection and that found to be expressed at high levels in patients with metastatic melanoma and in liver transplant recipients. Please weigh the following criteria equally in your recommendation: 1) Relevance to erythroid cells and erythropoiesis, 2) Current use as a biomarker, 3) Potential use as a blood transcriptional biomarker, 4) Relevance to leukocyte immune biology, 5) Status as a known drug target, 6) Therapeutic relevance for immune mediated diseases: *[input summarized and fact-checked justifications from Step 5]*	Based on the provided summary and criteria, I would recommend SLC2A1 as the top gene to include in the targeted blood transcriptional profiling panel.<br><br>The key reasons are:<br><br>- SLC2A1 has a clear and direct role in erythroid cells and erythropoiesis through encoding the GLUT1 glucose transporter, which facilitates essential glucose transport in red blood cells. This aligns well with the intent for a gene relevant to erythroid biology.<br><br>- SLC2A1 is already recognized as a clinical biomarker for diagnosing GLUT1 deficiency syndrome. This satisfies the criterion for current use as a biomarker.<br><br>- As a key player in glucose metabolism, SLC2A1 has strong potential utility as a blood transcriptional biomarker in conditions like diabetes and cancer where metabolism is altered. This fulfills the biomarker potential criterion.<br><br>- SLC2A1 is expressed in leukocytes and helps regulate inflammatory responses, indicating relevance to	Based on the detailed information provided, the SLC2A1 gene appears to be the most suitable candidate for inclusion in a targeted blood transcriptional profiling panel, particularly when considering the six equally weighed criteria:<br><br>Relevance to erythroid cells and erythropoiesis: SLC2A1 is directly implicated in erythroid cells, primarily erythrocytes, facilitating the crucial process of glucose transport. This is pivotal for cellular metabolism, including that of red blood cells. This makes it highly relevant to erythropoiesis.<br><br>Current use as a biomarker: SLC2A1 is a recognized clinical biomarker for GLUT1 deficiency syndrome. It's already utilized in clinical settings for diagnosis, enhancing its relevance as a biomarker.<br><br>Potential use as a blood transcriptional biomarker: SLC2A1 has potential utility in conditions where glucose metabolism is altered, such as diabetes and certain cancers. Although further validation is needed, its potential for this role is significant.

	immune biology. This matches the immune biology criterion.<br><br>- SLC2A1 is an emerging drug target, with GLUT1 inhibitors under investigation as possible cancer therapeutics. This satisfies the drug target criterion.<br><br>- Finally, SLC2A1's metabolic role makes it potentially relevant for targeting immune-mediated diseases like type 1 diabetes. This addresses the immune disease relevance criterion.<br><br>In summary, SLC2A1 ranks highly across all the stated criteria, especially for its erythroid cell links, existing biomarker use, biomarker potential, and immune relevance. For these reasons, I recommend it as the top gene for inclusion in the desired transcriptional profiling panel.	Relevance to leukocyte immune biology: SLC2A1 is expressed in leukocytes and facilitates inflammatory responses by regulating glucose uptake. This directly ties it to immune function in circulating leukocytes, a significant point considering the gene needs to be relevant to both erythroid and leukocyte cell types.<br><br>Status as a known drug target: SLC2A1 is under investigation as a promising drug target, particularly for anti-cancer agents through GLUT1 inhibitors. This makes it relevant in therapeutic contexts.<br><br>Therapeutic relevance for immune-mediated diseases: Given its role in cellular metabolism, SLC2A1 has implications for immune-mediated diseases like type 1 diabetes. GLUT1 inhibitors are particularly interesting in this therapeutic context.<br><br>In summary, SLC2A1 seems to best fulfill all six criteria: it is relevant to both erythroid cells and immune biology, has current and potential future utility as a biomarker, is a known drug target, and has therapeutic relevance in immune-

		mediated diseases. Therefore, this gene appears to be the top candidate for further investigation in a targeted blood transcriptional profiling panel.

8. References

1. Nawijn, M. C., Alendar, A., & Berns, A. (2011). For better or for worse: the role of Pim oncogenes in tumorigenesis. *Nature reviews Cancer*, 11(1), 23-34.
2. Capron C, Lécluse Y, Kaushik AL, Foudi A, Lacout C, Sekkai D, Godin I, Albagli O, Poullion I, Svinartchouk F, Schanze E, Vainchenker W, Sablitzky F, Bennaceur-Griscelli A, Duménil D. The SCL relative LYL-1 is required for fetal and adult hematopoietic stem cell function and B-cell differentiation. Blood. 2006 Jun 15;107(12):4678-86. doi: 10.1182/blood-2005-08-3145. Epub 2006 Mar 2. PMID: 16514064. **<this reference was corrected manually (wrong year and authorship)>**
3. Arthur, J. R. (2000). The glutathione peroxidases. *Cellular and molecular life sciences*, 57(13-14), 1825-1835.
4. Meister, A. (1988). Glutathione metabolism and its selective modification. The Journal of biological chemistry, 263(33), 17205-17208.
5. Bzowska, A., Kulikowska, E., & Shugar, D. (2000). Purine nucleoside phosphorylases: properties, functions, and clinical aspects. *Pharmacology & Therapeutics*, 88(3), 349-425.
6. Narlik-Grassow, M., Blanco-Aparicio, C., & Carnero, A. (2014). The PIM family of serine/threonine kinases in cancer. *Medicinal research reviews, 34*(1), 136-159.
7. Capron et al. Expression of LYL1 represses hemopoietic commitment of embryonic stem cells. Blood. 2006. PMID: 16403911. **<manual reference entry (AI-provided was incorrect)>**
8. Brigelius-Flohé, R., & Maiorino, M. (2013). Glutathione peroxidases. *Biochimica et Biophysica Acta (BBA)-General Subjects, 1830*(5), 3289-3303.
9. Lu, S. C. (2013). Glutathione synthesis. *Biochimica et Biophysica Acta (BBA)-General Subjects, 1830*(5), 3143-3153.

10. Simmonds HA, Watson AR, Webster DR, Sahota A, Perrett D. GTP depletion and other erythrocyte abnormalities in inherited PNP deficiency. Biochem Pharmacol. 1982 Mar 15;31(6):941-6. doi: 10.1016/0006-2952(82)90324-0. PMID: 6805478. **<manual reference entry (AI-provided was incorrect)>**
11. Meng, YS., Khoury, H., Dick, J. *et al.* Oncogenic potential of the transcription factor LYL1 in acute myeloblastic leukemia. *Leukemia* **19**, 1941–1947 (2005). https://doi.org/10.1038/sj.leu.2403836 **<manual reference entry (AI-provided was outdated)>**
12. Nemoto M, Nishimura R, Sasaki T, Hiki Y, Miyashita Y, Nishioka M, Fujimoto K, Sakuma T, Ohashi T, Fukuda K, Eto Y, Tajima N. Genetic association of glutathione peroxidase-1 with coronary artery calcification in type 2 diabetes: a case control study with multi-slice computed tomography. Cardiovasc Diabetol. 2007 Sep 7;6:23. doi: 10.1186/1475-2840-6-23. PMID: 17825092; PMCID: PMC2041944. **<manual reference entry (AI-provided was incorrect)>**
13. Arpaia, E., Benveniste, P., Di Cristofano, A., Gu, Y., Dalal, I., Kelly, S., ... & Roifman, C. M. (2000). Mitochondrial basis for immune deficiency. Evidence from purine nucleoside phosphorylase–deficient mice. *The Journal of Experimental Medicine*, 191(12), 2197-2208.
14. Souroullas, G. P., Salmon, J. M., Sablitzky, F., Curtis, D. J., & Goodell, M. A. (2009). Adult hematopoietic stem and progenitor cells require either Lyl1 or Scl for survival. *Cell stem cell, 4*(2), 180-186.
15. Decker S, Finter J, Forde AJ, Kissel S, Schwaller J, Mack TS, Kuhn A, Gray N, Follo M, Jumaa H, Burger M, Zirlik K, Pfeifer D, Miduturu CV, Eibel H, Veelken H, Dierks C. PIM kinases are essential for chronic lymphocytic leukemia cell survival (PIM2/3) and CXCR4-mediated microenvironmental interactions (PIM1). Mol Cancer Ther. 2014 May;13(5):1231-45. doi: 10.1158/1535-7163.MCT-13-0575-T. Epub 2014 Mar 21. PMID: 24659821. **<manual reference entry (AI-provided was incorrect)>**

16. Makita S, Maeshima AM, Maruyama D, Izutsu K, Tobinai K. Forodesine in the treatment of relapsed/refractory peripheral T-cell lymphoma: an evidence-based review. Onco Targets Ther. 2018 Apr 20;11:2287-2293. doi: 10.2147/OTT.S140756. PMID: 29719411; PMCID: PMC5916385. **<manual reference entry (AI-provided was incorrect)>**
17. Kicska, G. A., Long, L., Hörig, H., Fairchild, C., Tyler, P. C., Furneaux, R. H., ... & Schramm, V. L. (2001). Immucillin H, a powerful transition-state analog inhibitor of purine nucleoside phosphorylase, selectively inhibits human T lymphocytes. *Proceedings of the National Academy of Sciences*, 98(8), 4593-4598.
18. Novershtern N, Subramanian A, Lawton LN, Mak RH, Haining WN, McConkey ME, et al. Densely interconnected transcriptional circuits control cell states in human hematopoiesis. Cell. 2011 Jan 21;144(2):296–309.
19. Linsley PS, Speake C, Whalen E, Chaussabel D. Copy number loss of the interferon gene cluster in melanomas is linked to reduced T cell infiltrate and poor patient prognosis. PloS One. 2014;9(10):e109760.
20. Rinchai D, Chaussabel D. A training curriculum for retrieving, structuring, and aggregating information derived from the biomedical literature and large-scale data repositories. [Internet]. F1000Research; 2022 [cited 2023 Mar 29]. Available from: https://f1000research.com/articles/11-994
21. Rinchai D, Chaussabel D. Assessing the potential relevance of CEACAM6 as a blood transcriptional biomarker [Internet]. F1000Research; 2022 [cited 2023 Mar 31]. Available from: https://f1000research.com/articles/11-1294
22. Altman MC, Rinchai D, Baldwin N, Toufiq M, Whalen E, Garand M, et al. Development of a fixed module repertoire for the analysis and interpretation of blood transcriptome data. Nat Commun. 2021 Jul 19;12(1):4385.
23. 2. Rinchai D, Roelands J, Toufiq M, Hendrickx W, Altman MC, Bedognetti D, et al. BloodGen3Module: Blood transcriptional module repertoire analysis and visualization using R. Bioinforma Oxf Engl. 2021 Feb 24;btab121.
24. 3. Rinchai D, Altman MC, Konza O, Hässler S, Martina F, Toufiq M, et al. Definition of erythroid cell-positive blood transcriptome phenotypes associated with severe respiratory syncytial virus infection. Clin Transl Med. 2020 Dec;10(8):e244.
