# Supplementary material for: Harnessing large language models (LLMs) for candidate gene prioritization and selection
Source: J Transl Med. 2023 Oct 16;21:728. doi: 10.1186/s12967-023-04576-8 (PMC10580627; doi:10.1186/s12967-023-04576-8)
Supplement: Supplementary file 8 — Additional file 8: Is the prioritization report for module M13.30 authored by Mohammed Toufiq. The file is shared in the PDF format. [file 12967_2023_4576_MOESM8_ESM.pdf]

M13.30 Prioritization report

Prepared by: Mohammed Toufiq (The Jackson Laboratory for Genomic Medicine, Farmington, CT, USA)

1. Composition

The module M13.30 belongs to Aggregate A37 from the BloodGen3 fixed module repertoire (1, 2). This module aggregate is associated with circulating erythroid cells. It was found to be associated with RSV disease severity (3). We showed in the same study that the abundance of A27 transcripts is also elevated in the blood of patients with stage IV melanoma (3).

It comprises 29 genes: ABCC13, ADIPOR1, ARL4A, BLVRB, BOAT (ATXN1L), C16ORF35, CARM1, CES3, CSDA, FBXO9, FIS1, FKBP8, FLJ20489, GLUL, GSPT1, GUK1, GYPC, HAGH, HBM, HBQ1, HPS1, PBX1, PTMS, SHARPIN, SLC25A39, ST6GALNAC4, TMEM86B, UBL7, UBXD1.

2. Functional convergence

Functional Themes	Associated Genes	Comments
Protein Modification and Regulation	CARM1, UBL7, PTMS, FBXO9	Genes are involved in protein modification and regulation, such as CARM1 (histone arginine methylation which is important for transcription regulation), UBL7 (ubiquitin-like protein that can be covalently attached to proteins), PTMS (involved in post-translational modification), and FBXO9 (part of the ubiquitin-proteasome system for protein degradation).
Metabolic Processes	ADIPOR1, GLUL, HAGH, GYPC	These genes participate in metabolic processes. For example, ADIPOR1 is implicated in adiponectin signaling and influences glucose levels and fatty acid catabolism, GLUL is part of the urea cycle and glutamate metabolism, HAGH plays a role in detoxification of glyoxals, and GYPC is part of the glycophorin C protein complex involved in the lifespan of red blood cells.
Cellular Degradation and Homeostasis	FBXO9, UBXD1, FIS1, CSDA, FKBP8	These genes have roles in maintaining cellular homeostasis, such as through the involvement in cellular degradation pathways (FBXO9, UBXD1, FIS1), or in protein folding and stability (CSDA, FKBP8).
Transcription Regulation	PBX1, CARM1	PBX1 and CARM1, are involved in the regulation of gene expression.
Mitochondrial Functions	FIS1, SLC25A39	FIS1 is involved in mitochondrial and peroxisomal division, while SLC25A39 is a mitochondrial carrier protein, hinting at a possible convergence in mitochondrial function.

3. Scoring and prioritization

Genes were scored on six criteria using GPT-4 and Claude. The scores were averaged, and candidate genes ranked according to their cumulative scores (**Figure 1**, Methods: Step 3 and Step 4).

The two LLMs were requested to score each gene on the following six statements:
a. The gene is associated with erythroid cells or erythropoiesis.
b. The gene is currently being used as a biomarker in clinical settings.
c. The gene has potential value as a blood transcriptional biomarker.
d. The gene is relevant to circulating leukocytes immune biology.
e. The gene is a known drug target.
f. The gene is therapeutically relevant for immune-mediated diseases.

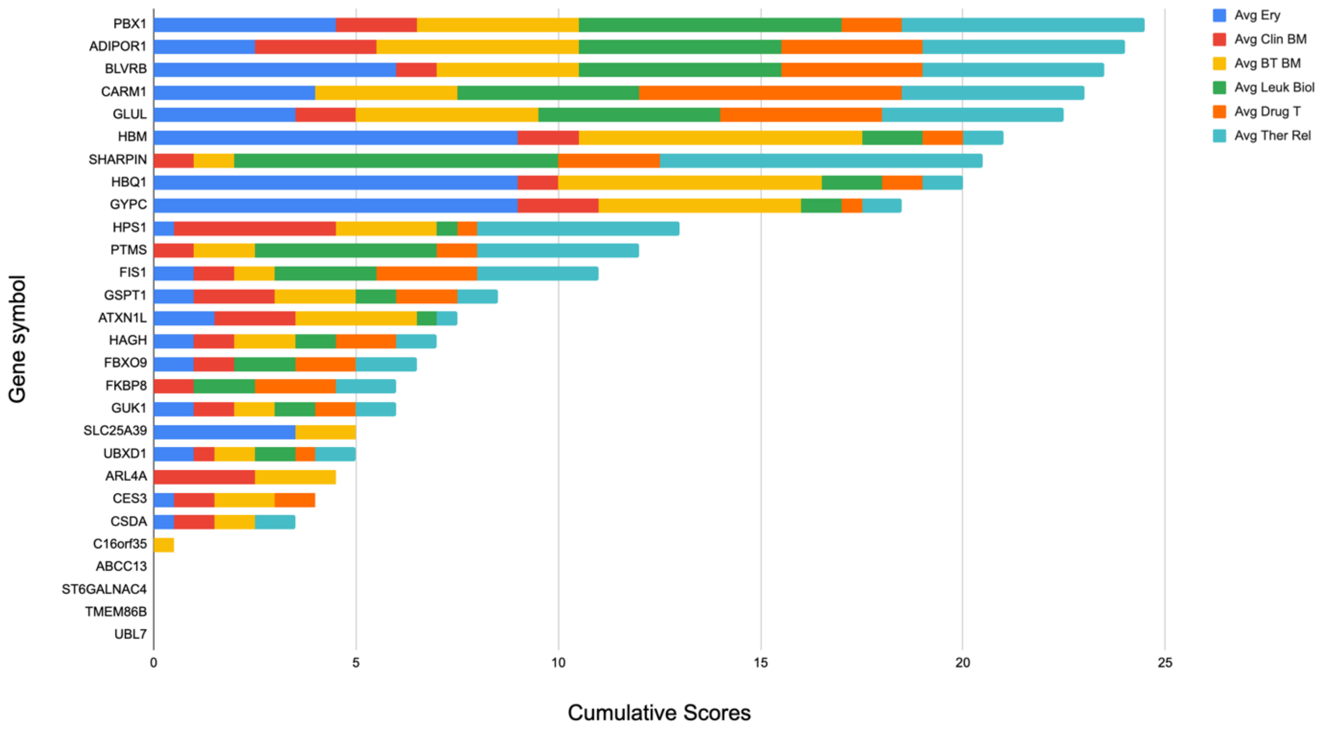

Figure 1: The stacked bar graph shows cumulative scores across six criteria for the 29 M13.30 genes.

The top five genes selected for further evaluation were: PBX1, ADIPOR1, BLVRB, CARM1 and GLUL **(Figure 2**).

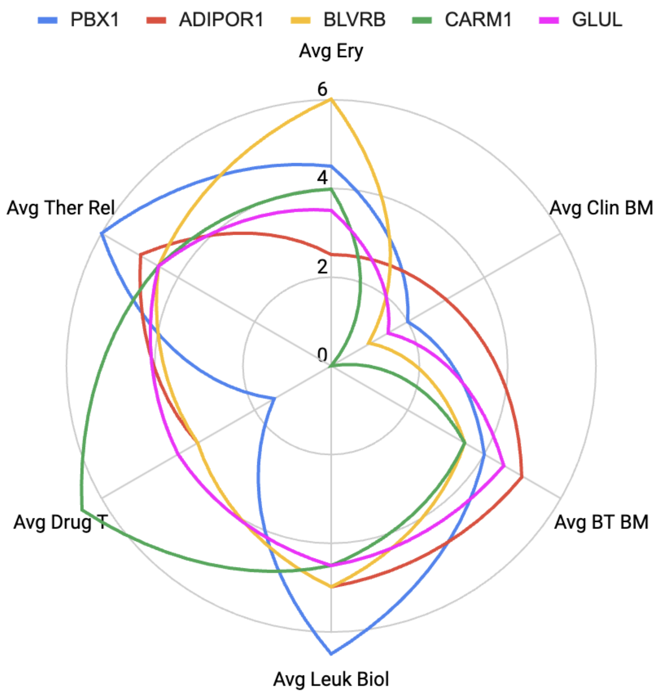

Figure 2: The radar plot represents individual scores for the 5 top scoring genes: PBX1, ADIPOR1, BLVRB, CARM1 and GLUL.

4. Knowledge-driven evaluation of top five candidates

Justifications given by the GPT-4 and Claude for the scores provided across the 6 criteria were compiled and summarized by GPT-4 (Methods: Step 5). For each statement pertinent references were retrieved using GPT-4 or Claude, with the relevance of each reference checked and attributed manually by researcher authoring this report.

Function: "The PBX1, ADIPOR1, BLVRB, CARM1, and GLUL genes encode proteins integral to a variety of biological processes. The PBX1 gene codes for a transcription factor that plays a critical role in embryonic development and cell differentiation, significantly contributing to organogenesis through the regulation of gene expression patterns (4, 5). ADIPOR1 encodes a receptor protein for adiponectin, a hormone essential for glucose levels regulation and fatty acid breakdown. This receptor primarily expresses in skeletal muscle tissue, suggesting its key role in metabolic regulation (6, 7, 8, 9). The BLVRB gene is central to heme metabolism, converting biliverdin into bilirubin, a potent antioxidant, thereby aiding in cellular protection against oxidative stress (10, 11) The protein encoded by the CARM1 gene functions as a histone arginine methyltransferase and a transcriptional coactivator for several nuclear receptors, including the estrogen receptor alpha. This protein contributes significantly to cellular functions such as growth and differentiation (12, 13). Finally, the GLUL gene codes for the Glutamate-Ammonia Ligase enzyme, which is instrumental in nitrogen metabolism by catalyzing the

formation of glutamine from glutamate and ammonia, thereby regulating glutamine metabolism (14)”.

a. Relevance to erythroid cells and erythropoiesis: “Preliminary research indicates potential roles of PBX1, BLVRB, CARM1, ADIPOR1, and GLUL in erythropoiesis, albeit these roles remain largely undefined, highlighting the need for further investigation. PBX1, implicated in hematopoietic development and potentially erythropoiesis, requires additional research to elucidate its precise function in this domain (4, 5). Similarly, BLVRB, crucial in heme catabolism, could play a significant role in erythropoiesis given its importance in erythroid cells, yet the specifics of this association are still to be determined (15,59). The link between CARM1 and erythropoiesis is only suggested by limited evidence, and further exploration is necessary to discern the magnitude and characteristics of its involvement (16). Furthermore, while not traditionally associated with erythropoiesis, initial findings suggest potential roles of ADIPOR1 and GLUL in this process. The precise contributions and involvement of these two genes, however, remain uncertain and require comprehensive substantiation through detailed studies. Thus, the exact nature of these genes' roles and relationships with erythroid cells needs to be accurately defined through rigorous validation”.

b. Is used as a clinical biomarker: “Although preliminary evidence points to potential roles of PBX1, ADIPOR1, BLVRB, CARM1, and GLUL as prognostic markers in various diseases, their widespread adoption as clinical biomarkers remain unrealized as of 2021. PBX1, associated with certain cancers such as breast cancer and leukemia, requires further validation before it can be broadly applied in oncological clinical settings (17, 18). Similarly, ADIPOR1, suggested as a biomarker in metabolic disorders, obesity, and type 2 diabetes, needs thorough validation before routine integration into clinical biomarker panels (19, 20, 21, 22). Despite its hypothetical roles in various biological processes, BLVRB's use as a clinical biomarker is not yet established, highlighting the necessity for further exploration (23). While CARM1 is implicated in numerous cellular processes, its integration as a clinical biomarker is still pending, necessitating additional research (24, 25). Lastly, while studies suggest a potential role for GLUL as a biomarker in certain cancers and liver diseases, it is not yet adopted as a standard clinical biomarker, underscoring the need for further comprehensive validation”.

c. Potential relevance as a blood transcriptional biomarker: “While PBX1, ADIPOR1, BLVRB, CARM1, and GLUL all have established roles in various biological processes, their utilization as blood transcriptional biomarkers is still not definitively established due to limited evidence. PBX1's recognized role in hematopoietic development hints at its potential as a biomarker, but further research and validation are needed (5, 26). Similarly, despite preliminary findings suggesting the utility of ADIPOR1 as a biomarker in metabolic diseases, comprehensive validation is necessary (21). BLVRB's role in heme metabolism could theoretically make it a useful biomarker, but the current evidence supporting this is insufficient, demanding further investigations (27). Although CARM1's function in transcriptional regulation might suggest it as a potential biomarker, definitive proof is still lacking, necessitating further research. Lastly,

despite correlations between GLUL expression and certain diseases, existing evidence for its utility as a transcriptional biomarker is limited, indicating the need for further validation and extensive research (28)”.

d. Relevance to leukocytes immune biology: “PBX1, known for its significant role in hematopoietic development, is intrinsically tied to leukocyte biology, given the critical involvement of leukocytes in hematopoiesis. This association emphasizes PBX1's potential relevance in understanding immune cell functions (5, 29). Although preliminary evidence indicates potential links between ADIPOR1, BLVRB, CARM1, and GLUL and leukocyte biology, these relationships are not definitively established. ADIPOR1's role in regulating inflammatory responses through adiponectin suggests a connection to leukocyte biology, but direct investigations are currently lacking (30, 31, 32). While not directly associated with leukocytes, BLVRB may influence immune cell function indirectly through its end product, bilirubin, known for its immune-modulating properties (33). Similarly, CARM1, acknowledged for its regulatory role in immune responses (34, 35) and GLUL, critical for glutamine synthesis—a crucial nutrient for immune cells—may have implications for leukocyte biology. However, all these potential links require further comprehensive studies and validation for conclusive elucidation”.

e. Is a known drug target: “PBX1, despite its implications in tumorigenesis, is not recognized as a primary drug target, as it lacks qualifying characteristics for direct pharmacological interventions (36, 37). Similarly, ADIPOR1 (8, 38, 39) and BLVRB (40, 41, 42), although investigated for their potential roles in therapeutic interventions for metabolic disorders and various diseases respectively, are not yet considered major drug targets, with no drugs directly targeting these genes available as of 2021. This underscores the need for further research to elucidate their utility in drug development. Contrarily, CARM1, due to its crucial role in cellular growth and differentiation, especially in oncological contexts, is emerging as a potential epigenetic drug target, indicating new directions for therapeutics design (43, 44). Finally, despite investigations into GLUL's potential as a therapeutic target for specific cancers and neurodegenerative disorders, its broad recognition as a direct drug target is still pending as of 2021, highlighting the importance of ongoing research in this area (45)”.

f. Potential therapeutic relevance for immune-mediated diseases: “PBX1, recognized for its role in hematopoietic development and association with hematologic malignancies, particularly leukemias, exhibits potential therapeutic relevance in immune-mediated diseases. However, this potential necessitates further research for full elucidation (5, 46, 47). Similarly, ADIPOR1, the receptor for adiponectin involved in anti-inflammatory processes, suggests therapeutic applicability in immune-mediated diseases, yet requires additional evidence for solid validation (8, 31, 48, 49). BLVRB, contributing to the production of antioxidant and anti-inflammatory bilirubin, also implies potential therapeutic relevance for immune-mediated diseases, but the current evidence is preliminary and inconclusive. Consequently, additional investigation is necessary to establish this connection robustly (10, 50, 51). CARM1, known for its involvement in immune responses, shows potential therapeutic relevance for immune-mediated diseases, although existing research is not conclusive, warranting further

investigation (52, 53). Finally, the GLUL gene, responsible for glutamine synthesis crucial to immune cell function, suggests potential therapeutic applicability for immune-mediated diseases. Despite some initial evidence, the association remains unconfirmed, highlighting the need for further comprehensive studies for validation and exploration of its therapeutic potential (54)".

5. Examining expression patterns of top 5 candidates across leukocyte populations

The expression patterns of the top 5 candidate genes were examined across diverse leukocyte populations and hematopoietic precursors using two reference transcriptomic datasets.

5.1 Densely interconnected transcriptional circuits control cell states in human hematopoiesis – (GSE24759)
http://developmentalimmunology.gxbsidra.org/dm3/geneBrowser/show/4000026

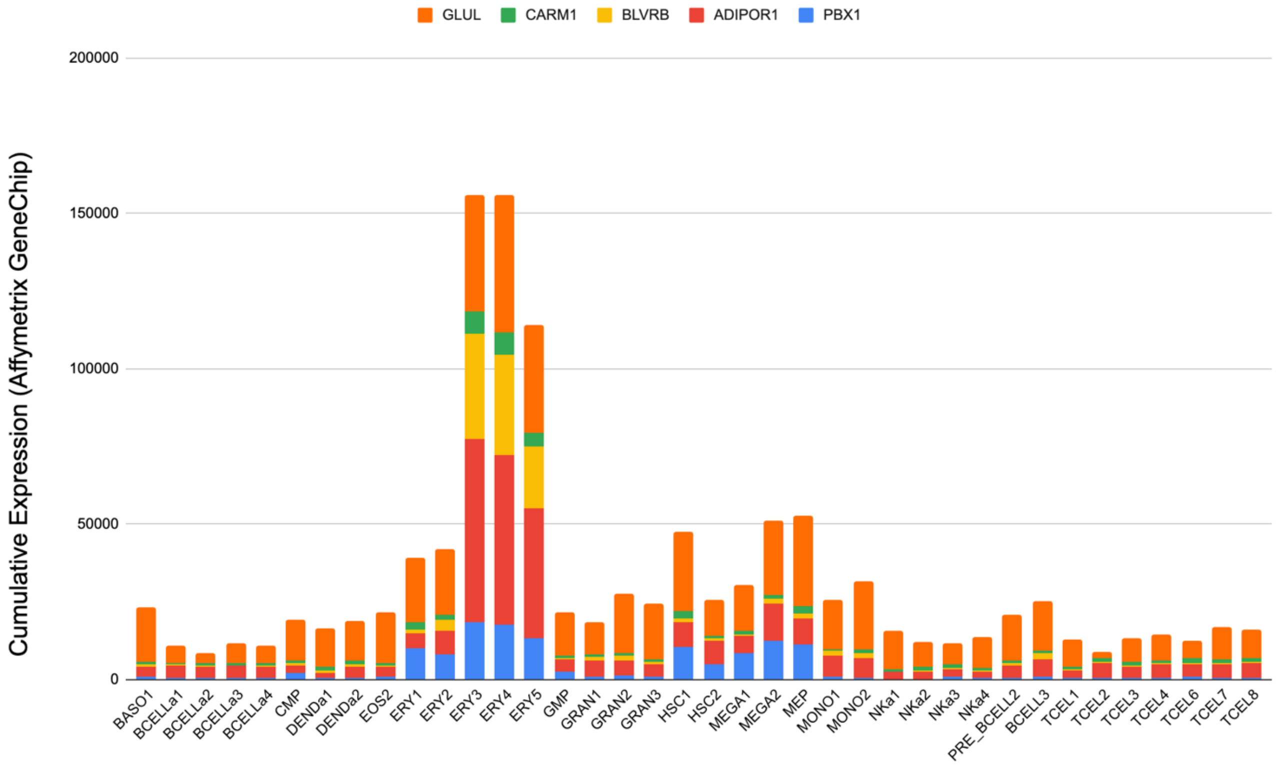

Figure 3: This stacked bar graph shows the expression levels of five candidate genes. ERY3-5 populations are CD71+ GLYA+ erythroid cells. The original article provides full experimental details: (55). The web link above also provides access to more complete information about this dataset as well as individual gene profiles.

5.2 Next generation sequencing of human immune cell subsets across diseases – (GSE60424)
http://cd2k.gxbsidra.org/dm3/geneBrowser/show/4000098

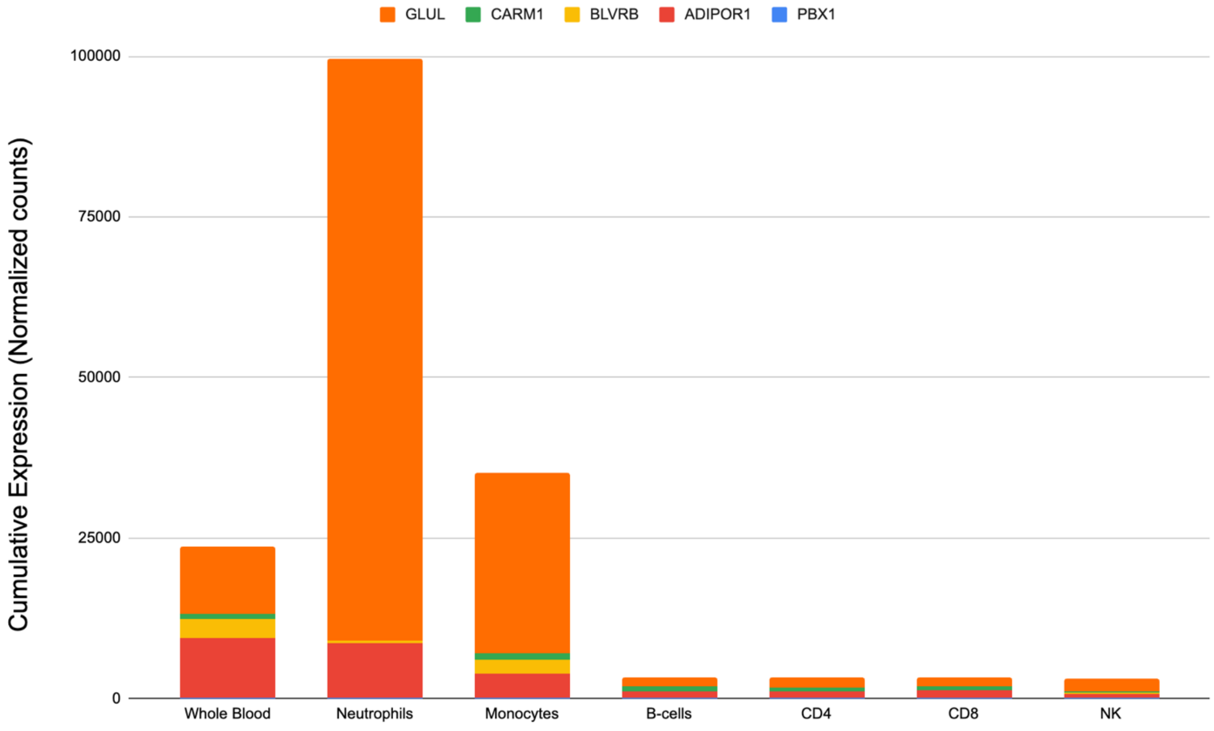

Figure 4: This stacked bar graph shows the expression levels measured by RNAseq of the five candidate genes across six leukocyte populations and whole blood. The abundance is shown for each gene in each population for an average of up to 20 samples. Additional experimental details are accessible via the link provided above and is available as part of this sample series GEO record (GSE60424) (56).

5.3 BloodGen3 reference cohorts (GSE100150)

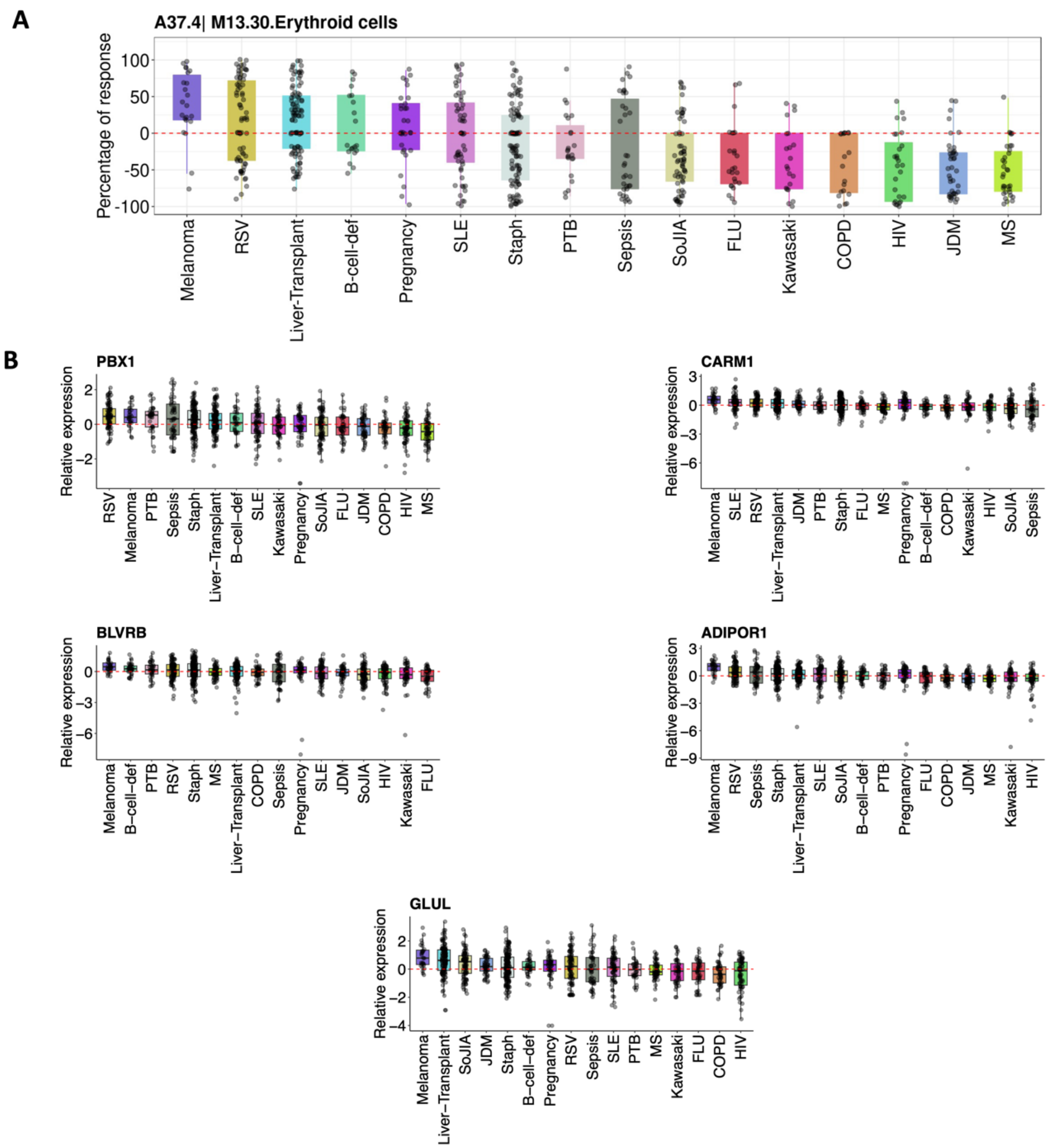

Figure 5: The box plot in panel A shows the changes in abundance of transcripts comprising the M13.30 module, expressed within a cohort as the percentage of genes showing differential expression between each patient and the respective set of control subjects for this cohort. Differential expression is based on fixed cut-off (Log2 Fold Change >1.5, difference>10). These values are expressed as % module activity and can range from 100% (all genes are upregulated) to -100% (all genes are downregulated). The box plot in panel B show expression levels for the top 5 scoring M13.30 genes in patient cohorts relative to respective control subjects matched for demographic variables.

6. Overall pick(s) for M13.30

We aim to establish a universal immune profiling assay that measures transcript abundance of a panel of genes in whole blood using small- or meso-scale molecular profiling technology. Compelling candidates identified here from the M13.30 selection pool will be subjected to more in-depth evaluation using a pre-established workflow (57, 58).

Claude and GPT-4 were prompted to select a top candidate on the basis of the information summarized above in section 4, taking into account all six scoring criteria equally (Methods: Step 7).

<u>Claude chose BLVRB</u>:

Here is a summary of the key conclusions regarding the selection of a candidate gene for inclusion in a targeted blood transcriptional profiling panel:

Initial Summary:

- Based on preliminary evidence, BLVRB was recommended due to its role in heme metabolism and potential relevance to erythropoiesis.

Leukocyte RNA-seq Data:

- RNA-seq data showing highest BLVRB expression in whole blood and monocytes supported its erythroid associations and biomarker potential.

Leukocyte Microarray Data:

- Microarray data revealed high BLVRB expression specifically in erythroid precursor cells, confirming its erythroid specificity.

Patient Cohort RNA Abundance Data:

- Moderate decreases in BLVRB RNA levels across immune-related disease states provided further evidence for its usefulness as a broad biomarker reflecting immune activity.

In summary, BLVRB was consistently recommended throughout the process based on multiple lines of evidence indicating its high expression in erythroid lineages and involvement in heme metabolism, along with detectable expression changes in immune cell samples and disease

cohorts. The data sources all strengthen the case for BLVRB as a top candidate gene meeting both the erythroid and immune biology goals for the targeted blood profiling panel.

<u>GPT-4 chose PBX1</u>:

Certainly! Here are the key conclusions we can draw from the data and conversation so far:

1. Gene Overview:
    - PBX1 is associated with the formation and function of blood cells.
    - ADIPOR1 is linked with metabolism and has a role in immune function.
    - BLVRB is involved in cell proliferation, differentiation, and apoptosis, with specific importance in early erythroid differentiation.
    - CARM1 is linked with transcriptional regulation, cell growth, and apoptosis.
    - GLUL plays a role in nitrogen metabolism and is also crucial for the brain's neurotransmission.

2. RNA Abundance in Different Conditions:
    - For PBX1: Elevated RNA abundance in conditions like B-cell deficiency, metastatic melanoma, active pulmonary tuberculosis, acute respiratory syncytial virus infection, and sepsis. Decreased RNA abundance is seen in conditions such as chronic obstructive pulmonary disease, acute influenza, HIV infection, juvenile dermatomyositis, and multiple sclerosis.
    - For ADIPOR1: Elevated RNA abundance in metastatic melanoma and acute respiratory syncytial virus infection. A significant decrease is observed in HIV infection and Kawasaki disease.
    - For BLVRB: Elevated RNA abundance in conditions like B-cell deficiency and metastatic melanoma. Significant decrease is seen in acute influenza, HIV infection, and Kawasaki disease.
    - For CARM1: Elevated RNA abundance in conditions like juvenile dermatomyositis and metastatic melanoma. A decrease is seen in conditions such as HIV infection, Kawasaki disease, and sepsis.
    - For GLUL: Elevated RNA abundance in conditions like juvenile dermatomyositis. A significant decrease is observed in HIV infection.

3. General Observations:
    - Most genes show variable RNA abundance patterns across different conditions, implying that their expression is modulated in response to various health states.
    - Diseases like HIV infection often result in decreased RNA abundance across multiple genes, suggesting a broad impact on transcriptional activity.
    - Some genes like CARM1 and ADIPOR1 have increased expression in specific conditions like metastatic melanoma, hinting at possible roles in disease pathology or as potential biomarkers.

Recommendation for Top Candidate:

Based on the summarized information, if the goal is to select a gene that is most responsive across a wide range of conditions (both upregulated and downregulated), **PBX1** stands out. It shows a broad range of responses, being affected in conditions like B-cell deficiency, metastatic melanoma, HIV infection, and more. This variability suggests that PBX1 might be involved in a range of physiological and pathological processes, making it a valuable inclusion in a targeted blood transcriptional profiling panel. However, the final decision should also consider the specific goals of the panel and the diseases or conditions it aims to target.

This summary provides an overview of the data presented and its potential implications. Further research and validations would be essential to establish any clinical significance.

Given its comparatively higher expression levels and restriction across leukocyte populations, the top candidate for this module is PBX1. Considering this, in conjunction with the rationale provided by both LLMs and our understanding of BLVRB and PBX1 biological and clinical relevance, we identify PBX1 as our primary M13.30 selection for subsequent characterization.

**Prompts**	**CLAUDE**	**GPT-4**
PROMPT 1: Based on the summary below, please recommend the top candidate gene to include in a targeted blood transcriptional profiling panel. The intent is for the selected gene to be representative of an erythroid cell signature associated with response to mRNA vaccines, severity of RSV infection and that found to be expressed at high levels in patients with metastatic melanoma and in liver transplant recipients. Please weigh the following criteria equally in your recommendation: 1) Relevance to erythroid cells and erythropoiesis, 2) Current use as a biomarker, 3) Potential use as a blood transcriptional biomarker, 4) Relevance to leukocyte immune biology, 5) Status as a known drug target, 6) Therapeutic relevance for immune mediated diseases: *[input summarized and fact-checked justifications from Step 5]*	Based on the provided summary and criteria, I would recommend BLVRB as the top candidate gene to include in the targeted blood transcriptional profiling panel.<br>The key reasons are:<br>• BLVRB plays a significant role in heme metabolism, which is intrinsically important for erythropoiesis and erythroid cell biology. This aligns closely with the criterion for erythroid relevance.<br>• BLVRB may have some relevance to immune cell biology through the immune-modulating effects of its product bilirubin.<br>• Though not an established biomarker yet, BLVRB's involvement in heme metabolism suggests potential utility as a blood transcriptional biomarker.	Given the detailed summary provided on the five candidate genes (PBX1, ADIPOR1, BLVRB, CARM1, and GLUL) and the six criteria for evaluation, we can rank the genes based on their relevance and potential as a targeted blood transcriptional profiling panel marker.<br><br>1. Relevance to erythroid cells and erythropoiesis:<br>• PBX1 has implications in hematopoietic development and potentially erythropoiesis.<br>• BLVRB is crucial in heme catabolism and might have significance in erythroid cells.<br>• CARM1's link to erythropoiesis is suggested but

	- The other genes have more indirect links to erythropoiesis (PBX1, CARM1) or no erythroid associations (ADIPOR1, GLUL). - BLVRB is not currently a drug target, but has implications in diseases like melanoma and for transplant recipients through its antioxidant bilirubin. In summary, BLVRB stands out as the top choice because of its specific role in heme metabolism, which is intrinsically tied to erythropoiesis, as well as its antioxidant and immune-modulating functions. The other genes are weaker fits for the stated criteria. Additional research can further confirm BLVRB's utility as a biomarker and relevance to immune biology and disease.	not strongly established. - ADIPOR1 and GLUL have preliminary evidence suggesting a role in erythropoiesis but require more research. 2. Current use as a biomarker: - None of the genes are established as widespread clinical biomarkers by 2021. 3. Potential use as a blood transcriptional biomarker: - PBX1's role in hematopoietic development might make it a potential biomarker. - BLVRB due to its role in heme metabolism might be a biomarker but needs further validation. - ADIPOR1, CARM1, and GLUL all need more validation to establish as blood transcriptional biomarkers. 4. Relevance to leukocyte immune biology: - PBX1 has significant relevance due to its role in hematopoietic development. - ADIPOR1 has a potential

		connection due to its role in regulating inflammatory responses.<br>• BLVRB may have an indirect effect via bilirubin's immune-modulating properties.<br>• CARM1 and GLUL both have some potential relevance but need more studies for clarification.<br>5. Status as a known drug target:<br>• CARM1 is emerging as a potential epigenetic drug target due to its significant role in cellular functions.<br>• PBX1, ADIPOR1, BLVRB, and GLUL have not been established as major drug targets as of 2021.<br>6. Therapeutic relevance for immune mediated diseases:<br>• PBX1 has potential therapeutic relevance due to its associations with hematologic malignancies.<br>• ADIPOR1 is involved in anti-inflammatory processes and might have therapeutic relevance.<br>• BLVRB contributes to antioxidant production and might have potential relevance.<br>• CARM1's involvement in immune responses

		suggests some therapeutic potential.<br>• GLUL's role in glutamine synthesis suggests potential applicability but needs more validation.<br><br>Given the above analysis:<br>• PBX1 scores high in relevance to erythroid cells and hematopoiesis, potential use as a blood transcriptional biomarker, relevance to leukocyte immune biology, and potential therapeutic relevance for immune-mediated diseases.<br>• CARM1 is noted for its emergence as a drug target and potential therapeutic relevance for immune-mediated diseases.<br>• BLVRB has potential due to its role in heme catabolism and erythropoiesis.<br>• ADIPOR1 and GLUL seem to have less pronounced evidence in the described categories but might still be relevant in some contexts.<br><br>Recommendation: Given the evaluation criteria, PBX1 seems to be the top candidate gene to include in a targeted blood transcriptional profiling panel. It appears to be the most relevant for erythroid cells, has ties to leukocyte biology, and holds potential therapeutic relevance. However, it's crucial to note that as the roles of many of these genes are not yet conclusively defined, continuous research and re-evaluation are essential.

<table>
<tr>
<td>PROMP 2: Now that you have provided an initial recommendation based on the summary, take into account the following RNA-sequencing expression data from key immune cell types and whole blood samples: [input textualized transcriptional data]. Given this additional RNA-seq expression data, does your recommended gene selection remain the same? Please explain which gene you would recommend at this stage and why, incorporating both the summarized information provided earlier and RNA-seq data in your rationale."</td>
<td>After reviewing the additional RNA-seq expression data, I would maintain my original recommendation of BLVRB as the top candidate gene.

The key reasons are:
The RNA-seq data shows BLVRB expression primarily in whole blood and monocytes, aligning with its erythroid and heme metabolism relevance.
In contrast, the other genes like PBX1, ADIPOR1, CARM1, and GLUL exhibit broader expression across immune cells, lacking erythroid specificity.
While BLVRB expression is low in most immune cells, its detection in whole blood supports its potential as a blood transcriptional biomarker.
The expression patterns reinforce the summary indicating BLVRB's erythroid associations versus more systemic roles for the other genes.
In summary, the RNA-seq data provides supporting evidence that BLVRB has highest expression in cell types related to erythropoiesis and heme metabolism, which was the rationale for originally selecting it. The additional data is consistent with the summary information pointing to BLVRB as the top candidate for meeting the aims of an erythroid-focused blood transcriptional biomarker.</td>
<td>Let's begin by summarizing the RNA-seq expression data for each gene across the various immune cells and whole blood samples:

PBX1:
Highest expression in Whole Blood (10), followed by Neutrophils (6), with moderate to low expression across other immune cells.
Average across all samples: ~5.14
2. ADIPOR1:
Highest expression in Whole Blood (462) and Neutrophils (429), followed by Monocytes (189). Very low expression in specific immune cells such as B-cells (54) and T-cells (51 for CD4 and 61 for CD8).
Average across all samples: ~159.9
3. BLVRB:
Highest expression in Whole Blood (145) and Monocytes (110). Very low expression in specific immune cells like Neutrophils (13), B-cells (3), and T-cells (3 for both CD4 and CD8).
Average across all samples: ~39.7
4. CARM1:
Relatively uniform expression across different immune cells with highest expression in Monocytes (49) and lowest in Neutrophils (5).
Average across all samples: ~30
5. GLUL:
Highest expression in Neutrophils (4524),</td>
</tr>
</table>

		followed by Monocytes (1410) and Whole Blood (525). Specific immune cells like B-cells (68) and T-cells (73 for CD4 and 70 for CD8) show moderate expression.<br>• Average across all samples: ~964.57<br><br>Considering the RNA-seq data, GLUL shows the highest average expression across all immune cells and whole blood, making it a prime candidate for blood transcriptional profiling. The significantly high expression of GLUL in neutrophils also supports its relevance in leukocyte biology, satisfying criterion 4.<br>Let's now incorporate the summarized information from earlier:<br><br>1. Relevance to erythroid cells and erythropoiesis: From the earlier information, all genes have potential relevance, but PBX1 and BLVRB seemed slightly more directly associated. However, this wasn't conclusive.<br><br>2. Current use as a biomarker: As of 2021, none of the genes were extensively adopted as clinical biomarkers, although there was some preliminary evidence pointing towards their potential roles.<br><br>3. Potential use as a blood transcriptional biomarker: Given the RNA-seq data, GLUL has a strong signal, especially in neutrophils, which indicates its potential as a blood transcriptional biomarker.<br><br>4. Relevance to leukocyte immune biology: PBX1 has intrinsic ties to leukocyte biology, but the high

		expression of GLUL, particularly in neutrophils, makes it relevant too.<br><br>5. Status as a known drug target: CARM1 was noted to be emerging as an epigenetic drug target. GLUL had potential for specific cancers and neurodegenerative disorders.<br><br>6. Therapeutic relevance for immune-mediated diseases: Both GLUL and CARM1 had potential therapeutic applicability for immune-mediated diseases.<br><br>Given the combination of the RNA-seq data and the initial summary, while PBX1 and BLVRB had some promising connections to erythroid cells, GLUL stands out due to its:<br><br>1. High expression levels, especially in neutrophils, suggesting its potential use as a blood transcriptional biomarker.<br>2. Relevance to leukocyte biology.<br>3. Therapeutic relevance for immune-mediated diseases.<br>Recommendation: I would recommend GLUL as the top candidate gene to include in a targeted blood transcriptional profiling panel, especially considering its high expression in neutrophils and monocytes, and its potential therapeutic relevance.
PROMPT 3: Now that you have provided an initial recommendation based on the summary and the RNAseq data, take into account the following microarray expression data from key immune cell types and hematopoietic precursors: *[input textualized transcriptional data]*. Given this additional microarray expression data, does your recommended gene selection remain the same? Please explain which gene you would recommend at this stage and why, incorporating the summarized information and RNA-seq data provided earlier, as	After reviewing the microarray expression data, I would maintain my recommendation of BLVRB as the top candidate gene.<br><br>The key reasons are:<br><br>• The microarray data shows the highest expression of BLVRB in erythroid precursor cells, aligning with its role in heme metabolism and erythropoiesis.<br>• BLVRB exhibits more modest expression across	*[N/A: input exceed GPT-4 limits. Attempts to break down inputting the textualized data were unsuccessful as GPT-4 lost the thread of the conversation. Thus, we chose to skip to the next prompt]*

well as this new microarray data in your rationale.	leukocytes and immune cell types, supporting its potential as a blood transcriptional biomarker.<br>• In contrast, other genes like PBX1, ADIPOR1 and GLUL lack erythroid-specificity based on their broad expression profiles.<br>• While CARM1 has some erythroid expression, it is lower than BLVRB in those precursor cells.<br>• The microarray data reinforces the RNA-seq patterns indicating the erythroid specificity of BLVRB versus systemic roles for the other genes.<br><br>In summary, the addition of the microarray expression data provides further evidence that BLVRB has the best expression profile for an erythroid-focused blood transcriptional biomarker. The high erythroid and modest immune cell expression fits the intended purpose. Therefore, BLVRB remains the top choice after incorporating the new microarray information.	
PROMPT 4: Now that you have provided an initial recommendation based on the summary and the immune cells RNAseq and microarray data, take into account the following averaged log2 fold changes in RNA abundance in patient cohorts compared to controls. *[input textualized transcriptional data].* Given this additional data, does your recommended gene selection remain the same? Please explain which gene you would recommend at this stage and why, incorporating the summarized information, the leukocytes RNA-seq and microarray data provided earlier, as well as these new patient cohort profiles in your rationale.	After reviewing the RNA abundance log fold change data for the various patient cohorts, I would maintain my recommendation of BLVRB as the top candidate gene.<br><br>The key reasons are:<br><br>• BLVRB showed the most consistent decreases in patients with conditions like influenza, HIV, and juvenile arthritis, implying it positively correlates with immune activity.<br>• The decreases were moderate overall (in -0.2 to -0.8 range), indicating BLVRB levels change but are not radically altered.	This is a substantial amount of data, but let's break it down step by step.<br><br>1. PBX1 RNA Abundance:<br><br>• Increased in: B-cell deficiency, metastatic melanoma, active pulmonary tuberculosis, acute respiratory syncytial virus infection, sepsis, and liver transplant recipients under maintenance therapy.<br>• Decreased in: COPD, acute influenza, HIV, juvenile dermatomyositis, Kawasaki disease, multiple sclerosis, pregnancy,

	• The other genes had more variable patterns of increase/decrease across cohorts.<br>• BLVRB's association with conditions like melanoma fits with its known biology.<br>• The cohort patterns align with the intended use of BLVRB as a biomarker reflecting immune shifts.<br><br>In summary, the cohort data provides additional evidence that BLVRB expression exhibits moderate but detectable changes in diverse immune-related conditions. This fits well with the goals of a broad blood transcriptional biomarker. The consistency and moderation of BLVRB changes make it a better fit as a biomarker gene than the other variable candidates. Thus, I continue to recommend BLVRB as the top choice based on the full body of provided data.	systemic onset juvenile idiopathic arthritis.<br>• Marginal/Near Neutral Change: systemic lupus erythematosus.<br><br>2. ADIPOR1 RNA Abundance:<br><br>• Increased in: metastatic melanoma, acute respiratory syncytial virus infection, sepsis, systemic lupus erythematosus, systemic onset juvenile idiopathic arthritis, staphylococcus aureus infection, and liver transplant recipients under maintenance therapy.<br>• Decreased in: COPD, acute influenza, HIV, juvenile dermatomyositis, Kawasaki disease, multiple sclerosis, pregnancy, active pulmonary tuberculosis.<br>• Marginal/Near Neutral Change: B-cell deficiency.<br><br>3. BLVRB RNA Abundance:<br><br>• Increased in: B-cell deficiency, metastatic melanoma, active pulmonary tuberculosis, and acute respiratory syncytial virus infection.<br>• Decreased in: COPD, acute influenza, HIV, juvenile dermatomyositis, Kawasaki disease, multiple sclerosis, pregnancy, sepsis, systemic lupus erythematosus, systemic onset juvenile idiopathic arthritis, and liver transplant recipients under maintenance therapy.

		• Marginal/Near Neutral Change: staphylococcus aureus infection.<br><br>4. CARM1 RNA Abundance:<br><br>• Increased in: juvenile dermatomyositis, metastatic melanoma, acute respiratory syncytial virus infection, and liver transplant recipients under maintenance therapy.<br>• Decreased in: B-cell deficiency, COPD, acute influenza, HIV, Kawasaki disease, multiple sclerosis, pregnancy, sepsis, and systemic onset juvenile idiopathic arthritis.<br>• Marginal/Near Neutral Change: active pulmonary tuberculosis, systemic lupus erythematosus, and staphylococcus aureus infection.<br><br>5. GLUL RNA Abundance:<br><br>• Your message got cut off after the part where it mentioned that the GLUL RNA in juvenile dermatomyositis increased. Thus, I'm unable to evaluate the entire set of conditions in relation to GLUL.<br><br>Given this analysis, the following patterns can be noticed:<br><br>• PBX1 has a mixed expression pattern across the conditions, but it is interesting to see the increased abundance in conditions such as sepsis, acute respiratory syncytial virus infection, and active pulmonary tuberculosis.

		• ADIPOR1 has notably increased expression in metastatic melanoma, and a significant decrease in HIV patients.<br>• BLVRB generally has a decreased expression in most conditions, but an increased expression in conditions like metastatic melanoma.<br>• CARM1 has an increased expression in conditions such as metastatic melanoma and acute respiratory syncytial virus infection, with a decrease in many other conditions.<br><br>Given the expression patterns, certain conditions and genes appear to correlate in terms of increased or decreased abundance. This might provide hints towards potential pathways and mechanisms that might be altered in these conditions.<br>For a full understanding, it would be essential to perform pathway analyses and functional annotations to see how these genes might be affecting various cellular processes. It would also be of value to determine the statistical significance of these changes, the sample size, and if any other clinical or experimental factors might influence these readings.
PROMPT 5: Could you please summarize the key conclusions you have drawn from the conversation so far?	Here is a summary of the key conclusions regarding the selection of a candidate gene for inclusion in a targeted blood transcriptional profiling panel:<br><br>Initial Summary:<br>• Based on preliminary evidence, BLVRB was recommended due to its role in heme metabolism and potential relevance to erythropoiesis.	Certainly! Here are the key conclusions we can draw from the data and conversation so far:<br><br>1. Gene Overview:<br>• PBX1 is associated with the formation and function of blood cells.<br>• ADIPOR1 is linked with metabolism and has a role in immune function.<br>• BLVRB is involved in cell proliferation, differentiation, and

	Leukocyte RNA-seq Data:<br>• RNA-seq data showing highest BLVRB expression in whole blood and monocytes supported its erythroid associations and biomarker potential.<br><br>Leukocyte Microarray Data:<br>• Microarray data revealed high BLVRB expression specifically in erythroid precursor cells, confirming its erythroid specificity.<br><br>Patient Cohort RNA Abundance Data:<br><br>• Moderate decreases in BLVRB RNA levels across immune-related disease states provided further evidence for its usefulness as a broad biomarker reflecting immune activity.<br><br>In summary, BLVRB was consistently recommended throughout the process based on multiple lines of evidence indicating its high expression in erythroid lineages and involvement in heme metabolism, along with detectable expression changes in immune cell samples and disease cohorts. The data sources all strengthen the case for BLVRB as a top candidate gene meeting both the erythroid and immune biology goals for the targeted blood profiling panel.	apoptosis, with specific importance in early erythroid differentiation.<br>• CARM1 is linked with transcriptional regulation, cell growth, and apoptosis.<br>• GLUL plays a role in nitrogen metabolism and is also crucial for the brain's neurotransmission.<br><br>2. RNA Abundance in Different Conditions:<br><br>• For PBX1: Elevated RNA abundance in conditions like B-cell deficiency, metastatic melanoma, active pulmonary tuberculosis, acute respiratory syncytial virus infection, and sepsis. Decreased RNA abundance is seen in conditions such as chronic obstructive pulmonary disease, acute influenza, HIV infection, juvenile dermatomyositis, and multiple sclerosis.<br>• For ADIPOR1: Elevated RNA abundance in metastatic melanoma and acute respiratory syncytial virus infection. A significant decrease is observed in HIV infection and Kawasaki disease.<br>• For BLVRB: Elevated RNA abundance in conditions like B-cell deficiency and metastatic melanoma. Significant decrease is seen in acute influenza, HIV infection, and Kawasaki disease.<br>• For CARM1: Elevated RNA abundance in conditions like juvenile dermatomyositis and metastatic melanoma. A decrease is seen in conditions such as HIV

		infection, Kawasaki disease, and sepsis.<br>• For GLUL: Elevated RNA abundance in conditions like juvenile dermatomyositis. A significant decrease is observed in HIV infection.<br><br>3. General Observations:<br><br>• Most genes show variable RNA abundance patterns across different conditions, implying that their expression is modulated in response to various health states.<br>• Diseases like HIV infection often result in decreased RNA abundance across multiple genes, suggesting a broad impact on transcriptional activity.<br>• Some genes like CARM1 and ADIPOR1 have increased expression in specific conditions like metastatic melanoma, hinting at possible roles in disease pathology or as potential biomarkers.<br><br>Recommendation for Top Candidate:<br><br>• Based on the summarized information, if the goal is to select a gene that is most responsive across a wide range of conditions (both upregulated and downregulated), PBX1 stands out. It shows a broad range of responses, being affected in conditions like B-cell deficiency, metastatic melanoma, HIV infection, and more. This variability suggests that PBX1 might be involved in a range of

		physiological and pathological processes, making it a valuable inclusion in a targeted blood transcriptional profiling panel. However, the final decision should also consider the specific goals of the panel and the diseases or conditions it aims to target.<br><br>This summary provides an overview of the data presented and its potential implications. Further research and validations would be essential to establish any clinical significance.

7. References

1. Altman, M.C. *et al*. Development of a fixed module repertoire for the analysis and interpretation of blood transcriptome data. Nat Commun. 2021 Jul 19;12(1):4385.

2. Rinchai, D. *et al*. BloodGen3Module: Blood transcriptional module repertoire analysis and visualization using R. Bioinformatics. 2021 Aug 25;37(16):2382-2389.

3. Rinchai, D. *et al*. Definition of erythroid cell-positive blood transcriptome phenotypes associated with severe respiratory syncytial virus infection. Clin Transl Med. 2020 Dec;10(8):e244.

4. Selleri, L. *et al*. Requirement for Pbx1 in skeletal patterning and programming chondrocyte proliferation and differentiation. Development. 2001 Sep;128(18):3543-57.

5. DiMartino, J.F. *et al*. The Hox cofactor and proto-oncogene Pbx1 is required for maintenance of definitive hematopoiesis in the fetal liver. Blood. 2001 Aug 1;98(3):618-26.

6. Yamauchi, T. *et al*. Adiponectin stimulates glucose utilization and fatty-acid oxidation by activating AMP-activated protein kinase. Nat Med. 2002 Nov;8(11):1288-95.

7. Iwabu, M. *et al*. Adiponectin and AdipoR1 regulate PGC-1alpha and mitochondria by Ca(2+) and AMPK/SIRT1. Nature. 2010 Apr 29;464(7293):1313-9.

8. Yamauchi, T. *et al*. Targeted disruption of AdipoR1 and AdipoR2 causes abrogation of adiponectin binding and metabolic actions. Nat Med. 2007 Mar;13(3):332-9.

9. Yamauchi, T. *et al*. Cloning of adiponectin receptors that mediate antidiabetic metabolic effects. Nature. 2003 Jun 12;423(6941):762-9.

10. Kapitulnik, J. *et al*. Pleiotropic functions of biliverdin reductase: cellular signaling and generation of cytoprotective and cytotoxic bilirubin. Trends Pharmacol Sci. 2009 Mar;30(3):129-37.

11. Sedlak, T.W. *et al*. Bilirubin benefits: cellular protection by a biliverdin reductase antioxidant cycle. Pediatrics. 2004 Jun;113(6):1776-82.

12. Lee, Y.H *et al*. Minireview: protein arginine methylation of nonhistone proteins in transcriptional regulation. Mol Endocrinol. 2009 Apr;23(4):425-33.

13. Bedford, M.T. *et al*. Protein arginine methylation in mammals: who, what, and why. Mol Cell. 2009 Jan 16;33(1):1-13.

14. Krajewski, W.W. et al. Crystal structures of mammalian glutamine synthetases illustrate substrate-induced conformational changes and provide opportunities for drug and herbicide design. J Mol Biol. 2008 Jan 4;375(1):217-28.

15. Pereira, P.J. et al. Structure of human biliverdin IXbeta reductase, an early fetal bilirubin IXbeta producing enzyme. Nat Struct Biol. 2001 Mar;8(3):215-20.

16. Greenblatt, S.M. et al. CARM1 Is Essential for Myeloid Leukemogenesis but Dispensable for Normal Hematopoiesis. Cancer Cell. 2018 Jun 11;33(6):1111-1127.e5.

17. Liu, X.M. et al. PBX1 attributes as a determinant of connexin 32 downregulation in Helicobacter pylori-related gastric carcinogenesis. World J Gastroenterol. 2017 Aug 7;23(29):5345-5355.

18. Alharbi, R.A. et al. The role of HOX genes in normal hematopoiesis and acute leukemia. Leukemia. 2013 Apr;27(5):1000-8.

19. Byeon, J.S. et al. Adiponectin and adiponectin receptor in relation to colorectal cancer progression. Int J Cancer. 2010 Dec 15;127(12):2758-67.

20. Yoneda, K. et al. Expression of adiponectin receptors, AdipoR1 and AdipoR2, in normal colon epithelium and colon cancer tissue. Oncol Rep. 2008 Sep;20(3):479-83.

21. Kang, E.S. et al. Variants of the adiponectin and adiponectin receptor-1 genes and posttransplantation diabetes mellitus in renal allograft recipients. J Clin Endocrinol Metab. 2012 Jan;97(1):E129-35.

22. Wang, H. et al. Adiponectin receptor 1 gene (ADIPOR1) as a candidate for type 2 diabetes and insulin resistance. Diabetes. 2004 Aug;53(8):2132-6.

23. Mueller, C. et al. The heme degradation pathway is a promising serum biomarker source for the early detection of Alzheimer's disease. J Alzheimers Dis. 2010;19(3):1081-91.

24. Elakoum, R. et al. CARM1 and PRMT1 are dysregulated in lung cancer without hierarchical features. Biochimie. 2014 Feb;97:210-8.

25. Wu, D. et al. CARM1 promotes non-small cell lung cancer progression through upregulating CCNE2 expression. Aging (Albany NY). 2020 Jun 2;12(11):10578-10593.

26. Rosa, P.D. et al. The homeodomain transcription factor Prep1 (pKnox1) is required for hematopoietic stem and progenitor cell activity. Dev Biol. 2007 Nov 15;311(2):324-34.

27. Overhaus, M. et al. Biliverdin protects against polymicrobial sepsis by modulating inflammatory mediators. Am J Physiol Gastrointest Liver Physiol. 2006 Apr;290(4):G695-703.

28. Halama, Al. et al. Advancing Cancer Treatment by Targeting Glutamine Metabolism-A Roadmap. Cancers (Basel). 2022 Jan 22;14(3):553.

29. Ficara, F. et al. Pbx1 regulates self-renewal of long-term hematopoietic stem cells by maintaining their quiescence. Cell Stem Cell. 2008 May 8;2(5):484-96.

30. Luo, Y. et al. Adiponectin: a versatile player of innate immunity. J Mol Cell Biol. 2016 Apr;8(2):120-8.

31. Peng, Y.J. et al. Adiponectin and adiponectin receptor 1 overexpression enhance inflammatory bowel disease. J Biomed Sci. 2018 Mar 14;25(1):24.

32. Takemura, Y. et al. Adiponectin modulates inflammatory reactions via calreticulin receptor-dependent clearance of early apoptotic bodies. J Clin Invest. 2007 Feb;117(2):375-86.

33. Boon, A.C. et al. Circulating bilirubin and defense against kidney disease and cardiovascular mortality: mechanisms contributing to protection in clinical investigations. Am J Physiol Renal Physiol. 2014 Jul 15;307(2):F123-36.

34. Srour, N. et al. The Influence of Arginine Methylation in Immunity and Inflammation. J Inflamm Res. 2022 May 13;15:2939-2958.

35. Kumar, S. et al. CARM1 Inhibition Enables Immunotherapy of Resistant Tumors by Dual Action on Tumor Cells and T Cells. Cancer Discov. 2021 Aug;11(8):2050-2071.

36. Morgan, R. et al. Targeting HOX/PBX dimers in cancer. Oncotarget. 2017 May 9;8(19):32322-32331.

37. Shah, N. et al. The Hox genes and their roles in oncogenesis. Nat Rev Cancer. 2010 May;10(5):361-71.

38. Okada-Iwabu, M. et al. A small-molecule AdipoR agonist for type 2 diabetes and short life in obesity. Nature. 2013 Nov 28;503(7477):493-9.

39. Holland, W.L. et al. Receptor-mediated activation of ceramidase activity initiates the pleiotropic actions of adiponectin. Nat Med. 2011 Jan;17(1):55-63.

40. Maines, M.D. New insights into biliverdin reductase functions: linking heme metabolism to cell signaling. Physiology (Bethesda). 2005 Dec;20:382-9.

41. Barone, E. et al. The Janus face of the heme oxygenase/biliverdin reductase system in Alzheimer disease: it's time for reconciliation. Neurobiol Dis. 2014 Feb;62:144-59.

42. Gibbs, P.E.M. et al. Biliverdin reductase: more than a namesake - the reductase, its Peptide fragments, and biliverdin regulate activity of the three classes of protein kinase C. Front Pharmacol. 2012 Mar 13;3:31.

43. Chen, D. et al. Regulation of transcription by a protein methyltransferase. Science. 1999 Jun 25;284(5423):2174-7.

44. Bedford, M.T. et al. Arginine methylation at a glance. J Cell Sci. 2007 Dec 15;120(Pt 24):4243-6.

45. Curi, R. et al. Molecular mechanisms of glutamine action. J Cell Physiol. 2005 Aug;204(2):392-401.

46. Ao, X. et al. PBX1 is a valuable prognostic biomarker for patients with breast cancer. Exp Ther Med. 2020 Jul;20(1):385-394.

47. Kamps, M.P. et al. The human t(1;19) translocation in pre-B ALL produces multiple nuclear E2A-Pbx1 fusion proteins with differing transforming potentials. Genes Dev. 1991 Mar;5(3):358-68.

48. Frommer, K.W. et al. Adiponectin-mediated changes in effector cells involved in the pathophysiology of rheumatoid arthritis. Arthritis Rheum. 2010 Oct;62(10):2886-99.

49. Ohashi, K. et al. Adiponectin promotes macrophage polarization toward an anti-inflammatory phenotype. J Biol Chem. 2010 Feb 26;285(9):6153-60.

50. Wegiel, B. et al. Cell surface biliverdin reductase mediates biliverdin-induced anti-inflammatory effects via phosphatidylinositol 3-kinase and Akt. J Biol Chem. 2009 Aug 7;284(32):21369-78.

51. Liu, Y. et al. Biliverdin reductase, a major physiologic cytoprotectant, suppresses experimental autoimmune encephalomyelitis. Free Radic Biol Med. 2006 Mar 15;40(6):960-7.

52. Cheng, D. et al. Small molecule regulators of protein arginine methyltransferases. J Biol Chem. 2004 Jun 4;279(23):23892-9.

53. Morales, Y. et al. Biochemistry and regulation of the protein arginine methyltransferases (PRMTs). Arch Biochem Biophys. 2016 Jan 15;590:138-152.

54. Cruzat, V. et al. Glutamine: Metabolism and Immune Function, Supplementation and Clinical Translation. Nutrients. 2018 Oct 23;10(11):1564.

55. Novershtern, N. et al. Densely interconnected transcriptional circuits control cell states in human hematopoiesis. Cell. 2011 Jan 21;144(2):296–309.

56. Linsley, P.S. et al. Copy number loss of the interferon gene cluster in melanomas is linked to reduced T cell infiltrate and poor patient prognosis. PloS One. 2014;9(10):e109760.

57. Rinchai, D. et al. A training curriculum for retrieving, structuring, and aggregating information derived from the biomedical literature and large-scale data repositories. [Internet]. F1000Research; 2022 [cited 2023 Mar 29]. Available from: https://f1000research.com/articles/11-994

58. Rinchai, D. et al. Assessing the potential relevance of CEACAM6 as a blood transcriptional biomarker [Internet]. F1000Research; 2022 [cited 2023 Mar 31]. Available from: https://f1000research.com/articles/11-1294

59. Kumar, S. et al. Free heme toxicity and its detoxification systems in human. Toxicol Lett. 2005 Jul 4;157(3):175-88.
