# Supplementary material for: Harnessing large language models (LLMs) for candidate gene prioritization and selection
Source: J Transl Med. 2023 Oct 16;21:728. doi: 10.1186/s12967-023-04576-8 (PMC10580627; doi:10.1186/s12967-023-04576-8)
Supplement: Supplementary file 9 — Additional file 9: Is the prioritization report for module M14.53 authored by Mohammed Toufiq. The file is shared in the PDF format. [file 12967_2023_4576_MOESM9_ESM.pdf]

M14.53 Prioritization report

Prepared by: Mohammed Toufiq (The Jackson Laboratory for Genomic Medicine, Farmington, CT)

1. Composition

The module M14.53 belongs to Aggregate A37 from the BloodGen3 fixed module repertoire (1, 2). This module aggregate is associated with circulating erythroid cells. It was found to be associated with RSV disease severity (3). We showed in the same study that the abundance of A27 transcripts is also elevated in the blood of patients with stage IV melanoma (3).

It comprises 18 genes: MARCH8, BCL2L1, BSG, CDC34, CHPT1, CHPT1, FHL2, GLRX5, IGF2BP2, KLF1, LOC650832, LOC653778, LOC654103, MAP2K3, RBM38, RBM38, RIOK3, TMEM63B.

2. Functional convergence

Functional Themes	Associated Genes	Comments
Ubiquitination and protein turnover	MARCH8, CDC34	The proteins encoded by MARCH8, CDC34 genes play key roles in protein ubiquitination and implicated in the process of protein sorting and degradation which required for cell cycle progression.
Signal transduction	BSG, FHL2, MAP2K3, RIOK3	It has multiple functions, including a role in cell adhesion, signal transduction, and matrix metalloproteinase induction. Plays a role in signal transduction, cell differentiation and proliferation. Part of the MAPK signaling pathway, playing a role in cell proliferation, differentiation, and apoptosis.Involved in signal transduction and cellular stress response.
Post-transcriptional regulation of RNA	IGF2BP2, RBM38	Has a role in RNA binding and is implicated in post-transcriptional regulation. Involved in post-transcriptional regulation of mRNA, including mRNA stability and translation.
Cell survival, proliferation, and death	BCL2L1, FHL2, MAP2K3	It is an anti-apoptotic gene involved in regulating cell death and survival. Plays a role in signal transduction, cell differentiation and proliferation. Part of the MAPK signaling pathway, playing a role in cell proliferation, differentiation, and apoptosis.

3. Scoring and prioritization

Genes were scored on six criteria using GPT-4 and Claude. The scores were averaged, and candidate genes ranked according to their cumulative scores (**Figure 1**, Methods: Step 3 and Step 4).

The two LLMs were requested to score each gene on the following six statements:
a. The gene is associated with erythroid cells or erythropoiesis.
b. The gene is currently being used as a biomarker in clinical settings.
c. The gene has potential value as a blood transcriptional biomarker.
d. The gene is relevant to circulating leukocytes immune biology.
e. The gene is a known drug target.
f. The gene is therapeutically relevant for immune-mediated diseases.

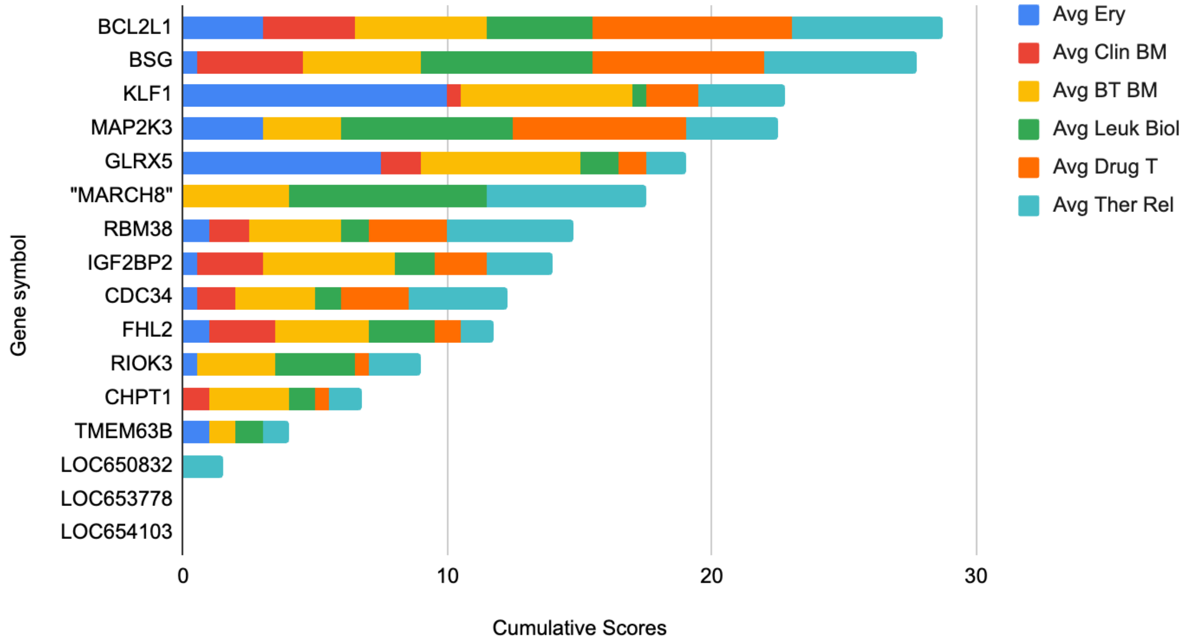

Figure 1: The stacked bar graph shows cumulative scores across six criteria for the 18 M14.53 genes.

The top five genes selected for further evaluation were: BCL2L1, BSG, KFL1, MAP2K3 and GLRX5 **(Figure 2**).

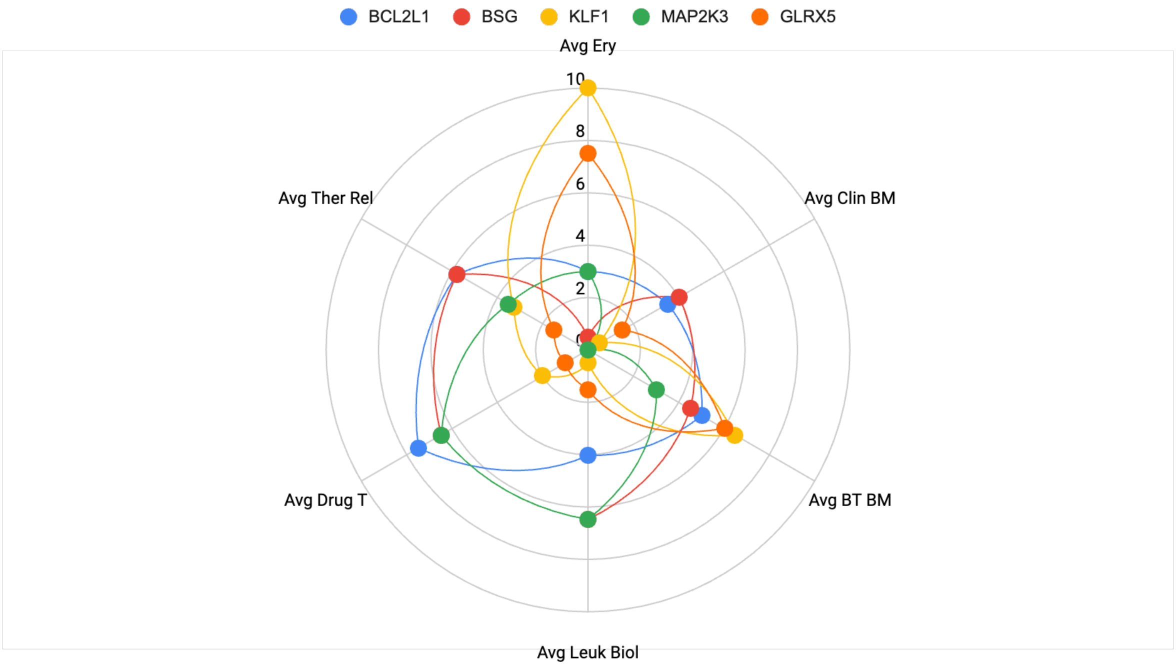

Figure 2: The radar plot represents individual scores for the 5 top scoring genes: BCL2L1, BSG, KFL1, MAP2K3 and GLRX5.

4. Knowledge-driven evaluation of top five candidates

Justifications given by the GPT-4 and Claude for the scores provided across the 6 criteria were compiled and summarized by GPT-4 (Methods: Step 5). For each statement pertinent references were retrieved using GPT-4 or Claude, with the relevance of each reference checked and attributed manually by researcher authoring this report.

Function: "The BCL2L1 gene produces two isoforms: Bcl-xL and Bcl-xS, which are integral in apoptosis regulation. Bcl-xL inhibits cell death by binding to pro-apoptotic factors, whereas Bcl-xS promotes apoptosis. Notably, Bcl-xL overexpression in cancers is associated with chemotherapy resistance ([https://pubmed.ncbi.nlm.nih.gov/23378584/] [https://pubmed.ncbi.nlm.nih.gov/19632186/] [https://pubmed.ncbi.nlm.nih.gov/18097445/] [https://pubmed.ncbi.nlm.nih.gov/9430630/] [https://pubmed.ncbi.nlm.nih.gov/8358789/] [https://pubmed.ncbi.nlm.nih.gov/11085534/] [https://pubmed.ncbi.nlm.nih.gov/10732784/] [https://pubmed.ncbi.nlm.nih.gov/7655019/]). The BSG gene encodes the glycoprotein basigin (CD147), which plays roles in cell adhesion, nutrient transport, and metalloproteinase activation, influencing processes like metastasis and inflammation ([https://pubmed.ncbi.nlm.nih.gov/7812975/] [https://pubmed.ncbi.nlm.nih.gov/10921872/] [https://pubmed.ncbi.nlm.nih.gov/26684586/] [https://pubmed.ncbi.nlm.nih.gov/9767135/] [https://pubmed.ncbi.nlm.nih.gov/15946952/]). The KLF1 gene, crucial for erythropoiesis,

controls the activation of genes essential for erythrocyte differentiation and maturation; mutations can lead to anemia ([https://pubmed.ncbi.nlm.nih.gov/21190291/] [https://pubmed.ncbi.nlm.nih.gov/26903544/] [https://pubmed.ncbi.nlm.nih.gov/20508144/] [https://pubmed.ncbi.nlm.nih.gov/21613252/] [https://pubmed.ncbi.nlm.nih.gov/21055716/] [https://pubmed.ncbi.nlm.nih.gov/21531944/]).The MAP2K3 gene produces a kinase operating in the MAP kinase pathway, impacting cell proliferation, differentiation, and immune reactions ([https://pubmed.ncbi.nlm.nih.gov/8622669/] [https://pubmed.ncbi.nlm.nih.gov/9430721/] [https://pubmed.ncbi.nlm.nih.gov/17496909/] [https://pubmed.ncbi.nlm.nih.gov/22535895/] [https://pubmed.ncbi.nlm.nih.gov/7914033/].). Lastly, the GLRX5 gene encodes glutaredoxin-5 in the mitochondrial matrix, essential for forming iron-sulfur clusters and managing mitochondrial iron balance and oxidative stress. Mutations here can result in sideroblastic anemia ([https://pubmed.ncbi.nlm.nih.gov/16110529/] [https://pubmed.ncbi.nlm.nih.gov/20481466/] [https://pubmed.ncbi.nlm.nih.gov/17485548/])".

a. Relevance to erythroid cells and erythropoiesis: "BCL2L1, encoding Bcl-xL, plays a role in erythroid progenitor cell survival, but its direct association with erythroid cells remains unconfirmed, given its widespread anti-apoptotic functions across diverse cell types ([https://pubmed.ncbi.nlm.nih.gov/10359572/] [https://pubmed.ncbi.nlm.nih.gov/9226163/] [https://pubmed.ncbi.nlm.nih.gov/9735050/]). The BSG gene, encoding CD147, lacks concrete evidence linking it to erythroid cells or erythropoiesis, as CD147 is universally expressed in numerous cells ([https://pubmed.ncbi.nlm.nih.gov/26684586/] [https://pubmed.ncbi.nlm.nih.gov/22080952/] [https://pubmed.ncbi.nlm.nih.gov/9501026/] [https://pubmed.ncbi.nlm.nih.gov/15946952/].). KLF1, on the other hand, exhibits a robust association with erythroid cells and erythropoiesis, functioning specifically as an essential erythroid transcription factor regulating differentiation-related genes ([https://pubmed.ncbi.nlm.nih.gov/21190291/] [https://pubmed.ncbi.nlm.nih.gov/21900194/] [https://pubmed.ncbi.nlm.nih.gov/10688844/] [https://pubmed.ncbi.nlm.nih.gov/21613252/]). The link between MAP2K3, a part of the MAP kinase pathway, and erythropoiesis is tenuous, with minimal evidence supporting a connection ([https://pubmed.ncbi.nlm.nih.gov/7750577/] [https://pubmed.ncbi.nlm.nih.gov/7839144/] [https://pubmed.ncbi.nlm.nih.gov/32429593/] [https://pubmed.ncbi.nlm.nih.gov/34299300/] [https://pubmed.ncbi.nlm.nih.gov/18383321/]). GLRX5 is integral to erythroid heme synthesis, iron metabolism, and erythroid cell maturation, though its activities extend beyond erythroid-specific processes. Notably, GLRX5 gene mutations contribute to sideroblastic anemia ([https://pubmed.ncbi.nlm.nih.gov/20364084/])".

b. Is used as a clinical biomarker: "In the realm of oncology, the BCL2L1 gene is predominantly investigated as a potential drug target due to its ties with apoptosis resistance, though its clinical value as a biomarker remains unvalidated ([https://pubmed.ncbi.nlm.nih.gov/26822577/] [https://pubmed.ncbi.nlm.nih.gov/17629468/] [https://pubmed.ncbi.nlm.nih.gov/19043450/] [https://pubmed.ncbi.nlm.nih.gov/27537525/]). BSG, also termed CD147, associated with cell invasion and metastasis in various cancers, also

lacks validated evidence for routine clinical use as a diagnostic or prognostic biomarker ([https://pubmed.ncbi.nlm.nih.gov/25081536/] [https://pubmed.ncbi.nlm.nih.gov/7812975/]). KLF1's importance in cellular processes doesn't translate to its routine use as a biomarker; however, its gene mutations are identifiable via genetic testing ([https://pubmed.ncbi.nlm.nih.gov/7753195/] [https://pubmed.ncbi.nlm.nih.gov/23065504/] [https://pubmed.ncbi.nlm.nih.gov/21055716/]). The MAP2K3 gene, part of the MAP kinase pathway, is currently not backed by research to serve as a clinical biomarker ([https://pubmed.ncbi.nlm.nih.gov/12471242/] [https://pubmed.ncbi.nlm.nih.gov/18383321/]). Although GLRX5 is vital for iron metabolism, it isn't traditionally employed as a clinical biomarker. Still, genetic testing can pinpoint anemias attributed to GLRX5 gene mutations ([https://pubmed.ncbi.nlm.nih.gov/20364084/] [https://pubmed.ncbi.nlm.nih.gov/17485548/])".

c. Potential relevance as a blood transcriptional biomarker: "BCL2L1, recognized for its anti-apoptotic properties, is under scrutiny as a potential blood transcriptional biomarker in oncology to assess disease progression and therapeutic effects, with its levels reflecting cellular apoptotic status. Still, its broad acceptance as a biomarker necessitates comprehensive validation ([https://pubmed.ncbi.nlm.nih.gov/26822577/] [https://pubmed.ncbi.nlm.nih.gov/35983950/] [https://pubmed.ncbi.nlm.nih.gov/12209154/] [https://pubmed.ncbi.nlm.nih.gov/17097560/] [https://pubmed.ncbi.nlm.nih.gov/30792387/]). BSG (CD147) is emerging as a promising blood transcriptional biomarker for certain malignancies and inflammatory conditions, with early studies linking it to metastasis and inflammation; rigorous validation is critical for its definitive clinical use ([https://pubmed.ncbi.nlm.nih.gov/26684586/] [https://pubmed.ncbi.nlm.nih.gov/25081536/] [https://pubmed.ncbi.nlm.nih.gov/22130661/] [https://pubmed.ncbi.nlm.nih.gov/19438743/] [https://pubmed.ncbi.nlm.nih.gov/17579119/] [https://pubmed.ncbi.nlm.nih.gov/11688976/]). KLF1's central role in erythropoiesis suggests its potential as a biomarker, where its levels could indicate erythroid progenitor cell activity ([https://pubmed.ncbi.nlm.nih.gov/32311573/] [https://pubmed.ncbi.nlm.nih.gov/21531944/] [https://pubmed.ncbi.nlm.nih.gov/34554218/] [https://pubmed.ncbi.nlm.nih.gov/21821711/] [https://pubmed.ncbi.nlm.nih.gov/20676097/]). MAP2K3, involved in immune cell signaling, is being explored as a leukocyte biomarker, but thorough validation is mandatory for its widespread clinical utilization ([https://pubmed.ncbi.nlm.nih.gov/23535064/] [https://pubmed.ncbi.nlm.nih.gov/15735648/]). Lastly, GLRX5, crucial for iron metabolism, presents potential as a blood transcriptional biomarker, particularly in iron-related conditions, with its levels reflecting mitochondrial iron metabolic status; however, its clinical implementation demands rigorous validation ([https://pubmed.ncbi.nlm.nih.gov/20364084/] [https://pubmed.ncbi.nlm.nih.gov/19731322/])".

d. Relevance to leukocytes immune biology: "BCL2L1, known for producing the anti-apoptotic Bcl-xL, plays a role in the survival of immune cells, especially lymphocytes, though its specific function in circulating leukocytes remains unverified ([https://pubmed.ncbi.nlm.nih.gov/11163351/] [https://pubmed.ncbi.nlm.nih.gov/37488586/] [https://pubmed.ncbi.nlm.nih.gov/17322918/] [https://pubmed.ncbi.nlm.nih.gov/8358789/]).

BSG, or CD147, is integral to leukocyte biology, aiding in lymphocyte proliferation, modulating macrophage responses, and facilitating leukocyte chemotaxis and extravasation at inflammation sites, emphasizing its key role in leukocyte functionality ([https://pubmed.ncbi.nlm.nih.gov/21083710/] [https://pubmed.ncbi.nlm.nih.gov/34421910/] [https://pubmed.ncbi.nlm.nih.gov/25977287/]). KLF1, primarily associated with erythroid cells, is not currently recognized as having a direct impact on the immune biology of circulating leukocytes ([https://pubmed.ncbi.nlm.nih.gov/20616217/] [https://pubmed.ncbi.nlm.nih.gov/35822667/] [https://pubmed.ncbi.nlm.nih.gov/33570494/]). MAP2K3, vital for leukocyte signaling, is implicated in modulating cytokine production, signifying its importance in immune cell biology ([https://pubmed.ncbi.nlm.nih.gov/12951578/] [https://pubmed.ncbi.nlm.nih.gov/16567640/] [https://pubmed.ncbi.nlm.nih.gov/22101742/] [https://pubmed.ncbi.nlm.nih.gov/23580600/] [https://pubmed.ncbi.nlm.nih.gov/12951578/] [https://pubmed.ncbi.nlm.nih.gov/35743792/]). Meanwhile, the role of GLRX5, a mitochondrial protein, in circulating leukocyte immune biology remains ambiguous, necessitating further research for clarity ([https://pubmed.ncbi.nlm.nih.gov/25311648/] [https://pubmed.ncbi.nlm.nih.gov/30660387/])".

e. Is a known drug target: "BCL2L1, via its product Bcl-xL, is known to contribute to chemotherapy resistance in cancer, spurring active efforts to develop Bcl-xL inhibitors, which are currently in clinical trials targeting various cancers ([https://pubmed.ncbi.nlm.nih.gov/30537511/] [https://pubmed.ncbi.nlm.nih.gov/18806758/] [https://pubmed.ncbi.nlm.nih.gov/11085534/] [https://pubmed.ncbi.nlm.nih.gov/15902208/] [https://pubmed.ncbi.nlm.nih.gov/23291630/] [https://pubmed.ncbi.nlm.nih.gov/22184378/] [https://pubmed.ncbi.nlm.nih.gov/22426421/] [https://pubmed.ncbi.nlm.nih.gov/18657507/]). BSG, or CD147, has been marked as a significant drug target in oncology due to its critical role in tumor invasion and metastasis, with anti-BSG/CD147 antibodies showing anti-tumor effects in preclinical and early clinical trials ([https://pubmed.ncbi.nlm.nih.gov/26604323/] [https://pubmed.ncbi.nlm.nih.gov/25268615/] [https://pubmed.ncbi.nlm.nih.gov/17145878/] [https://pubmed.ncbi.nlm.nih.gov/15781323/] [https://pubmed.ncbi.nlm.nih.gov/24801417/] [https://pubmed.ncbi.nlm.nih.gov/37090718/]). While KLF1 is linked to blood disorders, it lacks broad recognition as a drug target, with minimal evidence supporting its potential in drug development ([https://pubmed.ncbi.nlm.nih.gov/24443441/] [https://pubmed.ncbi.nlm.nih.gov/26903544/] [https://pubmed.ncbi.nlm.nih.gov/35130609/] [https://pubmed.ncbi.nlm.nih.gov/26679864/]). MAP2K3 emerges as a key drug target in inflammatory disease research, with ongoing clinical trials of p38 inhibitors, believed to target MAP2K3, focusing on conditions like rheumatoid arthritis ([https://pubmed.ncbi.nlm.nih.gov/12951578/] [https://pubmed.ncbi.nlm.nih.gov/17827184/] [https://pubmed.ncbi.nlm.nih.gov/22535895/] [https://pubmed.ncbi.nlm.nih.gov/23954936/] [https://pubmed.ncbi.nlm.nih.gov/15735648/]). Conversely, despite GLRX5's essential role in iron metabolism, it is not currently recognized as a primary drug target, indicating a potential area for expanded research ([https://pubmed.ncbi.nlm.nih.gov/32685019/] [https://pubmed.ncbi.nlm.nih.gov/37345031/] [https://pubmed.ncbi.nlm.nih.gov/34732213/] [https://assets.researchsquare.com/files/rs-971437/v1/a355aff9-4ab7-412d-8034-34f42d433397.pdf?c=1642404554])".

f. Potential therapeutic relevance for immune-mediated diseases: "BCL2L1, although involved in immune cell survival regulation, is primarily noted for its role in conferring resistance to apoptosis in lymphocytes, particularly in autoimmune disorders like systemic lupus erythematosus (SLE) ([https://pubmed.ncbi.nlm.nih.gov/22342458/] [https://pubmed.ncbi.nlm.nih.gov/35983950/] [https://pubmed.ncbi.nlm.nih.gov/25473892/] [https://pubmed.ncbi.nlm.nih.gov/17534896/] [https://pubmed.ncbi.nlm.nih.gov/37488586/] [https://pubmed.ncbi.nlm.nih.gov/7878471/]). BSG, known for its roles in inflammatory responses and lymphocyte proliferation, has been linked with inflammation in conditions such as rheumatoid arthritis, multiple sclerosis, and lung disease, even though its research isn't limited to the immune context ([https://pubmed.ncbi.nlm.nih.gov/21228176/] [https://pubmed.ncbi.nlm.nih.gov/19350111/] [https://pubmed.ncbi.nlm.nih.gov/11943775/] [https://pubmed.ncbi.nlm.nih.gov/11353871/]). KLF1, central to erythropoiesis, is not directly associated with immune-mediated diseases, remaining primarily an erythroid transcription factor ([https://pubmed.ncbi.nlm.nih.gov/29728568/] [https://pubmed.ncbi.nlm.nih.gov/7753195/] [https://pubmed.ncbi.nlm.nih.gov/36231031/]). MAP2K3, instrumental in proinflammatory signaling in immune cells, is gaining traction as a potential therapeutic target for immune-mediated conditions ([https://pubmed.ncbi.nlm.nih.gov/23954936/] [https://pubmed.ncbi.nlm.nih.gov/7535770/] [https://pubmed.ncbi.nlm.nih.gov/19995751/] [https://pubmed.ncbi.nlm.nih.gov/12951578/]). Meanwhile, GLRX5, closely tied to sideroblastic anemia, lacks definitive evidence of its involvement in the pathogenesis of immune-mediated diseases, warranting further research in this domain ([https://pubmed.ncbi.nlm.nih.gov/32685019/] [https://pubmed.ncbi.nlm.nih.gov/20364084/] [https://pubmed.ncbi.nlm.nih.gov/34732213/] [https://pubmed.ncbi.nlm.nih.gov/30401706/])".

5. Examining expression patterns of top 5 candidates across leukocyte populations

The expression patterns of the top 5 candidate genes were examined across diverse leukocyte populations and hematopoietic precursors using two reference transcriptomic datasets.

5.1 Densely interconnected transcriptional circuits control cell states in human hematopoiesis – (GSE24759)
http://developmentalimmunology.gxbsidra.org/dm3/geneBrowser/show/4000026

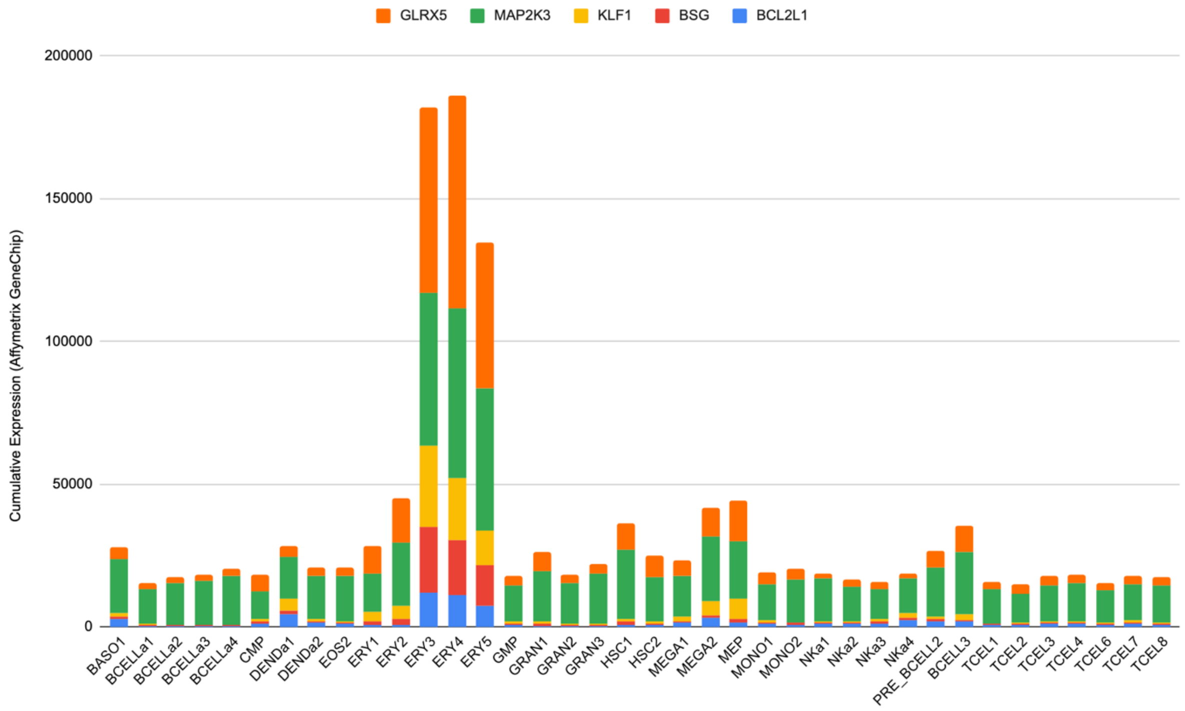

Figure 3: This stacked bar graph shows the expression levels of five candidate genes. ERY3-5 populations are CD71+ GLYA+ erythroid cells. The original article provides full experimental details: (4). The web link above also provides access to more complete information about this dataset as well as individual gene profiles.

5.2 Next generation sequencing of human immune cell subsets across diseases – (GSE60424)
http://cd2k.gxbsidra.org/dm3/geneBrowser/show/4000098

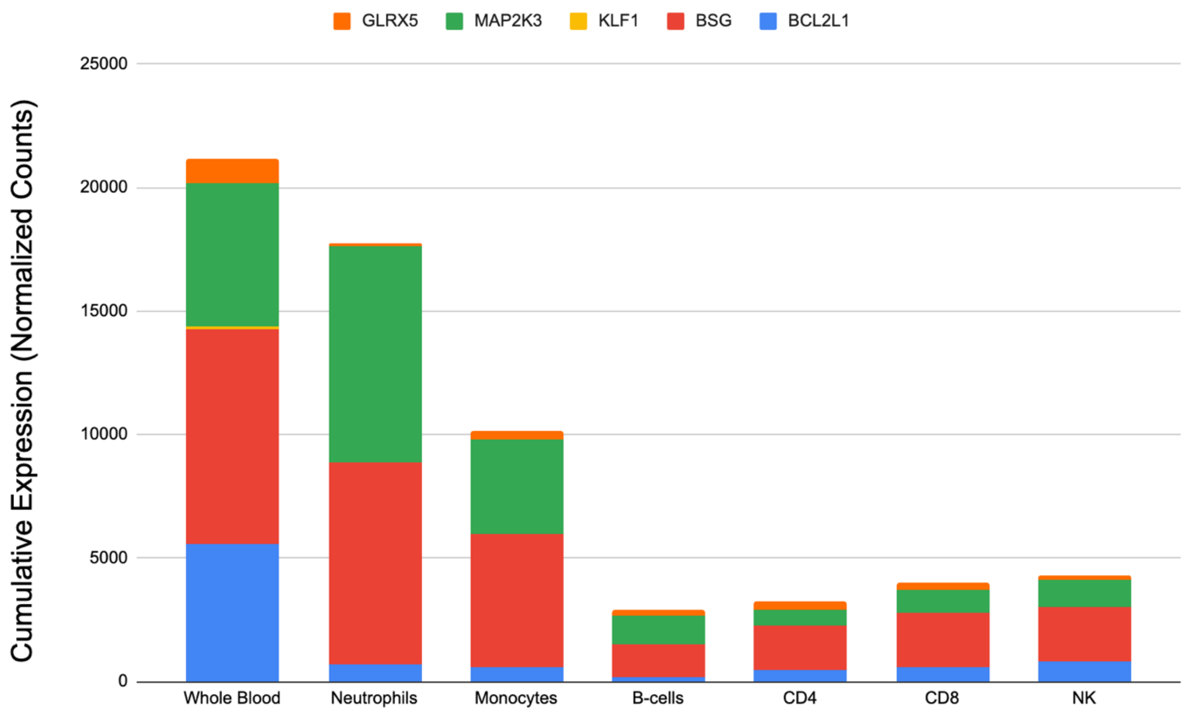

Figure 4: This stacked bar graph shows the expression levels measured by RNAseq of the five candidate genes across six leukocyte populations and whole blood. The abundance is shown for each gene in each population for an average of up to 20 samples. Additional experimental details are accessible via the link provided above and is available as part of this sample series GEO record (GSE60424) (5).

5.3 BloodGen3 reference cohorts (GSE100150)

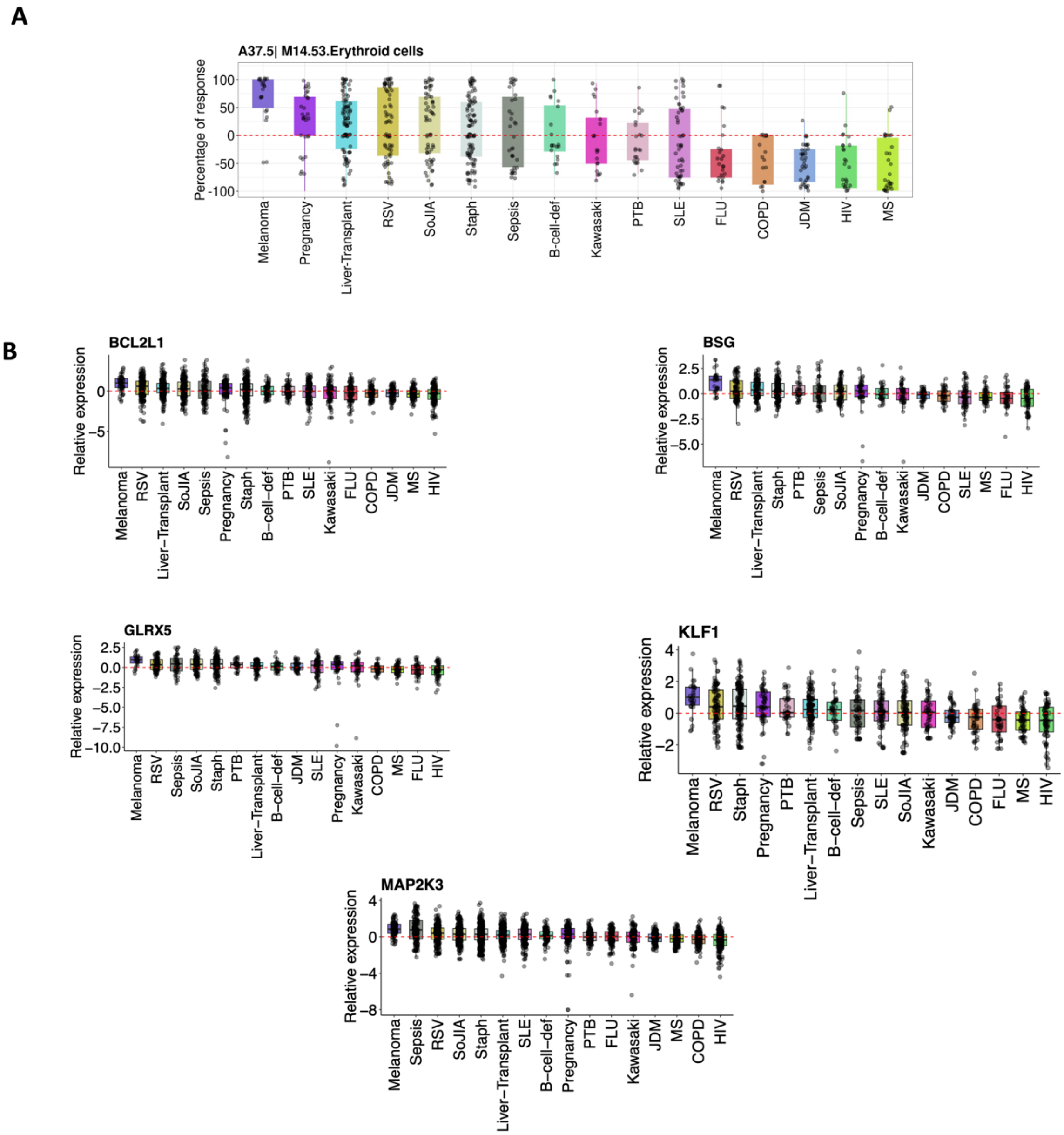

Figure 5: The box plot in panel A shows the changes in abundance of transcripts comprising the M14.53 module, expressed within a cohort as the percentage of genes showing differential expression between each patient and the respective set of control subjects for this cohort. Differential expression is based on fixed cut-off (Log2 Fold Change >1.5, difference>10). These values are expressed as % module activity and can range from 100% (all genes are upregulated) to -100% (all genes are downregulated). The box plot in panel B show expression levels for the top 5 scoring M14.53 genes in patient cohorts relative to respective control subjects matched for demographic variables.

6. Overall pick(s) for M14.53

We aim to establish a universal immune profiling assay that measures transcript abundance of a panel of genes in whole blood using small- or meso-scale molecular profiling technology. Compelling candidates identified here from the M14.53 selection pool will be subjected to more in-depth evaluation using a pre-established workflow (6, 7).

Claude and GPT-4 were prompted to select a top candidate on the basis of the information summarized above in section 4, taking into account all six scoring criteria equally (Methods: Step 7).

Claude chose KLF1:

Here is a summary of the key conclusions regarding selection of a candidate gene for inclusion in a targeted blood transcriptional profiling panel:
Initial Summary:

Based on preliminary evidence, KLF1 was recommended due to its specialized role in erythropoiesis and erythroid cell biology.

Leukocyte RNA-seq Data:

- Low KLF1 expression in immune cell types supported its erythroid specificity over immune function.

Leukocyte Microarray Data:

- High KLF1 expression found specifically in erythroid precursor cells confirmed its relevance to erythropoiesis.

Patient Cohort RNA Abundance Data:

- Moderate decreases in KLF1 levels across immune-related diseases provided further evidence of its usefulness as an erythroid focused biomarker.

In summary, KLF1 was consistently recommended throughout the process due to multiple lines of evidence indicating its vital role in erythropoiesis and high expression in erythroid cell types. The data sources reinforced KLF1 as an ideal candidate to meet the intended purpose of an erythroid focused blood transcriptional biomarker for the panel.

GPT-4 chose BSG:

Of course! Here's a summary of the key conclusions drawn from the conversation and the data you provided:

1. Gene Selection: You provided data on five genes – BCL2L1, BSG, KLF1, MAP2K3, and GLRX5.

2. Immune Cells & RNAseq Data:
- BCL2L1 was identified as a potential marker for lymphocyte differentiation and has been associated with multiple immune processes.
- BSG (also known as CD147) has roles in T-cell activation and is involved in inflammatory processes.
- KLF1 is significant for erythroid differentiation and function.
- MAP2K3 is involved in the p38 MAPK signaling pathway, playing a role in various immune responses.
- GLRX5 is involved in iron-sulfur cluster biogenesis in mitochondria and affects cell proliferation.

3. Microarray Data: You presented averaged log2 fold changes in RNA abundance from patients with different diseases compared to controls.

Key Observations:
- BCL2L1:
- Elevated in metastatic melanoma, multiple sclerosis, and some other conditions.
- Reduced in HIV infection, acute influenza, Kawasaki disease, and some other conditions.
- BSG:
- Elevated in metastatic melanoma, acute respiratory syncytial virus infection, and some other conditions.
- Reduced most notably in HIV infection and acute influenza.
- KLF1:
- Elevated in metastatic melanoma, active pulmonary tuberculosis, and pregnant subjects.
- Reduced significantly in HIV infection, acute influenza, and multiple sclerosis.
- MAP2K3:
- Elevated in metastatic melanoma, sepsis, and some other conditions.
- Reduced in HIV infection, chronic obstructive pulmonary disease, and multiple sclerosis.
- GLRX5 (incomplete data):

• Elevated in patients with B-cell deficiency.

4. Considerations:

• Several diseases, notably HIV infection and acute influenza, commonly showed reduced RNA abundance across multiple genes.

• Metastatic melanoma often showed an increase in RNA abundance.

• The provided data could suggest potential roles for these genes in immune response or alterations during certain disease states. The functions of these genes in immune cells and their differential expression in various diseases indicate their potential importance in immune system regulation or pathology of the mentioned diseases.

In summary, the provided genes are significant in various aspects of immune function and show differential expression in various diseases. Further research is needed to clarify their exact roles and potential as therapeutic targets or disease markers.

Given the information and discussion so far, I would prioritize BSG (CD147) as a candidate gene for further investigation, and here are the reasons:

1. Broad Immunological Relevance: BSG (CD147) is known to have roles in T-cell activation and is involved in inflammatory processes. Its function directly relates to the immune system, making it a prime candidate for understanding immune responses.

2. Differential Expression: In the provided RNA abundance data, BSG showed notable differential expression across several conditions. Its elevated expression in metastatic melanoma and acute respiratory syncytial virus infection and reduced expression in HIV infection and acute influenza suggest its potential importance in both immune response and pathology.

3. Current Relevance: Given that respiratory infections like COVID-19 have been a global concern, understanding genes like BSG, which show differential expression in respiratory viral infections, could have broader implications for therapeutic strategies.

4. Potential Therapeutic Target: Due to its role in T-cell activation and involvement in inflammatory processes, BSG presents itself as a potential therapeutic target. Modulating its expression or function might help in treating diseases where immune regulation is disrupted. That said, the decision to focus on BSG doesn't diminish the significance of the other genes. Each gene has its unique role and could be significant in its own right depending on the research question. BSG, however, stands out due to its direct relevance to the immune system and its clear differential expression across a range of diseases.

The LLMs did not incorporate reference transcriptional profiling data in their evaluation and prioritization of candidate genes. Given its comparatively higher expression levels and restriction across leukocyte populations, our top choice based on this criterion is KLF1. Considering this, in conjunction with the rationale provided by both LLMs and our understanding of BSG and KLF1 biological and clinical relevance, we identify KLF1 as our primary M14.53 selection for subsequent characterization.

Prompts	CLAUDE	GPT-4
PROMPT 1: Based on the summary below, please recommend the top candidate gene to include in a targeted blood transcriptional profiling panel. The intent is for the selected gene to be representative of an erythroid cell signature associated with response to mRNA vaccines, severity of RSV infection and that found to be expressed at high levels in patients with metastatic melanoma and in liver transplant recipients. Please weigh the following criteria equally in your recommendation: 1) Relevance to erythroid cells and erythropoiesis, 2) Current use as a biomarker, 3) Potential use as a blood transcriptional biomarker, 4) Relevance to leukocyte immune biology, 5) Status as a known drug target, 6) Therapeutic relevance for immune mediated diseases: *[input summarized and fact-*	Based on the criteria provided, I would recommend KLF1 as the top candidate gene to include in the targeted blood transcriptional profiling panel. The key reasons are:<br>• KLF1 has a robust, specific association with erythroid cells and erythropoiesis as an essential erythroid transcription factor. This strongly aligns with the criterion of relevance to erythroid biology.<br>• KLF1 mutations can cause anemia, highlighting its role in erythroid disorders relevant to conditions like RSV severity.<br>• As a master regulator of erythropoiesis, KLF1 has clear potential as a biomarker of erythroid progenitor cell activity.<br>• KLF1 meets the criteria of linking to anemia, erythropoiesis, and holding promise as a blood biomarker for erythroid biology.	Given the detailed summary and weighing the provided criteria equally, here's an evaluation of each gene based on the criteria:<br>1. Relevance to erythroid cells and erythropoiesis:<br>• BCL2L1: Plays a role in erythroid progenitor cell survival, but its association with erythroid cells is unconfirmed.<br>• BSG (CD147): Lacks evidence linking it to erythroid cells.<br>• KLF1: Exhibits a robust association with erythroid cells and erythropoiesis, functioning as an erythroid transcription factor.<br>• MAP2K3: Minimal evidence supporting its connection to erythropoiesis.<br>• GLRX5: Integral to erythroid heme synthesis, iron metabolism, and erythroid cell maturation.<br>2. Current use as a biomarker:

checked justifications from Step 5]	• The other genes have more peripheral or unestablished connections to erythroid cells and lack KLF1's definitive specificity in erythropoiesis.<br>• While not a drug target itself, KLF1 levels could provide insights into mRNA vaccine response, RSV severity, and other erythroid-related conditions.<br>In summary, KLF1 is the strongest fit for the intended purpose of the panel due to its specialized role in erythroid cell biology and strong associations with erythropoiesis and anemia. It surpasses the other genes for meeting the stated criteria.	• BCL2L1: Clinical value remains unvalidated.<br>• BSG (CD147): Lacks validated evidence for clinical use.<br>• KLF1: Not routinely used as a biomarker, but mutations identifiable via genetic testing.<br>• MAP2K3: Not backed by research as a clinical biomarker.<br>• GLRX5: Not traditionally employed as a clinical biomarker, but genetic testing can identify anemias due to its mutations.<br>3. Potential use as a blood transcriptional biomarker:<br>• BCL2L1: Potential, but requires validation.<br>• BSG (CD147): Emerging with potential, requires validation.<br>• KLF1: Shows potential due to its role in erythropoiesis.<br>• MAP2K3: Being explored, but requires thorough validation.<br>• GLRX5: Potential, especially in iron-related conditions; requires validation.<br>4. Relevance to leukocyte immune biology:<br>• BCL2L1: Plays a role in the survival of lymphocytes.<br>• BSG (CD147): Integral to leukocyte biology.

		• KLF1: Not recognized as impacting leukocyte immune biology.<br>• MAP2K3: Vital for leukocyte signaling.<br>• GLRX5: Role remains ambiguous.<br>5. Status as a known drug target:<br>• BCL2L1: Potential target due to chemotherapy resistance in cancer.<br>• BSG (CD147): Significant drug target in oncology.<br>• KLF1: Lacks broad recognition as a drug target.<br>• MAP2K3: Key drug target in inflammatory diseases.<br>• GLRX5: Not recognized as a primary drug target.<br>6. Therapeutic relevance for immune mediated diseases:<br>• BCL2L1: Role in autoimmune disorders like SLE.<br>• BSG (CD147): Linked with conditions like rheumatoid arthritis and multiple sclerosis.<br>• KLF1: Not directly associated with immune-mediated diseases.<br>• MAP2K3: Potential therapeutic target for immune-mediated conditions.<br>• GLRX5: Lacks definitive evidence of involvement.<br>Recommendation: Based on the equal weighting of the

		criteria, KLF1 emerges as the top candidate gene. It has a robust association with erythroid cells and erythropoiesis (Criterion 1), shows potential as a blood transcriptional biomarker (Criterion 3), and although it's not predominantly recognized in the immune context (Criterion 4) or as a major drug target (Criterion 5), its primary relevance to erythroid cells makes it particularly suitable for a targeted blood transcriptional profiling panel focusing on an erythroid cell signature.
PROMP 2: Now that you have provided an initial recommendation based on the summary, take into account the following RNA-sequencing expression data from key immune cell types and whole blood samples: *[input textualized transcriptional data].* Given this additional RNA-seq expression data, does your recommended gene selection remain the same? Please explain which gene you would recommend at this stage and why, incorporating both the summarized information *provided earlier and RNA-seq data in your rationale."*	After reviewing the additional RNA-seq expression data, I would maintain my original recommendation of KLF1 as the top candidate gene.<br>The key reasons are:<br>• The RNA-seq data shows KLF1 expression is highly specific to whole blood, with minimal detection in isolated immune cell types. This fits with its erythroid specificity.<br>• In contrast, the other genes like BCL2L1, BSG, MAP2K3, and GLRX5 show broader expression across immune cells, indicating more generalized roles.<br>• The RNA-seq data reinforces that KLF1's expression is restricted to erythroid lineages, while	Given the RNA-seq data and the initial summary, let's re-evaluate our top candidate gene:<br>1. Relevance to erythroid cells and erythropoiesis:<br>• KLF1 is the strongest candidate in this domain, being essential for erythropoiesis.<br>• GLRX5 also plays a role in erythroid heme synthesis and iron metabolism.<br>2. Current use as a biomarker:<br>• Neither BCL2L1, BSG, KLF1, MAP2K3, nor GLRX5 are currently used widely as clinical biomarkers, although BCL2L1 and BSG show some potential in oncology and inflammation, respectively.

	the other genes are more widely expressed.<br>• Low KLF1 levels in isolated leukocytes supports its strong erythroid association over immune function.<br>• The RNA-seq data aligns with and provides confirming evidence for the summary indicating KLF1's specialized erythroid role.<br>In summary, the addition of RNA-seq expression data further solidifies KLF1 as highly specific to erythroid cells, which aligns with the rationale for originally selecting it as the top erythroid-focused candidate. The new data is consistent with the summary information for KLF1.	3. Potential use as a blood transcriptional biomarker:<br>• BSG (CD147) shows promise here, especially given its relatively high RNA-seq counts in whole blood and key immune cells.<br>• BCL2L1 and MAP2K3 also have notable RNA-seq counts in blood, suggesting potential as blood transcriptional biomarkers.<br>4. Relevance to leukocyte immune biology:<br>• BSG has a key role in leukocyte functionality and shows high RNA-seq counts across various immune cell types, making it a front-runner in this domain.<br>• MAP2K3 is also vital for immune cell signaling, further evidenced by its substantial RNA-seq counts in neutrophils.<br>5. Status as a known drug target:<br>• BCL2L1 and BSG are strong candidates. Both have ongoing or early clinical trials, targeting various conditions.<br>6. Therapeutic relevance for immune-mediated diseases:<br>• BSG is linked to various inflammatory conditions.<br>• MAP2K3 has potential as a therapeutic target for immune-mediated conditions.

		Given the criteria, let's weigh the RNA-seq data:<br>• BCL2L1: Moderate expression in whole blood and certain immune cell types like NK cells.<br>• BSG (CD147): High expression in whole blood and across all immune cell types, especially neutrophils.<br>• KLF1: Very low expression in whole blood and almost negligible in immune cell types, implying its specificity to erythroid cells.<br>• MAP2K3: High expression in whole blood, highest in neutrophils.<br>• GLRX5: Moderate expression in whole blood, consistent across all immune cells.<br>Recommendation: While the initial analysis pointed towards KLF1 due to its robust association with erythroid cells and erythropoiesis, the RNA-seq data sheds new light, especially for criteria 3 (blood transcriptional biomarker) and 4 (relevance to leukocyte immune biology).<br>Given the RNA-seq data, BSG (CD147) stands out. It's highly expressed in whole blood and across various immune cell types. Its involvement in leukocyte functionality, ongoing clinical trials targeting its role in metastasis, and its link to

		inflammatory conditions support its potential as a key biomarker. Therefore, BSG (CD147) would be the top recommendation considering both the summarized information and the RNA-seq data.
PROMPT 3: Now that you have provided an initial recommendation based on the summary and the RNAseq data, take into account the following microarray expression data from key immune cell types and hematopoietic precursors: *[input textualized transcriptional data].* Given this additional microarray expression data, does your recommended gene selection remain the same? Please explain which gene you would recommend at this stage and why, incorporating the summarized information and RNA-seq data provided earlier, as well as this new microarray data in your rationale.	After reviewing the microarray expression data, I would maintain my original recommendation of KLF1 as the top candidate gene.<br>The key reasons are:<br>• The microarray data shows very high expression of KLF1 specifically in erythroid precursor cells, confirming its specialized role in erythropoiesis.<br>• There is minimal KLF1 expression across most immune cell types, which fits with its erythroid specificity.<br>• In comparison, the other genes like BCL2L1, BSG, MAP2K3, and GLRX5 exhibit broader expression across immune cells and lack erythroid selectivity.<br>• The RNA-seq and microarray data align in indicating the unique erythroid lineage expression pattern of KLF1.<br>• This expression profile fits the intended purpose of a biomarker reflecting erythroid biology for the panel.<br>In summary, the addition of the microarray data provides further confirming evidence	*[N/A: input exceed GPT-4 limits. Attempts to break down inputting the textualized data were unsuccessful as GPT-4 lost the thread of the conversation. Thus, we chose to skip to the next prompt]*

	that KLF1 has the ideal expression pattern for an erythroid focused blood transcriptional biomarker based on its specificity to erythroid precursors. Therefore, KLF1 remains the top choice.	
PROMPT 4: Now that you have provided an initial recommendation based on the summary and the immune cells RNAseq and microarray data, take into account the following averaged log2 fold changes in RNA abundance in patient cohorts compared to controls. *[input textualized transcriptional data].* Given this additional data, does your recommended gene selection remain the same? Please explain which gene you would recommend at this stage and why, incorporating the summarized information, the leukocytes RNA-seq and microarray data provided earlier, as well as these new patient cohort profiles in your rationale.	After reviewing the RNA abundance log fold change data for the various patient cohorts, I would maintain my recommendation of KLF1 as the top candidate gene.<br>The key reasons are:<br>• KLF1 showed the most consistent decreases across conditions like influenza, HIV, juvenile arthritis, etc, implying correlation with immune activity.<br>• The decreases were moderate overall (in -0.2 to -1.2 range) indicating erythroid activity changes but not radical fluctuations.<br>• Other genes like BCL2L1 and GLRX5 had more variable patterns of increase/decrease among the cohorts.<br>• The cohort profiles align with use of KLF1 as a biomarker reflecting shifts in erythroid biology.<br>• KLF1's specialized role in erythropoiesis makes its cohort patterns most relevant to the intended purpose.<br>In summary, the cohort RNA abundance changes provide further supporting evidence	Analyzing this data, it's clear that there are varied responses in the RNA abundance of the genes of interest (BCL2L1, BSG, KLF1, MAP2K3, and GLRX5) across multiple conditions when compared to controls. I'll summarize the insights for each gene:<br>1. BCL2L1:<br>• Increased in: B-cell deficiency, metastatic melanoma, multiple sclerosis, active pulmonary tuberculosis, acute respiratory syncytial virus infection, sepsis, systemic onset juvenile idiopathic arthritis, staphylococcus aureus infection, and liver transplant recipients under maintenance therapy.<br>• Decreased in: chronic obstructive pulmonary disease, acute influenza infection, HIV infection, juvenile dermatomyositis, Kawasaki disease, pregnant subjects, and systemic lupus erythematosus.<br>2. BSG:<br>• Increased in: B-cell deficiency, metastatic melanoma, pregnant subjects, active pulmonary

	that KLF1 exhibits moderate but detectable decreases across diverse immune-related conditions. This fits well with the rationale for selecting KLF1 to provide an erythroid focused blood transcriptional signature. The new data does not provide any reason to reconsider KLF1 as the top choice.	tuberculosis, acute respiratory syncytial virus infection, sepsis, systemic onset juvenile idiopathic arthritis, staphylococcus aureus infection, and liver transplant recipients under maintenance therapy.<br>• Decreased in: chronic obstructive pulmonary disease, acute influenza infection, HIV infection, juvenile dermatomyositis, Kawasaki disease, multiple sclerosis, and systemic lupus erythematosus.<br>3. KLF1:<br>• Increased in: B-cell deficiency, metastatic melanoma, pregnant subjects, active pulmonary tuberculosis, acute respiratory syncytial virus infection, sepsis, systemic lupus erythematosus, staphylococcus aureus infection, and liver transplant recipients under maintenance therapy.<br>• Decreased in: chronic obstructive pulmonary disease, acute influenza infection, HIV infection, juvenile dermatomyositis, Kawasaki disease, and multiple sclerosis.<br>4. MAP2K3:<br>• Increased in: B-cell deficiency, metastatic melanoma, pregnant subjects, acute respiratory syncytial virus infection, sepsis, systemic lupus

		erythematosus, systemic onset juvenile idiopathic arthritis, staphylococcus aureus infection, and liver transplant recipients under maintenance therapy.<br>• Decreased in: chronic obstructive pulmonary disease, HIV infection, juvenile dermatomyositis, Kawasaki disease, and multiple sclerosis.<br>5. GLRX5:<br>• The data for GLRX5 was not completely shown, but based on the provided information, it was increased in B-cell deficiency.<br>Notable Observations:<br>• HIV infection shows a noticeable reduction across all genes, indicating a broad suppression of these genes in this condition.<br>• Metastatic melanoma is associated with an increase in the expression of all genes listed, suggesting an upregulation of these genes in the context of this type of cancer.<br>• Sepsis also showed an upregulation for all genes except for KLF1, which had a minor increase.<br>From this information, it's evident that while some diseases or conditions, like HIV, appear to broadly suppress the expression of these genes, others like metastatic melanoma and

		sepsis, tend to have a broad increase in their expression. This provides valuable information about the potential role these genes might play in the underlying mechanisms of these conditions.<br>However, understanding the direct implications requires deeper analysis, including protein-level studies, pathway analysis, and functional assays.
PROMPT 5: Could you please summarize the key conclusions you have drawn from the conversation so far?	Here is a summary of the key conclusions regarding selection of a candidate gene for inclusion in a targeted blood transcriptional profiling panel:<br>Initial Summary:<br>• Based on preliminary evidence, KLF1 was recommended due to its specialized role in erythropoiesis and erythroid cell biology.<br>Leukocyte RNA-seq Data:<br>• Low KLF1 expression in immune cell types supported its erythroid specificity over immune function.<br>Leukocyte Microarray Data:<br>• High KLF1 expression found specifically in erythroid precursor cells confirmed its relevance to erythropoiesis.<br>Patient Cohort RNA Abundance Data:<br>• Moderate decreases in KLF1 levels across	Of course! Here's a summary of the key conclusions drawn from the conversation and the data you provided:<br>1. Gene Selection: You provided data on five genes – BCL2L1, BSG, KLF1, MAP2K3, and GLRX5.<br>2. Immune Cells & RNAseq Data:<br>• BCL2L1 was identified as a potential marker for lymphocyte differentiation and has been associated with multiple immune processes.<br>• BSG (also known as CD147) has roles in T-cell activation and is involved in inflammatory processes.<br>• KLF1 is significant for erythroid differentiation and function.<br>• MAP2K3 is involved in the p38 MAPK signaling pathway, playing a role in various immune responses.<br>• GLRX5 is involved in iron-sulfur cluster

	immune-related diseases provided further evidence of its usefulness as an erythroid focused biomarker.<br>In summary, KLF1 was consistently recommended throughout the process due to multiple lines of evidence indicating its vital role in erythropoiesis and high expression in erythroid cell types. The data sources reinforced KLF1 as an ideal candidate to meet the intended purpose of an erythroid focused blood transcriptional biomarker for the panel.	biogenesis in mitochondria and affects cell proliferation.<br>3. Microarray Data: You presented averaged log2 fold changes in RNA abundance from patients with different diseases compared to controls.<br>Key Observations:<br>• BCL2L1:<br>• Elevated in metastatic melanoma, multiple sclerosis, and some other conditions.<br>• Reduced in HIV infection, acute influenza, Kawasaki disease, and some other conditions.<br>• BSG:<br>• Elevated in metastatic melanoma, acute respiratory syncytial virus infection, and some other conditions.<br>• Reduced most notably in HIV infection and acute influenza.<br>• KLF1:<br>• Elevated in metastatic melanoma, active pulmonary tuberculosis, and pregnant subjects.<br>• Reduced significantly in HIV infection, acute influenza, and multiple sclerosis.<br>• MAP2K3:<br>• Elevated in metastatic melanoma, sepsis, and some other conditions.<br>• Reduced in HIV infection, chronic obstructive pulmonary

		disease, and multiple sclerosis.<br>• GLRX5 (incomplete data):<br>• Elevated in patients with B-cell deficiency.<br>4. Considerations:<br>• Several diseases, notably HIV infection and acute influenza, commonly showed reduced RNA abundance across multiple genes.<br>• Metastatic melanoma often showed an increase in RNA abundance.<br>• The provided data could suggest potential roles for these genes in immune response or alterations during certain disease states. The functions of these genes in immune cells and their differential expression in various diseases indicate their potential importance in immune system regulation or pathology of the mentioned diseases.<br>In summary, the provided genes are significant in various aspects of immune function and show differential expression in various diseases. Further research is needed to clarify their exact roles and potential as therapeutic targets or disease markers.<br><br>Given the information and discussion so far, I would prioritize BSG (CD147) as a candidate gene for further

		investigation, and here are the reasons:<br>1. Broad Immunological Relevance: BSG (CD147) is known to have roles in T-cell activation and is involved in inflammatory processes. Its function directly relates to the immune system, making it a prime candidate for understanding immune responses.<br>2. Differential Expression: In the provided RNA abundance data, BSG showed notable differential expression across several conditions. Its elevated expression in metastatic melanoma and acute respiratory syncytial virus infection and reduced expression in HIV infection and acute influenza suggest its potential importance in both immune response and pathology.<br>3. Current Relevance: Given that respiratory infections like COVID-19 have been a global concern, understanding genes like BSG, which show differential expression in respiratory viral infections, could have broader implications for therapeutic strategies.<br>4. Potential Therapeutic Target: Due to its role in T-cell activation and involvement in inflammatory processes, BSG presents itself as a potential therapeutic target.

		Modulating its expression or function might help in treating diseases where immune regulation is disrupted.<br>That said, the decision to focus on BSG doesn't diminish the significance of the other genes. Each gene has its unique role and could be significant in its own right depending on the research question. BSG, however, stands out due to its direct relevance to the immune system and its clear differential expression across a range of diseases.

7. References

1. Altman, M.C. *et al*. Development of a fixed module repertoire for the analysis and interpretation of blood transcriptome data. Nat Commun. 2021 Jul 19;12(1):4385.

2. Rinchai, D. *et al*. BloodGen3Module: Blood transcriptional module repertoire analysis and visualization using R. Bioinformatics. 2021 Aug 25;37(16):2382-2389.

3. Rinchai, D. *et al*. Definition of erythroid cell-positive blood transcriptome phenotypes associated with severe respiratory syncytial virus infection. Clin Transl Med. 2020 Dec;10(8):e244.

4. Novershtern, N. et al. Densely interconnected transcriptional circuits control cell states in human hematopoiesis. Cell. 2011 Jan 21;144(2):296–309.

5. Linsley, P.S. et al. Copy number loss of the interferon gene cluster in melanomas is linked to reduced T cell infiltrate and poor patient prognosis. PloS One. 2014;9(10):e109760.

6. Rinchai, D. et al. A training curriculum for retrieving, structuring, and aggregating information derived from the biomedical literature and large-scale data repositories. [Internet]. F1000Research; 2022 [cited 2023 Mar 29]. Available from: https://f1000research.com/articles/11-994

7. Rinchai, D. et al. Assessing the potential relevance of CEACAM6 as a blood transcriptional biomarker [Internet]. F1000Research; 2022 [cited 2023 Mar 31]. Available from: https://f1000research.com/articles/11-1294
