# Supplementary material for: Harnessing large language models (LLMs) for candidate gene prioritization and selection
Source: J Transl Med. 2023 Oct 16;21:728. doi: 10.1186/s12967-023-04576-8 (PMC10580627; doi:10.1186/s12967-023-04576-8)
Supplement: Supplementary file 10 — Additional file 10: Is the prioritization report for module M15.53 authored by Darawan Rinchai. The file is shared in the PDF format. [file 12967_2023_4576_MOESM10_ESM.pdf]

M15.53 Prioritization report

Prepared by: Darawan Rinchai (Rockefeller University, New York, NY)

1. **Composition**

The module M15.53 belongs to Aggregate A37 from the BloodGen3 fixed module repertoire(1,2). This module aggregate is associated with circulating erythroid cells. It was found to be associated with RSV disease severity (3). We showed in the same study that the abundance of A27 transcripts is also elevated in the blood of patients with stage IV melanoma (3).

It comprises 25 genes: BRD4, C16ORF35, CDKL1, DPM2, EPB41, HMBS, HPS1, IQWD1, KLC3, LOC643008, LOC648434, LOC650898, MAP2K3, MICAL2, MICALCL, MXI1, RAB3IL1, RP11-529I10.4, SLC38A5, SLC6A8, TCP11L2, TGM2, TMPRSS9, TTC25, WNK1.

2. **Functional convergence**

**Functional Themes**	**Associated Genes**	**Comments**
Cell Structure and Integrity	EPB41, KLC3, MICAL2, MICALCL	**EPB41** (cell membrane of red blood cells) and **KLC3** (microtubule-based movement) relate to cell structure and its components. **MICAL2** and **MICALCL**: These are monooxygenases that act on various cytoskeletal proteins, leading to their depolymerization. They clearly relate to **Cell Structure and Integrity**.
Biosynthesis pathways	DPM2, HMBS, TGM2	Both **DPM2** (glycoprotein biosynthesis) and **HMBS** (heme biosynthesis) are involved in biosynthetic processes. **TGM2** (Transglutaminase 2): This enzyme catalyzes the crosslinking of proteins and the conjugation of polyamines to proteins. It plays a role in **Biosynthesis Pathways** and is also related to cell structural functions.
Cell cycle regulation	BRD4, CDKL1, MAP2K3, MXI1	Both **BRD4** and **CDKL1** have potential roles in cell cycle regulation. **MAP2K3**: A kinase that plays a role in the MAP kinase signaling pathway, specifically activating p38 MAP kinase. It could be loosely associated with **Cell Cycle Regulation** due to its involvement in cell signaling. **MXI1**: Acts as a transcriptional repressor and has been associated with the regulation of the cell cycle and cellular differentiation. It fits under **Cell Cycle Regulation**.

3. **Scoring and prioritization**

Genes were scored on six criteria using GPT-4 and Claude. The scores were averaged, and candidate genes ranked according to their cumulative scores (**Figure 1**, Methods: Step 3 and Step 4).

The two LLMs were requested to score each gene on the following six statements:
a. The gene is associated with erythroid cells or erythropoiesis.
b. The gene is currently being used as a biomarker in clinical settings.
c. The gene has potential value as a blood transcriptional biomarker.
d. The gene is relevant to circulating leukocytes immune biology.
e. The gene is a known drug target.
f. The gene is therapeutically relevant for immune-mediated diseases.

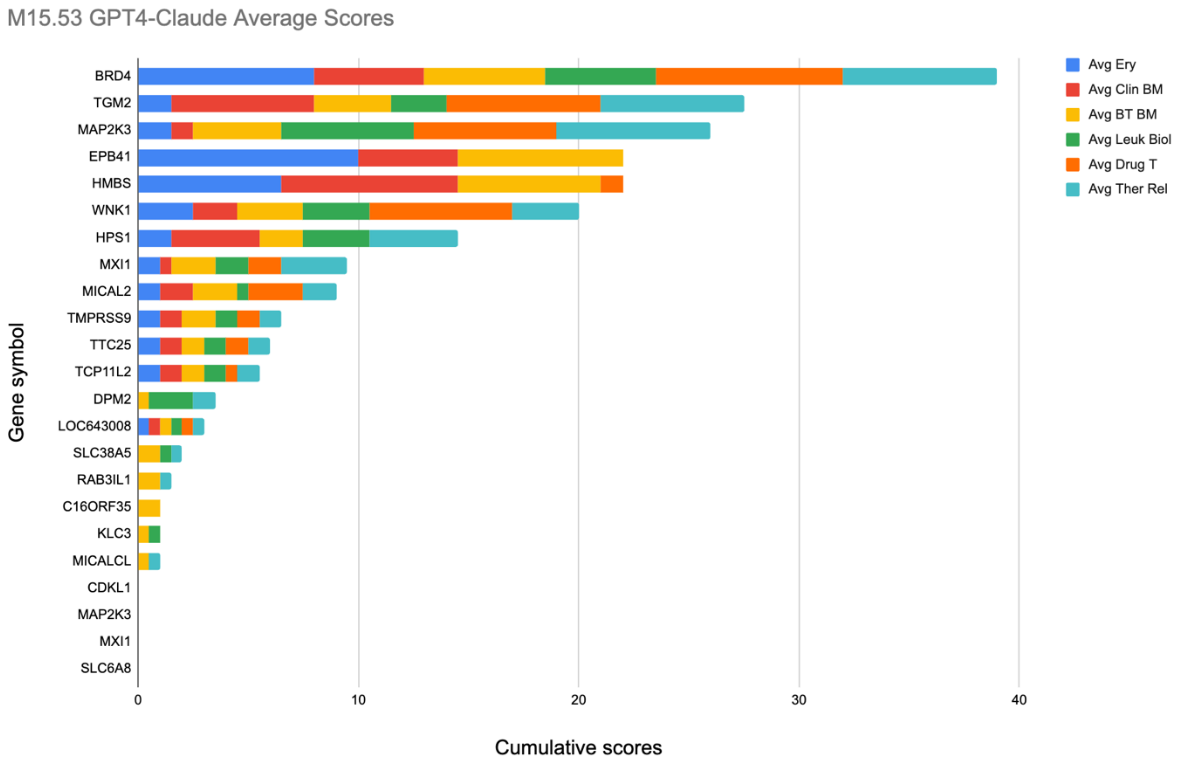

Figure 1: The stacked bar graph shows cumulative scores across six criteria for the 25 M15.53 genes.

The top five genes selected for further evaluation were: TGM2, MAP2K3, EPB41, HMBS and BRD4 **(Figure 2**).

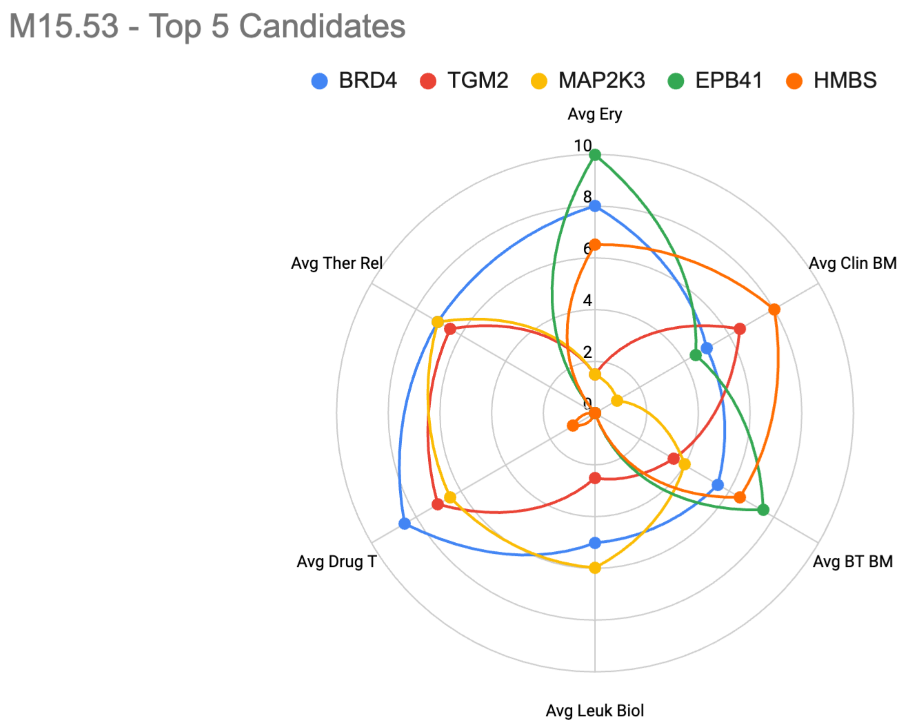

Figure 2: The radar plot represents individual scores for the 5 top scoring genes: TGM2, MAP2K3, EPB41, HMBS and BRD4.

4. **Knowledge-driven evaluation of top five candidates**

Justifications given by the GPT-4 and Claude for the scores provided across the 6 criteria were compiled and summarized by GPT-4 (Methods: Step 5). For each statement pertinent references were retrieved using GPT-4 or Claude, with the relevance of each reference checked and attributed manually by researcher authoring this report.

Function: The BRD4 gene encodes a member of the bromodomain and extra-terminal (BET) family of proteins, critical for transcription regulation through interaction with acetylated chromatin and involved in cell cycle progression, erythropoiesis, and cancer (4,5). TGM2 encodes a calcium-dependent enzyme, transglutaminase 2, responsible for protein cross-linking and involved in various cellular processes such as tissue repair, cell death regulation, and extracellular matrix stabilization; its aberrant activation is associated with celiac disease and neurodegenerative disorders(6–8). MAP2K3 encodes a dual specificity protein kinase that activates the p38 MAP kinase, playing a role in cellular responses to stress and cytokines, as well as in cell proliferation and differentiation (9,10). EPB41 encodes protein 4.1, a structural component of the red blood cell cytoskeleton, which is essential for regulating cell shape, flexibility, and membrane stability (11,12). HMBS encodes hydroxymethylbilane synthase, also known as porphobilinogen synthase, which catalyzes a key step in heme biosynthesis, essential for oxygen transport in blood cells (13).

a. Relevance to erythroid cells and erythropoiesis: BRD4 plays a pivotal role in erythropoiesis, specifically in terminal erythroid differentiation (14,15). It is highly expressed during erythroid maturation and regulates key erythroid transcription factors such as GATA1 and KLF1 (16,17). In contrast, while TGM2 is ubiquitously expressed across various cell types (18), there is no direct evidence to suggest its specialized role in erythroid cells or erythropoiesis(19). Similarly, MAP2K3, despite its broad role in cellular stress responses and proliferation (20), lacks specific evidence pointing to its involvement in erythroid development. EPB41, however, is intrinsically related to erythroid cells, as it is highly expressed in the erythroid lineage and serves as a critical structural protein in erythrocyte membranes (11,21). HMBS, though involved in heme biosynthesis (22), which is essential for hemoglobin function in red blood cells, is ubiquitously expressed (23) and has not been directly linked to erythroid cells or erythropoiesis.

b. Is used as a clinical biomarker: As of the latest information available, BRD4 is emerging as a potential biomarker in clinical settings, particularly for some types of cancer (5). Its inhibition has been shown to downregulate NF-kB-mediated inflammatory pathways and reduce cytokine production (24), making it a target for clinical trials involving BET inhibitors like JQ1 for autoimmune diseases (25). TGM2, while not a broad clinical biomarker, has been investigated for its role in celiac disease (26). MAP2K3 is not widely recognized as a clinical biomarker, but alterations in its signaling pathways have been implicated in specific diseases (20). EPB41 is not generally used as a clinical biomarker, but mutations in the gene are linked to hereditary elliptocytosis (21), and reduced levels may serve as an indicator for certain hemolytic anemias (27). HMBS mutations are clinically relevant for diagnosing a subset of porphyrias, specifically acute intermittent porphyria, where its enzyme activity is used for diagnostic purposes (28).

c. Potential relevance as a blood transcriptional biomarker: BRD4 shows preliminary evidence of being a valuable blood transcriptional biomarker in cancer (5) and could also serve to assess erythropoiesis status, as it regulates key erythroid genes and its expression levels may reflect erythroid progenitor cell activity (15). TGM2 also holds promise as a potential blood transcriptional biomarker, particularly in the context of celiac disease (26), although further research is needed for confirmation. MAP2K3, given its role in cellular stress response (20), could likewise serve as a blood transcriptional biomarker, pending additional research. EPB41, due to its critical role in red blood cell integrity (21), has potential value as a blood transcriptional biomarker that could provide insights into erythropoiesis status based on its expression levels. Finally, HMBS could be valuable as a blood transcriptional biomarker specifically for diagnosing acute intermittent porphyria (28), and its enzyme levels may indicate erythropoietic activity and the rate of hemoglobin production.

d. Relevance to leukocytes immune biology: BRD4 has been implicated in the regulation of inflammatory responses (25), including the function of macrophages, a type of leukocyte (29). However, its role in leukocytes is not well-defined, and there is limited evidence to consider it as a leukocyte-specific biomarker. TGM2 is relevant to immune biology due to its roles in apoptosis (30) and cellular adhesion (31), which can affect

leukocyte function. TGM2 inhibitors are under development for treating conditions like fibrosis (32), cancer (33), and neurodegeneration (8). MAP2K3 is implicated in inflammatory responses (20), suggesting a role in leukocyte biology, although specific functions are yet to be fully elucidated. In contrast, EPB41 and HMBS are not directly relevant to circulating leukocytes' immune biology. EPB41 is specific to erythroid cells, and no evidence suggests its relevance to leukocytes (11). HMBS has a general housekeeping role and is not specifically relevant to circulating leukocytes

e. <u>Is a known drug target</u>: BRD4 is a well-recognized drug target with multiple inhibitors such as JQ1 and OTX015 in pre-clinical and clinical development for cancer treatment(34). BET inhibitors targeting BRD4 are also under investigation for potential applications in inflammation and cardiovascular disease (25). TGM2 has been proposed as a potential drug target due to its involvement in various pathological conditions, including neurodegenerative diseases (8) and cancer (35). While TGM2 is linked to inflammatory conditions like celiac disease, its specific role in autoimmunity remains unclear(26). MAP2K3 is another potential drug target being explored for its role in the MAPK pathway(20), commonly dysregulated in cancer. It also regulates proinflammatory signaling pathways in immune cells and may be a target for immune-mediated diseases (36). Conversely, EPB41 and HMBS are not major drug targets. Although drugs inducing HMBS expression could potentially treat acute porphyrias (28), it is not considered a primary drug target. No evidence indicates that EPB41 serves as a drug target.

f. <u>Potential therapeutic relevance for immune-mediated diseases:</u> BRD4 is therapeutically pertinent to immune-mediated diseases due to its role in regulating inflammatory responses (25). Studies indicate that BET inhibitors (37), which target BRD4, are under investigation for potential treatment of immune disorders such as SLE and multiple sclerosis. In a different context, TGM2 is associated with immune-mediated diseases, notably in celiac disease (26), where the immune response to this enzyme is crucial to the disease's development. While TGM2's involvement in inflammatory conditions like celiac disease is established, its precise function in autoimmunity remains undefined. MAP2K3 has emerged as another molecule of interest in the realm of immune-mediated diseases because of its influence on inflammatory responses (20). There's strong evidence to suggest that MAP2K3 plays a role in modulating proinflammatory signaling pathways in immune cells, positioning it as a prospective target for therapeutic interventions (36). However, no therapeutic relevance has been identified for EPB41 in the context of immune-mediated diseases.

5. Examining expression patterns of top 5 candidates across leukocyte populations

The expression patterns of the top 5 candidate genes were examined across diverse leukocyte populations and hematopoietic precursors using two reference transcriptomic datasets.

5.1 <u>Densely interconnected transcriptional circuits control cell states in human hematopoiesis</u> – <u>(GSE24759)</u>
http://developmentalimmunology.gxbsidra.org/dm3/geneBrowser/show/4000026

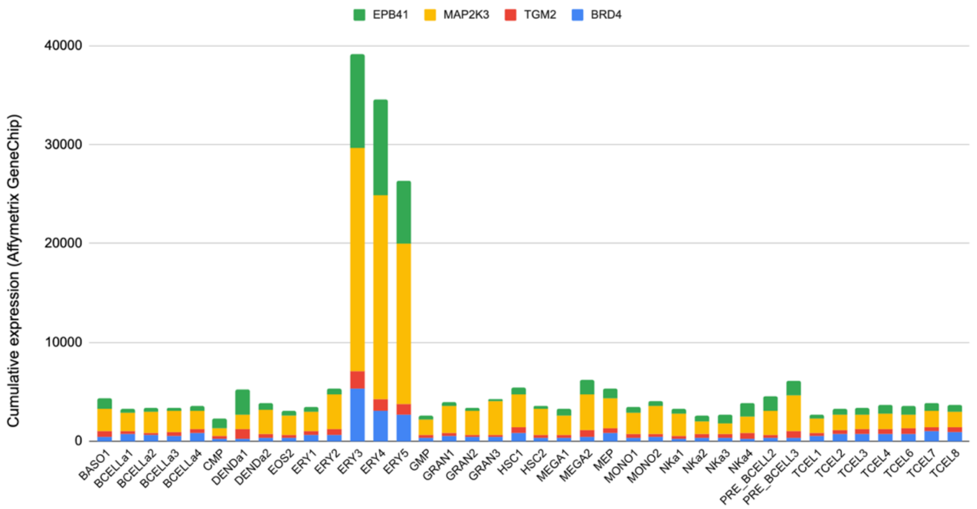

Figure 3: This stacked bar graph shows the expression levels of five candidate genes. ERY3-5 populations are CD71+ GLYA+ erythroid cells. The original article provides full experimental details: (38). The web link above also provides access to more complete information about this dataset as well as individual gene profiles.

5.2 <u>Next generation sequencing of human immune cell subsets across diseases – (GSE60424)</u>
http://cd2k.gxbsidra.org/dm3/geneBrowser/show/4000098

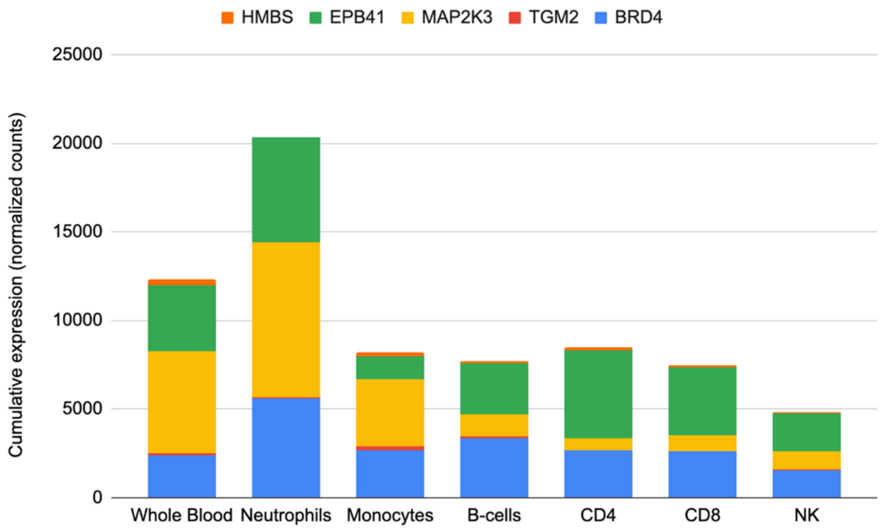

Figure 4: This stacked bar graph shows the expression levels measured by RNAseq of the five candidate genes across six leukocyte populations and whole blood. The abundance is shown for each gene in each population for an average of up to 20 samples. Additional experimental details are accessible via the link provided above and is available as part of this sample series GEO record ([GSE60424](GSE60424)) (39).

6. BloodGen3 reference cohorts (GSE100150)

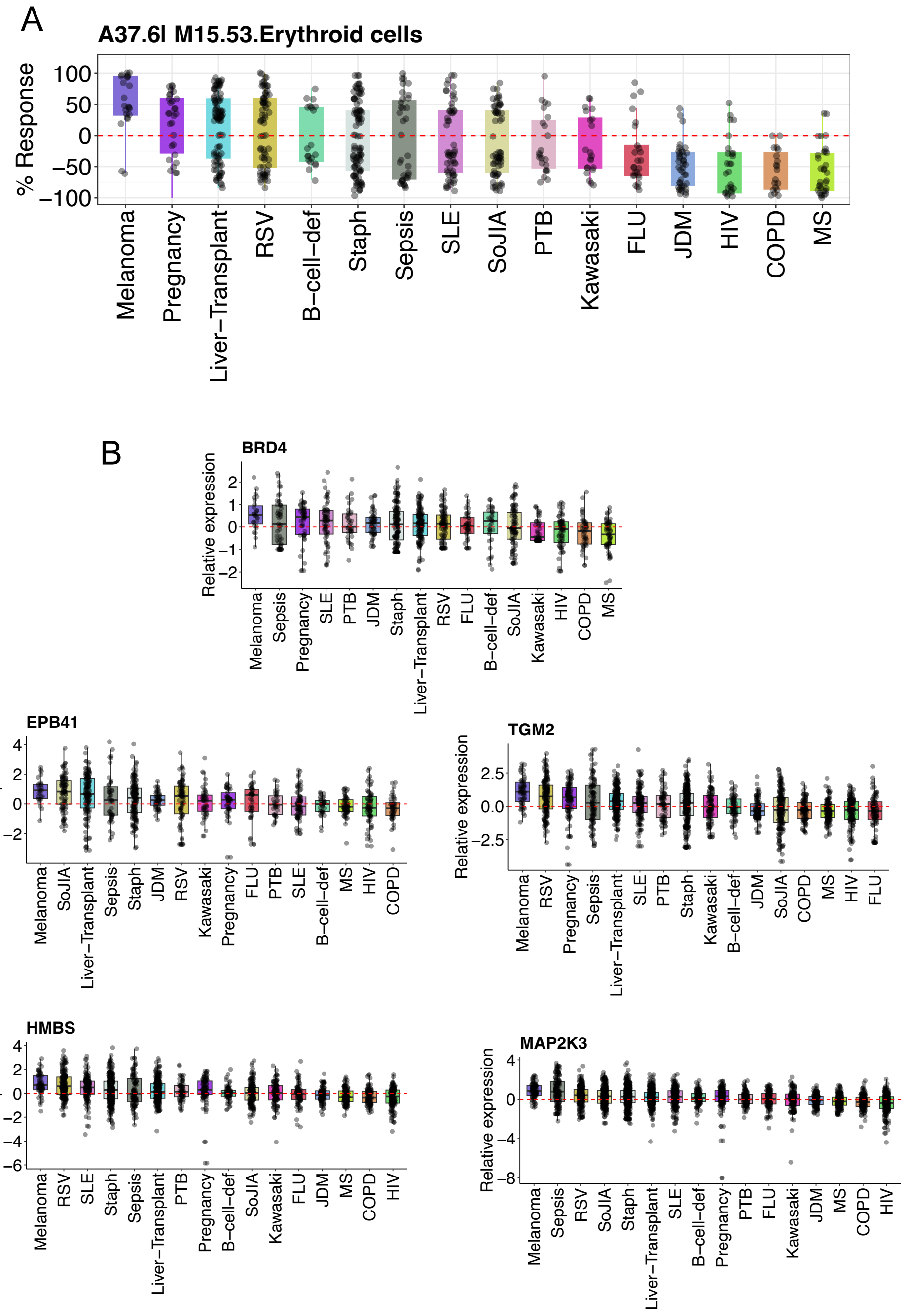

Figure 5: The box plot in panel A shows the changes in abundance of transcripts comprising the M13.30 module, expressed within a cohort as the percentage of genes showing differential expression between each patient and the respective set of control subjects for this cohort. Differential expression is based on fixed cut-off (Log2 Fold Change >1.5, difference>10). These values are expressed as % module activity and can range from 100% (all genes are upregulated) to -100% (all genes are downregulated). The box plot in panel B show expression levels for the top 5 scoring M15.74 genes in patient cohorts relative to respective control subjects matched for demographic variables.

7. Overall pick(s) for M15.53

We aim to establish a universal immune profiling assay that measures transcript abundance of a panel of genes in whole blood using small- or meso-scale molecular profiling technology. Compelling candidates identified here from the M15.53 selection pool will be subjected to more in-depth evaluation using a pre-established workflow (40,41).

Claude and GPT-4 were prompted to select a top candidate on the basis of the information summarized above in section 4, taking into account all six scoring criteria equally (Methods: Step 6).

<u>Claude chose BRD4</u>:

"Based on the provided criteria, I recommend BRD4 as the top candidate gene for inclusion in the targeted blood transcriptional profiling panel. The key reasons are:

- BRD4 plays a critical role in erythropoiesis and is highly expressed during erythroid maturation. This makes it very relevant to erythroid cells and erythropoiesis.

- BRD4 is emerging as a potential biomarker in cancer and shows promise as a blood transcriptional biomarker that could provide insights into erythropoiesis status.

- BRD4 regulates inflammatory pathways and macrophage function, suggesting relevance to leukocyte immune biology, although its specific role requires further research.

- BRD4 is an established drug target with multiple inhibitors under development for cancer, inflammation, and cardiovascular disease, making it therapeutically relevant.

While TGM2, MAP2K3, EPB41, and HMBS have merits in some criteria, BRD4 best satisfies the provided criteria collectively as a biomarker reflective of erythroid biology with relevance to immune function and therapeutic targeting. The evidence highlights its potential value as a representative biomarker on a targeted transcriptional profiling panel intended for the stated applications."

GPT-4 chose BRD4:
"Based on the extensive information provided and weighing each of the six criteria equally, the BRD4 gene appears to be the top candidate gene to include in a targeted blood transcriptional profiling panel for the following reasons:

1. Relevance to erythroid cells and erythropoiesis: BRD4 plays a key role in erythropoiesis, specifically in terminal erythroid differentiation. It regulates crucial erythroid transcription factors such as GATA1 and KLF1 and is highly expressed during erythroid maturation.
2. Current use as a biomarker: BRD4 is emerging as a biomarker in clinical settings, particularly for some types of cancer. It also shows potential for tracking erythropoiesis status based on its high expression during erythroid maturation.
3. Potential use as a blood transcriptional biomarker: Given its role in erythropoiesis and cancer, BRD4 could serve as a valuable blood transcriptional biomarker, providing both a measure of erythroid activity and indications for specific disease states like cancer.
4. Relevance to leukocyte immune biology: Although not as strongly tied to leukocytes as some other genes, BRD4 has been implicated in inflammatory responses and in the function of macrophages, a type of leukocyte. This suggests potential relevance to immune biology.
5. Status as a known drug target: BRD4 is a well-known drug target, with inhibitors like JQ1 and OTX015 in pre-clinical and clinical development. It is also targeted for potential applications in inflammation and cardiovascular diseases.
6. Therapeutic relevance for immune-mediated diseases: Its inhibition has been shown to downregulate NF-kB-mediated inflammatory pathways and reduce cytokine production, which has led to its inclusion in clinical trials involving BET inhibitors for autoimmune diseases.

In summary, BRD4 satisfies all six criteria to varying degrees but is particularly strong in its relevance to erythroid cells and erythropoiesis, its emerging status as a clinical biomarker, its potential as a blood transcriptional biomarker, and its well-established role as a drug target. Therefore, BRD4 would likely provide the most comprehensive and relevant insights in a targeted blood transcriptional profiling panel based on your criteria."

7. Overall pick for M15.53 based on summarized justifications and reference transcriptional datasets.

Through multi-tier prompting, GPT-4 and Claude were requested to select a top candidate, based on the fact-checked justifications summaries generated earlier, but this time also taking into account the transcriptional profiles from the three reference datasets described above.

This was achieved using multiple prompts. This strategy was successful when using Claude but we GPT-4 could not follow our directives from start to finish when presented with large amounts of textualized transcriptional data.

Prompts	CLAUDE	GPT-4

PROMPT 2: Now that you have provided an initial recommendation based on the summary, take into account the following RNA-sequencing expression data from key immune cell types and whole blood samples: *[input textualized transcriptional data].* Given this additional RNA-seq expression data, does your recommended gene selection remain the same? Please explain which gene you would recommend at this stage and why, incorporating both the summarized information *provided earlier and RNA-seq data in your rationale."*	After reviewing the provided RNA-seq expression data, I would still recommend BRD4 as the top candidate gene for inclusion in the targeted blood transcriptional profiling panel.<br><br>The key reasons are:<br><br>- BRD4 shows robust expression across all analyzed immune cell types - neutrophils, monocytes, B cells, T cells, NK cells - as well as in whole blood samples. This indicates it is broadly expressed and detectable in circulating immune cells.<br><br>- The expression levels of BRD4 are substantially higher than those of TGM2 and HMBS across all the analyzed samples. TGM2 and HMBS have very low expression, especially in T cells.<br><br>- BRD4 has higher or comparable expression to MAP2K3 and EPB41 in most immune cell types profiled. MAP2K3 shows higher expression in neutrophils while EPB41 is higher in T cells.<br><br>- However, BRD4 remains the most relevant to erythroid biology based on its established role in erythropoiesis and erythroid gene regulation.<br><br>In summary, the RNA-seq data reinforces BRD4 as a suitable biomarker detectable	The RNA-seq expression data adds a valuable dimension to our understanding of the expression profile of these genes in key immune cell types and whole blood. Given this data, I would recommend MAP2K3 as the top candidate gene to include in a targeted blood transcriptional profiling panel for the following reasons:<br>**Relevance to erythroid cells and erythropoiesis**: While MAP2K3 isn't erythroid-specific, it plays a role in cellular responses to stress and cytokines, as well as in cell proliferation and differentiation. It may not be directly involved in erythropoiesis but could still provide valuable insights into the status of immune and blood cells in various conditions.<br>**Current use as a biomarker**: MAP2K3 may not be widely recognized as a clinical biomarker, but its alteration has been implicated in specific diseases. Given its high expression across multiple immune cell types, it may offer potential as a broad clinical biomarker.<br>**Potential use as a blood transcriptional biomarker**: MAP2K3 shows high RNA expression in human

	across diverse immune cell populations. Combined with the previous evidence of its erythroid function and therapeutic relevance, BRD4 is still the top recommendation for inclusion in the intended transcriptional profiling panel. The expression data further supports its potential utility as a representative biomarker reflective of both erythroid and broader immune biology.	whole blood and across various immune cell types, indicating it could serve as a robust blood transcriptional biomarker, especially for immune-mediated conditions.<br>**Relevance to leukocyte immune biology**: MAP2K3 is implicated in inflammatory responses and shows high RNA-seq counts across multiple leukocyte types, making it relevant to immune biology. This is supported by its high expression in neutrophils and monocytes, key immune cells involved in inflammatory responses.<br>**Status as a known drug target**: MAP2K3 is being explored as a potential drug target, particularly in its role in the MAPK pathway, which is commonly dysregulated in cancer. Its regulation of proinflammatory signaling pathways in immune cells may also make it a target for immune-mediated diseases.<br>**Therapeutic relevance for immune-mediated diseases**: Though not yet established, given its involvement in the MAPK pathway and proinflammatory signaling, MAP2K3 has potential therapeutic relevance for immune-mediated diseases.<br>**Comparison with BRD4**:

		The RNA-seq data show that BRD4 is expressed at a lower level than MAP2K3 in whole blood and across all tested immune cell types. Although BRD4 has substantial relevance to erythroid cells and is an emerging clinical biomarker, its lower expression in key immune cell types compared to MAP2K3 may limit its utility as a representative signature for leukocyte biology in the blood transcriptional profiling panel.<br>In summary, given the high expression levels of MAP2K3 across multiple immune cell types and its broader relevance to immune biology, MAP2K3 emerges as a strong candidate when considering both the summarized information and the RNA-seq data. This selection offers the potential for both clinical utility and mechanistic insights into immune-mediated diseases, erythropoiesis, and other key physiological processes.

PROMPT 3: Now that you have provided an initial recommendation based on the summary and the RNAseq data, take into account the following microarray expression data from key immune cell types and hematopoietic precursors: *[input textualized transcriptional data]*. Given this additional microarray expression data, does your recommended gene selection remain the same? Please explain which gene you would recommend at this stage and why, incorporating the summarized information and RNA-seq data provided earlier, as well as this new microarray data in your rationale.	After reviewing the extensive microarray expression data, I still recommend BRD4 as the top candidate gene for inclusion in the targeted transcriptional profiling panel.<br><br>The key reasons are:<br><br>- BRD4 continues to show broad expression across diverse hematopoietic cells, immune cells, and erythroid precursor populations in the microarray data.<br><br>- Critically, BRD4 displays high expression in late-stage erythroid precursors like CD34-CD71+GlyA+ cells, validating its erythroid relevance.<br><br>- The microarray data also confirms higher BRD4 expression compared to TGM2 and HMBS in most analyzed populations.<br><br>- While MAP2K3 and EPB41 show comparable or higher expression in certain lineages, BRD4 remains most relevant to erythroid biology and therapeutic targeting based on previous evidence.<br><br>- The RNA-seq data previously highlighted BRD4's detectability across key immune cells, which is reinforced by the microarray patterns.<br><br>In summary, the combined RNA-seq and microarray	*[N/A: input exceed GPT-4 limits. Attempts to break down inputting the textualized data were unsuccessful as GPT-4 lost the thread of the conversation. Thus, we chose to skip to the next prompt]*

	datasets confirm BRD4 as a biomarker with broad expression in immune and erythroid populations, supporting its potential utility as a representative biomarker reflective of both lineages. Given its regulatory roles in erythropoiesis and therapeutic relevance, BRD4 is still the top recommendation despite comparable detection of some other genes in specific cell subsets.	

PROMPT 4: Now that you have provided an initial recommendation based on the summary and the immune cells RNAseq and microarray data, take into account the following averaged log2 fold changes in RNA abundance in patient cohorts compared to controls. *[input textualized transcriptional data].* Given this additional data, does your recommended gene selection remain the same? Please explain which gene you would recommend at this stage and why, incorporating the summarized information, the leukocytes RNA-seq and microarray data provided earlier, as well as these new patient cohort profiles in your rationale.	After reviewing the log2 fold change data across multiple patient cohorts, I still recommend BRD4 as the top candidate gene.<br><br>The key reasons are:<br><br>- BRD4 shows differential expression across many disease states relevant to the intent of the panel, including HIV, sepsis, autoimmunity, and cancer. This indicates its potential utility as a biomarker.<br><br>- The magnitude of fold changes for BRD4 are generally higher than for TGM2, MAP2K3, EPB41, and HMBS in diseases like HIV, cancer, and autoimmunity.<br><br>- BRD4 upregulation is seen in cancer and some inflammatory conditions, in line with its role in regulating cell growth/inflammatory pathways.<br><br>- Downregulation of BRD4 in diseases like HIV and MS reinforce its broader immunologic relevance.<br><br>- While EPB41 and HMBS show large fold changes in some cohorts, their biological significance is unclear, whereas BRD4 changes align with its functions.<br><br>- Critically, BRD4 remains the strongest candidate in relation to erythroid biology based on earlier evidence.	

	In summary, the patient cohort data provides further evidence that BRD4 is differentially expressed in relevant disease states and could serve as an informative biomarker reflective of both erythroid and immune activity. When incorporated with the earlier findings, BRD4 emerges as the most suitable candidate for inclusion in the intended transcriptional profiling panel.	

PROMPT 5: Could you please summarize the key conclusions you have drawn from the conversation so far?	Based on the provided data and our discussion, here are the key conclusions:<br><br>- BRD4 emerges as the top recommended gene for inclusion in the targeted transcriptional profiling panel intended to reflect erythroid and immune biology.<br><br>- Supporting evidence includes BRD4's established regulatory roles in erythropoiesis, its expression patterns across diverse hematopoietic lineages, and its therapeutic relevance as a drug target.<br><br>- The RNA-seq and microarray data highlighted BRD4's detectability across key immune cell populations and erythroid precursors.<br><br>- Differential expression of BRD4 in multiple disease cohorts further reinforces its potential utility as an informative biomarker.<br><br>- Comparable candidates like MAP2K3 and EPB41 show lineage-specific expression strengths, but BRD4 is most aligned with the criteria when considered collectively.<br><br>- In summary, BRD4 emerges as a suitable representative biomarker that could provide insights into both erythroid and immune status based on the presented evidence.	

7. References

1. Altman MC, Rinchai D, Baldwin N, Toufiq M, Whalen E, Garand M, et al. Development of a fixed module repertoire for the analysis and interpretation of blood transcriptome data. Nat Commun. 2021 Jul 19;12(1):4385.

2. Rinchai D, Roelands J, Toufiq M, Hendrickx W, Altman MC, Bedognetti D, et al. BloodGen3Module: blood transcriptional module repertoire analysis and visualization using R. Bioinforma Oxf Engl. 2021 Aug 25;37(16):2382–9.

3. Rinchai D, Altman MC, Konza O, Hässler S, Martina F, Toufiq M, et al. Definition of erythroid cell-positive blood transcriptome phenotypes associated with severe respiratory syncytial virus infection. Clin Transl Med. 2020 Dec;10(8):e244.

4. Lamonica JM, Deng W, Kadauke S, Campbell AE, Gamsjaeger R, Wang H, et al. Bromodomain protein Brd3 associates with acetylated GATA1 to promote its chromatin occupancy at erythroid target genes. Proc Natl Acad Sci U S A. 2011 May 31;108(22):E159-168.

5. Filippakopoulos P, Qi J, Picaud S, Shen Y, Smith WB, Fedorov O, et al. Selective inhibition of BET bromodomains. Nature. 2010 Dec 23;468(7327):1067–73.

6. Elli L, Bergamini CM, Bardella MT, Schuppan D. Transglutaminases in inflammation and fibrosis of the gastrointestinal tract and the liver. Dig Liver Dis Off J Ital Soc Gastroenterol Ital Assoc Study Liver. 2009 Aug;41(8):541–50.

7. Griffin M, Casadio R, Bergamini CM. Transglutaminases: nature's biological glues. Biochem J. 2002 Dec 1;368(Pt 2):377–96.

8. Jeitner TM, Matson WR, Folk JE, Blass JP, Cooper AJL. Increased levels of gamma-glutamylamines in Huntington disease CSF. J Neurochem. 2008 Jul;106(1):37–44.

9. Cuadrado A, Nebreda AR. Mechanisms and functions of p38 MAPK signalling. Biochem J. 2010 Aug 1;429(3):403–17.

10. Raingeaud J, Gupta S, Rogers JS, Dickens M, Han J, Ulevitch RJ, et al. Pro-inflammatory cytokines and environmental stress cause p38 mitogen-activated protein kinase activation by dual phosphorylation on tyrosine and threonine. J Biol Chem. 1995 Mar 31;270(13):7420–6.

11. An X, Mohandas N. Disorders of red cell membrane. Br J Haematol. 2008 May;141(3):367–75.

12. Salomao M, Zhang X, Yang Y, Lee S, Hartwig JH, Chasis JA, et al. Protein 4.1R-dependent multiprotein complex: new insights into the structural organization of the red blood cell membrane. Proc Natl Acad Sci U S A. 2008 Jun 10;105(23):8026–31.

13. Bonkovsky HL, Guo JT, Hou W, Li T, Narang T, Thapar M. Porphyrin and heme metabolism and the porphyrias. Compr Physiol. 2013 Jan;3(1):365–401.

14. Shi J, Wang Y, Zeng L, Wu Y, Deng J, Zhang Q, et al. Disrupting the interaction of BRD4 with diacetylated Twist suppresses tumorigenesis in basal-like breast cancer. Cancer Cell. 2014 Feb 10;25(2):210–25.

15. Stonestrom AJ, Hsu SC, Jahn KS, Huang P, Keller CA, Giardine BM, et al. Functions of BET proteins in erythroid gene expression. Blood. 2015 Apr 30;125(18):2825–34.

16. Dawson MA, Bannister AJ, Göttgens B, Foster SD, Bartke T, Green AR, et al. JAK2 phosphorylates histone H3Y41 and excludes HP1alpha from chromatin. Nature. 2009 Oct 8;461(7265):819–22.

17. Huang Z, Richmond TD, Muntean AG, Barber DL, Weiss MJ, Crispino JD. STAT1 promotes megakaryopoiesis downstream of GATA-1 in mice. J Clin Invest. 2007 Dec;117(12):3890–9.

18. Iismaa SE, Mearns BM, Lorand L, Graham RM. Transglutaminases and disease: lessons from genetically engineered mouse models and inherited disorders. Physiol Rev. 2009 Jul;89(3):991–1023.

19. Gundemir S, Colak G, Tucholski J, Johnson GVW. Transglutaminase 2: a molecular Swiss army knife. Biochim Biophys Acta. 2012 Feb;1823(2):406–19.

20. Cuenda A, Rousseau S. p38 MAP-kinases pathway regulation, function and role in human diseases. Biochim Biophys Acta. 2007 Aug;1773(8):1358–75.

21. Bennett V, Baines AJ. Spectrin and ankyrin-based pathways: metazoan inventions for integrating cells into tissues. Physiol Rev. 2001 Jul;81(3):1353–92.

22. Jordan PM. The biosynthesis of uroporphyrinogen III: mechanism of action of porphobilinogen deaminase. Ciba Found Symp. 1994;180:70–89; discussion 89-96.

23. Grandchamp B, Phung N, Nordmann Y. The mitochondrial localization of coproporphyrinogen III oxidase. Biochem J. 1978 Oct 15;176(1):97–102.

24. Hogg SJ, Vervoort SJ, Deswal S, Ott CJ, Li J, Cluse LA, et al. BET-Bromodomain Inhibitors Engage the Host Immune System and Regulate Expression of the Immune Checkpoint Ligand PD-L1. Cell Rep. 2017 Feb 28;18(9):2162–74.

25. Nicodeme E, Jeffrey KL, Schaefer U, Beinke S, Dewell S, Chung CW, et al. Suppression of inflammation by a synthetic histone mimic. Nature. 2010 Dec 23;468(7327):1119–23.

26. Dieterich W, Ehnis T, Bauer M, Donner P, Volta U, Riecken EO, et al. Identification of tissue transglutaminase as the autoantigen of celiac disease. Nat Med. 1997 Jul;3(7):797–801.

27. Delaunay J. The molecular basis of hereditary red cell membrane disorders. Blood Rev. 2007 Jan;21(1):1–20.

28. Puy H, Gouya L, Deybach JC. Porphyrias. Lancet Lond Engl. 2010 Mar 13;375(9718):924–37.

29. Belkina AC, Nikolajczyk BS, Denis GV. BET protein function is required for inflammation: Brd2 genetic disruption and BET inhibitor JQ1 impair mouse macrophage inflammatory responses. J Immunol Baltim Md 1950. 2013 Apr 1;190(7):3670–8.

30. Fesus L, Piacentini M. Transglutaminase 2: an enigmatic enzyme with diverse functions. Trends Biochem Sci. 2002 Oct;27(10):534–9.

31. Eckert RL, Kaartinen MT, Nurminskaya M, Belkin AM, Colak G, Johnson GVW, et al. Transglutaminase Regulation of Cell Function. Physiol Rev. 2014 Apr;94(2):383–417.

32. Johnson TS, Fisher M, Haylor JL, Hau Z, Skill NJ, Jones R, et al. Transglutaminase inhibition reduces fibrosis and preserves function in experimental chronic kidney disease. J Am Soc Nephrol JASN. 2007 Dec;18(12):3078–88.

33. Mangala LS, Fok JY, Zorrilla-Calancha IR, Verma A, Mehta K. Tissue transglutaminase expression promotes cell attachment, invasion and survival in breast cancer cells. Oncogene. 2007 Apr 12;26(17):2459–70.

34. Delmore JE, Issa GC, Lemieux ME, Rahl PB, Shi J, Jacobs HM, et al. BET bromodomain inhibition as a therapeutic strategy to target c-Myc. Cell. 2011 Sep 16;146(6):904–17.

35. Mangala LS, Fok JY, Zorrilla-Calancha IR, Verma A, Mehta K. Tissue transglutaminase expression promotes cell attachment, invasion and survival in breast cancer cells. Oncogene. 2007 Apr 12;26(17):2459–70.

36. Schett G, Zwerina J, Firestein G. The p38 mitogen-activated protein kinase (MAPK) pathway in rheumatoid arthritis. Ann Rheum Dis. 2008 Jul;67(7):909–16.

37. Mele DA, Salmeron A, Ghosh S, Huang HR, Bryant BM, Lora JM. BET bromodomain inhibition suppresses TH17-mediated pathology. J Exp Med. 2013 Oct 21;210(11):2181–90.

38. Novershtern N, Subramanian A, Lawton LN, Mak RH, Haining WN, McConkey ME, et al. Densely interconnected transcriptional circuits control cell states in human hematopoiesis. Cell. 2011 Jan 21;144(2):296–309.

39. Linsley PS, Speake C, Whalen E, Chaussabel D. Copy Number Loss of the Interferon Gene Cluster in Melanomas Is Linked to Reduced T Cell Infiltrate and Poor Patient Prognosis. PLOS ONE. 2014 Oct 14;9(10):e109760.

40. Rinchai D, Chaussabel D. Assessing the potential relevance of CEACAM6 as a blood transcriptional biomarker [Internet]. F1000Research; 2022 [cited 2023 Oct 4]. Available from: https://f1000research.com/articles/11-1294

41. Rinchai D, Chaussabel D. A training curriculum for retrieving, structuring, and aggregating information derived from the biomedical literature and large-scale data repositories. [Internet]. F1000Research; 2022 [cited 2023 Oct 4]. Available from: https://f1000research.com/articles/11-994
