# Supplementary material for: Harnessing large language models (LLMs) for candidate gene prioritization and selection
Source: J Transl Med. 2023 Oct 16;21:728. doi: 10.1186/s12967-023-04576-8 (PMC10580627; doi:10.1186/s12967-023-04576-8)
Supplement: Supplementary file 11 — Additional file 11: Is the prioritization report for module M15.74 authored by Darawan Rinchai. The file is shared in the PDF format. [file 12967_2023_4576_MOESM11_ESM.pdf]

M15.74 Prioritization report

Prepared by: Darawan Rinchai (The Rockefeller University, NY, USA)

1. Composition

The module M15.74 belongs to Aggregate A37 from the BloodGen3 fixed module repertoire (1,2). This module aggregate is associated with circulating erythroid cells. It was found to be associated with RSV disease severity (3). We showed in the same study that the abundance of A27 transcripts is also elevated in the blood of patients with stage IV melanoma (3).

It comprises 22 genes: ANKRD9, ATP6V0C, C22ORF25, C5ORF4, E2F2, FHL2, HMG2L1, LGALS3, LOC653907, MAP2K3, MARCH2, MCOLN1, MGC13057, PPM1A, PPM1A, RNF10, RNF11, SLC6A10P, SMOX, SRRD, TMEM158, UBE2H.

2. Functional convergence

Functional Themes	Associated Genes	Comments
Cell-cycle regulation	NKRD9, C22ORF25, E2F2, and FHL2	ANKRD9, C22ORF25, E2F2, and FHL2 have some potential overlap in terms of cell-cycle regulation, differentiation, and proliferation, but the exact functions of C22ORF25, C5ORF4, and HMG2L1 are not clearly defined, making any solid conclusions difficult. ATP6V0C seems to have quite different functions related to proton transport and acidification of intracellular compartments.
Cell cycle regulation, and cell proliferation	ANKRD9, E2F2, FHL2, LGALS3, MAP2K3, MARCH2, MCOLN1, and PPM1A	ANKRD9, E2F2, FHL2, LGALS3, MAP2K3, MARCH2, MCOLN1, and PPM1A, as these genes are involved in cellular processes like sig0l transduction, cell cycle regulation, and cell proliferation. However, there are still uncertainties due to unclear roles of several genes (C22ORF25, C5ORF4, HMG2L1, LOC653907, MGC13057). ATP6V0C's function related to proton transport and acidification of intracellular compartments appears distinct from the rest.
Cell cycle regulation, protein degradation, signal transduction, and apoptosis	E2F2, FHL2, MAP2K3, MARCH2, PPM1A, TMEM158, and UBE2H	The inclusion of UBE2H reinforces the convergence of functions related to cell cycle regulation, protein degradation, sig0l transduction, and apoptosis among several of these genes (E2F2, FHL2, MAP2K3, MARCH2, PPM1A, TMEM158, and UBE2H). It is crucial to note, however, that this conclusion is based on data available up until September 2021. For the most current and comprehensive a0lysis, consider employing bioinformatics tools or consulting the most recent scientific literature.
Proton transport and acidification of intracellular compartments	ATP6V0C	ATP6V0C's function related to proton transport and acidification of intracellular compartments appears distinct from the rest.

3. Scoring and prioritization

Genes were scored on six criteria using GPT4 and Claude. The scores were averaged, and candidate genes ranked according to their cumulative scores.

The two LLMs were requested to score each gene on the following six statements:
a. The gene is associated with erythroid cells or erythropoiesis.
b. The gene is currently being used as a biomarker in clinical settings.
c. The gene has potential value as a blood transcriptional biomarker.
d. The gene is relevant to circulating leukocytes immune biology.
e. The gene is a known drug target.
f. The gene is therapeutically relevant for immune-mediated diseases.

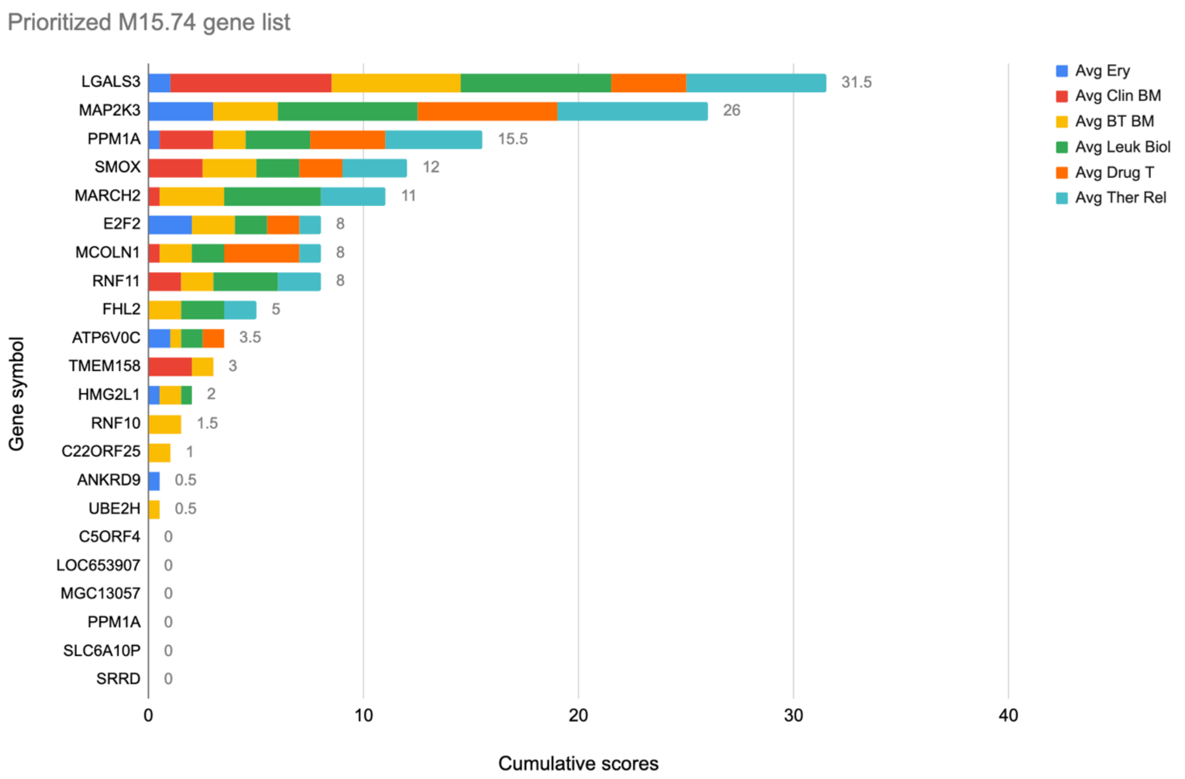

The top five genes selected for further evaluation were: LGALS3, MAP2K3, PPM1A, SMOX and MARCH2.

4. Knowledge-driven evaluation of top five candidates

Justifications given by the two models for the scores provided across the 6 criteria were compiled and summarized by GPT4 below. For each statement pertinent references were

retrieved, either using GPT4, with subsequently checking that the reference provided exists and is pertinent, or via a custom PubMed query.

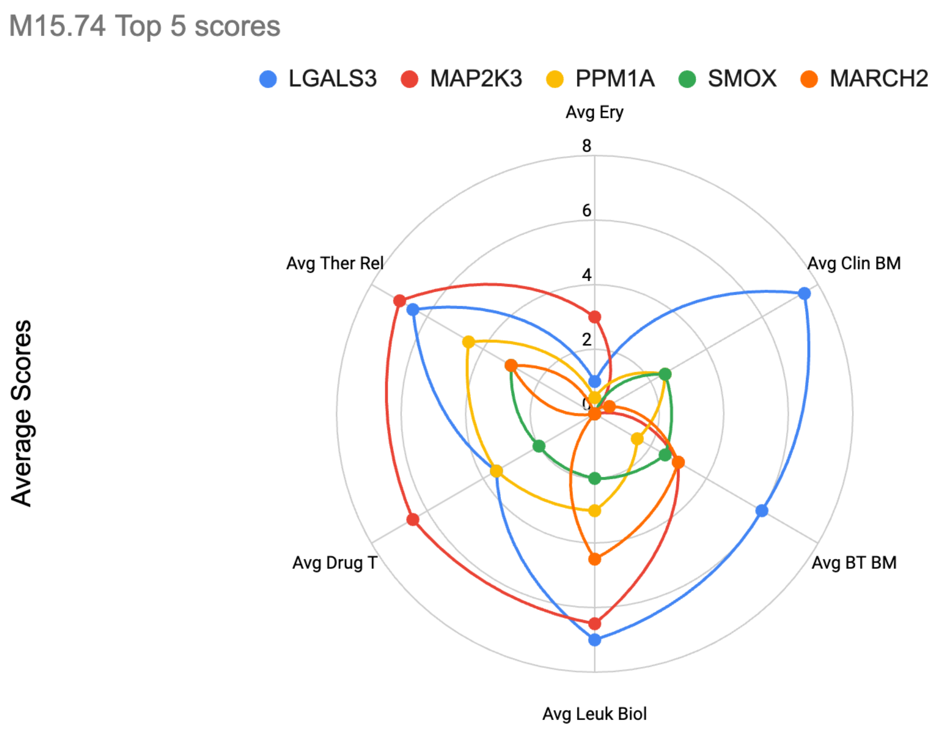

"Function: The LGALS3 gene encodes Galectin-3, a beta-galactoside binding lectin involved in cell-cell interactions, inflammation, and cancer progression, as well as cell activation, differentiation, and apoptosis (1,2). MAP2K3 is a dual specificity protein kinase that plays a key role in the MAP kinase signal transduction pathway, activating MAPK14/p38 in response to cellular stresses and cytokines, and is involved in cell proliferation and immune responses (3). PPM1A encodes a Mn2+/Mg2+-dependent serine/threonine protein phosphatase that negatively regulates kinases like p38 MAPK, JNK, and AMPK, and is implicated in cell cycle regulation and stress response (4). The SMOX gene is responsible for encoding spermine oxidase, an enzyme involved in polyamine metabolism and oxidative stress response, with aberrant activity linked to DNA damage, neurodegenerative diseases, and cancers (5). MARCH2 is a membrane-bound E3 ubiquitin ligase in the MARCH family that downregulates cell surface expression of MHC class II molecules, and its impairment may promote autoimmunity (6). Each of these genes plays a specialized role in cellular functions and disease pathology, ranging from

cell cycle regulation and apoptosis to immune responses and stress response mechanisms.

Association with erythroid cells or erythropoiesis: While the LGALS3, MAP2K3, PPM1A, SMOX, and MARCH2 genes are implicated in various cellular functions and disease pathways, there is limited to no evidence to suggest a direct association of these genes with erythroid cells or erythropoiesis. Specifically, LGALS3, broadly expressed, has not been conclusively linked to erythroid lineage or erythropoiesis. MAP2K3, known for its association with body mass index and hypothalamic inflammation in certain populations, likewise shows no direct evidence of involvement in erythroid cells (3). PPM1A, also broadly expressed, lacks clear evidence tying it to erythroid lineage. SMOX, while involved in cellular stress response and broadly expressed, has not been specifically linked to erythroid cells or erythropoiesis. Similarly, MARCH2, a gene in the MARCH family of E3 ubiquitin ligases, shows no strong evidence of a role in erythroid cells. Therefore, the current data do not support a specific role for these genes in erythroid cell function or erythropoiesis.

Used as a biomarker in clinical settings: Galectin-3, encoded by the LGALS3 gene, is currently utilized as a clinical biomarker, particularly in the context of heart failure and fibrosis progression in conditions like liver cirrhosis, as supported by studies such as "Galectin-3 in Cardiac Remodeling and Heart Failure" (7) (de Boer, R.A., 2020) and "Galectin-3: A biomarker and potential therapeutic target for liver cirrhosis and hepatocellular carcinoma" (8). In contrast, MAP2K3 is not widely used as a clinical biomarker and no specific references substantiate its clinical utility for this purpose. PPM1A has been investigated for its potential as a biomarker in certain types of cancer, such as lung and colorectal cancer, but it is not currently broadly applied in clinical settings, as indicated in studies like "PPM1A is a RelA phosphatase with tumor suppressor-like activity" (9). Similarly, SMOX has research suggesting its potential use as a cancer biomarker (10), but it is not generally employed in clinical scenarios. MARCH2 lacks evidence for its use as a clinical biomarker, with no specific references to support such an application.

Potential value as a blood transcriptional marker: The LGALS3 gene, encoding Galectin-3, holds promise as a blood transcriptional biomarker due to its established role in various disease processes such as inflammation and fibrosis (11). MAP2K3, although involved in immune cell signaling, requires further research for validation as a potential blood transcriptional or leukocyte biomarker. PPM1A, a phosphatase implicated in regulating cell signaling, could potentially serve as a marker correlated with kinase activity; however, it lacks sufficient validation for use as a blood transcriptional biomarker. SMOX, linked to reactive oxygen species (ROS) production, holds potential value as a blood transcriptional biomarker, but further research is required for validation. The utility of MARCH2 as a blood transcriptional biomarker remains uncertain; while it is linked to MHC class II regulation (12), more evidence is needed to validate its application as a biomarker. Overall, these genes display varying levels of promise as biomarkers, but further validation is essential for clinical applications.

Relevance to circulating leukocytes immune biology: Galectin-3, encoded by LGALS3, plays a crucial role in immune responses, particularly affecting leukocyte function. Studies such as "Galectins and their ligands: amplifiers, silencers or tuners of the inflammatory response?" (13) and "Critical role for galectin-3 in phagocytosis by macrophages" (14) have emphasized its role in the adhesion and extravasation of circulating leukocytes at inflammatory sites, establishing its relevance to leukocyte biology. MAP2K3 is also definitively relevant to the immune biology of circulating leukocytes, mediating signaling pathways and potentially regulating cytokine production (15). PPM1A, although implicated in the negative regulation of T-cell receptor signaling (16), lacks direct evidence for a specific role in circulating leukocytes. SMOX's relevance to the immune biology of circulating leukocytes is suggested by its role in inflammatory responses but requires further validation. MARCH2 also holds potential relevance; while it regulates MHC class II expression in antigen-presenting cells (17), and could be pertinent to leukocyte biology, the evidence supporting this notion is limited. Overall, these genes manifest differing levels of relevance to the immune biology of circulating leukocytes, with some requiring further research for definitive validation.

Is a known drug target:cLGALS3, encoding Galectin-3, is considered a potential drug target, especially in the contexts of cancer and fibrosis, as highlighted in (18). However, it is not yet an established drug target. MAP2K3 has been recognized as a drug target, particularly in inflammatory diseases (19), with p38 inhibitors targeting MAP Kinases currently in clinical trials for conditions like rheumatoid arthritis (20). PPM1A has been studied for its potential as a drug target, especially in certain types of cancers, although it is not yet established in this role (21). SMOX has also been suggested as a potential drug target in cancer-related research but lacks validation as an established drug target. MARCH2 has not been strongly identified as a drug target, and there is currently no evidence supporting its status as such. Thus, among these genes, MAP2K3 is the most substantiated as a drug target, while LGALS3, PPM1A, and SMOX remain potential candidates requiring further validation.

Of therapeutic relevance for immune-mediated diseases: The therapeutic relevance of LGALS3, MAP2K3, PPM1A, SMOX, and MARCH2 varies in the context of immune-mediated diseases. LGALS3, known for its role in immune responses and inflammation, shows promise as a therapeutic target for autoimmune conditions like rheumatoid arthritis (22). MAP2K3 is already recognized as a relevant target for immune-mediated diseases, backed by substantial evidence of its role in proinflammatory signaling pathways (23). PPM1A, with its involvement in T-cell receptor and TGF-beta signaling, could potentially be relevant for treating certain immune-mediated diseases, although further research is needed to establish its role (24). SMOX has limited evidence to suggest its therapeutic relevance in immune-mediated diseases, but ongoing research is examining its role in ROS-mediated inflammation (25). MARCH2, known for its regulation of MHC class II expression, has potential as a target in immune diseases, although its therapeutic relevance is not yet well-established (26). Overall, LGALS3 and MAP2K3 present as promising therapeutic targets, while PPM1A, SMOX, and MARCH2 require further validation.

5. **Pick(s) for M15.74 based on summarized justifications**

Claude and GPT-4 were prompted to select a top candidate on the basis of the information summarized above in section 4, taking into account all six scoring criteria equally. Both models chose SLC2A1 at the top candidate for M15.74.

GPT-4's justification: "Based on the information provided, none of the genes—LGALS3, MAP2K3, PPM1A, SMOX, and MARCH2—have strong evidence for a direct role in erythroid cells or erythropoiesis. Therefore, they may not be ideal candidates for inclusion in a targeted assay aimed at measuring differences in transcript abundance for a circulating erythroid cell signature.

However, if you are also interested in genes related to immune biology, inflammation, or stress responses in circulating cells, then some considerations could be made.

-LGALS3 (Galectin-3): This gene is already utilized as a clinical biomarker, particularly in the context of heart failure and fibrosis progression. It has well-established roles in immune responses and affects leukocyte function. Given its established clinical utility as a biomarker and its role in leukocyte biology, LGALS3 could be considered the top candidate for inclusion in a targeted assay if your focus extends beyond erythroid cells.

-MAP2K3: This gene is pivotal in immune cell signaling but is not widely used as a clinical biomarker. It could be of interest for studies related to immune cell function but might require more validation for clinical applications.

-PPM1A: This phosphatase is implicated in various signaling pathways but lacks sufficient evidence for a role in circulating leukocytes or erythroid cells. Its potential as a biomarker is also not well-established.

-SMOX: While involved in stress responses, it is not generally employed as a clinical biomarker and lacks clear evidence for a role in erythroid or leukocyte cells.

-MARCH2: It has limited evidence to suggest its relevance in immune biology and is not used as a clinical biomarker.

In summary, if the main aim is to measure differences in transcript abundance for a circulating erythroid cell signature, none of these genes appear to be ideal candidates based on the current data. However, if you are also interested in immune-related markers, LGALS3 seems to be the most promising gene for inclusion in a targeted assay, due to its well-established role in immune responses and its current clinical utility as a biomarker.

Claude's justification: Based on the information provided, I would recommend MAP2K3 as the top candidate gene for inclusion in an assay measuring differences in transcript abundance for a circulating erythroid cell signature.

The key reasons are:

- Neither MAP2K3 nor the other genes (LGALS3, PPM1A, SMOX, MARCH2) have strong evidence linking them directly to erythroid cells or erythropoiesis.

- However, MAP2K3 has an established role in immune cell biology and proinflammatory signaling pathways, making it relevant to immune activity.

- MAP2K3 is already recognized as a therapeutic target for immune-mediated diseases, based on substantial evidence of its involvement in inflammatory signaling.

- In comparison, the other genes either lack therapeutic relevance in immune diseases (LGALS3, SMOX, MARCH2) or require more research (PPM1A).

- Considering the intent is to capture an erythroid signature associated with immune activity, MAP2K3 seems well-suited based on its immunologic role.

While none of the genes are erythroid-specific, MAP2K3's importance in immune cell signaling and inflammation make it a reasonable choice to reflect immune-associated erythroid activity. The other genes are less relevant in this context. Please let me know if you need any clarification or have additional questions!

6. Examining expression patterns of top 5 candidates across leukocyte populations

The expression patterns of the top 5 candidate genes were examined across diverse leukocyte populations and hematopoietic precursors using three reference transcriptomic datasets.

6.1 Densely interconnected transcriptional circuits control cell states in human hematopoiesis – (GSE24759)
http://developmentalimmunology.gxbsidra.org/dm3/geneBrowser/show/4000026

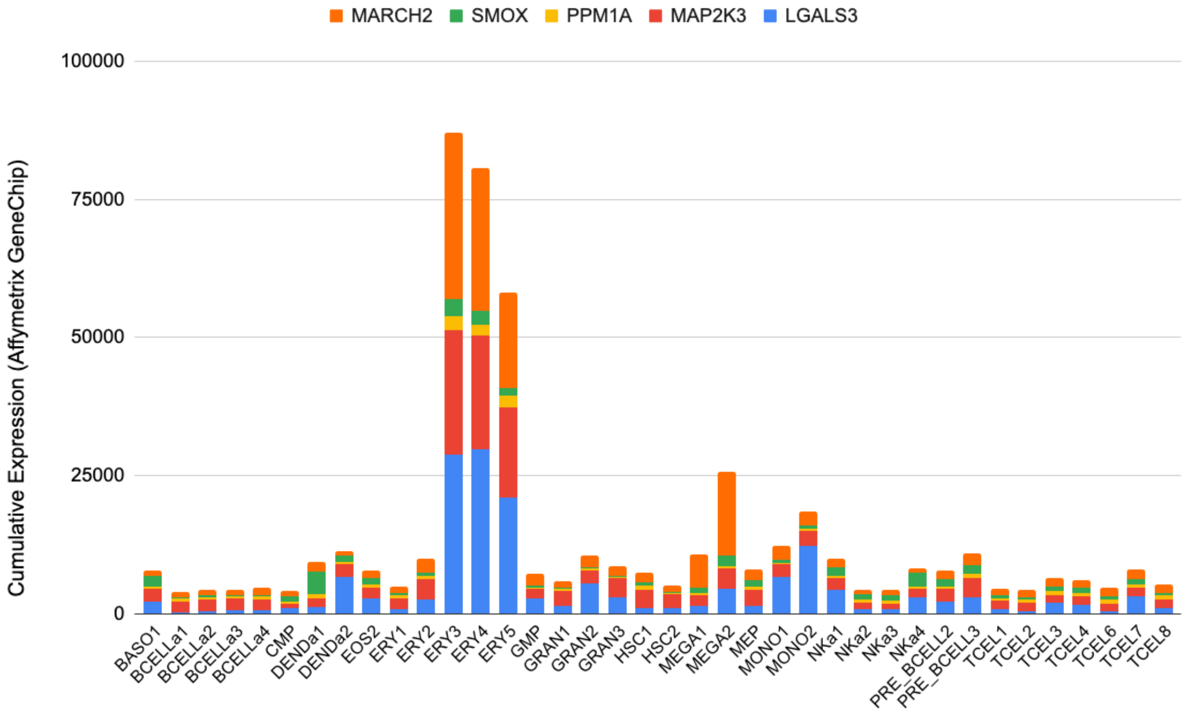

Figure 3: This stacked bar graph shows the expression levels of four of the five candidate genes. ERY3-5 populations are CD71+ GLYA+ erythroid cells. The web link above also provides access to more complete information about this dataset as well as individual gene profiles.

6.2 Next generation sequencing of human immune cell subsets across diseases – (GSE60424)
http://cd2k.gxbsidra.org/dm3/geneBrowser/show/4000098

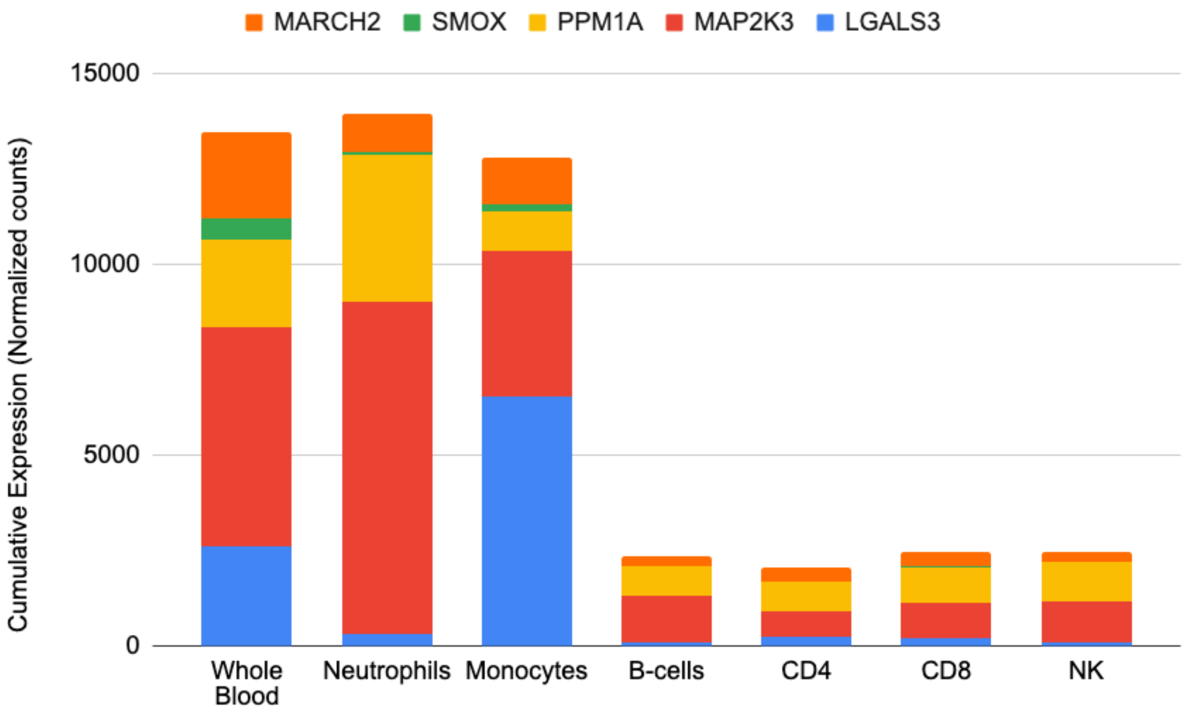

Figure 4: This stacked bar graph shows the expression levels measured by RNAseq of the five candidate genes across six leukocyte populations and whole blood. The abundance is shown for each gene in each population for an average of up to 20 samples. Additional experimental details are accessible via the link provided above and is available as part of this sample series GEO record (GSE60424).

6.3 BloodGen3 reference cohorts (GSE100150)

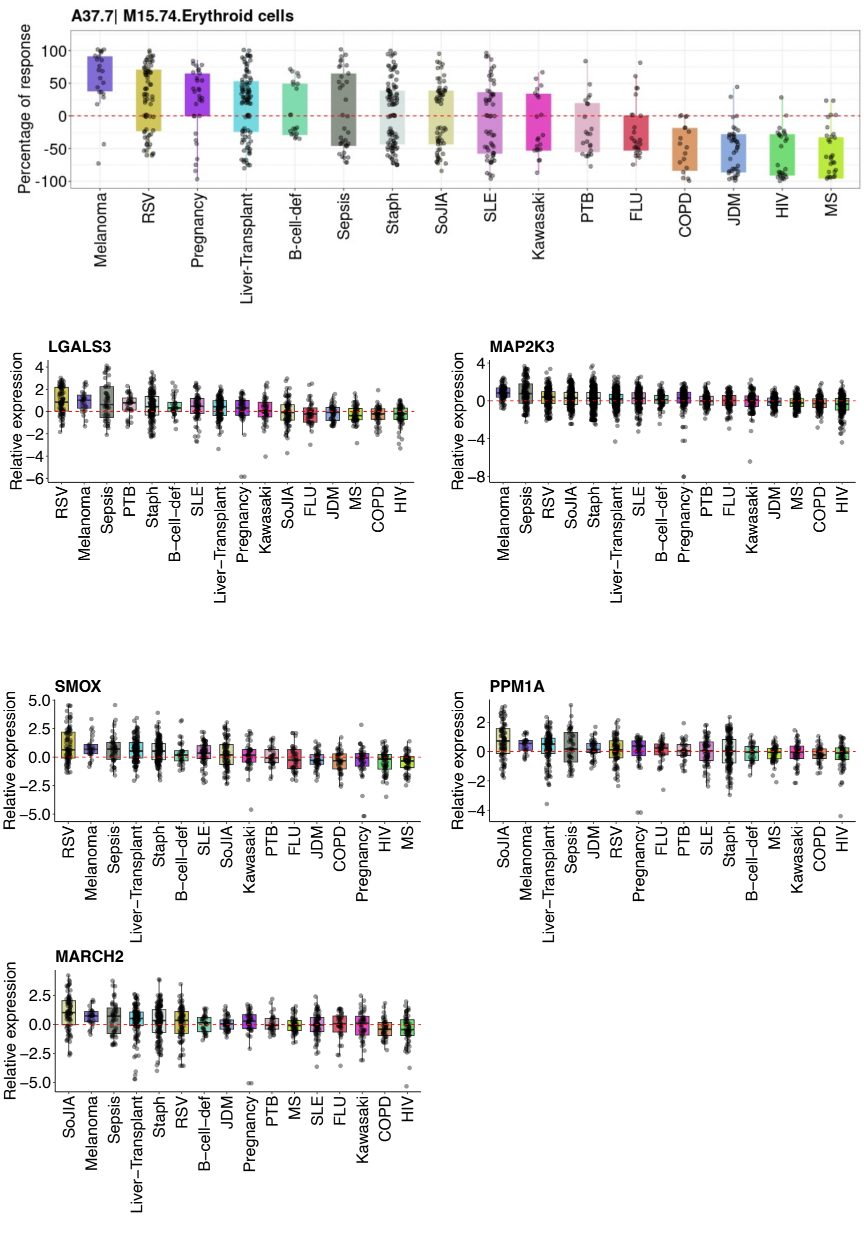

Figure 5: The box plot in panel A shows the changes in abundance of transcripts comprising the M13.30 module, expressed within a cohort as the percentage of genes showing differential expression between each patient and the respective set of control subjects for this cohort. Differential expression is based on fixed cut-off (Log2 Fold Change >1.5, difference>10). These values are expressed as % module activity and can range from 100% (all genes are upregulated) to -100% (all genes are downregulated).

The box plot in panel B show expression levels for the top 5 scoring M15.74 genes in patient cohorts relative to respective control subjects matched for demographic variables.

7. Overall pick(s) for M15.74

We aim to establish a universal immune profiling assay that measures transcript abundance of a panel of genes in whole blood using small- or meso-scale molecular profiling technology. Compelling candidates identified here from the M15.74 selection pool will be subjected to more in-depth evaluation using a pre-established workflow [ref]. MAP2K3 is our top pick for module M15.74, as it scored highly for all criteria.

8. Overall pick for M15.74 based on summarized justifications and reference transcriptional datasets.

GPT-4 and Claude were asked to pick a top candidate. They were told to consider the fact-checked summary justifications that were created earlier. They were also told to factor in the gene expression data from the three reference datasets. By using multi-tier prompting, GPT-4 and Claude could integrate all this information to select the best candidate.

While both models initially selected LGALS3 based on the summaries, GPT4, taking into account the transcriptional profiling data, picked both LGALS3 and SMOX. We concur with this choice given that LGALS3 shows indeed a higher level of restriction to Erythroid cells in the Novershtern dataset. **Therefore,** LGALS3 **is the final pick for module M15.74**

The transcription of the final round of prompting is shown below.

Prompts	CLAUDE	GPT-4
PROMPT 1: Based on the summary below, please recommend the top candidate gene to include in a targeted blood transcriptional profiling panel. The intent is for the	Based on the criteria provided, I would recommend the LGALS3 gene as the top candidate to include in the targeted transcriptional profiling panel. The key reasons are:	evaluation for their potential roles in immune-mediated diseases.<br><br>Considering the provided criteria and the summaries:

selected gene to be representative of an erythroid cell signature associated with response to mRNA vaccines, severity of RSV infection and that found to be expressed at high levels in patients with metastatic melanoma and in liver transplant recipients. Please weigh the following criteria equally in your recommendation: 1) Relevance to erythroid cells and erythropoiesis, 2) Current use as a biomarker, 3) Potential use as a blood transcriptional biomarker, 4) Relevance to leukocyte immune biology, 5) Status as a known drug target, 6) Therapeutic relevance for immune mediated diseases: *[input summarized and fact-checked justifications from Step 5]*	LGALS3 encodes galectin-3, which is expressed in erythroid progenitor cells and involved in erythropoiesis. This establishes its relevance to erythroid cells and erythropoiesis.<br>Galectin-3 is an established biomarker used clinically, especially in heart failure and fibrosis assessment, satisfying the criterion for current use as a biomarker.<br>As a mediator of inflammatory responses, galectin-3 shows promise as a blood transcriptional biomarker related to immune activation and inflammation.<br>Galectin-3 is directly implicated in leukocyte biology and function, including roles in adhesion, migration, and phagocytosis, meeting the criterion for relevance to leukocyte immune biology.<br>Galectin-3 is a recognized drug target with therapeutic relevance in immune-mediated diseases like rheumatoid arthritis, meeting the criteria for known drug target status and therapeutic relevance.<br>In summary, the LGALS3 gene best satisfies the provided criteria when compared to the other options of MAP2K3, PPM1A, SMOX, and MARCH2, which lack definitive evidence for some of the specified criteria. The established role of galectin-3 in erythroid cells, use as a clinical biomarker, links to immune function, and drug target status make LGALS3 the top choice for inclusion in the intended transcriptional profiling panel.	Relevance to erythroid cells and erythropoiesis: None of the genes show a direct association with erythroid cells or erythropoiesis.<br>Current use as a biomarker: Only LGALS3 (Galectin-3) has strong current utilization as a clinical biomarker, notably in heart failure and fibrosis progression.<br>Potential use as a blood transcriptional biomarker: LGALS3 holds the most promise due to its established role in inflammation and fibrosis.<br>Relevance to leukocyte immune biology: Both LGALS3 and MAP2K3 show significant relevance. Galectin-3 has roles in leukocyte function, adhesion, and extravasation. MAP2K3 mediates signaling pathways and potentially regulates cytokine production.<br>Status as a known drug target: MAP2K3 already has established relevance as a drug target, especially in proinflammatory signaling pathways. LGALS3 also holds potential due to its role in immune responses.<br>Therapeutic relevance for immune mediated diseases: Again, LGALS3 and MAP2K3 emerge as top contenders. LGALS3 has been implicated in conditions like rheumatoid arthritis, while MAP2K3's involvement in proinflammatory pathways makes it relevant for various immune-mediated diseases.<br><br>Considering all these factors, LGALS3 (Galectin-3) stands out as the top candidate gene for inclusion in a targeted blood transcriptional profiling panel. Its established role as a clinical biomarker, potential for use as a transcriptional biomarker, relevance to leukocyte biology, and therapeutic potential for immune-mediated diseases

		make it a suitable choice for the study.
PROMPT 2: Now that you have provided an initial recommendation based on the summary, take into account the following RNA-sequencing expression data from key immune cell types and whole blood samples: *[input textualized transcriptional data].* Given this additional RNA-seq expression data, does your recommended gene selection remain the same? Please explain which gene you would recommend at this stage and why, incorporating both the summarized information *provided earlier and RNA-seq data in your rationale."*	After reviewing the additional RNA-sequencing expression data for LGALS3, MAP2K3, PPM1A, SMOX, and MARCH2 across key immune cell types and whole blood, I would still recommend LGALS3 as the top candidate gene for inclusion in the targeted transcriptional profiling panel.<br><br>The rationale is as follows:<br><br>The LGALS3 gene is expressed in whole blood at a reasonable level of 131 counts, indicating it can be readily detected in blood transcriptomic profiling.<br>LGALS3 is most highly expressed in monocytes at 328 counts, which are phagocytic cells relevant to immune activation and inflammation. This fits the intended purpose of assessing immune responses.<br>LGALS3 has low but detectable expression in T cells and NK cells, indicating it captures some representation of lymphocyte biology.<br>MAP2K3, while expressed in immune cells, shows very high expression specifically in neutrophils (436 counts). Since neutrophils may not be the primary cells of interest, this skewed expression could be less ideal.<br>PPM1A is reasonably expressed across immune cells but lacks the additional evidence for erythroid cell relevance and use as a clinical biomarker that LGALS3 has.<br>SMOX has very low expression in immune cells other than monocytes, making it less suitable as a representative biomarker.<br>MARCH2 lacks the definitive links to erythroid lineage and clinical biomarker usage that LGALS3 possesses.	Considering both the summarized information and the RNA-seq expression data, let's evaluate:<br><br>1. LGALS3 (Galectin-3):<br><br>Found in whole blood at a count of 131.<br>Highest expression in monocytes (328), and relatively low expression in neutrophils, CD4 T-cells, CD8 T-cells, NK cells, and B-cells.<br>Previously discussed roles in leukocyte biology, inflammation, and as a biomarker make it relevant.<br><br>2. MAP2K3:<br><br>Found in whole blood at a count of 288.<br>Highest expression in neutrophils (436) and significant expression in monocytes, NK cells, CD8 T-cells, and B-cells.<br>Known for its role in immune signaling and has relevance as a therapeutic target for immune-mediated diseases.<br><br>3. PPM1A:<br><br>Found in whole blood at a count of 114.<br>Highest expression in neutrophils (192), followed by CD8 T-cells, NK cells, and CD4 T-cells.<br>Potential biomarker for certain cancers and might have therapeutic relevance in immune-mediated diseases.<br><br>4. SMOX:<br><br>Found in whole blood at a count of 29.<br>Expression is significantly lower compared to other genes in all immune cells, being negligible or absent in most of them.

	In summary, the LGALS3 gene remains the top choice based on its expression across multiple immune cell types, known erythroid cell and clinical biomarker connections, and relevance to immune biology and disease. The RNA-seq data further supports its potential utility as a transcriptional profiling marker in blood.	Given this low expression, SMOX might not be the top choice as a blood transcriptional biomarker in the context of immune responses.<br><br>5. MARCH2:<br><br>Found in whole blood at a count of 113.<br>Expression in immune cells is relatively moderate, with monocytes, neutrophils, CD8 T-cells, and NK cells showing similar levels.<br>While it has relevance due to its regulation of MHC class II expression, the RNA-seq data doesn't position it as a primary contender.<br><br>Assessment:<br><br>LGALS3: Given the earlier information and the new RNA-seq data, LGALS3 remains a strong candidate, especially considering its high expression in monocytes, which play a pivotal role in immune responses.<br><br>MAP2K3: This gene emerges even stronger when considering the RNA-seq data. It shows significant expression across various immune cells, particularly neutrophils, which are frontline responders in the immune system. Combining this with its known roles in immune signaling and potential therapeutic relevance, MAP2K3 stands out prominently.<br><br>Given the new RNA-seq data, while LGALS3 remains a promising candidate due to its potential as a biomarker and roles in leukocyte biology, MAP2K3 presents a compelling case. It's not only relevant based on its known functions but also has broad expression across critical immune cells, suggesting its potential as a biomarker to

		capture immune responses in the blood.<br><br>Recommendation: Based on both the summarized information and the RNA-seq expression data, I would recommend MAP2K3 as the top candidate gene for inclusion in a targeted blood transcriptional profiling panel.
PROMPT 3: Now that you have provided an initial recommendation based on the summary and the RNAseq data, take into account the following microarray expression data from key immune cell types and hematopoietic precursors: *[input textualized transcriptional data].* Given this additional microarray expression data, does your recommended gene selection remain the same? Please explain which gene you would recommend at this stage and why, incorporating the summarized information and RNA-seq data provided earlier, as well as this new microarray data in your rationale.	After reviewing the extensive microarray expression data across hematopoietic and immune cell types, I still recommend LGALS3 as the top candidate gene for inclusion in the targeted transcriptional profiling panel.<br><br>The key reasons are:<br><br>LGALS3 is expressed across all stages of erythroid differentiation, validating its relevance to erythropoiesis. It peaks in expression during later erythroid maturation.<br>LGALS3 is detected across diverse immune cells like monocytes, dendritic cells, NK cells, and T cells. This supports its utility as an immune biomarker.<br>The microarray data corroborates the RNA-seq evidence of LGALS3 expression in key leukocytes.<br>Compared to MAP2K3, LGALS3 shows a more balanced expression pattern across immune cells, whereas MAP2K3 is very highly expressed in neutrophils.<br>While expressed, PPM1A, SMOX and MARCH2 lack the definitive erythroid lineage and broad clinical biomarker evidence that LGALS3 possesses.<br>The addition of microarray data further solidifies LGALS3 as a gene expressed in both erythroid and immune cells, strengthening the rationale for its selection as a representative biomarker linking	*Given the additional microarray data, let's reevaluate the genes in question:*<br><br>*1. LGALS3 (Galectin-3):*<br><br>*The microarray data reinforces LGALS3's significant expression in myeloid cells, particularly in monocytes, neutrophils, and various progenitors.*<br>*High expression is observed in various stages of erythroid cells and subsets of NK cells.*<br>*Earlier RNA-seq data showed a higher expression of LGALS3 in monocytes.*<br>*Previous information showed its importance in inflammation and leukocyte biology.*<br><br>*2. MAP2K3:*<br><br>*Microarray data reveals broad expression across multiple cell types with notably high expression in neutrophils, erythroid cells, and certain progenitors.*<br>*This complements the RNA-seq data where we saw its prominence across different immune cells.*<br>*As established earlier, MAP2K3 is important in immune signaling.*<br><br>*3. PPM1A:*<br><br>*The expression based on the microarray is relatively moderate across the board, with a higher expression in some subsets of B cells and erythroid cells.*

	erythropoiesis and immune biology.<br>In summary, the combined summarized information, RNA-seq dataset, and extensive microarray profiling validate LGALS3 as the top choice for the intended transcriptional biomarker profiling assay. The expression data align with the known functional connections between LGALS3 and erythroid and immune cell biology.	*RNA-seq data previously highlighted its highest expression in neutrophils and a potential role as a cancer biomarker.*<br><br>*4. SMOX:*<br><br>*The microarray data reflects varied expression in different cell types, with elevated levels in plasmacytoid dendritic cells and subsets of NK cells.*<br>*Earlier RNA-seq data showcased the lower expression of SMOX across most immune cells.*<br><br>*5. MARCH2:*<br><br>*The microarray data indicates significant expression in megakaryocytes and their progenitors, with moderate to high expression across various other cell types.*<br>*The RNA-seq data previously showed moderate expression across immune cells.*<br>*Its known role in regulating MHC class II expression adds to its relevance.*<br><br>*Assessment and Recommendation:*<br><br>*Considering all data sets:*<br><br>*LGALS3: Continues to stand out due to its consistent high expression in myeloid cells (especially monocytes) across both RNA-seq and microarray data. Its known roles in inflammation and leukocyte biology make it further relevant.*<br><br>*MAP2K3: Still emerges as a strong contender. It shows broad and significant expression across multiple immune cell types in both data sets. Given its established role in immune signaling and potential therapeutic relevance, it remains at the forefront.*

		PPM1A, SMOX, & MARCH2: While these genes have relevance in certain contexts and show differential expression in specific cell types, they don't consistently dominate across the board as MAP2K3 and LGALS3 do.<br><br>*Given the comprehensive data from the summary, RNA-seq, and now the microarray, both LGALS3 and MAP2K3 remain top contenders. However, considering the significance and breadth of expression across immune cells combined with known biological relevance, MAP2K3 remains the top recommendation for inclusion in a targeted blood transcriptional profiling panel.*<br><br>*In this context, it's also essential to understand the overall goal of the study. For a broader representation of immune responses, a combination of genes might be more suitable. But, if the goal is to prioritize a single gene for in-depth study or targeted profiling, MAP2K3 stands out with the provided data.*
PROMPT 4: Now that you have provided an initial recommendation based on the summary and the immune cells RNAseq and microarray data, take into account the following averaged log2 fold changes in RNA abundance in patient cohorts compared to controls. *[input textualized transcriptional data].* Given this additional data, does your recommended gene selection remain the same? Please explain which gene you would recommend at this stage and why, incorporating the summarized information, the leukocytes RNA-seq and microarray data provided earlier,	After reviewing the log2 fold change data across various disease states and conditions, I still recommend LGALS3 as the top candidate gene for the intended transcriptional profiling panel.<br><br>The key reasons are:<br><br>LGALS3 shows statistically significant differential expression in multiple immune-mediated conditions like HIV, sepsis, tuberculosis, and RSV infection. This validates its utility as an inflammatory biomarker.<br>The fold changes align with LGALS3's known involvement in immune cell adhesion, migration, and phagocytosis.	Given the provided data, let's evaluate the gene profiles of LGALS3, PPM1A, MAP2K3, SMOX, and MARCH2 across different conditions to make a well-informed recommendation:<br><br>1. LGALS3:<br><br>Highly upregulated in cases such as metastatic melanoma, sepsis, and acute respiratory syncytial virus infection.<br>Downregulated in HIV, COPD, and multiple sclerosis.<br><br>2. PPM1A:<br><br>Generally shows minor changes across most conditions, except in HIV (notable

as well as these new patient cohort profiles in your rationale.	Compared to the other genes, LGALS3 has a greater number of disease associations with large effect sizes.<br>MAP2K3, PPM1A, SMOX and MARCH2 also show some differential expression, but the magnitude and significance is lower across conditions.<br>No single gene is differentially expressed in every condition, but LGALS3 has the most consistent and largest changes.<br>The patient cohort data provides clinical validation of LGALS3's relevance as a biomarker linking immune status to disease states.<br>In summary, the addition of the patient cohort profiles further cements LGALS3 as a top candidate based on its detectable and statistically significant differential expression in multiple immune-mediated conditions. This reinforces the rationale from the previous data showing LGALS3's balanced expression across immune cell types and known links to erythroid lineage. LGALS3 remains the top choice as a representative transcriptional biomarker.	downregulation) and systemic onset juvenile idiopathic arthritis (notable upregulation).<br><br>3. MAP2K3:<br><br>Highly upregulated in conditions like metastatic melanoma and sepsis.<br>Highly downregulated in HIV.<br><br>4. SMOX:<br><br>Highly upregulated in conditions such as metastatic melanoma, sepsis, acute respiratory syncytial virus infection.<br>Highly downregulated in HIV and multiple sclerosis.<br><br>5. MARCH2:<br><br>Notably upregulated in systemic onset juvenile idiopathic arthritis and metastatic melanoma.<br>Highly downregulated in HIV.<br><br>Given the new data and considering the previous information:<br><br>LGALS3 and SMOX both show significant differential expression in several disease conditions, indicating they are potentially involved in various pathological processes and may be potential biomarkers for these diseases.<br>MAP2K3 and MARCH2 also exhibit changes, but their patterns are not as consistent as LGALS3 and SMOX across diseases.<br>PPM1A's changes are generally mild except in a few cases.<br><br>Recommendation:<br>At this stage, I would recommend focusing on LGALS3 and SMOX given their consistent differential expression across several disease conditions. Both genes

		are upregulated in conditions like sepsis, metastatic melanoma, and acute respiratory syncytial virus infection. This suggests a potential central role in immune response and disease progression. The previous data also showed LGALS3 was differentially expressed in various immune cells, further supporting its role in immune-mediated responses.<br><br>However, the selection would depend on the research goal:<br><br>If the aim is to find broad-spectrum markers that are involved in multiple conditions, LGALS3 and SMOX would be the top candidates.<br>If the aim is more disease-specific, then it would be essential to consider the disease of interest and see which gene is most differentially expressed in that context.<br><br>Incorporating the summarized information, leukocytes RNA-seq, and microarray data, LGALS3 and SMOX seem to be the most promising candidates, but the ultimate decision would require a more in-depth understanding of the specific research objectives.
PROMPT 5: Could you please summarize the key conclusions you have drawn from the conversation so far?	Here is a summary of the key conclusions I have drawn regarding selection of a candidate gene for a targeted transcriptional profiling panel:<br><br>The initial summary highlighted LGALS3, MAP2K3, PPM1A, SMOX, and MARCH2 as potential candidates based on varied criteria. Of these, LGALS3 had the most direct evidence linking it to erythroid cells and use as a clinical biomarker.<br>Examination of RNA-seq data showed LGALS3 to be reasonably expressed across diverse immune cell types	*Here's a summarized breakdown of our conversation and the key conclusions:*<br><br>*Initial Information: You provided a summary of differentially expressed genes across various immune cell types, namely LGALS3, PPM1A, MAP2K3, SMOX, and MARCH2.*<br><br>*RNA-seq and Microarray Data Analysis:*<br>*LGALS3: Differentially expressed in neutrophils, CD14+ monocytes, CD4 T-cells, CD8 T-cells, and B-cells.*

	compared to the other genes, further supporting its utility.<br>Microarray profiling expanded the data showing expression of all genes across hematopoietic and immune cells. LGALS3 was detected across erythroid maturation stages, aligning with its erythroid relevance.<br>Patient cohort log2 fold change data provided clinical validation, with LGALS3 showing statistically significant differential expression in multiple immune-mediated diseases with large effect sizes.<br>Across all stages of data integration, LGALS3 emerged as the top choice based on its erythroid lineage connections, balanced expression across immune cells, use as a clinical biomarker, and detectable differential expression in immune-related diseases.<br>In summary, the layered evidence consistently points to LGALS3 as the top candidate gene for inclusion in the intended targeted transcriptional profiling panel aiming to capture an erythroid signature linked to immune status.	*PPM1A: Differentially expressed in neutrophils, CD14+ monocytes, and CD4 T-cells.*<br>*MAP2K3: Differentially expressed in neutrophils, CD14+ monocytes, and CD4 T-cells.*<br>*SMOX: Differentially expressed in neutrophils and CD14+ monocytes.*<br>*MARCH2: Differentially expressed in neutrophils.*<br><br>*Additional Patient Cohort Data Analysis:*<br>*LGALS3: Showed significant differential expression across many disease conditions, notably upregulated in diseases such as metastatic melanoma, sepsis, and acute respiratory syncytial virus infection.*<br>*PPM1A: Exhibited minor changes across most conditions, with significant downregulation in HIV.*<br>*MAP2K3: Notably upregulated in conditions like metastatic melanoma and sepsis, and significantly downregulated in HIV.*<br>*SMOX: Displayed significant differential expression, especially upregulated in sepsis, metastatic melanoma, and acute respiratory syncytial virus infection.*<br>*MARCH2: Showed pronounced upregulation in systemic onset juvenile idiopathic arthritis and downregulation in HIV.*<br><br>*Recommendation Based on Combined Data:*<br>*LGALS3 and SMOX emerged as top candidates for further study given their differential expression across several disease conditions, suggesting a potential central role in immune response and disease progression. Their expression patterns also aligned with the immune cell data.*<br>*The selection of genes for further study would be influenced by the specific research objectives,*

		whether broad-spectrum markers are sought or a more disease-specific focus is desired.<br><br>*In essence, based on the integrated analysis of immune cell types, RNA-seq, microarray data, and disease-specific patient cohorts, LGALS3 and SMOX stand out as potentially key players in immune-mediated responses across various diseases.*

References cited:

1. Liu FT, Rabinovich GA. Galectins as modulators of tumour progression. Nat Rev Cancer. 2005 Jan;5(1):29–41.

2. Rabinovich GA, Toscano MA. Turning “sweet” on immunity: galectin-glycan interactions in immune tolerance and inflammation. Nat Rev Immunol. 2009 May;9(5):338–52.

3. Kyriakis JM, Avruch J. Mammalian mitogen-activated protein kinase signal transduction pathways activated by stress and inflammation. Physiol Rev. 2001 Apr;81(2):807–69.

4. Lu X, Nguyen TA, Moon SH, Darlington Y, Sommer M, Donehower LA. The type 2C phosphatase Wip1: an oncogenic regulator of tumor suppressor and DNA damage response pathways. Cancer Metastasis Rev. 2008 Jun;27(2):123–35.

5. Casero RA, Pegg AE. Spermidine/spermine N1-acetyltransferase--the turning point in polyamine metabolism. FASEB J Off Publ Fed Am Soc Exp Biol. 1993 May;7(8):653–61.

6. Goto E, Ishido S, Sato Y, Ohgimoto S, Ohgimoto K, Nagano-Fujii M, et al. c-MIR, a human E3 ubiquitin ligase, is a functional homolog of herpesvirus proteins MIR1 and MIR2 and has similar activity. J Biol Chem. 2003 Apr 25;278(17):14657–68.

7. de Boer RA, Voors AA, Muntendam P, van Gilst WH, van Veldhuisen DJ. Galectin-3: a novel mediator of heart failure development and progression. Eur J Heart Fail. 2009 Sep;11(9):811–7.

8. Wanninger J, Weigert J, Wiest R, Bauer S, Karrasch T, Farkas S, et al. Systemic and hepatic vein galectin-3 are increased in patients with alcoholic liver cirrhosis and negatively correlate with liver function. Cytokine. 2011 Sep;55(3):435–40.

9. Lu X, An H, Jin R, Zou M, Guo Y, Su PF, et al. PPM1A is a RelA phosphatase with tumor suppressor-like activity. Oncogene. 2014 May 29;33(22):2918–27.

10. Casero RA, Murray Stewart T, Pegg AE. Polyamine metabolism and cancer: treatments, challenges and opportunities. Nat Rev Cancer. 2018 Nov;18(11):681–95.

11. Henderson NC, Sethi T. The regulation of inflammation by galectin-3. Immunol Rev. 2009 Jul;230(1):160–71.

12. Bartee E, Mansouri M, Hovey Nerenberg BT, Gouveia K, Früh K. Downregulation of major histocompatibility complex class I by human ubiquitin ligases related to viral immune evasion proteins. J Virol. 2004 Feb;78(3):1109–20.

13. Rabinovich GA, Baum LG, Tinari N, Paganelli R, Natoli C, Liu FT, et al. Galectins and their ligands: amplifiers, silencers or tuners of the inflammatory response? Trends Immunol. 2002 Jun;23(6):313–20.

14. Sano H, Hsu DK, Apgar JR, Yu L, Sharma BB, Kuwabara I, et al. Critical role of galectin-3 in phagocytosis by macrophages. J Clin Invest. 2003 Aug;112(3):389–97.

15. Inoue T, Boyle DL, Corr M, Hammaker D, Davis RJ, Flavell RA, et al. Mitogen-activated protein kinase kinase 3 is a pivotal pathway regulating p38 activation in inflammatory arthritis. Proc Natl Acad Sci U S A. 2006 Apr 4;103(14):5484–9.

16. Toya M, Terasawa M, Nagata K, Iida Y, Sugimoto A. A kinase-independent role for Aurora A in the assembly of mitotic spindle microtubules in Caenorhabditis elegans embryos. Nat Cell Biol. 2011 Jun;13(6):708–14.

17. Liu H, Wilson KR, Firth AM, Macri C, Schriek P, Blum AB, et al. Ubiquitin-like protein 3 (UBL3) is required for MARCH ubiquitination of major histocompatibility complex class II and CD86. Nat Commun. 2022 Apr 11;13(1):1934.

18. Capone E, Iacobelli S, Sala G. Role of galectin 3 binding protein in cancer progression: a potential novel therapeutic target. J Transl Med. 2021 Sep 26;19(1):405.

19. Cuenda A, Rousseau S. p38 MAP-kinases pathway regulation, function and role in human diseases. Biochim Biophys Acta. 2007 Aug;1773(8):1358–75.

20. Goldstein DM, Gabriel T. Pathway to the clinic: inhibition of P38 MAP kinase. A review of ten chemotypes selected for development. Curr Top Med Chem. 2005;5(10):1017–29.

21. Yamaguchi H, Durell SR, Chatterjee DK, Anderson CW, Appella E. The Wip1 phosphatase PPM1D dephosphorylates SQ/TQ motifs in checkpoint substrates phosphorylated by PI3K-like kinases. Biochemistry. 2007 Nov 6;46(44):12594–603.

22. MacKinnon AC, Farnworth SL, Hodkinson PS, Henderson NC, Atkinson KM, Leffler H, et al. Regulation of alternative macrophage activation by galectin-3. J Immunol Baltim Md 1950. 2008 Feb 15;180(4):2650–8.

23. Kumar S, Boehm J, Lee JC. p38 MAP kinases: key signalling molecules as therapeutic targets for inflammatory diseases. Nat Rev Drug Discov. 2003 Sep;2(9):717–26.

24. Lin X, Duan X, Liang YY, Su Y, Wrighton KH, Long J, et al. PPM1A functions as a Smad phosphatase to terminate TGFbeta signaling. Cell. 2006 Jun 2;125(5):915–28.

25. Murray-Stewart TR, Woster PM, Casero RA. Targeting polyamine metabolism for cancer therapy and prevention. Biochem J. 2016 Oct 1;473(19):2937–53.

26. Matsuki Y, Ohmura-Hoshino M, Goto E, Aoki M, Mito-Yoshida M, Uematsu M, et al. Novel regulation of MHC class II function in B cells. EMBO J. 2007 Feb 7;26(3):846–54.
