# Supplementary material for: Harnessing large language models (LLMs) for candidate gene prioritization and selection
Source: J Transl Med. 2023 Oct 16;21:728. doi: 10.1186/s12967-023-04576-8 (PMC10580627; doi:10.1186/s12967-023-04576-8)
Supplement: Supplementary file 12 — Additional file 12: Is the prioritization report for module M15.100 authored by Mohammed Toufiq. The file is shared in the PDF format. [file 12967_2023_4576_MOESM12_ESM.pdf]

M15.100 Prioritization report

Prepared by: Mohammed Toufiq (The Jackson Laboratory for Genomic Medicine, Farmington, CT, USA)

1. Composition

The module M15.100 belongs to Aggregate A37 from the BloodGen3 fixed module repertoire [1], [2]. This module aggregate is associated with circulating erythroid cells. It was found to be associated with RSV disease severity [3]. We showed in the same study that the abundance of A27 transcripts is also elevated in the blood of patients with stage IV melanoma [3].

It comprises 17 genes: ARHGEF12, C14ORF45, CISD2, CMBL, FLCN, GCAT, GYPE, HBBP1, LOC253012, PCSK1N, PLVAP, RHD, SLC6A9, SPTB, TBCEL, TMEM56, and YPEL4.

2. Functional convergence

Functional Themes	Associated Genes	Comments
Cell growth and differentiation	ARHGEF12, FLCN	This gene encodes a member of the Rho guanine nucleotide exchange factor family and is involved in the regulation of cell growth and differentiation.This gene is involved in the development of various types of benign skin tumors, lung cysts, and kidney tumors, and mutations in this gene are associated with Birt-Hogg-Dube syndrome.
Components of blood and erythrocyte structure	RHD, SPTB, GYPE	The protein encoded by this gene is the Rh polypeptide, which is part of the Rh blood group system.Encodes beta spectrin, a member of the spectrin gene family which is a crucial component of the cytoskeletal network that underlies the red cell plasma membrane. Encodes a member of the glycophorin family, playing a role in maintaining the stability of the red blood cell membrane.
Metabolic processes	CMBL, GCAT	Encodes an enzyme that has a role in metabolizing the cysteine conjugate of certain halogenated alkenes and alkanes. Involved in the breakdown and utilization of ketobutyrate and encoding an enzyme involved in the catabolic pathway of methionine.

3. Scoring and prioritization

Genes were scored on six criteria using GPT-4 and Claude. The scores were averaged, and candidate genes ranked according to their cumulative scores (**Figure 1**, Methods: Step 3 and Step 4).

The two LLMs were requested to score each gene on the following six statements:
a. The gene is associated with erythroid cells or erythropoiesis.

b. The gene is currently being used as a biomarker in clinical settings.
c. The gene has potential value as a blood transcriptional biomarker.
d. The gene is relevant to circulating leukocytes immune biology.
e. The gene is a known drug target.
f. The gene is therapeutically relevant for immune-mediated diseases.

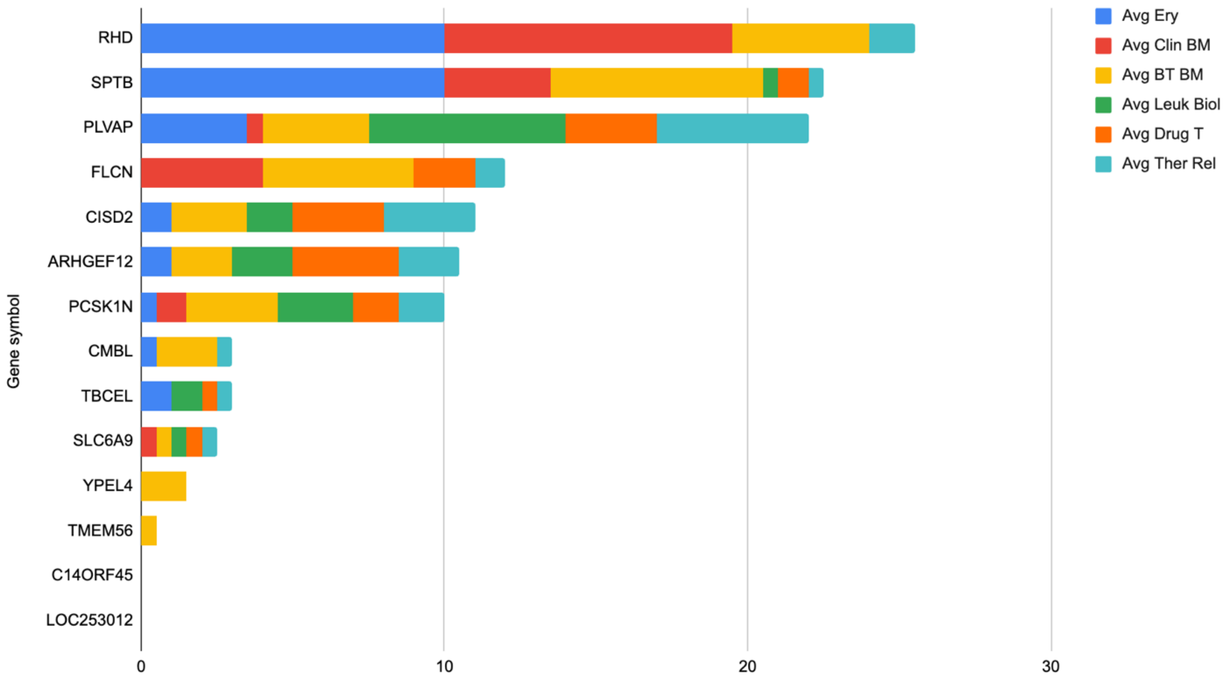

Figure 1: The stacked bar graph shows cumulative scores across six criteria for the 17 M15.100 genes.

The top five genes selected for further evaluation were: RHD, SPTB, PLVAP, FLCN, and CISD2 **(Figure 2**).

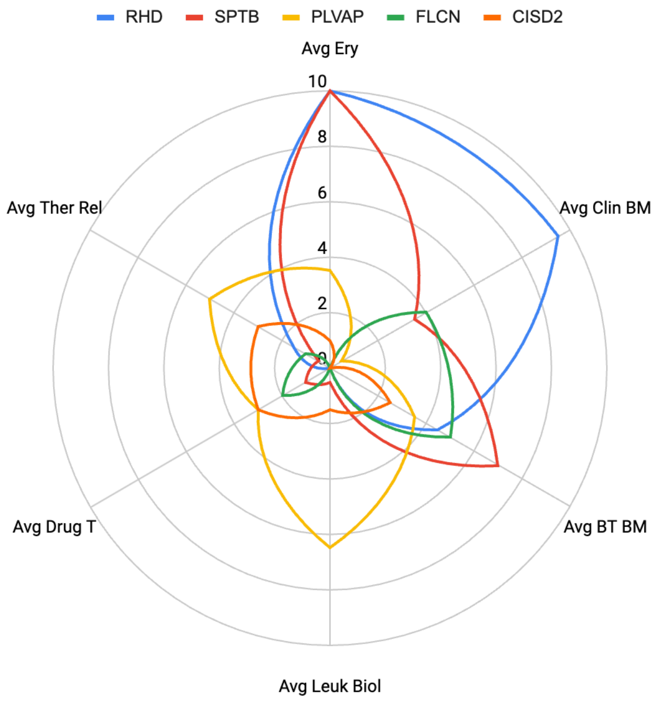

Figure 2: The radar plot represents individual scores for the 5 top scoring genes: RHD, SPTB, PLVAP, FLCN, and CISD2.

4. Knowledge-driven evaluation of top five candidates

Justifications given by the GPT-4 and Claude for the scores provided across the 6 criteria were compiled and summarized by GPT-4 (Methods: Step 5). For each statement pertinent references were retrieved using GPT-4 or Claude, with the relevance of each reference checked and attributed manually by researcher authoring this report.

Function: "The RHD gene is responsible for encoding the RhD protein, which constitutes the D antigen of the Rh blood group system. This antigen is a protein expressed on the surface of erythrocyte membranes. An individual's blood type is determined as Rh positive or Rh negative based on the presence or absence of this D antigen. Notably, the RhD antigen is the most immunogenic within the Rh system. The formation of anti-RhD antibodies can lead to conditions such as hemolytic disease in newborns and hemolytic transfusion reactions [4], [5]. The SPTB gene is responsible for encoding beta spectrin, an integral element of the cytoskeletal framework in erythrocytes. Beta spectrin, in conjunction with alpha spectrin, creates the spectrin heterodimer. These heterodimers organize and associate to construct a meshwork essential for preserving the erythrocytes' distinctive biconcave morphology and ensuring membrane durability during traversal through constricted capillaries. Furthermore, SPTB encodes the beta subunit of erythrocyte spectrin, a principal structural constituent of the red blood cell membrane skeleton. Working synergistically with protein 4.1, spectrin facilitates the

crosslinking of actin filaments, thereby imparting membrane adaptability and robustness [6]–[11]. PLVAP is an endothelial-specific protein essential for regulating vascular permeability and mediating leukocyte extravasation. It plays a pivotal role in the formation of diaphragms of fenestrae within endothelial cells and is instrumental in establishing the stomatal and fenestral diaphragms found in continuous and fenestrated capillaries. Furthermore, besides its presence on endothelial cells, PLVAP is also expressed on lymphocytes. Dysfunction of PLVAP can lead to vascular leakage and subsequent inflammation [12]–[15]. The FLCN gene encodes the protein folliculin, a vital regulator of cellular growth, proliferation, metabolism, and autophagy. Folliculin interacts with FNIP1/2 and AMPK to function as a tumor suppressor. Mutations in the FLCN gene are linked to Birt-Hogg-Dubé syndrome, manifesting as skin tumors, lung cysts, and kidney tumors, including its association with renal cancer [16]–[25]. The CISD2 gene encodes a protein that resides in the outer mitochondrial membrane, integral to the energy-producing compartments of cells. This protein is pivotal in controlling intracellular calcium levels and safeguards against cell death, ensuring the normal functionality of mitochondria. Moreover, CISD2 is essential for maintaining mitochondrial integrity and longevity. A deficiency in CISD2 compromises mitochondrial performance and is associated with Wolfram syndrome 2 [26]–[28], [29, p. 2], [30]".

a. <u>Relevance to erythroid cells and erythropoiesis:</u> "The RHD gene is closely associated with erythroid cells, given its role in coding for the D antigen present on erythrocytes. There is robust evidence indicating that RHD is specifically expressed in the erythroid lineage, serving as a primary membrane protein in erythrocytes [4], [5], [31], [32]. The SPTB gene exhibits a pronounced association with erythroid cells and the process of erythropoiesis. Deficiencies in spectrin, the product of the SPTB gene, are frequently implicated in hereditary spherocytosis, a disorder affecting red blood cells. Furthermore, there is compelling evidence that SPTB is specifically expressed within the erythroid lineage, functioning as a critical element of the erythrocyte membrane cytoskeleton [6]–[11], [33]–[36]. PLVAP is implicated in the formation of fenestrae in the endothelium of sinusoids, particularly within the spleen and bone marrow where erythropoiesis transpires. However, current evidence does not directly associate PLVAP with erythroid cells or the process of erythropoiesis itself. Notably, its expression is localized to the endothelium [12], [14], [37]. To date, there is no established direct correlation between the FLCN gene and erythroid cells or the process of erythropoiesis. Research has not found evidence pointing towards FLCN's specific association with erythroid cells or erythropoiesis. The gene exhibits a broad expression pattern across different cell types [38], [39]. Current research offers only limited evidence that directly links the CISD2 gene with erythroid cells or the erythropoiesis process. Investigations have not identified a specific association of CISD2 with erythroid cells or erythropoiesis. It's notable that the gene is broadly expressed across a range of cell types [29, p. 2], [40]–[43]".

b. <u>Is used as a clinical biomarker:</u> "The RHD gene, while not employed as a traditional biomarker, holds significant importance in clinical environments for blood typing and mitigating the risk of RhD incompatibility during pregnancy. RHD genotyping is consistently utilized to

forecast the likelihood of RhD incompatibility reactions [4], [44]–[46]. Mutations in the SPTB gene are linked with hereditary spherocytosis and other red blood cell-related disorders, suggesting its potential utility as a clinical biomarker for these conditions. However, there is no documented evidence supporting the direct clinical use of SPTB levels as a biomarker. The diagnosis of related conditions, such as elliptocytosis, primarily relies on genetic testing [36], [47]–[50]. In the current clinical landscape, PLVAP is not extensively utilized as a biomarker. There is a lack of documented evidence supporting the direct clinical application of PLVAP levels for biomarker purposes [51]–[54]. The FLCN gene, while associated with Birt-Hogg-Dubé syndrome, is not employed as a conventional biomarker in clinical settings. Instead, the diagnosis of Birt-Hogg-Dubé syndrome primarily relies on genetic testing [20], [55]–[58]. Currently, CISD2 is not extensively adopted as a biomarker in clinical contexts. There exists no substantiated evidence promoting the use of CISD2 levels for clinical biomarker applications. The primary methodology for diagnosing Wolfram syndrome 2 remains genetic testing [27], [29, p. 2], [41], [59]–[63]".

c. Potential relevance as a blood transcriptional biomarker: "The RHD gene, while not acting as a conventional blood transcriptional biomarker, is pivotal in delineating blood type, a fundamental component in transfusion medicine. Being an erythroid membrane protein, the levels of RHD can signify erythropoietic activity and have the potential to evaluate the rate of red cell production [4], [64], [65]. Due to its intrinsic connection with erythrocyte architecture and functionality, SPTB holds potential as an insightful blood transcriptional biomarker for disorders impacting red blood cells. As an integral erythroid structural protein, variations in SPTB levels may serve as an indicator of erythropoietic activity and the rate of red cell generation [47], [66]–[68]. Given its involvement in vascular biology, PLVAP may hold prospective merit as a blood transcriptional biomarker. Preliminary data suggest a correlation between PLVAP and vascular permeability. However, its validation and establishment as a reliable biomarker necessitate further investigation [12], [13], [53], [69]–[71]. Given its association with Birt-Hogg-Dubé syndrome, FLCN is posited as a potential blood transcriptional biomarker, especially in individuals possessing a familial history of the syndrome. The wider applicability of FLCN as a biomarker for other conditions remains to be validated. Functioning as a regulator of metabolism and growth, variations in FLCN levels may have implications for these processes, though comprehensive validation is still required [21], [24], [72], [73]. Preliminary data hint at the potential of CISD2 as a blood transcriptional biomarker, though comprehensive validation remains necessary. Being a mitochondrial protein, fluctuations in CISD2 levels might have implications for mitochondrial functionality. Further research is essential to confirm its utility in this capacity [26], [74]".

d. Relevance to leukocytes immune biology: "The RHD gene, predominantly expressed in erythroid cells, has no established relevance to the immune biology of circulating leukocytes. Its specificity lies primarily with erythroid cells, and its association with leukocytes remains unverified [75], [76]. The SPTB gene, predominantly involved in erythrocyte structure and function, holds limited relevance to the immune biology of circulating leukocytes. Its primary specificity is directed towards erythrocytes, and there is no established evidence indicating its association with leukocyte processes [77], [78]. Given its role in overseeing leukocyte

extravasation, PLVAP holds significance in the biology of circulating leukocytes. Expressed predominantly on lymphocytes, PLVAP facilitates their transmigration, underscoring its relevance in leukocyte biology [69]. To date, there is no empirical evidence indicating a substantial role for FLCN in the immune biology of circulating leukocytes. Specifically, no studies or data have elucidated a distinct function for FLCN within circulating leukocyte processes [72], [79]. Given its role in mitochondrial functionality, CISD2 might be considered relevant to the immune biology of circulating leukocytes, given the critical role mitochondria play in immune cell operations. However, to date, no specific evidence has been identified that directly links CISD2 to the processes of circulating leukocytes [No references]".

e. Is a known drug target: "In the current biomedical landscape, RHD is not recognized as a drug target. No documented evidence supports the proposition of RHD serving as a potential target for pharmacological intervention [80], [81]. While SPTB is not conventionally identified as a drug target in the current biomedical paradigm, investigations into spectrin's pivotal role in maintaining erythrocyte structural integrity hint at potential therapeutic avenues in the future. As of now, there is no documented evidence that designates SPTB as a target for pharmacological interventions [No reference]. Given its proposed role in diseases characterized by enhanced vascular permeability, PLVAP has been postulated as a potential therapeutic target. However, as of now, there is no empirical evidence that substantiates PLVAP as an established target for pharmacological interventions [No reference]. Given FLCN's involvement in cellular growth and proliferation pathways, it might be hypothesized as a potential drug target. However, in the current biomedical research landscape, FLCN has not been extensively investigated for this potential role, and there exists no concrete evidence supporting its categorization as an established pharmacological target [82]. While there exists evidence indicating that CISD2 may have potential utility as a drug target, especially within the realms of neurodegenerative diseases and cancer, due to its involvement in apoptosis and autophagy processes, it is currently not recognized as an established pharmacological target in the biomedical field [83]".

f. Potential therapeutic relevance for immune-mediated diseases: "The RHD gene, primarily known as an erythroid blood group antigen, does not possess direct therapeutic relevance for immune-mediated diseases. However, its significance is underscored in the context of immune responses arising from RhD incompatibility during pregnancy [80], [81], [84]–[89]. SPTB, chiefly involved in determining erythrocyte structure, is not currently recognized as having therapeutic relevance in the context of immune-mediated diseases. There is no documented evidence that associates SPTB directly with immune-mediated disease processes, underscoring its primary function in maintaining erythrocyte structural integrity [35], [48], [90], [91]. PLVAP's involvement in regulating leukocyte migration suggests potential therapeutic relevance for immune-mediated diseases. While there is evidence associating PLVAP with inflammation in the endothelium, its specific role in autoimmunity remains less explored and necessitates further investigation [52]. The role of FLCN in immune-mediated diseases remains ambiguous at present. Although it is primarily characterized as a tumor suppressor, there is no concrete evidence, as of yet, that directly associates FLCN deficiency

with immune-mediated conditions. Furthermore, no known immunomodulatory therapies currently target FLCN [20], [56], [73], [92]. Given its involvement in apoptosis and autophagy processes, CISD2 might be hypothesized as having relevance in the context of immune-mediated diseases. However, current evidence does not specifically associate CISD2 deficiency with such diseases. Its primary associations have been found with neurodegenerative conditions [59], [63], [93]–[98]".

5. Examining expression patterns of top 5 candidates across leukocyte populations

The expression patterns of the top 5 candidate genes were examined across diverse leukocyte populations and hematopoietic precursors using two reference transcriptomic datasets.

5.1 Densely interconnected transcriptional circuits control cell states in human hematopoiesis – (GSE24759)
http://developmentalimmunology.gxbsidra.org/dm3/geneBrowser/show/4000026

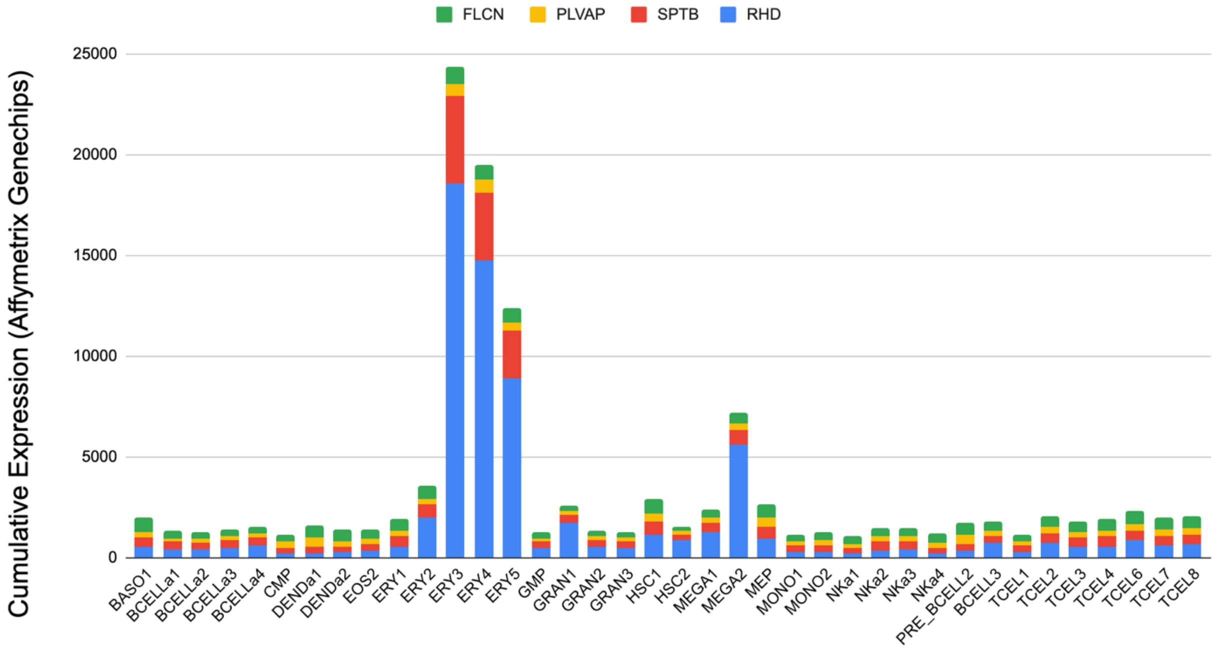

Figure 3: This stacked bar graph shows the expression levels of five candidate genes. ERY3-5 populations are CD71+ GLYA+ erythroid cells. The original article provides full experimental details: [99]. The web link above also provides access to more complete information about this dataset as well as individual gene profiles.

5.2 Next generation sequencing of human immune cell subsets across diseases – (GSE60424)
http://cd2k.gxbsidra.org/dm3/geneBrowser/show/4000098

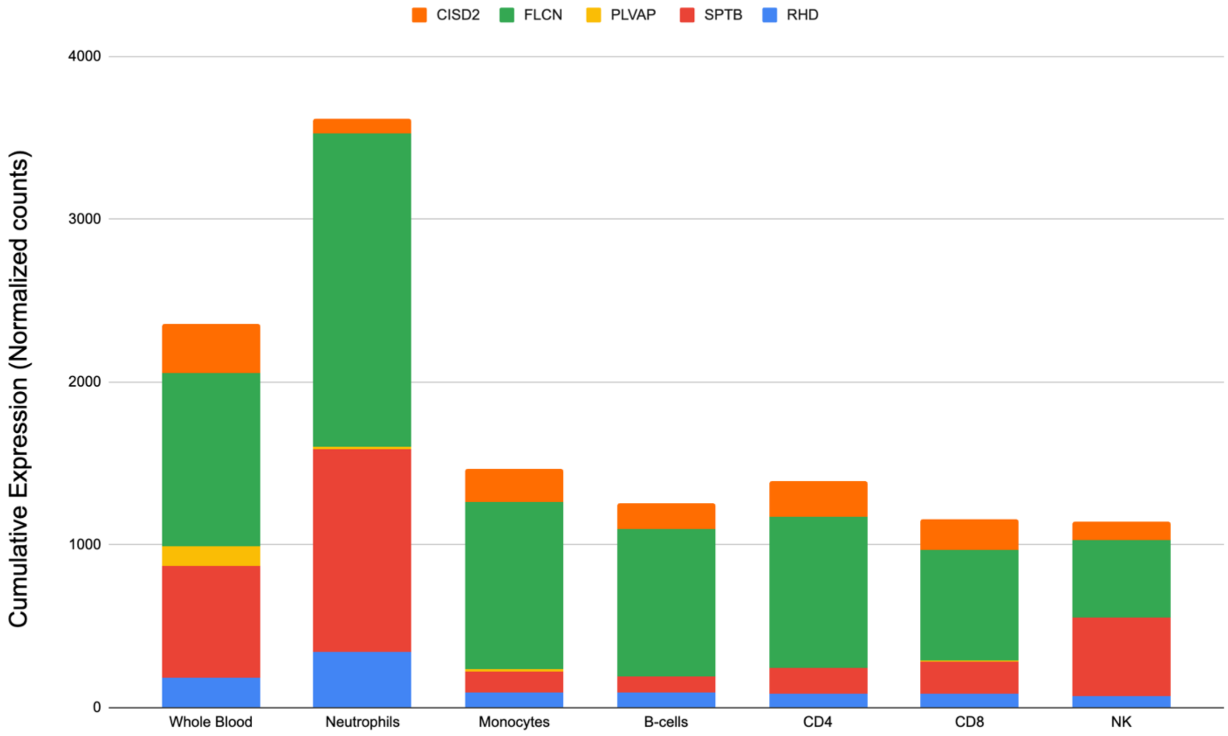

Figure 4: This stacked bar graph shows the expression levels measured by RNAseq of the five candidate genes across six leukocyte populations and whole blood. The abundance is shown for each gene in each population for an average of up to 20 samples. Additional experimental details are accessible via the link provided above and is available as part of this sample series GEO record (GSE60424) [100].

5.3 BloodGen3 reference cohorts (GSE100150)

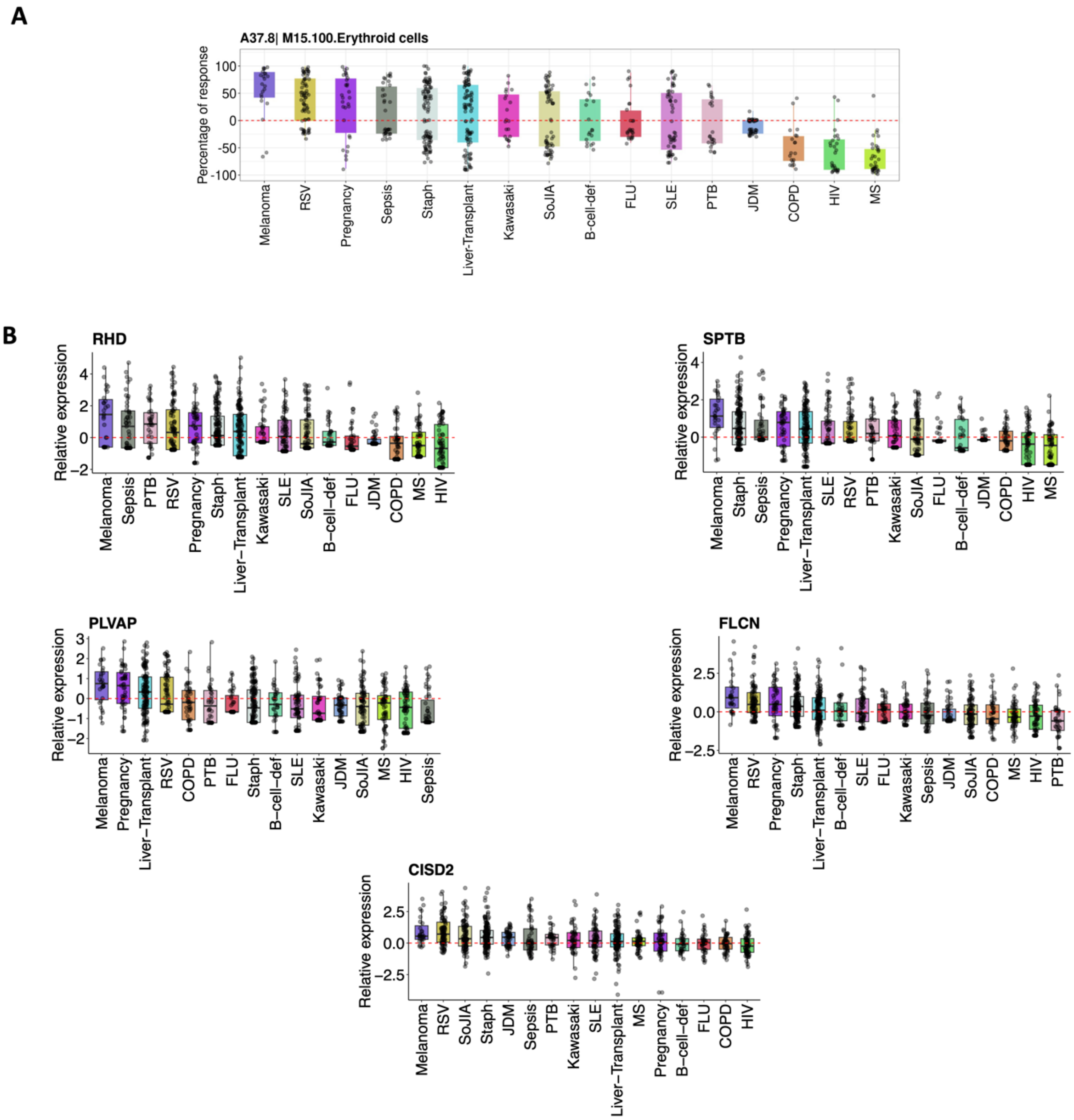

Figure 5: The box plot in panel A shows the changes in abundance of transcripts comprising the M15.100 module, expressed within a cohort as the percentage of genes showing differential expression between each patient and the respective set of control subjects for this cohort. Differential expression is based on fixed cut-off (Log2 Fold Change >1.5, difference>10). These values are expressed as % module activity and can range from 100% (all genes are upregulated) to -100% (all genes are downregulated). The box plot in panel B show expression levels for the top 5 scoring M15.100 genes in patient cohorts relative to respective control subjects matched for demographic variables.

6. Overall pick(s) for M15.100

We aim to establish a universal immune profiling assay that measures transcript abundance of a panel of genes in whole blood using small- or meso-scale molecular profiling technology. Compelling candidates identified here from the M15.100 selection pool will be subjected to more in-depth evaluation using a pre-established workflow [101], [102].

Claude and GPT-4 were prompted to select a top candidate on the basis of the information summarized above in section 4, taking into account all six scoring criteria equally (Methods: Step 7).

<u>Claude chose SPTB</u>:

Here is a summary of the key conclusions so far in our conversation about selecting a candidate gene to include in a targeted transcriptional profiling panel:

Initial Assessment

- Based on the initial summary, SPTB was recommended as the top gene choice due to its erythroid-specificity, known functional role in erythrocyte biology, and biomarker potential. In comparison, other genes like RHD, PLVAP, FLCN and CISD2 had less direct erythroid evidence.

Expression Data Evaluation

- The RNA-seq and microarray expression data across immune cells and whole blood samples confirmed that SPTB had the most erythroid-restricted expression pattern compared to the other genes. Its expression was robustly detected.
- RHD was also erythroid relevant but showed a broader expression profile extending to immune cells. Its expression in whole blood was lower and more variable than SPTB based on RNA-seq.

Patient Cohort Profiles

- Across multiple immune-mediated diseases, SPTB showed mild-moderate upregulation in expression compared to controls. This supported its biomarker prospects.
- In comparison, RHD was more variably up and down-regulated across patient cohorts. The other genes showed minimal changes.

Key Conclusions

- Overall, the combined data at multiple levels solidifies SPTB as an optimal erythroid representative for inclusion in the panel based on its erythroid specificity, detectable expression, and upregulation in immune conditions.

- SPTB consistently stood out as aligning closely with the criteria outlined compared to other genes.

GPT-4 chose RHD:

Certainly! Here's a summary of our conversation:

1. Research Objective:
• You're working on understanding the role of certain genes, especially in relation to immune cell populations and their differential expression in various health conditions.
• These genes are: RHD, SPTB, PLVAP, FLCN, and CISD2.

2. Initial Data Analysis:
• We began by examining the differential expression of these genes in various immune cell populations using RNAseq and microarray data. Key observations included:
• RHD showed substantial variation in T cells, particularly CD8 T cells and memory resting CD4 T cells.
• SPTB was majorly expressed in neutrophils.
• PLVAP had substantial expression in dendritic cells.
• FLCN showed significant expression in naïve CD4 T cells.
• CISD2 showed variation mainly in memory activated CD4 T cells and monocytes.

3. Disease-related Differential Expression:
• You then provided a comprehensive list of log2 fold changes for these genes across numerous health conditions as compared to controls. Some notable patterns are:
• RHD: Substantial increased expression in conditions like metastatic melanoma, Kawasaki disease, active pulmonary tuberculosis, sepsis, and during pregnancy.
• SPTB: Elevated expression in metastatic melanoma, sepsis, and staphylococcus aureus infection.
• PLVAP: Decreased expression in several conditions like B-cell deficiency, HIV, multiple sclerosis, sepsis, and others. However, an increase was noted in metastatic melanoma and during pregnancy.
• FLCN: Elevated expression in metastatic melanoma, during pregnancy, and in respiratory syncytial virus infection.
• CISD2: This gene showed variations across multiple conditions, but the provided data was cut off.

4. Potential Implications:
• RHD: Its variability across multiple immune cell types and significant upregulation in several conditions might suggest a broader role in immune responses.
• SPTB: Its predominant expression in neutrophils and changes in conditions like sepsis and infections could link it to frontline immune responses.
• PLVAP: Given its high expression in dendritic cells and downregulation in many conditions, it might be involved in antigen presentation or initial immune recognition.

• FLCN: Elevated expression in conditions like melanoma might hint at a role in tumor-associated immune mechanisms.
• CISD2: Its importance is yet to be discussed in detail.

To move forward, I recommended:
• Exploring functional studies or literature reviews for these genes to understand their role in immune mechanisms.
• Conducting pathway analysis to see how these genes might interact in signaling cascades or cellular processes.
• Considering network analysis to explore potential interacting proteins or genes that might provide more context to the observations.

Given the discussion so far:
• RHD: Showed substantial variation across T cell types and significant upregulation in conditions like metastatic melanoma, Kawasaki disease, active pulmonary tuberculosis, sepsis, and during pregnancy. Given its broad range of differential expression across conditions and cell types, it seems to be a strong candidate.
• SPTB: Its major expression in neutrophils and upregulation in conditions like sepsis and infections makes it relevant, especially for frontline immune responses.
• PLVAP: Although it showed a high expression in dendritic cells, it exhibited decreased expression in many conditions. While this downregulation might be important to understand specific immune mechanisms, its role as a marker in a gene panel is unclear unless the panel is specifically designed to monitor suppression of certain immune pathways.
• FLCN: Elevated expression in metastatic melanoma and during pregnancy could be of interest, especially if the panel aims to study tumor-associated immune mechanisms or reproductive immunology.
• CISD2: The data provided for this gene was cut off, so a comprehensive assessment was not made. However, given its differential expression in memory activated CD4 T cells and monocytes, further investigation is warranted.

Conclusion: If you have to prioritize, RHD seems to be the most promising candidate given its differential expression across a variety of conditions and immune cell types. SPTB and FLCN are also intriguing based on their specific patterns of expression in certain conditions and cell types.

Considering this, in conjunction with the rationale provided by both LLMs and our understanding of RHD and SPTB biological and clinical relevance, we identify SPTB as our primary M15.100 selection for subsequent characterization.

Prompts	CLAUDE	GPT-4
PROMPT 1: Based on the summary below, please recommend the top candidate gene to include in a targeted blood transcriptional profiling panel. The intent is for the selected gene to be representative of an erythroid cell signature associated with response to mRNA vaccines, severity of RSV infection and that found to be expressed at high levels in patients with metastatic melanoma and in liver transplant recipients. Please weigh the following criteria equally in your recommendation: 1) Relevance to erythroid cells and erythropoiesis, 2) Current use as a biomarker, 3) Potential use as a blood transcriptional biomarker, 4) Relevance to leukocyte immune biology, 5) Status as a known drug target, 6) Therapeutic relevance for immune mediated diseases: *[input summarized and fact-checked justifications from Step 5]*	Based on weighing the criteria equally, I would recommend the SPTB gene as the top candidate to include in the targeted blood transcriptional profiling panel.<br><br>The key reasons are:<br><br>• SPTB has high relevance to erythroid cells and erythropoiesis, encoding beta spectrin which is vital for erythrocyte cytoskeletal structure.<br>• While not currently used as a biomarker, SPTB has clear potential as a blood transcriptional biomarker for erythropoiesis rates and red blood cell disorders.<br>• SPTB is erythroid-specific with no known direct relevance to leukocyte biology.<br>• Though not an established drug target, SPTB hints at therapeutic potential for erythrocyte disorders.<br>• SPTB has no identified therapeutic significance for	Based on the criteria provided and the information on each gene presented in the summary, let's analyze the top candidate gene for inclusion in the targeted blood transcriptional profiling panel:<br><br>1. Relevance to erythroid cells and erythropoiesis:<br>• RHD: High relevance. Expressed on erythrocytes and delineates Rh blood type.<br>• SPTB: High relevance. Vital for erythrocyte's cytoskeleton and shape.<br>• PLVAP: Low relevance. Not directly linked to erythroid cells but involved in erythropoiesis sites.<br>• FLCN: No known relevance.<br>• CISD2: No known relevance.<br><br>2. Current use as a biomarker:<br>• RHD: Used in blood typing.<br>• SPTB: Potential, but not broadly employed.<br>• PLVAP: Not employed.<br>• FLCN: Diagnosis based on genetic testing.<br>• CISD2: Genetic testing for specific conditions.<br><br>3. Potential use as a blood transcriptional biomarker:<br>• RHD: Potential to indicate erythropoietic activity.<br>• SPTB: Potential for red blood cell disorders.

	immune-mediated diseases.<br><br>In comparison:<br><br>• RHD is highly erythroid-relevant but lacks biomarker potential beyond blood typing.<br>• PLVAP has leukocyte relevance but less direct erythroid evidence.<br>• FLCN and CISD2 have ambiguous erythroid connections.<br><br>Overall, SPTB appears to best fit the specified criteria for an erythroid-representative gene in the panel context described. Its known erythroid functions and biomarker prospects make it a sound choice.	• PLVAP: Emerging potential.<br>• FLCN: Uncertain relevance.<br>• CISD2: Initial promise but needs research.<br><br>4. Relevance to leukocyte immune biology:<br>• RHD: No known relevance.<br>• SPTB: No known relevance.<br>• PLVAP: High relevance, especially its role in leukocyte transmigration.<br>• FLCN: No known relevance.<br>• CISD2: Potential, given mitochondria's role in immune cells.<br><br>5. Status as a known drug target:<br>• RHD: No current evidence.<br>• SPTB: No current evidence.<br>• PLVAP: Potential but unconfirmed.<br>• FLCN: Potential but unconfirmed.<br>• CISD2: Potential but not definitive.<br><br>6. Therapeutic relevance for immune-mediated diseases:<br>• RHD: Relevance in managing immune responses in RhD incompatibility.<br>• SPTB: No identified therapeutic relevance.<br>• PLVAP: Potential therapeutic significance.<br>• FLCN: No known relevance.<br>• CISD2: More related to neurodegenerative disorders.<br><br>Recommendation:<br>Given the equal weight of the six criteria:

		• RHD stands out due to its high relevance to erythroid cells (1), its use in blood typing (2), potential as a blood transcriptional biomarker (3), and its therapeutic significance in RhD incompatibility (6).<br><br>• SPTB is also significant because of its direct involvement in erythroid cell structure (1) and its potential as a blood transcriptional biomarker for erythropoiesis (3).<br><br>• PLVAP shines in its relevance to leukocyte immune biology (4) and potential therapeutic significance for immune-mediated diseases (6).<br><br>Considering all aspects, RHD seems to be the top candidate, closely followed by SPTB, due to its high relevance to erythroid cells, current clinical use, and potential as a blood transcriptional biomarker.
PROMP 2: Now that you have provided an initial recommendation based on the summary, take into account the following RNA-sequencing expression data from key immune cell types and whole blood samples: *[input textualized transcriptional data].* Given this additional RNA-seq expression data, does your	After reviewing the additional RNA-sequencing expression data for the RHD, SPTB, PLVAP, FLCN, and CISD2 genes across multiple human whole blood samples, I would still recommend the SPTB gene as the top choice to include in the targeted transcriptional profiling panel.	Given the provided RNA-seq data, let's evaluate each gene's average expression in human whole blood:<br><br>1. RHD:<br>• Sum: 55.649 (from provided values)<br>• Average: 55.649/7 = 7.949857142857142<br>2. SPTB:

recommended gene selection remain the same? Please explain which gene you would recommend at this stage and why, incorporating both the summarized information *provided earlier and RNA-seq data in your rationale."*	The key reasons are:<br><br>- The RNA-seq data shows SPTB is robustly expressed in whole blood at moderate levels (average of ~15 counts), supporting its relevance as an erythroid signature gene.<br><br>- RHD, while erythroid-relevant, shows lower and more variable expression (average of ~5 counts) in whole blood based on the RNA-seq data.<br><br>- The other genes like PLVAP, FLCN, and CISD2 exhibit either low (PLVAP) or high but variable (FLCN, CISD2) expression in whole blood, with less direct erythroid evidence.<br><br>- SPTB remains the most erythroid-specific gene with moderate RNA expression levels detected in whole blood samples.<br><br>- Earlier summarized information highlighted SPTB's erythroid functionality and biomarker potential.<br>In summary, the additional RNA-seq expression data is consistent with and reinforces SPTB as a sound choice for an erythroid representative gene in the desired targeted blood transcriptional profiling panel, based on its	• Sum: 170.8662857142857 (from provided values)<br>• Average: 170.8662857142857/7 = 24.4094693877551<br>3. PLVAP:<br>• Sum: 7.813142857142857 (from provided values)<br>• Average: 7.813142857142857/7 = 1.116163265306122<br>4. FLCN:<br>• Sum: 359.9718571428571 (from provided values)<br>• Average: 359.9718571428571/7 = 51.42455030612245<br>5. CISD2:<br>• Sum: 66.8385 (from provided values)<br>• Average: 66.8385/7 = 9.548357142857142<br>From the expression data:<br>• FLCN has the highest average RNA expression in whole blood.<br>• SPTB has the second highest average RNA expression in whole blood.<br>• RHD and CISD2 have moderate RNA expression in whole blood.<br>• PLVAP has the lowest RNA expression in whole blood.<br>Considering the summarized information provided earlier:<br>1. RHD: High relevance to erythroid cells, not a conventional biomarker but pivotal in blood typing, lacks potential as a blood transcriptional biomarker, has no known relevance to leukocyte immune biology,

	specificity, expression levels, and relevance to the criteria outlined. The RNA-seq findings further support the earlier rationale for selecting SPTB.	not identified as a drug target, lacks direct therapeutic significance for immune-mediated diseases.<br>2. SPTB: High relevance to erythroid cells, linked to hereditary spherocytosis but not a traditional biomarker, offers potential as a blood transcriptional biomarker, no significant relevance to leukocyte biology, not identified as a drug target, has no identified therapeutic relevance for immune-mediated diseases.<br>3. PLVAP: Moderate relevance to erythroid cells, not broadly employed as a clinical biomarker, emerging potential as a blood biomarker, plays a pivotal role in the biology of circulating leukocytes, potential therapeutic target.<br>4. FLCN: No direct relevance to erythroid cells, potential biomarker in the context of familial syndromes, no clear relevance to circulating leukocyte biology, potential as a drug target unestablished, primary characterization as a tumor suppressor.<br>5. CISD2: No direct relevance to erythroid cells, not acknowledged as a biomarker, potential as a blood biomarker under-investigated, association with mitochondrial functionality suggests potential relevance

		to leukocyte biology but no direct evidence.<br><br>Recommendation:<br>Given the criteria and the expression data:<br>1. FLCN has the highest RNA expression, but its relevance to erythroid cells and erythropoiesis is questionable, as is its current use as a biomarker or potential as a blood transcriptional biomarker.<br>2. SPTB shows both high relevance to erythroid cells and a fairly high RNA expression in whole blood. It offers potential as a blood transcriptional biomarker and is linked to red blood cell disorders.<br>Considering all the factors, while FLCN has the highest RNA expression in the dataset, SPTB seems to be the most appropriate candidate based on its high relevance to erythroid cells, potential as a blood transcriptional biomarker, and moderate expression in human whole blood. This recommendation aligns with the initial suggestion, even when factoring in the RNA-seq data.
PROMPT 3: Now that you have provided an initial recommendation based on the summary and the RNAseq data, take into account the following microarray expression data from key	After reviewing the additional microarray expression data, I would still recommend SPTB as the top candidate gene to include in the targeted transcriptional profiling panel.	*[N/A: input exceed GPT-4 limits. Attempts to break down inputting the textualized data were unsuccessful as GPT-4 lost the thread of the conversation.*

immune cell types and hematopoietic precursors: *[input textualized transcriptional data].* Given this additional microarray expression data, does your recommended gene selection remain the same? Please explain which gene you would recommend at this stage and why, incorporating the summarized information and RNA-seq data provided earlier, as well as this new microarray data in your rationale.	The key reasons are:<br><br>- The microarray data confirms SPTB is highly expressed in erythroid lineage cells like erythroblast subsets compared to other cell types. This aligns with its known erythroid functionality.<br><br>- SPTB expression is more erythroid-specific compared to the other genes based on the microarray expression across cell types.<br><br>- RHD, while very erythroid relevant, shows a broader expression pattern in immune cells based on the microarray data.<br><br>- PLVAP, FLCN, CISD2 continue to show ambiguous or non-erythroid expression patterns.<br><br>- The RNA-seq data previously showed moderate SPTB expression in whole blood samples, supporting its detection and relevance.<br>- Earlier summarized information highlighted SPTB's erythroid role and biomarker potential.<br><br>In totality, the combined expression data at the RNA and protein level solidifies SPTB as an ideal erythroid representative with known	*Thus, we chose to skip to the next prompt]*

	biology and detectable expression. The new microarray findings reinforce the rationale for choosing SPTB to capture an erythroid signature.	
PROMPT 4: Now that you have provided an initial recommendation based on the summary and the immune cells RNAseq and microarray data, take into account the following averaged log2 fold changes in RNA abundance in patient cohorts compared to controls. *[input textualized transcriptional data].* Given this additional data, does your recommended gene selection remain the same? Please explain which gene you would recommend at this stage and why, incorporating the summarized information, the leukocytes RNA-seq and microarray data provided earlier, as well as these new patient cohort profiles in your rationale.	After reviewing the additional data on expression changes in various patient cohorts, I would still recommend SPTB as the top gene to include in the targeted transcriptional profiling panel.<br><br>The key reasons are:<br><br>- Across multiple immune-mediated conditions like SLE, juvenile arthritis, and sepsis, SPTB shows consistent mild-moderate increases in expression compared to controls. This aligns with its potential as a biomarker.<br><br>- In comparison, RHD, though erythroid relevant, shows more variable up and down expression changes across cohorts. Its biomarker prospects appear less consistent.<br><br>- The other genes like PLVAP, FLCN, and CISD2 show ambiguous or minimal expression changes in patient cohorts.<br><br>- The earlier RNA-seq and microarray data demonstrated SPTB's	The given data provides a comprehensive picture of how the expression of specific genes (RHD, SPTB, PLVAP, FLCN, and CISD2) changes across various conditions/diseases when compared to controls. Let's analyze these findings to gain some insights:<br>RHD<br>• Generally overexpressed in conditions such as Kawasaki disease, metastatic melanoma, pregnancy, active pulmonary tuberculosis, acute respiratory syncytial virus infection, sepsis, systemic lupus erythematosus, systemic onset juvenile idiopathic arthritis, staphylococcus aureus infection, and liver transplant recipients under maintenance therapy.<br>• Notably, it's significantly downregulated in HIV infection, which is intriguing since it's a decrease of more than 1-fold.<br>• Overall, RHD is predominantly upregulated across several conditions, especially in those involving immune responses.<br>SPTB<br>• Most conditions indicate an upregulation except for

	erythroid specificity and expression detectability.<br><br>- Summarized information highlighted SPTB's erythroid biology and biomarker potential.<br><br>In totality, SPTB continues to stand out as an erythroid representative with biomarker prospects based on its detectable expression and mild-moderate upregulation across multiple immune conditions. The patient cohort findings add further support for SPTB as a meaningful erythroid signature gene with relevance to immune activity.	chronic obstructive pulmonary disease, HIV infection, juvenile dermatomyositis, and multiple sclerosis.<br>• Metastatic melanoma and staphylococcus aureus infection show the most significant upregulation of this gene.<br>• This gene might have a role in the immune response, given its modulation in various infections and immune-mediated conditions.<br>PLVAP<br>• This gene appears to be mostly downregulated in several conditions, especially HIV infection, B-cell deficiency, chronic obstructive pulmonary disease, acute influenza infection, juvenile dermatomyositis, Kawasaki disease, multiple sclerosis, active pulmonary tuberculosis, sepsis, systemic lupus erythematosus, systemic onset juvenile idiopathic arthritis, and staphylococcus aureus infection.<br>• The notable exceptions are metastatic melanoma, pregnant subjects, and liver transplant recipients, where it is upregulated.<br>• The downregulation in most inflammatory and infectious diseases suggests that PLVAP might have a protective role or be

		suppressed as a disease mechanism.<br>FLCN<br>• This gene is predominantly upregulated in metastatic melanoma, pregnant subjects, and acute respiratory syncytial virus infection.<br>• It is notably downregulated in HIV infection and active pulmonary tuberculosis.<br>• Given the varied response across conditions, it suggests that FLCN might be involved in specific pathways that are triggered in some diseases and suppressed in others.<br>CISD2<br>• The data for CISD2 appears to be truncated, but from what is visible, there isn't a significant change in B-cell deficiency and chronic obstructive pulmonary disease.<br><br>From these observations:<br>1. HIV infection appears to have the most consistent effect on the downregulation of these genes, especially with RHD and PLVAP, which are significantly suppressed.<br>2. Metastatic melanoma induces a strong upregulation in all genes, especially RHD and SPTB, suggesting that these genes might have roles in tumor progression or the body's response to the tumor.<br>3. Diseases with autoimmune components like systemic

		lupus erythematosus and systemic onset juvenile idiopathic arthritis seem to modulate these genes differently. While RHD and SPTB are upregulated, PLVAP is downregulated.<br>4. It's interesting to note the change in pregnant subjects. Pregnancy is known to cause immune modulation, which might be reflected in the upregulation of RHD, SPTB, PLVAP, and FLCN.<br><br>Considering these data alongside the immune cell-specific expression data given previously, it's evident that these genes play a role in immune modulation and response. Depending on the condition or disease, these genes can be either upregulated or downregulated, which further underscores their potential importance in the immune response. Continued research is needed to delve deeper into the specific pathways and mechanisms through which these genes operate, especially in the context of these diseases.
PROMPT 5: Could you please summarize the key conclusions you have drawn from the conversation so far?	Here is a summary of the key conclusions so far in our conversation about selecting a candidate gene to include in a targeted transcriptional profiling panel:<br><br>Initial Assessment	Certainly! Here's a summary of our conversation:<br>1. Research Objective:<br>• You're working on understanding the role of certain genes, especially in relation to immune cell populations and their

	- Based on the initial summary, SPTB was recommended as the top gene choice due to its erythroid-specificity, known functional role in erythrocyte biology, and biomarker potential. In comparison, other genes like RHD, PLVAP, FLCN and CISD2 had less direct erythroid evidence.<br><br>Expression Data Evaluation<br>- The RNA-seq and microarray expression data across immune cells and whole blood samples confirmed that SPTB had the most erythroid-restricted expression pattern compared to the other genes. Its expression was robustly detected.<br><br>- RHD was also erythroid relevant but showed a broader expression profile extending to immune cells. Its expression in whole blood was lower and more variable than SPTB based on RNA-seq.<br><br>Patient Cohort Profiles<br>- Across multiple immune-mediated diseases, SPTB showed mild-moderate upregulation in expression compared to controls. This supported its biomarker prospects.<br><br>- In comparison, RHD was more variably up and down-regulated across patient	differential expression in various health conditions.<br>• These genes are: RHD, SPTB, PLVAP, FLCN, and CISD2.<br><br>2. Initial Data Analysis:<br>• We began by examining the differential expression of these genes in various immune cell populations using RNAseq and microarray data. Key observations included:<br>• RHD showed substantial variation in T cells, particularly CD8 T cells and memory resting CD4 T cells.<br>• SPTB was majorly expressed in neutrophils.<br>• PLVAP had substantial expression in dendritic cells.<br>• FLCN showed significant expression in naïve CD4 T cells.<br>• CISD2 showed variation mainly in memory activated CD4 T cells and monocytes.<br><br>3. Disease-related Differential Expression:<br>• You then provided a comprehensive list of log2 fold changes for these genes across numerous health conditions as compared to controls. Some notable patterns are:<br>• RHD: Substantial increased expression in conditions like metastatic melanoma, Kawasaki disease, active pulmonary tuberculosis, sepsis, and during pregnancy.

	cohorts. The other genes showed minimal changes.<br><br>Key Conclusions<br>- Overall, the combined data at multiple levels solidifies SPTB as an optimal erythroid representative for inclusion in the panel based on its erythroid specificity, detectable expression, and upregulation in immune conditions.<br><br>- SPTB consistently stood out as aligning closely with the criteria outlined compared to other genes.	• SPTB: Elevated expression in metastatic melanoma, sepsis, and staphylococcus aureus infection.<br>• PLVAP: Decreased expression in several conditions like B-cell deficiency, HIV, multiple sclerosis, sepsis, and others. However, an increase was noted in metastatic melanoma and during pregnancy.<br>• FLCN: Elevated expression in metastatic melanoma, during pregnancy, and in respiratory syncytial virus infection.<br>• CISD2: This gene showed variations across multiple conditions, but the provided data was cut off.<br><br>4. Potential Implications:<br>• RHD: Its variability across multiple immune cell types and significant upregulation in several conditions might suggest a broader role in immune responses.<br>• SPTB: Its predominant expression in neutrophils and changes in conditions like sepsis and infections could link it to frontline immune responses.<br>• PLVAP: Given its high expression in dendritic cells and downregulation in many conditions, it might be involved in antigen presentation or initial immune recognition.

		• FLCN: Elevated expression in conditions like melanoma might hint at a role in tumor-associated immune mechanisms.<br>• CISD2: Its importance is yet to be discussed in detail.<br>To move forward, I recommended:<br>• Exploring functional studies or literature reviews for these genes to understand their role in immune mechanisms.<br>• Conducting pathway analysis to see how these genes might interact in signaling cascades or cellular processes.<br>• Considering network analysis to explore potential interacting proteins or genes that might provide more context to the observations.<br><br>Given the discussion so far:<br><br>• RHD: Showed substantial variation across T cell types and significant upregulation in conditions like metastatic melanoma, Kawasaki disease, active pulmonary tuberculosis, sepsis, and during pregnancy. Given its broad range of differential expression across conditions and cell types, it seems to be a strong candidate.<br><br>• SPTB: Its major expression in neutrophils and upregulation in conditions like sepsis and infections makes it relevant, especially

		for frontline immune responses.<br><br>• PLVAP: Although it showed a high expression in dendritic cells, it exhibited decreased expression in many conditions. While this downregulation might be important to understand specific immune mechanisms, its role as a marker in a gene panel is unclear unless the panel is specifically designed to monitor suppression of certain immune pathways.<br><br>• FLCN: Elevated expression in metastatic melanoma and during pregnancy could be of interest, especially if the panel aims to study tumor-associated immune mechanisms or reproductive immunology.<br><br>• CISD2: The data provided for this gene was cut off, so a comprehensive assessment was not made. However, given its differential expression in memory activated CD4 T cells and monocytes, further investigation is warranted.<br>Conclusion: If you have to prioritize, RHD seems to be the most promising candidate given its differential expression across a variety of conditions and immune cell types. SPTB and FLCN are also intriguing based on their specific patterns of

		expression in certain conditions and cell types. However, the final choice should be based on the specific objectives of the gene panel and the diseases or conditions you aim to target.

7. References

[1] M. C. Altman *et al.*, “Development of a fixed module repertoire for the analysis and interpretation of blood transcriptome data,” *Nat. Commun.*, vol. 12, no. 1, p. 4385, Jul. 2021, doi: 10.1038/s41467-021-24584-w.

[2] D. Rinchai *et al.*, “BloodGen3Module: blood transcriptional module repertoire analysis and visualization using R,” *Bioinforma. Oxf. Engl.*, vol. 37, no. 16, pp. 2382–2389, Aug. 2021, doi: 10.1093/bioinformatics/btab121.

[3] D. Rinchai *et al.*, “Definition of erythroid cell-positive blood transcriptome phenotypes associated with severe respiratory syncytial virus infection,” *Clin. Transl. Med.*, vol. 10, no. 8, p. e244, Dec. 2020, doi: 10.1002/ctm2.244.

[4] N. D. Avent and M. E. Reid, “The Rh blood group system: a review,” *Blood*, vol. 95, no. 2, pp. 375–387, Jan. 2000.

[5] K. J. Moise, “Management of rhesus alloimmunization in pregnancy,” *Obstet. Gynecol.*, vol. 100, no. 3, pp. 600–611, Sep. 2002, doi: 10.1016/s0029-7844(02)02180-4.

[6] V. Bennett and A. J. Baines, “Spectrin and ankyrin-based pathways: metazoan inventions for integrating cells into tissues,” *Physiol. Rev.*, vol. 81, no. 3, pp. 1353–1392, Jul. 2001, doi: 10.1152/physrev.2001.81.3.1353.

[7] V. Bennett and D. M. Gilligan, “The spectrin-based membrane skeleton and micron-scale organization of the plasma membrane,” *Annu. Rev. Cell Biol.*, vol. 9, pp. 27–66, 1993, doi: 10.1146/annurev.cb.09.110193.000331.

[8] S. E. Lux, “Anatomy of the red cell membrane skeleton: unanswered questions,” *Blood*, vol. 127, no. 2, pp. 187–199, Jan. 2016, doi: 10.1182/blood-2014-12-512772.

[9] N. Mohandas and E. Evans, “Mechanical properties of the red cell membrane in relation to molecular structure and genetic defects,” *Annu. Rev. Biophys. Biomol. Struct.*, vol. 23, pp. 787–818, 1994, doi: 10.1146/annurev.bb.23.060194.004035.

[10] M. Salomao *et al.*, “Protein 4.1R-dependent multiprotein complex: new insights into the structural organization of the red blood cell membrane,” *Proc. Natl. Acad. Sci. U. S. A.*, vol. 105, no. 23, pp. 8026–8031, Jun. 2008, doi: 10.1073/pnas.0803225105.

[11] J. Palek, “Hereditary elliptocytosis, spherocytosis and related disorders: consequences of a deficiency or a mutation of membrane skeletal proteins,” *Blood Rev.*, vol. 1, no. 3, pp. 147–168, Sep. 1987, doi: 10.1016/0268-960x(87)90031-2.

[12] R. V. Stan *et al.*, “The diaphragms of fenestrated endothelia: gatekeepers of vascular permeability and blood composition,” *Dev. Cell*, vol. 23, no. 6, pp. 1203–1218, Dec. 2012, doi: 10.1016/j.devcel.2012.11.003.
[13] L. Herrnberger, K. Ebner, B. Junglas, and E. R. Tamm, “The role of plasmalemma vesicle-associated protein (PLVAP) in endothelial cells of Schlemm’s canal and ocular capillaries,” *Exp. Eye Res.*, vol. 105, pp. 27–33, Dec. 2012, doi: 10.1016/j.exer.2012.09.011.
[14] L. Herrnberger, R. Seitz, S. Kuespert, M. R. Bösl, R. Fuchshofer, and E. R. Tamm, “Lack of endothelial diaphragms in fenestrae and caveolae of mutant Plvap-deficient mice,” *Histochem. Cell Biol.*, vol. 138, no. 5, pp. 709–724, Nov. 2012, doi: 10.1007/s00418-012-0987-3.
[15] J. Wisniewska-Kruk *et al.*, “Molecular analysis of blood-retinal barrier loss in the Akimba mouse, a model of advanced diabetic retinopathy,” *Exp. Eye Res.*, vol. 122, pp. 123–131, May 2014, doi: 10.1016/j.exer.2014.03.005.
[16] M. Baba *et al.*, “Folliculin encoded by the BHD gene interacts with a binding protein, FNIP1, and AMPK, and is involved in AMPK and mTOR signaling,” *Proc. Natl. Acad. Sci. U. S. A.*, vol. 103, no. 42, pp. 15552–15557, Oct. 2006, doi: 10.1073/pnas.0603781103.
[17] Y. Hasumi *et al.*, “Homozygous loss of BHD causes early embryonic lethality and kidney tumor development with activation of mTORC1 and mTORC2,” *Proc. Natl. Acad. Sci. U. S. A.*, vol. 106, no. 44, pp. 18722–18727, Nov. 2009, doi: 10.1073/pnas.0908853106.
[18] M. Baba *et al.*, “Kidney-targeted Birt-Hogg-Dube gene inactivation in a mouse model: Erk1/2 and Akt-mTOR activation, cell hyperproliferation, and polycystic kidneys,” *J. Natl. Cancer Inst.*, vol. 100, no. 2, pp. 140–154, Jan. 2008, doi: 10.1093/jnci/djm288.
[19] Z.-Y. Tsun *et al.*, “The folliculin tumor suppressor is a GAP for the RagC/D GTPases that signal amino acid levels to mTORC1,” *Mol. Cell*, vol. 52, no. 4, pp. 495–505, Nov. 2013, doi: 10.1016/j.molcel.2013.09.016.
[20] F. H. Menko *et al.*, “Birt-Hogg-Dubé syndrome: diagnosis and management,” *Lancet Oncol.*, vol. 10, no. 12, pp. 1199–1206, Dec. 2009, doi: 10.1016/S1470-2045(09)70188-3.
[21] L. El-Houjeiri *et al.*, “Folliculin impairs breast tumor growth by repressing TFE3-dependent induction of the Warburg effect and angiogenesis,” *J. Clin. Invest.*, vol. 131, no. 22, p. e144871, Nov. 2021, doi: 10.1172/JCI144871.
[22] Y. Isono *et al.*, “FLCN alteration drives metabolic reprogramming towards nucleotide synthesis and cyst formation in salivary gland,” *Biochem. Biophys. Res. Commun.*, vol. 522, no. 4, pp. 931–938, Feb. 2020, doi: 10.1016/j.bbrc.2019.11.184.
[23] M. Yan *et al.*, “The tumor suppressor folliculin regulates AMPK-dependent metabolic transformation,” *J. Clin. Invest.*, vol. 124, no. 6, pp. 2640–2650, Jun. 2014, doi: 10.1172/JCI71749.
[24] H. Hasumi *et al.*, “Regulation of mitochondrial oxidative metabolism by tumor suppressor FLCN,” *J. Natl. Cancer Inst.*, vol. 104, no. 22, pp. 1750–1764, Nov. 2012, doi: 10.1093/jnci/djs418.
[25] M. Aregger *et al.*, “Systematic mapping of genetic interactions for de novo fatty acid synthesis identifies C12orf49 as a regulator of lipid metabolism,” *Nat. Metab.*, vol. 2, no. 6, pp. 499–513, Jun. 2020, doi: 10.1038/s42255-020-0211-z.
[26] S. E. Wiley, A. N. Murphy, S. A. Ross, P. van der Geer, and J. E. Dixon, “MitoNEET is an iron-containing outer mitochondrial membrane protein that regulates oxidative capacity,”

Proc. Natl. Acad. Sci. U. S. A., vol. 104, no. 13, pp. 5318–5323, Mar. 2007, doi: 10.1073/pnas.0701078104.
[27] Y.-F. Chen *et al.*, “Cisd2 deficiency drives premature aging and causes mitochondria-mediated defects in mice,” *Genes Dev.*, vol. 23, no. 10, pp. 1183–1194, May 2009, doi: 10.1101/gad.1779509.
[28] C.-Y. Wu *et al.*, “A persistent level of Cisd2 extends healthy lifespan and delays aging in mice,” *Hum. Mol. Genet.*, vol. 21, no. 18, pp. 3956–3968, Sep. 2012, doi: 10.1093/hmg/dds210.
[29] S. Amr *et al.*, “A homozygous mutation in a novel zinc-finger protein, ERIS, is responsible for Wolfram syndrome 2,” *Am. J. Hum. Genet.*, vol. 81, no. 4, pp. 673–683, Oct. 2007, doi: 10.1086/520961.
[30] M. Cagalinec *et al.*, “Role of Mitochondrial Dynamics in Neuronal Development: Mechanism for Wolfram Syndrome,” *PLoS Biol.*, vol. 14, no. 7, p. e1002511, Jul. 2016, doi: 10.1371/journal.pbio.1002511.
[31] F. F. Wagner *et al.*, “Weak D alleles express distinct phenotypes,” *Blood*, vol. 95, no. 8, pp. 2699–2708, Apr. 2000.
[32] Y. Colin, B. Chérif-Zahar, C. Le Van Kim, V. Raynal, V. Van Huffel, and J. P. Cartron, “Genetic basis of the RhD-positive and RhD-negative blood group polymorphism as determined by Southern analysis,” *Blood*, vol. 78, no. 10, pp. 2747–2752, Nov. 1991.
[33] P. G. Gallagher, “Hereditary elliptocytosis: spectrin and protein 4.1R,” *Semin. Hematol.*, vol. 41, no. 2, pp. 142–164, Apr. 2004, doi: 10.1053/j.seminhematol.2004.01.003.
[34] N. Mohandas and P. G. Gallagher, “Red cell membrane: past, present, and future,” *Blood*, vol. 112, no. 10, pp. 3939–3948, Nov. 2008, doi: 10.1182/blood-2008-07-161166.
[35] Y. Liu *et al.*, “A novel SPTB gene mutation in neonatal hereditary spherocytosis: A case report,” *Exp. Ther. Med.*, vol. 20, no. 4, pp. 3253–3259, Oct. 2020, doi: 10.3892/etm.2020.9062.
[36] S. Perrotta, P. G. Gallagher, and N. Mohandas, “Hereditary spherocytosis,” *Lancet Lond. Engl.*, vol. 372, no. 9647, pp. 1411–1426, Oct. 2008, doi: 10.1016/S0140-6736(08)61588-3.
[37] R. V. Stan, L. Ghitescu, B. S. Jacobson, and G. E. Palade, “Isolation, cloning, and localization of rat PV-1, a novel endothelial caveolar protein,” *J. Cell Biol.*, vol. 145, no. 6, pp. 1189–1198, Jun. 1999, doi: 10.1083/jcb.145.6.1189.
[38] S.-B. Hong, H. Oh, V. A. Valera, M. Baba, L. S. Schmidt, and W. M. Linehan, “Inactivation of the FLCN tumor suppressor gene induces TFE3 transcriptional activity by increasing its nuclear localization,” *PloS One*, vol. 5, no. 12, p. e15793, Dec. 2010, doi: 10.1371/journal.pone.0015793.
[39] J. Li, S. Wada, L. K. Weaver, C. Biswas, E. M. Behrens, and Z. Arany, “Myeloid Folliculin balances mTOR activation to maintain innate immunity homeostasis,” *JCI Insight*, vol. 5, no. 6, pp. e126939, 126939, Mar. 2019, doi: 10.1172/jci.insight.126939.
[40] C.-H. Yeh, Z.-Q. Shen, C.-C. Lin, C.-K. Lu, and T.-F. Tsai, “Rejuvenation: Turning Back Time by Enhancing CISD2,” *Int. J. Mol. Sci.*, vol. 23, no. 22, p. 14014, Nov. 2022, doi: 10.3390/ijms232214014.
[41] C. Rouzier *et al.*, “A novel CISD2 mutation associated with a classical Wolfram syndrome phenotype alters Ca2+ homeostasis and ER-mitochondria interactions,” *Hum. Mol. Genet.*, vol. 26, no. 9, pp. 1599–1611, May 2017, doi: 10.1093/hmg/ddx060.

[42] Z.-Q. Shen *et al.*, “CISD2 maintains cellular homeostasis,” *Biochim. Biophys. Acta BBA - Mol. Cell Res.*, vol. 1868, no. 4, p. 118954, Apr. 2021, doi: 10.1016/j.bbamcr.2021.118954.

[43] C. Huettmann *et al.*, “Iron Deficiency Caused by Intestinal Iron Loss-Novel Candidate Genes for Severe Anemia,” *Genes*, vol. 12, no. 12, p. 1869, Nov. 2021, doi: 10.3390/genes12121869.

[44] R. Mitra, N. Mishra, and G. P. Rath, “Blood groups systems,” *Indian J. Anaesth.*, vol. 58, no. 5, pp. 524–528, Sep. 2014, doi: 10.4103/0019-5049.144645.

[45] K. Finning, P. Martin, J. Summers, E. Massey, G. Poole, and G. Daniels, “Effect of high throughput RHD typing of fetal DNA in maternal plasma on use of anti-RhD immunoglobulin in RhD negative pregnant women: prospective feasibility study,” *BMJ*, vol. 336, no. 7648, pp. 816–818, Apr. 2008, doi: 10.1136/bmj.39518.463206.25.

[46] L. S. Chitty *et al.*, “Diagnostic accuracy of routine antenatal determination of fetal RHD status across gestation: population based cohort study,” *BMJ*, vol. 349, p. g5243, Sep. 2014, doi: 10.1136/bmj.g5243.

[47] L. Da Costa, J. Galimand, O. Fenneteau, and N. Mohandas, “Hereditary spherocytosis, elliptocytosis, and other red cell membrane disorders,” *Blood Rev.*, vol. 27, no. 4, pp. 167–178, Jul. 2013, doi: 10.1016/j.blre.2013.04.003.

[48] L. Da Costa *et al.*, “Diagnostic tool for red blood cell membrane disorders: Assessment of a new generation ektacytometer,” *Blood Cells. Mol. Dis.*, vol. 56, no. 1, pp. 9–22, Jan. 2016, doi: 10.1016/j.bcmd.2015.09.001.

[49] X.-X. Yan, A. Jeromin, and A. Jeromin, “Spectrin Breakdown Products (SBDPs) as Potential Biomarkers for Neurodegenerative Diseases,” *Curr. Transl. Geriatr. Exp. Gerontol. Rep.*, vol. 1, no. 2, pp. 85–93, Jun. 2012, doi: 10.1007/s13670-012-0009-2.

[50] A. Meglic *et al.*, “SPTB related spherocytosis in a three-generation family presenting with kidney failure in adulthood due to co-occurrence of UMOD disease causing variant,” *Nefrologia*, vol. 40, no. 4, pp. 421–428, 2020, doi: 10.1016/j.nefro.2019.10.009.

[51] L. A. Strickland *et al.*, “Plasmalemmal vesicle-associated protein (PLVAP) is expressed by tumour endothelium and is upregulated by vascular endothelial growth factor-A (VEGF),” *J. Pathol.*, vol. 206, no. 4, pp. 466–475, Aug. 2005, doi: 10.1002/path.1805.

[52] Y. Wen, Y. Wang, Y. Huang, Z. Liu, and C. Hui, “PLVAP protein expression correlated with microbial composition, clinicopathological features, and prognosis of patients with stomach adenocarcinoma,” *J. Cancer Res. Clin. Oncol.*, vol. 149, no. 10, pp. 7139–7153, Aug. 2023, doi: 10.1007/s00432-023-04607-3.

[53] L. De Leo *et al.*, “Circulating PV-1 as a marker of celiac disease-associated liver injury,” *Biomark. Med.*, vol. 14, no. 18, pp. 1675–1681, Dec. 2020, doi: 10.2217/bmm-2020-0281.

[54] I. Klaassen, J. M. Hughes, I. M. C. Vogels, C. G. Schalkwijk, C. J. F. Van Noorden, and R. O. Schlingemann, “Altered expression of genes related to blood-retina barrier disruption in streptozotocin-induced diabetes,” *Exp. Eye Res.*, vol. 89, no. 1, pp. 4–15, Jun. 2009, doi: 10.1016/j.exer.2009.01.006.

[55] M. L. Nickerson *et al.*, “Mutations in a novel gene lead to kidney tumors, lung wall defects, and benign tumors of the hair follicle in patients with the Birt-Hogg-Dubé syndrome,” *Cancer Cell*, vol. 2, no. 2, pp. 157–164, Aug. 2002, doi: 10.1016/s1535-6108(02)00104-6.

[56] L. S. Schmidt and W. M. Linehan, “FLCN: The causative gene for Birt-Hogg-Dubé syndrome,” *Gene*, vol. 640, pp. 28–42, Jan. 2018, doi: 10.1016/j.gene.2017.09.044.
[57] L. S. Schmidt and W. M. Linehan, “Molecular genetics and clinical features of Birt-Hogg-Dubé syndrome,” *Nat. Rev. Urol.*, vol. 12, no. 10, pp. 558–569, Oct. 2015, doi: 10.1038/nrurol.2015.206.
[58] E. Bandini *et al.*, “A Novel FLCN Variant in a Suspected Birt-Hogg-Dubè Syndrome Patient,” *Int. J. Mol. Sci.*, vol. 24, no. 15, p. 12418, Aug. 2023, doi: 10.3390/ijms241512418.
[59] Q.-Q. Zhu, L. Tian, D.-L. Li, Z.-H. Wu, Y.-Y. He, and H.-K. Zhang, “Elevated CISD2 expression predicts poor diagnosis and promotes invasion and migration of prostate cancer cells,” *Eur. Rev. Med. Pharmacol. Sci.*, vol. 24, no. 12, pp. 6597–6604, Jun. 2020, doi: 10.26355/eurrev_202006_21645.
[60] H.-Y. Liao, B. Liao, and H.-H. Zhang, “CISD2 plays a role in age-related diseases and cancer,” *Biomed. Pharmacother. Biomedecine Pharmacother.*, vol. 138, p. 111472, Jun. 2021, doi: 10.1016/j.biopha.2021.111472.
[61] Z.-Q. Shen *et al.*, “CISD2 Haploinsufficiency Disrupts Calcium Homeostasis, Causes Nonalcoholic Fatty Liver Disease, and Promotes Hepatocellular Carcinoma,” *Cell Rep.*, vol. 21, no. 8, pp. 2198–2211, Nov. 2017, doi: 10.1016/j.celrep.2017.10.099.
[62] W.-M. Kung *et al.*, “Anti-Inflammatory CDGSH Iron-Sulfur Domain 2: A Biomarker of Central Nervous System Insult in Cellular, Animal Models and Patients,” *Biomedicines*, vol. 10, no. 4, p. 777, Mar. 2022, doi: 10.3390/biomedicines10040777.
[63] S.-M. Li *et al.*, “Upregulation of CISD2 augments ROS homeostasis and contributes to tumorigenesis and poor prognosis of lung adenocarcinoma,” *Sci. Rep.*, vol. 7, no. 1, p. 11893, Sep. 2017, doi: 10.1038/s41598-017-12131-x.
[64] J. A. Sugrue *et al.*, “Rhesus negative males have an enhanced IFNγ-mediated immune response to influenza A virus,” *Genes Immun.*, vol. 23, no. 2, pp. 93–98, Apr. 2022, doi: 10.1038/s41435-022-00169-5.
[65] Z. Zhang *et al.*, “Accurate long-read sequencing allows assembly of the duplicated RHD and RHCE genes harboring variants relevant to blood transfusion,” *Am. J. Hum. Genet.*, vol. 109, no. 1, pp. 180–191, Jan. 2022, doi: 10.1016/j.ajhg.2021.12.003.
[66] K. Mukherjee and J. J. Bieker, “Transcriptional Control of Gene Expression and the Heterogeneous Cellular Identity of Erythroblastic Island Macrophages,” *Front. Genet.*, vol. 12, p. 756028, 2021, doi: 10.3389/fgene.2021.756028.
[67] A. Su, X. Chen, Z. Zhang, B. Xu, C. Wang, and Z. Xu, “Integrated transcriptomic and metabolomic analysis of rat serum to investigate potential target of puerarin in the treatment post-traumatic stress disorder,” *Ann. Transl. Med.*, vol. 9, no. 24, p. 1771, Dec. 2021, doi: 10.21037/atm-21-6009.
[68] A. C. Wilson *et al.*, “Heme metabolism genes Downregulated in COPD Cachexia,” *Respir. Res.*, vol. 21, no. 1, p. 100, May 2020, doi: 10.1186/s12931-020-01336-w.
[69] J. Keuschnigg, T. Henttinen, K. Auvinen, M. Karikoski, M. Salmi, and S. Jalkanen, “The prototype endothelial marker PAL-E is a leukocyte trafficking molecule,” *Blood*, vol. 114, no. 2, pp. 478–484, Jul. 2009, doi: 10.1182/blood-2008-11-188763.
[70] J. Wisniewska-Kruk *et al.*, “A novel co-culture model of the blood-retinal barrier based on primary retinal endothelial cells, pericytes and astrocytes,” *Exp. Eye Res.*, vol. 96, no. 1, pp. 181–190, Mar. 2012, doi: 10.1016/j.exer.2011.12.003.

[71] E. K. Bosma, C. J. F. van Noorden, R. O. Schlingemann, and I. Klaassen, “The role of plasmalemma vesicle-associated protein in pathological breakdown of blood-brain and blood-retinal barriers: potential novel therapeutic target for cerebral edema and diabetic macular edema,” *Fluids Barriers CNS*, vol. 15, no. 1, p. 24, Sep. 2018, doi: 10.1186/s12987-018-0109-2.

[72] L. S. Schmidt *et al.*, “Germline BHD-mutation spectrum and phenotype analysis of a large cohort of families with Birt-Hogg-Dubé syndrome,” *Am. J. Hum. Genet.*, vol. 76, no. 6, pp. 1023–1033, Jun. 2005, doi: 10.1086/430842.

[73] I. Tai-Nagara *et al.*, “Blood and lymphatic systems are segregated by the FLCN tumor suppressor,” *Nat. Commun.*, vol. 11, no. 1, p. 6314, Dec. 2020, doi: 10.1038/s41467-020-20156-6.

[74] H. Li, X. Wang, Q. Yang, L. Cheng, and H.-L. Zeng, “Identification of iron metabolism-related genes as diagnostic signatures in sepsis by blood transcriptomic analysis,” *Open Life Sci.*, vol. 18, no. 1, p. 20220549, 2023, doi: 10.1515/biol-2022-0549.

[75] A. M. Hall, M. A. Vickers, E. McLeod, and R. N. Barker, “Rh autoantigen presentation to helper T cells in chronic lymphocytic leukemia by malignant B cells,” *Blood*, vol. 105, no. 5, pp. 2007–2015, Mar. 2005, doi: 10.1182/blood-2003-10-3563.

[76] L. Della Valle, S. E. Dohmen, O. J. H. M. Verhagen, M. A. Berkowska, G. Vidarsson, and C. Ellen van der Schoot, “The majority of human memory B cells recognizing RhD and tetanus resides in IgM+ B cells,” *J. Immunol. Baltim. Md 1950*, vol. 193, no. 3, pp. 1071–1079, Aug. 2014, doi: 10.4049/jimmunol.1400706.

[77] C. W. Cairo *et al.*, “Dynamic regulation of CD45 lateral mobility by the spectrin-ankyrin cytoskeleton of T cells,” *J. Biol. Chem.*, vol. 285, no. 15, pp. 11392–11401, Apr. 2010, doi: 10.1074/jbc.M109.075648.

[78] D. Pradhan and J. Morrow, “The spectrin-ankyrin skeleton controls CD45 surface display and interleukin-2 production,” *Immunity*, vol. 17, no. 3, pp. 303–315, Sep. 2002, doi: 10.1016/s1074-7613(02)00396-5.

[79] M. Baba *et al.*, “The folliculin-FNIP1 pathway deleted in human Birt-Hogg-Dubé syndrome is required for murine B-cell development,” *Blood*, vol. 120, no. 6, pp. 1254–1261, Aug. 2012, doi: 10.1182/blood-2012-02-410407.

[80] W. A. Flegel, “Molecular genetics and clinical applications for RH,” *Transfus. Apher. Sci. Off. J. World Apher. Assoc. Off. J. Eur. Soc. Haemapheresis*, vol. 44, no. 1, pp. 81–91, Feb. 2011, doi: 10.1016/j.transci.2010.12.013.

[81] G. Daniels, “Variants of RhD--current testing and clinical consequences,” *Br. J. Haematol.*, vol. 161, no. 4, pp. 461–470, May 2013, doi: 10.1111/bjh.12275.

[82] M. R. Woodford *et al.*, “The FNIP co-chaperones decelerate the Hsp90 chaperone cycle and enhance drug binding,” *Nat. Commun.*, vol. 7, p. 12037, Jun. 2016, doi: 10.1038/ncomms12037.

[83] S. Tamir *et al.*, “Nutrient-deprivation autophagy factor-1 (NAF-1): biochemical properties of a novel cellular target for anti-diabetic drugs,” *PloS One*, vol. 8, no. 5, p. e61202, 2013, doi: 10.1371/journal.pone.0061202.

[84] F. Y. Chan *et al.*, “Prenatal RHD gene determination and dosage analysis by PCR: clinical evaluation,” *Prenat. Diagn.*, vol. 21, no. 4, pp. 321–326, Apr. 2001, doi: 10.1002/pd.60.

[85] J. M. Despotovic, P. T. McGann, M. Smeltzer, B. Aygun, and R. E. Ware, “RHD zygosity predicts degree of platelet response to anti-D immune globulin treatment in children with immune thrombocytopenia,” *Pediatr. Blood Cancer*, vol. 60, no. 9, pp. E106-108, Sep. 2013, doi: 10.1002/pbc.24574.
[86] E. Hussein and J. Teruya, “Serologic findings of RhD alleles in Egyptians and their clinical implications,” *Transfus. Apher. Sci. Off. J. World Apher. Assoc. Off. J. Eur. Soc. Haemapheresis*, vol. 51, no. 2, pp. 184–187, Oct. 2014, doi: 10.1016/j.transci.2014.08.014.
[87] I. von Zabern, F. F. Wagner, J. M. Moulds, J. J. Moulds, and W. A. Flegel, “D category IV: a group of clinically relevant and phylogenetically diverse partial D,” *Transfusion (Paris)*, vol. 53, no. 11 Suppl 2, pp. 2960–2973, Nov. 2013, doi: 10.1111/trf.12145.
[88] H. Ansart-Pirenne, M. Asso-Bonnet, P.-Y. Le Pennec, M. Roussel, C. Patereau, and F. Noizat-Pirenne, “RhD variants in Caucasians: consequences for checking clinically relevant alleles,” *Transfusion (Paris)*, vol. 44, no. 9, pp. 1282–1286, Sep. 2004, doi: 10.1111/j.1537-2995.2004.04063.x.
[89] C. M. Westhoff *et al.*, “RHCE*ceMO is frequently in cis to RHD*DAU0 and encodes a hr(S) -, hr(B) -, RH:-61 phenotype in black persons: clinical significance,” *Transfusion (Paris)*, vol. 53, no. 11 Suppl 2, pp. 2983–2989, Nov. 2013, doi: 10.1111/trf.12271.
[90] S. Hu, D. Jue, J. Albanese, Y. Wang, and Q. Liu, “Utilization of spectrins βI and βIII in diagnosis of hepatocellular carcinoma,” *Ann. Diagn. Pathol.*, vol. 39, pp. 86–91, Apr. 2019, doi: 10.1016/j.anndiagpath.2019.02.009.
[91] Z. Du, G. Luo, K. Wang, Z. Bing, and S. Pan, “Identification of a novel heterozygous SPTB mutation by whole genome sequencing in a Chinese patient with hereditary spherocytosis and atrial septal defect: a case report,” *BMC Pediatr.*, vol. 21, no. 1, p. 291, Jun. 2021, doi: 10.1186/s12887-021-02771-4.
[92] Y. Hasumi *et al.*, “Folliculin (Flcn) inactivation leads to murine cardiac hypertrophy through mTORC1 deregulation,” *Hum. Mol. Genet.*, vol. 23, no. 21, pp. 5706–5719, Nov. 2014, doi: 10.1093/hmg/ddu286.
[93] W.-M. Kung and M.-S. Lin, “The NFκB Antagonist CDGSH Iron-Sulfur Domain 2 Is a Promising Target for the Treatment of Neurodegenerative Diseases,” *Int. J. Mol. Sci.*, vol. 22, no. 2, p. 934, Jan. 2021, doi: 10.3390/ijms22020934.
[94] Y. Yang, Y.-S. Bai, and Q. Wang, “CDGSH Iron Sulfur Domain 2 Activates Proliferation and EMT of Pancreatic Cancer Cells via Wnt/β-Catenin Pathway and Has Prognostic Value in Human Pancreatic Cancer,” *Oncol. Res.*, vol. 25, no. 4, pp. 605–615, Apr. 2017, doi: 10.3727/096504016X14767450526417.
[95] L. Yang *et al.*, “A novel prognostic score model incorporating CDGSH iron sulfur domain2 (CISD2) predicts risk of disease progression in laryngeal squamous cell carcinoma,” *Oncotarget*, vol. 7, no. 16, pp. 22720–22732, Apr. 2016, doi: 10.18632/oncotarget.8150.
[96] B. Chen *et al.*, “CISD2 associated with proliferation indicates negative prognosis in patients with hepatocellular carcinoma,” *Int. J. Clin. Exp. Pathol.*, vol. 8, no. 10, pp. 13725–13738, 2015.
[97] L. Wang *et al.*, “Overexpressed CISD2 has prognostic value in human gastric cancer and promotes gastric cancer cell proliferation and tumorigenesis via AKT signaling pathway,” *Oncotarget*, vol. 7, no. 4, pp. 3791–3805, Jan. 2016, doi: 10.18632/oncotarget.6302.

[98] L. Liu, M. Xia, J. Wang, W. Zhang, Y. Zhang, and M. He, "CISD2 expression is a novel marker correlating with pelvic lymph node metastasis and prognosis in patients with early-stage cervical cancer," *Med. Oncol. Northwood Lond. Engl.*, vol. 31, no. 9, p. 183, Sep. 2014, doi: 10.1007/s12032-014-0183-5.
[99] N. Novershtern *et al.*, "Densely interconnected transcriptional circuits control cell states in human hematopoiesis," *Cell*, vol. 144, no. 2, pp. 296–309, Jan. 2011, doi: 10.1016/j.cell.2011.01.004.
[100] P. S. Linsley, C. Speake, E. Whalen, and D. Chaussabel, "Copy number loss of the interferon gene cluster in melanomas is linked to reduced T cell infiltrate and poor patient prognosis," *PloS One*, vol. 9, no. 10, p. e109760, 2014, doi: 10.1371/journal.pone.0109760.
[101] D. Rinchai and D. Chaussabel, "Assessing the potential relevance of CEACAM6 as a blood transcriptional biomarker," *F1000Research*, vol. 11, p. 1294, Nov. 2022, doi: 10.12688/f1000research.126721.1.
[102] D. Rinchai and D. Chaussabel, "A training curriculum for retrieving, structuring, and aggregating information derived from the biomedical literature and large-scale data repositories.," *F1000Research*, vol. 11, p. 994, Sep. 2022, doi: 10.12688/f1000research.122811.1.
